# Efficient molecular motors in liquid crystal networks enable the integration of fluorescence with large opto-mechanical effects

**DOI:** 10.1038/s41467-026-76177-0

**Published:** 2026-07-30

**Authors:** Guiying Long, Jiahui Meng, Alexander Ryabchun, Ben L. Feringa

**Affiliations:** 1 Stratingh Institute for Chemistry, University of Groningen, Nijenborgh 3, 9747 AG Groningen, The Netherlands; 2 SCNU-UG International Joint Laboratory of Molecular Science and Displays, National Center for International Research on Green Optoelectronics, South China Normal University, Guangzhou, 510006 China

**Keywords:** Liquid crystals, Polymer synthesis, Polymers

## Abstract

Liquid crystal polymer networks (LCPNs) exhibit remarkable light-responsive actuation, yet the molecular-level design rules governing their performance remain elusive. Here, we develop a series of molecular motor-based photo-responsive units with tunable rigidity, and different substituents, enabling precise modulation of LCPN mechanics and photo-responsive behavior. By systematic study and comparing these motors with conventional azobenzene and second-generation molecular motors, we establish clear structure-property relationships that link molecular design to macroscopic actuation efficiency and network stiffness. Notably, our motor-integrated LCPNs also exhibit intrinsic fluorescence, enabling shape-encoded pattern visualization without the need for additional fluorescent molecules. This multifunctional liquid crystal-motor hybrid system integrates light-induced actuation, mechanical tunability, and fluorescence signaling, offering design principles for next-generation soft actuators and intelligent photonic devices.

## Abstract (web summary)

Liquid crystal polymer networks exhibit light triggered actuation, though controlling their macroscopic behavior through molecular design remains challenging. Here the authors integrate molecular motors into the network to tune photoactuation, mechanical properties and fluorescence response.

## Introduction

In recent years, liquid crystal polymer networks (LCPNs) have emerged as ideal material systems for the construction of advanced soft actuators and photonic devices, owing to their unique combination of anisotropy, molecular order, and tunable mechanical properties^1–3^. By integrating the self-assembly capabilities of liquid crystals with the elastic responsiveness of polymer networks, LCPNs exhibit rapid and reversible shape transformations under external stimuli, particularly light^4–6^. This molecular-to-macroscopic cooperative response mechanism confers LCPNs with significant potential in a wide range of applications, including adaptive actuators, tunable photonic crystals, dynamic displays, and complex bioinspired systems^7–12^.

The core strategy for achieving light-driven reversible deformation in LCPNs relies on the integration of photo-isomerizable units such as azobenzenes^9,13–17^, diarylethenes^18–20^, hydrazones^21^, and molecular motors^22–27^. These responsive units undergo specific structural transformations upon exposure to light of appropriate wavelengths, enabling the system to directly convert light energy into mechanical deformation, thereby efficiently amplifying molecular operation to macroscopic functional output. Among these, light-driven molecular motors, with their unique structural dynamics and high programmability, are regarded as extremely promising units for the next generation of highly efficient light-responsive materials^28–30^. Nevertheless, despite the increasing number of reports on LCPN-based systems, there are major challenges, especially significant knowledge gaps in the material design and in the understanding of structure-property relationships. First, there is a lack of systematic comparative studies on different photo-responsive units within LCPNs, particularly concerning their actuation performance and mechanical behavior. This makes it difficult to evaluate the effectiveness of distinct molecular design strategies, which limits the further development of light-responsive LCPN’s. Second, although molecular motors exhibit high structural design flexibility, the mechanisms by which their chemical configurations influence the network structure, mechanical stiffness, and energy conversion efficiency of LCPNs remain poorly understood and lack experimental validation. Consequently, this has imposed constraints on the precise tuning and optimization of the LCPN performance. Furthermore, most existing LCPN systems focus on single-mode functionality. Realizing multifunctional integration typically requires complex composite systems, which introduces major synthesis and processing challenges and material compatibility issues^15,31–33^.

**Fig. 1:**
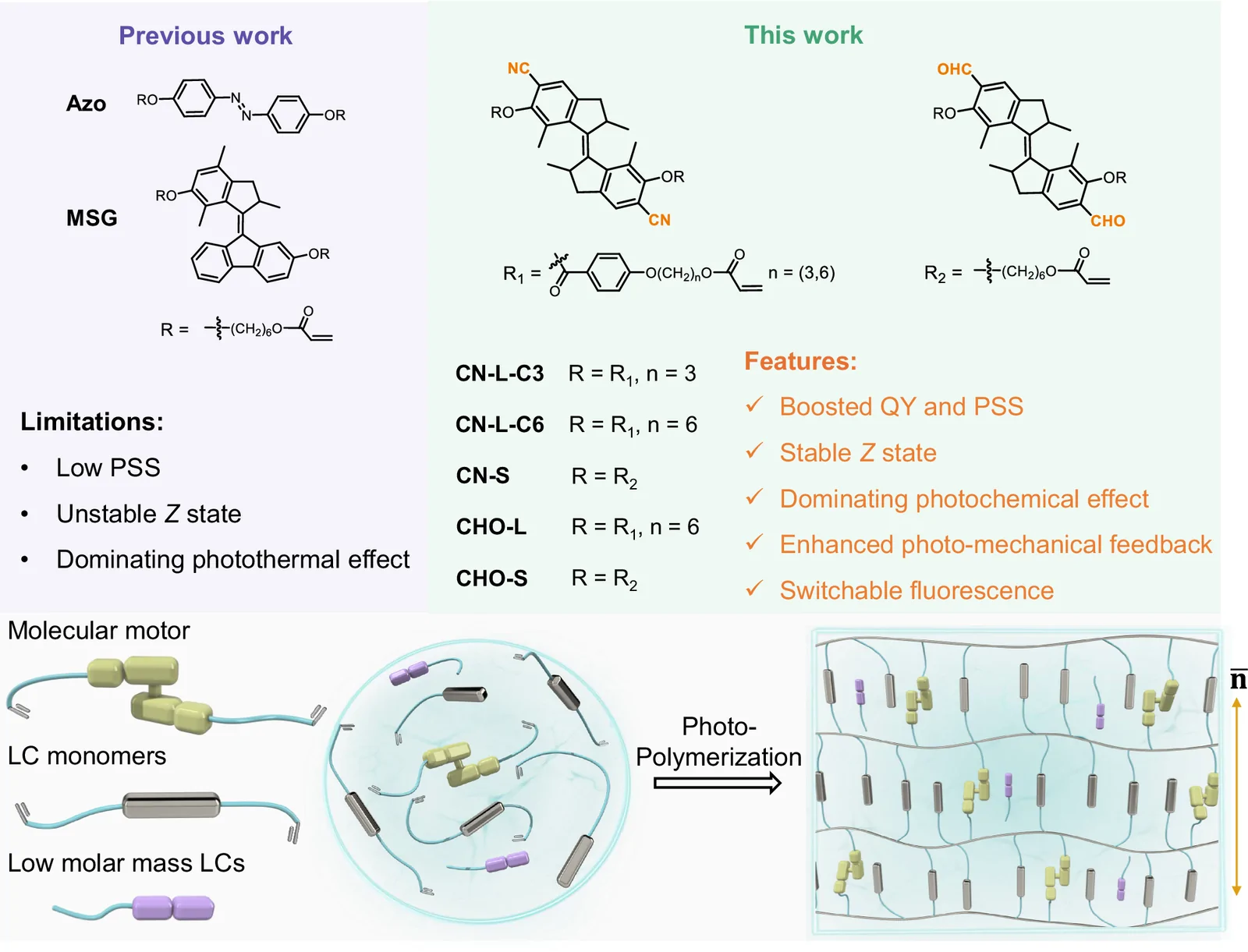
Motorized liquid crystal polymer network. Previous LCPN systems with molecular optical switches, including azobenzene (**Azo**) and second-generation motor (**MSG**), suffer from limitations such as low photostationary state (PSS), switching rates, unstable *Z* state, and pronounced photothermal effects (left panel). The designed photo-responsive polymerizable units contain highly efficient cyano (CN) and aldehyde (CHO) functionalized first-generation molecular motors, exhibiting enhanced quantum yield (QY) and PSS, improved opto-mechanical feedback, and switchable fluorescence properties (right panel). The bottom panel shows a schematic representation of the molecular motor-embedded LCPN. The molecular components undergo a photo-polymerization to form a crosslinked polymer network with specific molecular alignment ($\bar{{{{\bf{n}}}}}$).

To address these challenges, in this study, we designed and synthesized a series of novel molecular motors based on precise structural regulation at the molecular level and systematically investigated their effect on LCPNs performance. Here, we explore different molecular motor crosslinkers by tuning of motor cores with extended rigidity, flexible chain lengths, and functional group electronic effects. Meanwhile, more frequently used molecular motors of the second-generation (characterized by lower quantum yields, less favorable positions of the photostationary state, and faster rotation rates compared to first-generation motors)^30,34^ as well as a conventional azobenzene photoswitch were used as control, enabling systematic comparisons of mechanical properties, actuation performance, and functional outputs (Fig. 1). Through a comprehensive structure-property analysis, we revealed the profound influence of the molecular structure of the light-responsive units on the macroscopic behavior of LCPNs, particularly highlighting the cooperative coupling between photomechanical conversion efficiency and network stiffness. These insights provide fundamental guidance for the rational design of high-performance LCPNs. Moreover, benefiting from the intrinsic fluorescence of the motor core, the resulting LCPNs not only exhibit light-driven deformation and tunable mechanical responses, but also provide stable fluorescence, achieving synergistic integration of mechanical responses and light signal readout within a single material system. Therefore, this study presents a molecular engineering strategy for constructing multifunctional, integrated, and intelligent LCPNs, offering a promising design concept for next-generation responsive materials.

## Results and discussion

### Material design

According to our recent studies^35,36^, bis-aldehyde molecular motors have demonstrated superior performance compared to traditional first-generation molecular motor structures, showing advantages such as red-shifted absorption, higher photo-efficiency, and improved PSS. The corresponding LCPN systems also exhibited enhanced macroscopic performance. To further understand how molecular structure influences LCPN behavior and to enhance the functionality of motors within such networks, this study carried out a systematic structural optimization and extension design based on first-generation molecular motors.

Specifically, we constructed a series of photo-responsive molecular units by tuning three key structural elements: (1) the type of electronic substituent groups (-CN and -CHO); (2) the length of flexible chain segments (spacers); (3) the rigid extension of the motor core. Firstly, strong electron-withdrawing groups were introduced such as cyano or aldehyde groups into the motor core, which contribute to the enhancement of the intermolecular weak interactions and thus improve the mechanical properties of the LCPN system^37–40^ as it will be further discussed. Acrylate terminal groups were also appended onto the motor molecules to act as covalent crosslinkers to facilitate copolymerization with LC monomers. Secondly, we designed flexible spacer chains of different lengths (*n* = 3 or 6) to connect the motor core to the acrylate groups, both to provide enough freedom for the motor to rotate in the polymer matrix and to explore the effect of chain length on the network modulus and responsiveness. Finally, to explore the role of the rigid extended structure of the motor core on the opto-mechanical performance, we introduced a benzene ring between the flexible chain and the motor core via ester bonds to increase the magnitude of geometrical change of the motor during the isomerization process, potentially amplifying the mechanical response. This design strategy not only enhances the functional properties and compatibility of the molecular motors in polymer networks but also enables a systematic investigation of how molecular-level modifications impact LCPN performance, which can help deepen the understanding of the motor-driven actuation mechanism and serve as valuable guidance for future molecular design in motorized soft materials.

Here, we designed and selected the light-driven molecular motors **CN-L-C3, CN-L-C6,** **CN-S,** **CHO-L**, and **CHO-S**, and then co-polymerized them as covalent cross-linkers in LCPNs consisting of mono-acrylate monomers (**C6BP,** **C6BP6**) and a diacrylate (**C6M**), thereby enabling the effective transduction of molecular motion to macroscopic deformation. In order to reduce the elastic modulus of the LCPNs, a non-polymerizable low molar mass LC mixture (**E7)** acting as plasticizer^41,42^ was also added. In addition, commonly used photo-responsive units including azobenzene (**Azo**) and previously reported second-generation motor (**MSG**)^25^ were used as control compounds to compare their performance. The chemical structures of all photo-responsive units are shown in Fig. 1, and the corresponding LCPN compositions are summarized in Supplementary Table 3. Synthesis and characterization of all motorized units are provided in SI (sections 2 and 3). Details of **CHO-L** are available from our previous work^43^.

### Rotatory behavior of molecular motor in solution

**Fig. 2:**
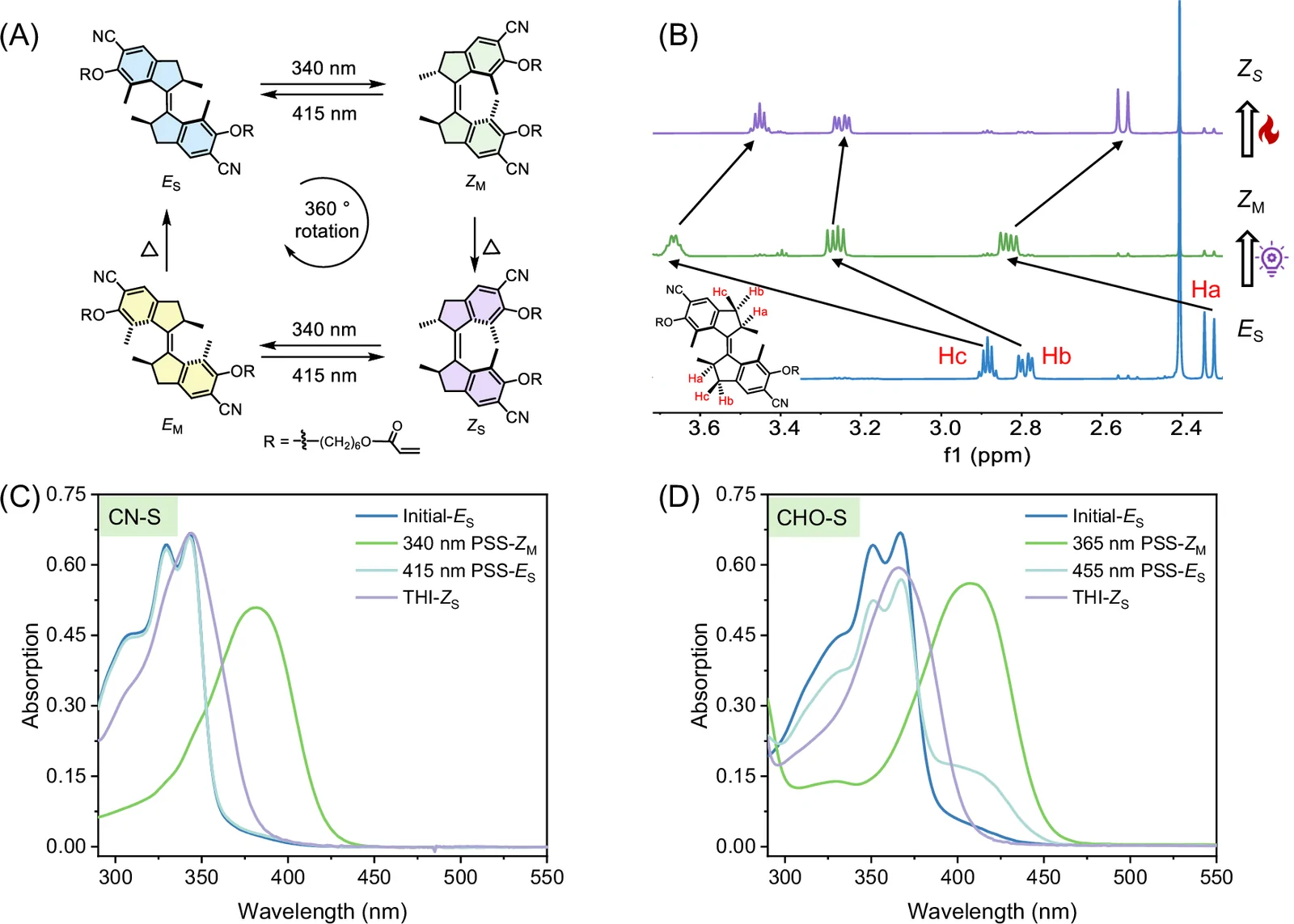
Characterization of functionalized motors rotation in solution. **A** Full rotational cycle of molecular motors **CN-S**, which consists of two photochemical steps of *E*/*Z* isomerization followed THI. **B** Partial ^1^H-NMR of **CN-S** in different states (600 MHz, CD_2_Cl_2_, 25 °C). The initial *E*_S_-isomer (blue line) was converted to *Z*_M_ (green line) exposure to 340 nm light for 90 min. The THI to *Z*_S_ (purple line) was performed by keeping the sample at r.t. in the dark for 4 days. **C CN-S*-****E*_S_ (dark blue line) undergoes photo-isomerization to the *Z*_M_ state (green line) upon 340 nm UV irradiation, followed by recovery to the photostationary *E*_S_ state (light blue line) upon 415 nm irradiation (5 × 10^-5 ^M, THF, 20 °C). The motor **CN-S***-Z*_M_ undergoes a thermal helix inversion (THI) to form the *Z*_S_ state (purple line) (5×10^-5 ^M, toluene, 60 °C, 3.5 h). **D CHO-S** follows a similar process: 365 nm UV light converts the *E*_S_ state (dark blue line) to the *Z*_M_ state (green line), which then reverts under 455 nm light (light blue line) (5 × 10^-5 ^M, THF, 20 °C). The **CHO-S***-Z*_M_ undergoes THI to form the *Z*_S_ state (purple line) (5 × 10^-5 ^M, toluene, 60 °C, 5.5 h).

The designed molecular motors were able to undergo a four-step unidirectional rotation under external stimuli, which included two photochemical isomerization processes, each followed by a thermal helix inversion (THI) step, completing a 360° unidirectional rotational cycle (Fig. 2A), as confirmed by ^1^H-NMR and UV-vis spectroscopy.

Taking the motor **CN-S** as an example, Fig. 2B shows the partial ^1^H-NMR spectra of **CN-S** in different states. The prominent characteristic signal peaks of the first-generation motor molecule are the protons Ha, Hb, and Hc. The protons Ha, Hb, and Hc correspond to signals at 2.88 ppm, 2.79 ppm, and 2.33 ppm, respectively, as indicated in Fig. 2B (blue line), which is attributed to different chemical environments resulting from the conformation of the five-membered ring. The sample was then irradiated with UV light (λ = 340 nm) at room temperature until it reached the photostationary state (PSS), resulting in a significant change in the spectra, indicating the formation of a new isomer *Z*_M_ (Fig. 2B, green line). The new peaks at 3.66 ppm, 3.26 ppm, and 2.83 ppm were attributed to the Ha, Hb, and Hc protons of *Z*_M_, respectively. Quantification of the isomer conversion ratio by integration yielded a ratio of *Z*_M_ to *E*_S_ of 90:10. The samples were subsequently stored in a dark environment for 4 d, and the characteristic peaks were further shifted to 3.45 ppm, 3.25 ppm, and 2.55 ppm, respectively (Fig. 2B, purple line). This indicates that *Z*_M_ was converted to *Z*_S_ by THI process. Spectral analysis showed that the ratio of *Z*_S_ to *Z*_M_ was more than 99:1, confirming nearly complete thermal conversion under these conditions. Similarly, motors **CN-L-C3** and **CN-L-C6** undergo photoisomerization from *E*_S_ to *Z*_M_ under irradiation of UV light at 340 nm and the ratio of *Z*_M_ to *E*_S_ is 97:3 and 95:5 at PSS, respectively, and then the motor**s** can be rotated almost completely from *Z*_M_ to *Z*_S_ after the sample is kept in a dark environment for 3 weeks (Supplementary Fig. 35b, c). The motor **CHO-S** underwent *E*_S_ → *Z*_M_ photoisomerization under 365 nm irradiation, reaching a *Z*_M_: *E*_S_ ratio of 97:3 at PSS. After storage in the dark for 5 d, the *Z*_M_ isomer almost completely converted to *Z*_S_-isomer (Supplementary Fig. 35d).

The above rotatory behavior was further verified by UV-vis spectroscopy. The initial absorption bands (330 nm and 343 nm) of **CN-S** gradually decreased under 340 nm irradiation and a new absorption band centered at 382 nm was formed, which associates with photochemical formation of *Z*_M_, and this process was reversible under blue light (λ = 415 nm) irradiation (Fig. 2C). The THI process from *Z*_M_ to *Z*_S_ can also be monitored in real time by UV-vis spectroscopy, ultimately resulting in the formation of an absorption band centered at 344 nm. Kinetic studies and Eyring analysis allowed us to estimate the standard Gibbs free energy of activation (Δ^‡^*G*^о^) for the *Z*_M_ → *Z*_S_ transition as 102.8 kJ/mol. The half-life time (*t*_1/2_) of *Z*_M_ at room temperature was calculated to be ~67 h (Supplementary Fig. 37a, b and Supplementary Table 1). Similar studies were carried out for **CHO-S** (Fig. 2D, Supplementary Fig. 37g, h and Supplementary Table 1), **CN-L-C3** (Supplementary Fig. 36a, Supplementary Fig. 37c, d and Supplementary Table 1) and **CN-L-C6** (Supplementary Fig. 36b, Supplementary Fig. 37e, f and Supplementary Table 1), and the results revealed that Δ^‡^*G*^о^ for the *Z*_M_ → *Z*_S_ transition were 103.7, 106.9, and 107.5 kJ/mol, corresponding to half-life time of approximately 97, 359, and 354 h at room temperature, respectively.

These values are similar to those obtained from structurally related motors. Compared to the unsubstituted first-generation molecular motors^27^, their UV absorption is red-shifted, and kinetic studies indicate that **CN-S-***Z*_M_, **CHO-S-***Z*_M_**, CN-L-C3-***Z*_M_ and **CN-L-C6-***Z*_M_ are relatively stable, which is advantageous for our material design. Additionally, the quantum yield (QY) of the photochemical isomerization of the motors **CN-S,** **CHO-S, CN-L-C3** and **CN-L-C6** (*E*_S_ → *Z*_M_) were determined to be about 11%, 85%, 13% and 7%, respectively (see experimental details in Supplementary Fig. 38 and Supplementary Table 2). Combined photochemical data for all tested cyano and aldehyde motors in solution is provided in Table 1. It is found that the rigid extension of motor core structure leads to significant increase of *t*_1/2_ indicating higher stability of the *Z*_M_, which goes in line with our previous observations^44^. Moreover, the PSSs of all the motors under UV light exceed 90%, indicating that the photo-isomerization process is highly efficient which is favorable for their application in materials. In addition, the quantum yield of the photochemical isomerization (*E*_S_ → *Z*_M_) process of the aldehyde-based motors is significantly higher than that of the cyano motors, which exhibits a superior photo-efficiency.

**Table 1:** The half-life, photostationary state ratio, and quantum yield of the first-generation molecular motor derivatives functionalized with cyano and aldehyde groups

	*t*_1/2_ (h)	PSS (*Z*_M_: *E*_S_)	*Ф* (*E*_S_→*Z*_M_)	*Ф* (*Z*_M_→*E*_S_)
CHO-L ^a^	437.8	97:3	71.2% ± 5.4%	9.2% ± 1.7%
CHO-S ^a^	96.7	97:3	84.6% ± 1.5%	16.3% ± 0.4%
CN-L-C3 ^b^	359.0	97:3	13.0% ± 0.7%	1.4% ± 0.7%
CN-L-C6 ^b^	353.5	95:5	6.8% ± 0.2%	1.6% ± 0.04%
CN-S ^b^	66.9	90:10	10.8% ± 0.5%	5.6% ± 0.5%

^[a]^ and ^[b]^ were studied under exposure to 365 nm and 340 nm light, respectively.

The above spectroscopic data confirm that both cyano and aldehyde-modified molecular motor motifs can achieve controlled unidirectional rotation in solution. These motors feature red-shifted absorption, *Z*_M_ stability, high QY (therefore fast response), making them ideal candidates for integration into liquid crystal-based soft actuators and efficiently driving macroscopic deformation.

### Light-driven deformations of motorized networks

Previous studies have demonstrated that the rotary motion of molecular motors can be effectively amplified from the molecular to the macroscopic scale, enabling controllable deformation of materials. Building on these findings and the systematic investigation of the photo-responsive behavior of the motors in solution, we conducted a comparative study to further explore how structural differences in photo-responsive units influence the photomechanical performance of LCPNs. LCPN films with planar alignment were prepared by proportionally mixing structurally different photo-responsive moieties with LC monomers (**C6M, C6BP, C6BP6**) and a non-polymerized low molar mass LC mixture **E7**, and inducing polymerization under 470 nm illumination in the presence of photoinitiator. The structures of all the compounds and the preparation process are shown in Supplementary Table 3.

**Fig. 3:**
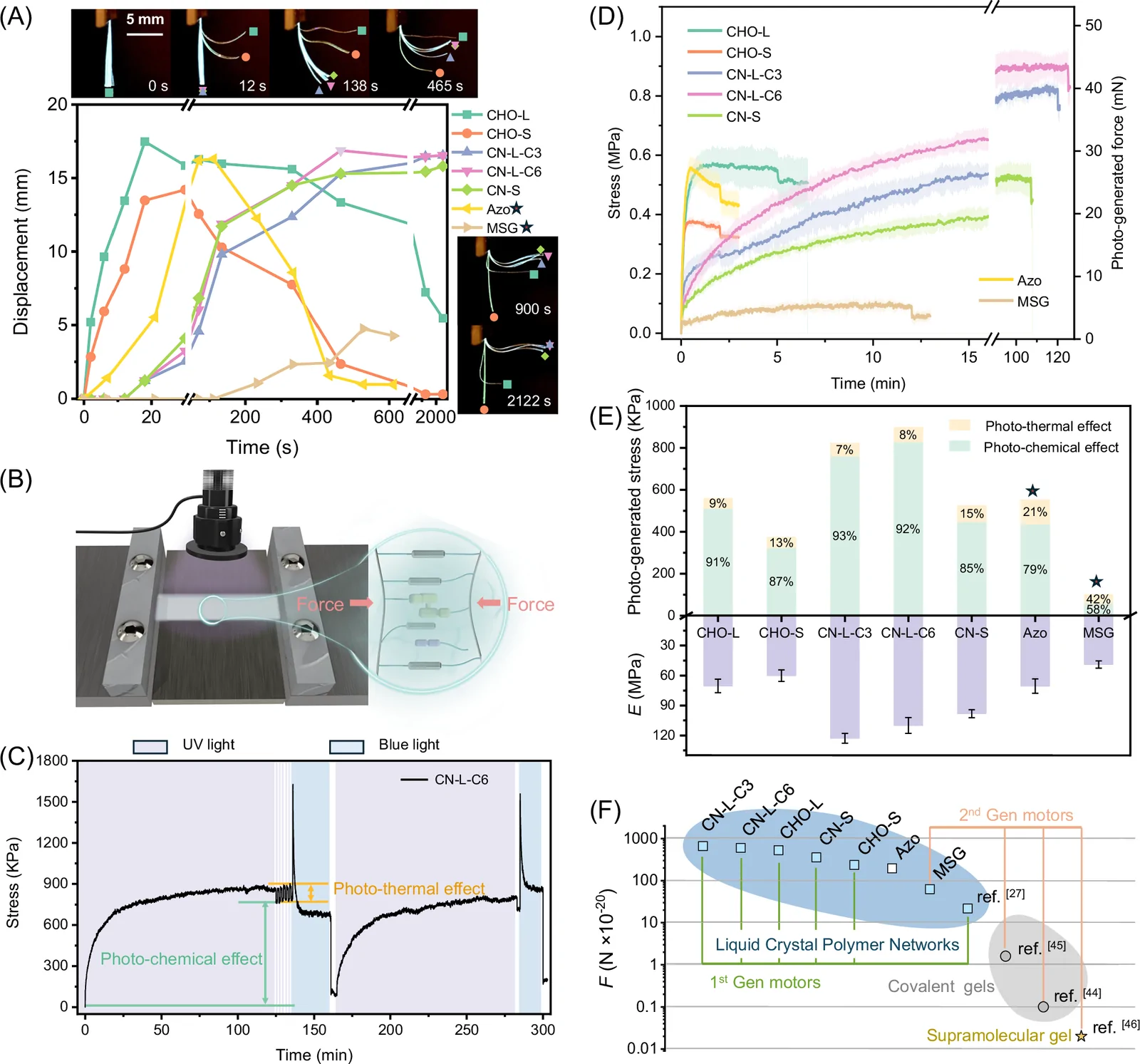
Comparative study on the photo-actuation performance and opto-mechanical properties of LCPNs motorized with different photo-responsive units. **A** Tip displacement curves of different LCPN ribbons upon UV light irradiation. The actuation deformation of LCPN systems based on cyano motors and aldehyde motors were conducted under 340 nm UV irradiation, while those based on **Azo** and **MSG** motors were carried out under 365 nm UV irradiation, both under equivalent power conditions. The snapshots illustrate the actuation process, with the corresponding sample names indicated using scatters matching the displacement curves. The intensity of UV light was 2.2 mW/cm². **B** Schematic diagram of the experimental setup for measuring opto-mechanical properties. The LCPN ribbon was pre-strain (approx. 1%) and the mechanical force was monitored while exposing to light. **C** Photo-generated mechanical stress in LCPN ribbons containing **CN-L-C6** under irradiation at different wavelengths. UV and blue light exposure induced gradual stress accumulation and subsequent release in the material, attributed to the photochemical effect due to rotation of the molecular motor. The instantaneous rise or fall in stress is attributed to the photothermal effect. **D** Building up mechanical stress upon UV irradiation for LCPNs containing different photo-responsive units. The shaded areas represent error ranges with three or more control tests under the same conditions. **E** Comparative analysis of the mechanical performance of LCPNs with different photo-responsive units. The upper part of the figure shows the maximum generated mechanical stress and the relative contributions of photochemical and photothermal effects. The lower part presents the elastic modulus of each network, with error bars representing standard error based on at least three independent measurements. Stress values for cyano motor- and aldehyde motor-based systems were measured under 340 nm irradiation, while azobenzene and the second-generation molecular motor were tested at 365 nm under equivalent power conditions. The intensities of UV and blue light were 13.4 mW/cm² and 188.5 mW/cm², respectively. All LCPN films were planar-aligned and cut along the direction of alignment. Mechanical testing was performed in the same direction as the alignment. **F** Comparative analysis of approximate estimate of forces generated by different light responsive active units in different materials under UV irradiation. The value of force was calculated per single molecular unit (see section 14 of SI).

In order to clarify the effect of the differences in molecular structure on the actuation performance, we first conducted a comparative study on the light-induced deformation behavior of different LCPNs under the same conditions. By utilizing a standardized LCPN matrix and alignment procedure, we minimized extrinsic network variables, allowing for a direct comparison of the intrinsic transduction efficiency of the structurally modified molecular motors. Although we acknowledge that variations in the polymer matrix or motor loading would undoubtedly influence the absolute magnitude of the response, here we used a standardized LCPN scaffold for the direct and systematic study. The LCPNs containing different photo-responsive units were cut into ribbons with a size of 1 mm × 1 cm, and one end of each ribbon was fixed on a glass substrate. The deformation of the LCPNs was observed under 340 nm UV light irradiation (2.2 mW∙cm^-2^). A collimated light beam with a 5 cm diameter ensured consistent irradiation across all ribbons. In the initial state, all the ribbons were upright, and started to bend toward the light source when the light was on. Upon continued exposure, the photoisomerization of the responsive units progressed throughout the film thickness until reaching the PSS state, during which the tip displacement of the ribbons began to decrease gradually. Figure 3A (Supplementary Movie 1, Supplementary Movie 2) shows the snapshots of the deformation of different LCPN ribbons and tip displacement curves under irradiation. The experimental results show that the LCPNs incorporating aldehyde-functionalized motors (**CHO-L** and **CHO-S**) exhibited the fastest response rates under 340 nm light irradiation, which correlates with their higher QY. Notably, **CHO-L** showed a maximum bending comparable to that of **CN-L-C3** and **CN-L-C6**, and significantly greater than for **CHO-S** and **CN-S**. This enhanced performance can be attributed to the rigid extension of the motor core, which induces more pronounced geometric changes during (difference in molecular length upon isomerization is 4.4Å versus 11.4Å for short and long motor cores, respectively, as evidenced by DFT calculations, Supplementary Fig. 43) isomerization, thereby effectively amplifying the molecular-scale isomerization behavior and increasing the macroscopic deformation of the system. It is important to emphasize that the photochemically driven actuation of motorized LCPNs is fully reversible, as demonstrated by cyclic irradiation with UV and blue light (Supplementary Fig. 41). Moreover, this behavior does not arise from large-scale network reorganization, as evidenced by the unchanged liquid crystalline order (see polarized UV-vis spectroscopy data in Supplementary Fig. 44) and actuation can even occur at low temperatures under water (Supplementary Fig. 42).

Moreover, conventional azobenzene (**Azo**) and a second-generation motor (**MSG**) were introduced into similar LCPNs and used for comparison. To provide a fair assessment of the intrinsic mechanical response these photoresponsive units, comparisons were conducted by using 365 nm UV light (which is close to their absorption maxima) of the same intensity 2.2 mW∙cm^-2^ (Fig. 3A, and Supplementary Fig. 40 for experimental details). The results show that the **Azo** system performs similarly to aldehyde-based LCPN but notably slower. All first-generation motors and **Azo** switch demonstrate similar values of maximal achievable curvature, however cyano systems reach these values considerably later likely due to the lower efficiency of photoisomerization. In contrast, **MSG** exhibited the weakest overall actuation performance, probably due to lower QY and poorer PSS, which limited effective force transduction and deformation output.

These results clearly demonstrate that the molecular structure of the photo-responsive unit has a significant effect on the actuation behavior of the studied LCPNs. Although the findings provide structural guidance for the optimization of light-active networks' performance, it is important to note that such performance may be significantly influenced by the mechanical characteristics of the networks and liquid crystalline structures.

### Opto-mechanical properties of motorized networks

Given our claim that molecular motors can efficiently convert light energy into mechanical energy under UV irradiation, we systematically investigated the mechanical properties of LCPN films incorporating different photo-responsive units, including elastic modulus and photo-generated stress (or force) measurements. Differential scanning calorimetry showed that all tested LCPNs are in the rubbery state, with glass transition temperatures ranging from 8.9 to 14.8 °C - well below room temperature (Supplementary Fig. 39). The elastic moduli, determined from the stress-strain curves, range from approximately 60 to 120 MPa, with higher values observed for networks incorporating cyano motors (Fig. 3E, lower panel).

To quantify light-generated stress, flat LCPN ribbons (with molecular alignment along the long axis of the ribbon) were clamped between a load cell and a motorized stage, with a pre-strain of ~1% applied. Such pre-strain ensures that the sample is tensioned and that the load cell operates within a regime sensitive enough to detect small force fluctuations, keeping the network in a linear elastic regime. Before measurements, the stress was allowed to stabilize, ensuring minimal residual stress relaxation. Upon exposure to light, photoisomerization of the embedded motors triggered contraction along the alignment direction, generating measurable mechanical stress. Keeping the strain constant, the load cell recorded the change in mechanical stress generated in the material during light exposure in real time as schematically depicted in Fig. 3B, C shows the stress response behavior of **CN-L-C6** samples under different light conditions. Upon UV irradiation, the stress gradually increases predominantly due to isomerization of molecular motors (photochemical effect) and reaches a saturation value after about 80 min. The UV light intensity of 13.4 mW/cm^2^ was specifically chosen as a balance between activating a maximal fraction of motors and minimizing thermal contributions (typically below 15%), allowing the onset of actuation to be clearly resolved in the stress curves. Additional IR imaging experiments demonstrated that the temperature increase in this case ranges from 0.5 to 1.7 ^o^C indicating a very limited temperature contribution (Supplementary Fig. 46). After turning off the UV light, a rapid and minor reduction of stress was observed which was associated with the photothermal effect contribution upon UV light irradiation. However, the main part of the generated stress remained unchanged upon switching off the light which combined with the cold-water control experiments (Supplementary Fig. 42), indicates that the macroscopic response is driven predominantly by the photochemical isomerization of the molecular motors, while photothermal contributions remain secondary at the intensities used. Important to note that to isolate the photochemical contribution to the generated stress, we calculated the difference between the total recorded stress and the photothermal contribution (Fig. 3C and E). When exposed to blue light of high-intensity which is necessary to accelerate back *Z* to *E* photoisomerization, the stress jumps up rapidly due to the photothermal effect (temperature increase was found as 25 ^o^C, Supplementary Fig. 46) followed by gradual decreases to reach equilibrium after 10 min. After turning off the blue light, the stress returns to the initial state, which indicates that the motor **CN-L-C6** has recovered from the *Z*_M_ state to the *E*_S_ state, and the stress generated by UV irradiation has been erased by the blue light. There was no significant difference in the stress profile upon repeating the exposure cycles, indicating the fatigue resistance of the system.

To ensure the data was both representative and reliable, all measurements were repeated at least three times across all systems, including control compounds. Figure 3D presents the evolution of mechanical stress for each sample during UV exposure, while Fig. 3E (upper part) summarizes the maximum photogenerated stress and the respective contributions of photochemical and photothermal effects. The results reveal that the motor **CN-L** is able to generate significantly larger stress than motor **CN-S**, and similarly, **CHO-L** exhibits superior performance compared to **CHO-S**, suggesting that the rigid extension of the motor core structure effectively enhances photomechanical responses, which is attributed to the greater molecular geometrical change associated with photoisomerization. Importantly, by selecting the proper light intensity we managed to limit the photothermal contribution below 10%, maximizing stress values caused by motors rotation only. Additionally, aldehyde motor-based LCPNs and **Azo** controls displayed faster stress responses upon 365 nm irradiation, which is in line with the previously mentioned photo-deformation of networks. However, **Azo** samples showed an obvious stress relaxation after reaching the maximum, indicating a thermal relaxation of *Z*-**Azo**, accelerated by more pronounced photothermal effect (approx. 21%). Interestingly, **CN-L-C6** generated slightly higher stress than **CN-L-C3**, suggesting that the extension of the flexible spacers between motor core and main polymer chain allows more freedom for the motor rotation. It is worth noting that the photogenerated force of the LCPNs constructed based on the cyano motor is significantly higher than that of the aldehyde motor systems under similar conditions, which is mainly attributed to the introduction of the cyano as a strong electron-withdrawing group, whose larger (and more directional) dipole moment substantially strengthens the coupling between the motors and other components within the LCPN, allowing for better alignment inside the network. The direction and the quality of molecular alignment of **CN-L-C6** and **CHO-L** motors were investigated by using polarized light UV-vis spectroscopy supported by TD-DFT calculations (see details in SI, sections 11 and 12). From these measurements (Supplementary Fig. 45), we found that the CN-based motor exhibits significantly higher orientational order compared to the CHO analogue (linear dichroism *D* ≈ 0.37 vs. 0.22). Given that the photogenerated force arises from directional molecular contraction along the motor axis, the degree of alignment directly determines how effectively individual molecular motions are translated into macroscopic stress. In this context, the higher degree of alignment of the CN motors leads to a more cooperative and directional force generation within the network, which explains the larger measured photogenerated forces despite their lower quantum yield (accounted for by the slower buildup of these forces). The better alignment of the motors (which play the role of crosslinkers) leads to increased network rigidity. The significantly higher modulus of the cyano-based LCPNs compared to the aldehyde-based system further supports this point (Fig. 3E, lower part). As a control, the **MSG** sample exhibits the weakest photo-generated stress (upon 365 nm exposure) and elastic modulus, with the photothermal effect accounting for up to 42%, indicating that the photo-mechanical efficiency of this system is significantly limited, which is also confirmed by hampered deformation of the corresponding ribbon (Fig. 3A).

Additionally, we calculated approximate estimates the total force generated by the motor cores as well as contributed by each molecule within the LCPN networks under UV irradiation (Table 2, detailed calculation processes assumptions made are provided in the section 14 of SI). The data shows that the LCPN system based on the cyano motor exhibits significantly higher photogenerated force compared to the aldehyde motor. Meanwhile, the extended rigidity of the motor core also effectively increased the output force. Compared to our previously reported system based on unsubstituted first-generation motors^27^, the approximate estimate of generated force is enhanced by more than one order of magnitude. These results further validate the effectiveness of incorporating electron-withdrawing groups to increase the network modulus and thereby amplify the photo-generated force, offering a robust structure-property design strategy. Moreover, under identical conditions, both cyano- and aldehyde-based networks exhibit significantly greater photo-generated forces than conventional photo-responsive motifs such as **Azo** and **MSG**, indicating superior efficiency in converting light energy into mechanical work (Fig. 3F).

**Table 2:** Estimated force contribution by the gram of photo-responsive molecular core (*F*_core_) and the contribution of each individual active unit (*F*_unit_)

Material	*F*_core_ (N/g)	*F*_unit_ (N ×10^-20^)
CN-L-C3	6520	659
CN-L-C6	5940	600
CHO-L	5190	529
CN-S	5800	355
CHO-S	3770	235
Azo	5520	195
MSG	1070	62.4
1^st^-gen motor in LCPN^27^	380	21.8
2^nd^ gen motor in Gel^46^	28.3	1.6
2^nd^ gen motor in Hydrogel^45^	2.1	0.1
Muscle fibers (supramolecular polymer)^47^	0.3	0.02

Furthermore, we compared the estimated force contributions of molecular motors of these LCPNs with previously reported materials in which molecular motors were embedded, including gels^45,46^, and supramolecular muscles-like fibers^47^. As shown in Table 2 and Fig. 3F, the approximate estimate of photo-generated force of our system considerably exceeds that of other soft materials. These results strongly suggest that the LCPN systems constructed based on molecular motors reported in this study are capable of generating stronger mechanical outputs and enabling more effective mechanical actuation. Therefore, the molecular structure, especially the electronic effects of substituents, the degree of rigid extension of the motor core, and the length of the flexible spacers, have a significant influence on overall mechanical performance of LCPN materials.

### Coupled photoactuation-fluorescence behavior

**Fig. 4:**
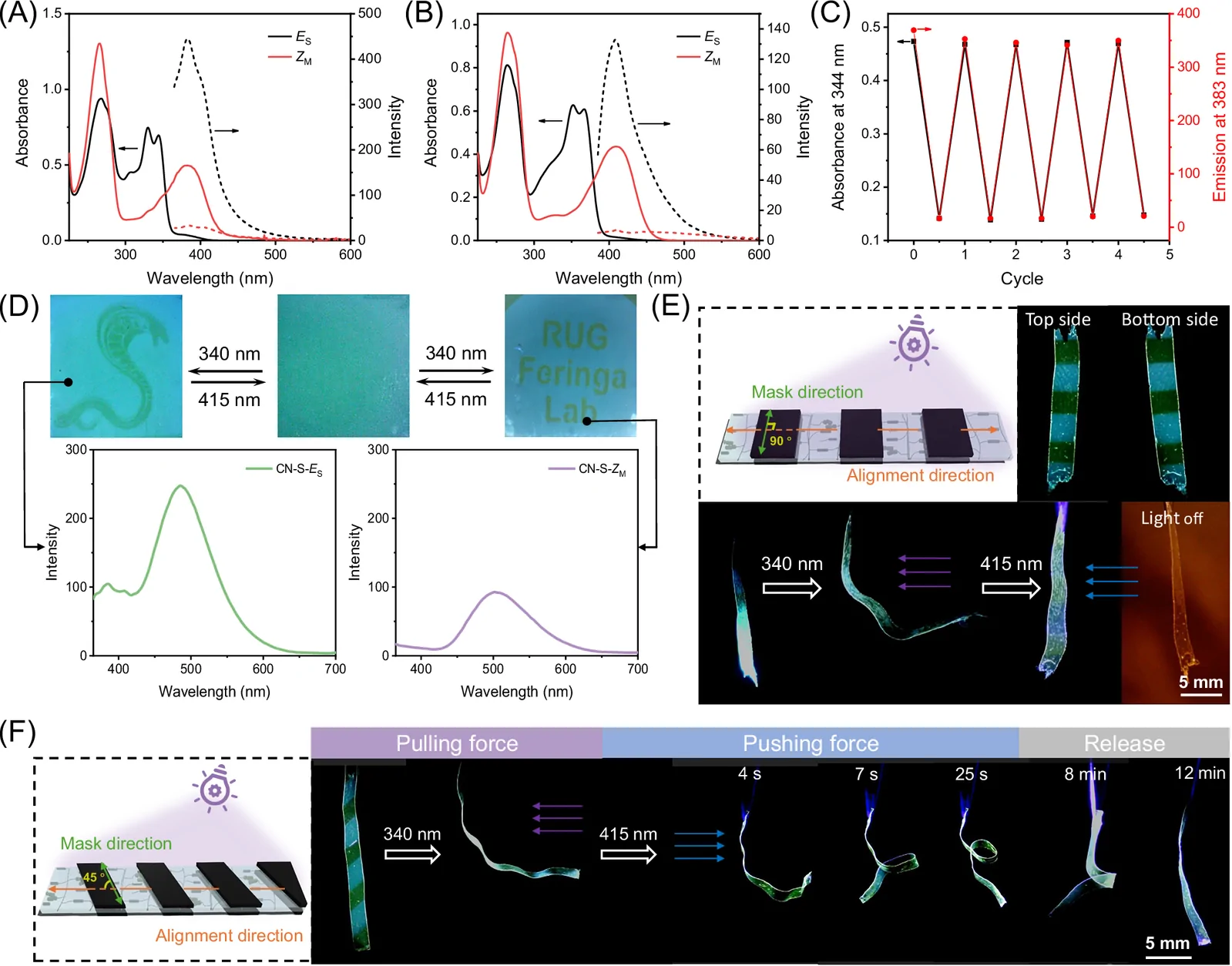
Coupling of shape-shifting behavior and switchable fluorescence of cyano motors. Absorbance and emission spectra of **CN-L-C6** (**A**) and **CHO-L** (**B**) in *E*_S_ and *Z*_M_ states measured in DCM solution. **C** Fatigue study of **CN-L-C6** upon cyclic irradiation with 340 nm (to produce *Z*_M_) and 415 nm light (to produce *E*_S_). **D** Reversible visualization of predefined patterns on **CN-S** LCPN films using mask exposure. Under 340 nm irradiation, the LCPN film displays patterns such as a snake and the text “RUG Feringa Lab”, which can be erased under 415 nm light. The blue regions indicate the initial state, while the green regions correspond to UV-exposed areas, as indicated in the fluorescence spectra below. **E** Shape encoding of LCPN ribbon using a mask. The stripes of the mask were oriented perpendicular to the LC alignment. The ribbon was exposed from both sides. The corresponding striped fluorescence pattern is visible under UV excitation. Upon alternating exposure to 340 nm and 415 nm light from the same side, the ribbon exhibited reversible bending deformation. **F** LCPN ribbon was shape-encoded by a mask. The stripes of the mask were oriented at 45° relative to the LC alignment. The ribbon was exposed from both sides. Under 340 nm irradiation, the ribbon undergoes a bend toward the light source. When 415 nm light was applied to the opposite side, the ribbon bends away from the light, yielding a left-handed spiral shape. Prolonged exposure to 415 nm light resulted in the gradual unwinding of the spiral to the initial straight shape.

During the investigation of the light-driven deformation behavior of LCPN films, we observed that samples containing cyano and aldehyde motors exhibited noticeable fluorescence under UV illumination. This phenomenon prompted a further examination of their fluorescence response upon UV light excitation. To elucidate the origin of fluorescence, we first performed studies of the molecular motors in solution. As shown in Fig. 4A, B, both **CN-L-C6** and **CHO-L** motors exhibit pronounced fluorescence in their *E*_S_ states, with emission maxima centered at 383 nm and 410 nm, respectively. In contrast, the corresponding *Z*_M_ states are nearly non-emissive. The emission profiles closely follow the absorption spectra, confirming that fluorescence originates from the intrinsic electronic structure of the motors. Quantitatively, **CN-L-C6** motor exhibits approx. 4-fold higher emission intensity compared to the **CHO-L** (Supplementary Fig. 47a). This difference correlates with its lower photoisomerization efficiency, indicating competition between radiative decay and photochemical isomerization pathways in the excited state. The aldehyde group promotes efficient photochemical isomerization via additional non-radiative channels^35^, whereas the cyano group favors emissive decay (likely due to π-accepting character stabilizing planar charge transfer state) and limits access to reactive states. The reversibility and fatigue resistance of the fluorescence response in solution was studied under repeated 340 nm/415 nm irradiation cycles (Fig. 4C) and demonstrates good photochemical robustness.

Compared to solution, the emission spectra of LCPNs exhibit notable differences, particularly the appearance of a long-wavelength emission band around 480 nm and a relative suppression of the ~380 nm peak (Supplementary Fig. 48a). To clarify this effect, we prepared thin film (~2 μm), in which the short-wavelength emission becomes dominant like in solution (Supplementary Fig. 48b). This observation indicates that the apparent spectral shift in thicker films originates primarily from reabsorption (inner filter effects), as supported by the corresponding transmission spectra (Supplementary Fig. S48). While the precise photophysical origin of the red-shifted emission band in these LCPN films remains to be fully elucidated, it likely arises from network-induced conformational constraints of the molecular motors or specific electronic interactions with the surrounding polymer environment. More information on fluorescent properties can be found in Supporting Information (section 15).

To demonstrate the potential of actuating and fluorescent read-out combined in a single molecular scaffold, we prepared a 25 μm-thick **CN-S** layer. The short-wavelength emission peak of **CN-S** was diminished while long-wavelength peak was red-shifted from 480 nm to 500 nm and reduced in intensity upon prolong irradiation with 340 nm UV light (Fig. 4D). Given its fluorescence response, we explored the use of this feature for encoding of optical patterns. After UV exposure of the LCPN film through the mask (containing either a snake pattern or text), one can clearly see the emission patterns which could be effectively erased under 415 nm light irradiation, thus realizing the reversible optical information recording (Fig. 4D).

Further, combining its fluorescent properties with mechanical properties, we designed shape-coded pattern visualization experiments to demonstrate the multiple response advantages of this system. When a striped photomask was applied perpendicularly to the LC alignment direction and irradiated with 340 nm UV light, a clear pattern emerged. Upon peeling the film from the substrate, the patterned LCPN ribbon exhibited notable bending deformation (Supplementary Fig. 51a). When the mask stripes were applied at a 45° angle relative to the LC alignment, the ribbon folded into a right-handed (RH) spiral (Supplementary Fig. 51b). These observations indicate that even if photoisomerization occurs in only a part of the film, molecular motors generate enough mechanical stress to drive the macroscopic deformation demonstrating the high mechano-actuating ability of the motor.

We also studied the response behavior of films exposed through the mask from both sides. When the mask stripes were perpendicular to the LC alignment and applied to both sides of the ribbon, subsequent 340 nm UV irradiation led to symmetric motor activation in the exposed regions (Fig. 4E, left top panel). When the ribbon was removed from the substrate, it remained straight due to balanced internal stresses, and the fluorescent pattern was clearly visible (Fig. 4E, right top panel). If the ribbon was then irradiated unilaterally with 340nm light, the motors in non-exposed areas of the ribbon continued isomerizing, which disrupted the stress balance and induced bending motion toward the light source. This deformation could be reversed by illuminating the same side with 415 nm light, which triggered the motors to revert from the *Z*_M_ to the *E*_S_ state, thereby releasing the accumulated stress accompanied by erasure of fluorescent pattern (Fig. 4E, bottom panel, Supplementary Movie 3).

When the ribbon was masked on both sides at an angle of 45° relative to the LC alignment (Fig. 4F, left panel) and irradiated with 340 nm UV light, it remained straight. Upon further unilateral and homogeneous (without the masks) irradiation with UV light, the ribbon deformed toward the light source caused by motors pulling the network in non-exposed areas of the ribbon (appeared as blue). Subsequently, when the other side of the film was exposed to 415 nm blue light, the ribbon first bent away from the light source caused by the motor pushing the network in exposed areas (appeared as green) of the ribbon due to back *Z*_M_ to *E*_S_ photoisomerization taking place while non-irradiated areas are “silent” since they *E*_S_ state does not absorb blue light. This process yielded the subsequent folding of the ribbon into a chiral shape (left-handed spring). Further irradiation with 415 nm light the spiral shape was gradually unfolded and the ribbon returned to the initial straight shape. This is attributed to the increasing the light penetration by increasing the exposure time which erases the gradients of *Z*_M_ and *E*_S_ states across the film thickness accounted for the deformation (Fig. 4F, Supplementary Movie 4).

These results demonstrate that the LCPN films exhibit pronounced switchable fluorescence, enabling precise photopatterning and shape encoding. The coupling of fluorescence read-out with the reversible mechanical deformation behavior not only confers multiple response properties to the material but also provides design strategies and application potentials for the realization of light-controlled visualization, information encryption, and the construction of smart soft devices.

In summary, we successfully designed and synthesized first-generation cyano-motors and aldehyde-motors, and further constructed a liquid-crystalline polymer networks based on these motors capable of versatile deformation. By systematically modulating the chemical structure of the motorized crosslinkers, such as substituent groups, extension of the motor core structure and flexible chain length, the structure-property relationships governing the macroscopic behavior of the materials were analyzed and compared with the second-generation molecular motors and commonly used azobenzene photoswitches as well as with so far reported motorized soft materials. The comparative studies of photo-actuated deformation behavior and mechanical performance of LCPNs revealed a significant influence of molecular structure on actuation behavior, photo-generated stress, and elastic modulus. Notably, LCPNs based on cyano-motors exhibited superior photo-mechanical properties, while those based on aldehyde-motors showed faster response. Additionally, modulation of the flexible chain length effectively tuned the rotational freedom of the motors, further impacting actuation efficiency and rigidity of the materials. Approximate estimations confirmed that the photo-generated force from the single motor unit in our systems far exceeds that of conventional photo-responsive molecules such as azobenzene and the second-generation molecular motors, verifying their potential in the development of highly efficient light-driven soft actuators. More importantly, these systems exhibited remarkable and switchable fluorescence fingerprints. For the first time, we systematically observed and investigated the coupling between photoactuation and fluorescence response in motor-driven LCPNs. Compared to traditional composite systems, our functional molecular design realized synergistic integration of efficient actuation and visual encoding. Through mask patterning and photo-controlled shape encoding, the high level of controllability and multifunctionality of these systems was demonstrated. The developed motorized LCPN systems' broad application prospects in light-controlled actuators and patterned functional materials are paving the way for the engineering of molecularly driven adaptive materials.

## Methods

### Synthesis

The synthetic routes of motors **CN-S,** **CN-L-C3,** **CN-L-C6**, and **CHO-S**, as well as the structural characterization of all intermediates involved (¹H-NMR, ¹³C-NMR, and HRMS), are provided in Sections 2 and 3 of the Supplementary Information. The synthesis of **CHO-L** was carried out following a previously developed procedure^43^. **Azo** was purchased from Synthon Chemicals and used without further purification. The synthesis of **MSG** was carried out following the procedure reported in the previous work^25^. The ¹H-NMR data of CHO^35^, C3OTBS^43^, C6OTBS^48^, CHO-L^43^, and MSG^25^ were consistent with those reported in the literature.

### Spectroscopic measurements

NMR spectra of the rotational cycles of the motor in solution were collected on a Varian Unity Plus spectrometer (¹H: 500 MHz, ¹³C: 126 MHz). UV-vis absorption spectra of the sample in solution were measured on a Hewlett-Packard 8454 diode array spectrometer in a 1 cm quartz cuvette. In situ irradiation experiments were performed using fiber-coupled Thorlab LEDs (M340L5, M365FP1, M415L4, M455F3). For the fluorescence properties study of motorized LCPNs, the spectrofluorometer JASCO FP6200 equipped with EFA-382 epifluorescence accessory was used. Light intensities were measured by a powermeter PM400 equipped with a thermal power sensor S401C (Thorlabs).

### Samples preparation

LCPN films were prepared by using home-made 25 μm-thick glass LC-cells with polyimide aligning layers. The approach is based on well-established protocols of LCPNs fabrication^21,23–25,27,41^ and details are provided in Section 6 of the SI. LCPNs were produced by blue light (λ = 470 nm) photopolymerization of monomeric mixtures (compositions are shown in Supplementary Table 3) introduced into LC cell, followed by thermal annealing. Detailed protocol for LCPNs fabrication is provided in Section 7 of the Supplementary Information.

### Mechanical measurements

Elastic modulus of the LCPNs was calculated from the recorded stress-strain curves using a Modular Force Stage (MFS, Linkam). A constant extension rate of 50 μm/s was used. All polymer ribbons (thickness of 25 μm) were cut along the LC alignment direction to ensure stretching was performed parallel to the molecular orientation. To study photo-generated forces, the ribbons were subjected to a pre-strain (1 %) and were irradiated with light of different wavelengths (340 nm, 365 nm, and 415 nm) using mounted LEDs (Thorlabs). The photo-generated force of the films was monitored in real time during irradiation.

### Shape encoding of LCPN films

The prepared LCPN films were cut along the alignment direction into ribbons (2 mm × 20 mm) and placed flat on glass slides. The films were then covered with photomasks of different patterns, followed by UV irradiation (340 nm). The distance between the light source and the sample was fixed at 15 cm, with a beam diameter of 2 cm and an intensity of 13.4 mW/cm². After irradiation, the films were peeled off the slides to obtain shape-encoded LCPN films.

## Supplementary information


Supplementary Information
Description of Additional Supplementary Files
Supplementary Movie 1
Supplementary Movie 2
Supplementary Movie 3
Supplementary Movie 4
Peer Review File


## Source data


Source data


## Data Availability

Data that support the findings of this study are available within the main text or supplementary information. All data are available from the corresponding author upon request. All UV-vis spectra, fluorescence spectra, and mechanical performance data generated in this study are available as source data. Source data are provided with this paper.
