# Supplementary material for: Efficient molecular motors in liquid crystal networks enable the integration of fluorescence with large opto-mechanical effects
Source: Nat Commun. 2026 Jul 30;17:9241. doi: 10.1038/s41467-026-76177-0 (PMC13527073; doi:10.1038/s41467-026-76177-0)
Supplement: Supplementary file 1 [file 41467_2026_76177_MOESM1_ESM.pdf]

Supplementary Information

Efficient Molecular Motors in Liquid Crystal Networks Enable the Integration of Fluorescence with Large Opto-mechanical Effects

Guiying Long [1, 2], Jiahui Meng [2], Alexander Ryabchun*[1], Ben L. Feringa*[1, 2]

1 Stratingh Institute for Chemistry, University of Groningen, Nijenborgh 3, 9747 AG Groningen, The Netherlands.

[2] SCNU-UG International Joint Laboratory of Molecular Science and Displays, National Center for International Research on Green Optoelectronics, South China Normal University, Guangzhou 510006, China.

*Corresponding authors: Dr. A. Ryabchun (a.ryabchun@rug.nl), Prof. B. L. Feringa (b.l.feringa@rug.nl)

Table of Contents

Supplementary Methods **1**

1 General information 1

2 Synthetic routes for the cyano motors, aldehyde motors, and MSG 2

3 Characterization of compounds 9

Supplementary Discussion **25**

4 ^{1}H-NMR studies of motors CN-L-C3, CN-L-C6 and CHO-S in solution 25

5 UV-vis studies of motor in solution 27

5.1 Photochemical isomerization processes 27

5.2 Thermal Helical Inversion process 27

5.3 Photoisomerization quantum yield 29

6 Preparation of LC cells 32

7 Preparation LC mixtures and LCPNs 32

8 DSC characterization of LCPNs 33

9 Comparative study on the photo-actuation performance of LCPNs driven by different photo-responsive units 33

10 Fatigue study of bending motion and bending under water 34

11 DFT calculations 35

12 Molecular alignment studies 36

13 Thermal imaging study 39

14 Mechanical properties 39

15 Fluorescence properties of cyano and aldehyde motros 43

16 Shape encoding of LCPN ribbons 46

17 Optimized geometries of cyano and aldehyde motors 46

Supplementary Reference **55**

Supplementary Methods

1 General information

All reagents from commercial sources such as Aldrich, TCI, or Merck were used as received. Anhydrous DCM, THF, toluene was obtained from a solvent purification system (MBRAUN SPS systems, MBSPS-800). **Azo** was purchased from Synthon Chemicals and used without further purification. Flash column chromatography was performed using silica gel (SiO2) purchased from Merck (type 9385, 230-400 mesh) or on a Büchi Reveleris purification system with Büchi cartridges. High resolution mass spectrometry (ESI or APCI-MS) was performed on a LTQ Orbitrap XL spectrometer with ESI or APCI ionization. NMR spectra were recorded on Varian AMX400 (^1H: 400 MHz, ^{13}C: 101 MHz), Varian Unity Plus (^1H: 500 MHz, ^{13}C: 126 MHz) and Bruker Innova600 (^1H: 600 MHz, ^{13}C: 151 MHz) spectrometers. Chemical shifts are quoted in parts per million (ppm) relative to the residual solvent signal (for $CDCl_3$ δ 7.26 for ^1H, δ 77.16 for ^{13}C and for CD_2Cl_2 δ 5.32 for ^1H, δ 53.84 for ^{13}C). For ^1H-NMR spectroscopy, the splitting pattern of peaks is designated as follows: s (singlet), d (doublet), t (triplet), m (multiplet), br (broad), or dd (doublet of doublets). UV-vis absorption spectra were measured on a Hewlett-Packard 8454 diode array spectrometer in a 1 cm quartz cuvette. All the irradiation experiments in solutions were performed using Thorlab LEDs (M340L5, M365FP1, M415L4, M455F3). Solvents used for spectroscopic studies were of spectroscopic grade (UVASOL, Merck) or from a solvent purification system. Polarized light absorbance spectra were recorded by AVASPEC-ULS2048CL-EVO (Avantes) spectrometer equipped with Glan-Taylor polarizer on a rotational mount (ELL14K, Thorlabs). Irradiation of liquid crystal samples were performed using mounted LEDs (Throlab) at 340 nm, 365 nm, 415 nm and 455 nm. Light intensities were measured with light powermeter PM400 (Thorlabs). All mechanical property measurements of the materials were conducted using Modular Force Stage (MFS) of Linkam. The basic MFS system consists of an MFS stage, a Base, a Force Beam, and a T9X System Controller. Differential Scanning Calorimetry (DSC) were carried out on a Netzsch DSC 300 Caliris Select in a dry nitrogen atmosphere at 10 K/min rate. For thermal imaging, IR camera (FLIR, E5) was used.

2 Synthetic routes for the cyano motors, aldehyde motors, and MSG

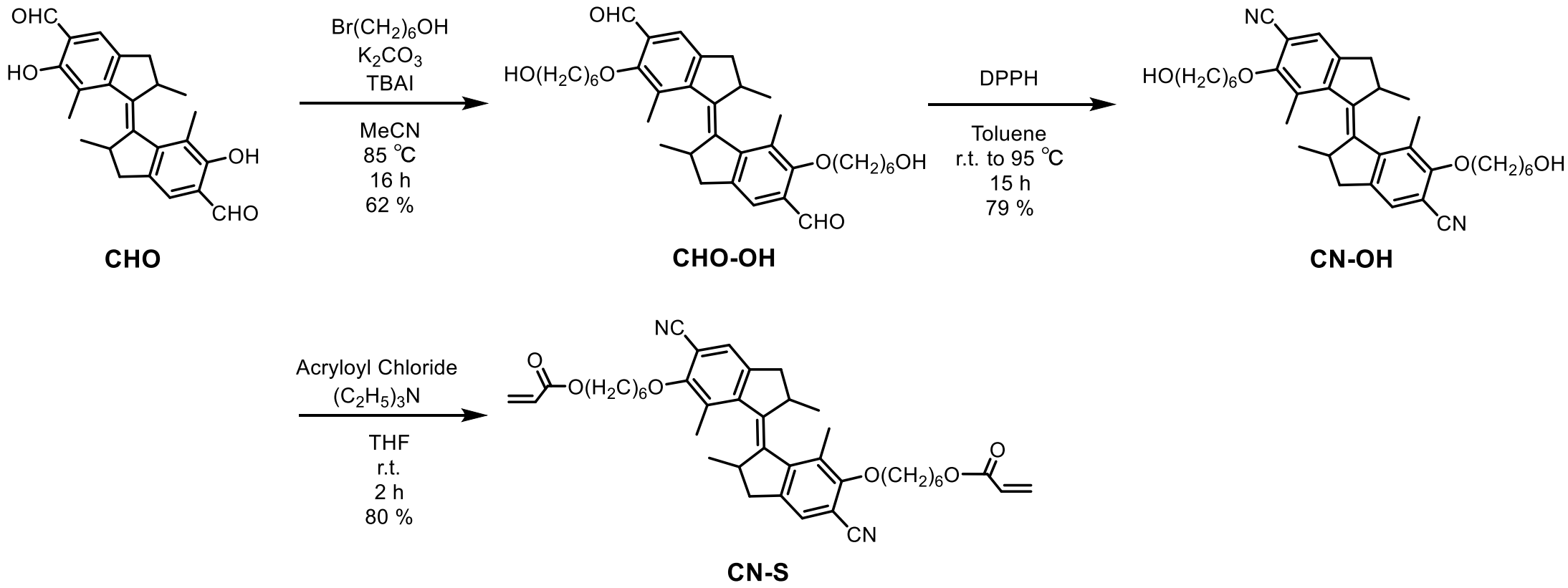

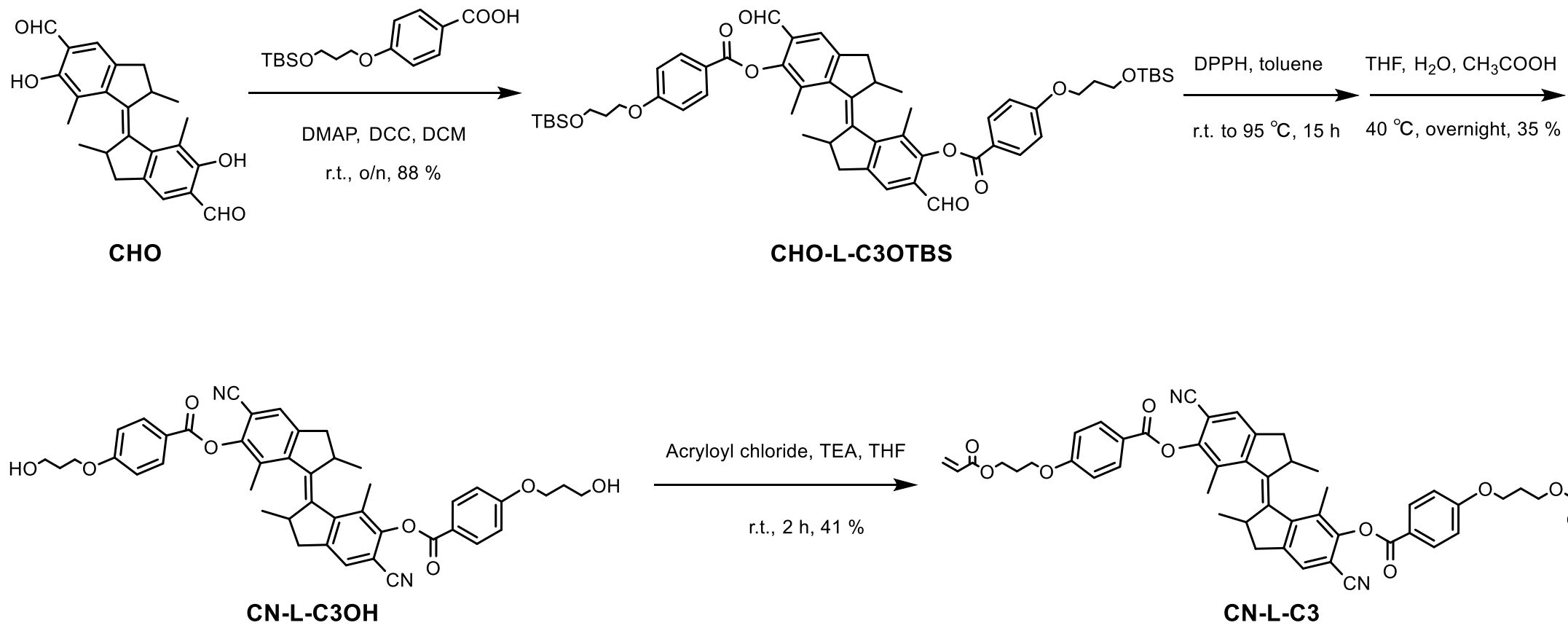

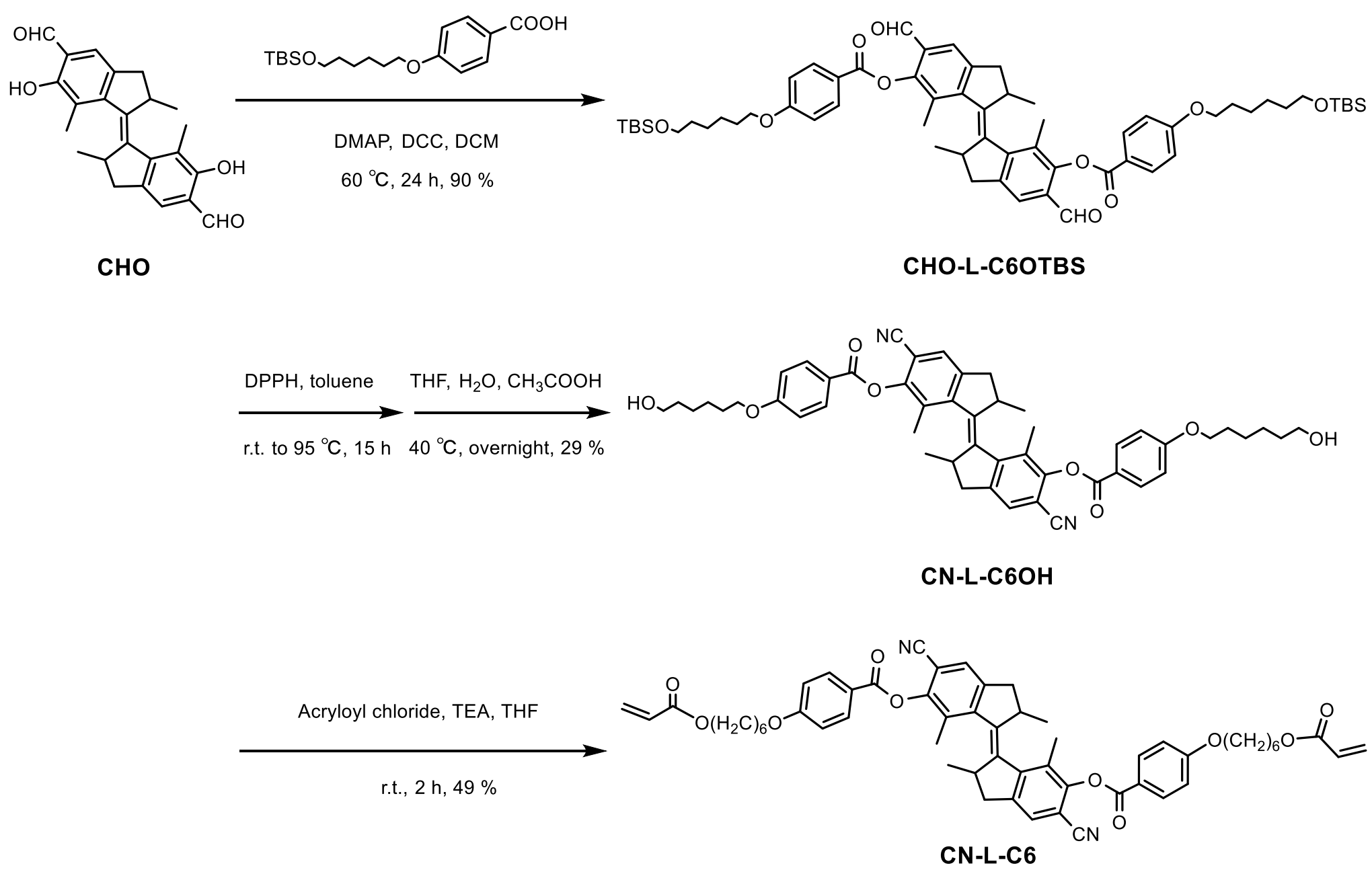

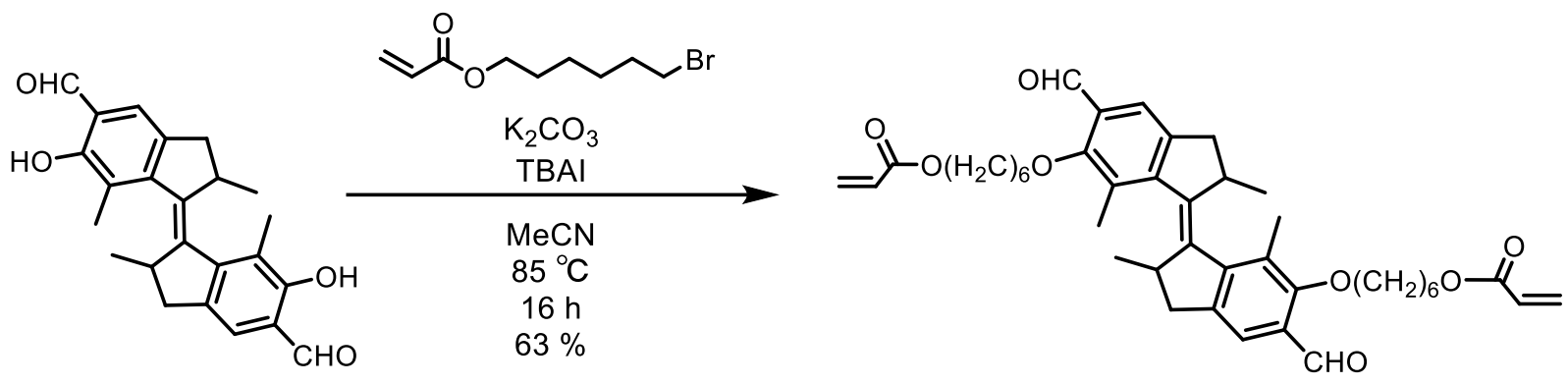

CHO **CHO-S**

Supplementary Fig. 1 | Synthesis of the motors. Synthetic routes for the light-driven rotary molecular motors **CN-S, CN-L-C3, CN-L-C6** and **CHO-S.**

(*E*)-6,6'-dihydroxy-2,2',7,7'-tetramethyl-2,2',3,3'-tetrahydro-[1,1'-biindenylidene]-5,5'-dicarbaldehyde

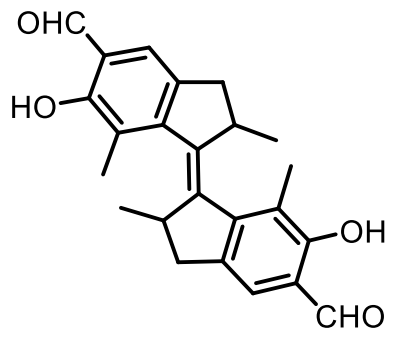

Compound **CHO** was synthesized following the procedure in ref [1]. ^{1}H-NMR (400 MHz, Chloroform-*d*) δ 11.57 (d, *J* = 0.9 Hz, 1H), 9.83 (d, *J* = 1.5 Hz, 1H), 7.24 (s, 1H), 2.93 (p, *J* = 6.4 Hz, 1H), 2.75 (dd, *J* = 14.1, 5.7 Hz, 1H), 2.38 (s, 3H), 2.31 (d, *J* = 14.1 Hz, 1H), 1.13 (d, *J* = 6.5 Hz, 3H).

(*E*)-6,6'-bis((6-hydroxyhexyl)oxy)-2,2',7,7'-tetramethyl-2,2',3,3'-tetrahydro-[1,1'-biindenylidene]-5,5'-dicarbaldehyde (CHO-OH)

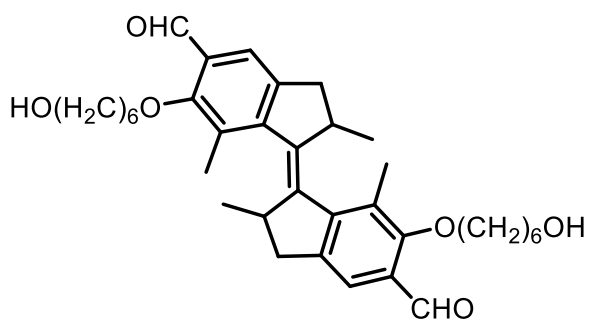

To a solution of **CHO** (40 mg, 0.10 mmol) in MeCN (10 mL), K_2CO_3 (41.5 mg, 0.3 mmol), tetrabutylammonium iodide (110.8 mg, 0.3 mmol) and 6-bromo-1-hexanol (72.4 mg, 0.4 mmol) were added. The mixture was stirred at 85 °C under nitrogen for 16 h. After cooling to room temperature, the mixture was acidified using a saturated aqueous NH_4Cl solution to pH = 6-7. The product was extracted with EtOAc (3 × 20 mL). The combined organic layers were washed with brine (50 mL), then dried over Na_2CO_3. The solvent was removed in vacuo and the residue was purified by column chromatography (SiO_2, pentane: EtOAc= 1: 3) to yield motor **CHO-OH** (36 mg, 0.06 mmol, 62 %) as yellow oil.

^{1}H NMR (600 MHz, Chloroform-*d*) δ 10.39 (s, 1H), 7.59 (s, 1H), 4.11 (dt, *J* = 9.2, 6.5 Hz, 1H), 3.94 (dt, *J* = 7.7, 5.8 Hz, 1H), 3.72 (t, *J* = 6.5 Hz, 2H), 2.94 – 2.88 (m, 1H), 2.83 – 2.77 (m, 1H), 2.44 (s, 3H), 2.35 (d, *J* = 14.5 Hz, 1H), 1.94 (dq, *J* = 8.6, 6.6 Hz, 2H), 1.70 – 1.64 (m, 2H), 1.63 – 1.60 (m, 2H), 1.55 – 1.49 (m, 2H), 1.14 (d, *J* = 6.5 Hz, 3H).

^{13}C NMR (151 MHz, Chloroform-*d*) δ 190.4, 161.1, 149.2, 143.8, 139.8, 128.6, 128.0, 121.9, 76.3, 62.9, 43.2, 34.0, 32.7, 30.2, 25.9, 25.7, 18.5, 16.4.

HRMS (ESI, m/z) calculated for $C_{36}H_{48}O_6Na$ $[M+Na]^+$: 599.3343, found 599.3347.

(*E*)-6,6'-bis((6-hydroxyhexyl)oxy)-2,2',7,7'-tetramethyl-2,2',3,3'-tetrahydro-[1,1'-biindenylidene]-5,5'-dicarbonitrile (CN-OH)

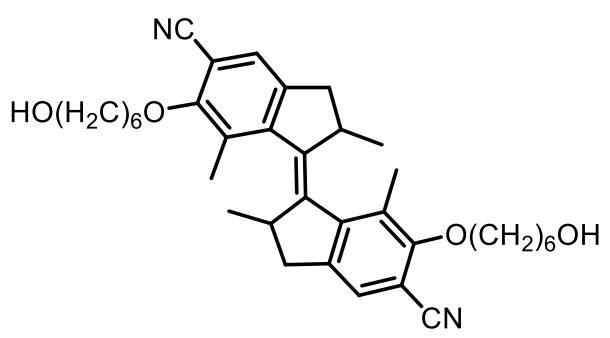

Compound **CHO-OH** (60 mg, 0.1 mmol) and O-(Diphenylphosphoryl)hydroxylamine (DPPH, 53.6 mg, 0.23 mmol) were dissovled in dry-toluene (3 mL). The mixture was stirred at room temperature under nitrogen for 3 h. The reaction was then heated to 95 °C for 12 h. After cooling to room temperature, water (10 mL) was added to quench the reaction and the mixture was extracted with EtOAc (3 × 20 mL). The combined organic layers were washed with brine (50 mL), then dried over Na_2CO_3 and concentrated under

vacuum. The crude product was purified by column chromatography (SiO_2, pentane: EtOAc= 1: 2) to yield motor **CN-OH** (45 mg, 0.08 mmol, 79 %) as yellow oil.

^{1}H NMR (600 MHz, Methylene Chloride-d_2) δ 7.31 (s, 1H), 4.22 (dt, *J* = 9.1, 6.5 Hz, 1H), 4.09 (dt, *J* = 9.1, 6.5 Hz, 1H), 3.67 (q, *J* = 6.1 Hz, 2H), 2.89 (p, *J* = 6.4 Hz, 1H), 2.79 (dd, *J* = 14.5, 5.6, 1H), 2.41 (s, 3H), 2.33 (d, *J* = 14.4 Hz, 1H), 1.98 – 1.90 (m, 2H), 1.64 (dtd, *J* = 13.2, 7.8, 6.6 Hz, 4H), 1.54 – 1.49 (m, 2H), 1.13 (d, *J* = 6.5 Hz, 3H).

^{13}C NMR (151 MHz, Methylene Chloride-d_2) δ 159.8, 147.7, 143.3, 139.6, 128.9, 126.7, 117.5, 104.6, 74.6, 62.6, 43.2, 39.7, 32.8, 30.3, 25.7, 25.5, 18.1, 16.4.

HRMS (ESI, m/z) calculated for $C_{36}H_{46}N_2O_4Na$ $[M+Na]^+$: 593.3356, found 593.3350.

(*E*)-((5,5'-dicyano-2,2',7,7'-tetramethyl-2,2',3,3'-tetrahydro-[1,1'-biindenylidene]-6,6'-diyl)bis(oxy))bis(hexane-6,1-diyl) diacrylate (CN-S)

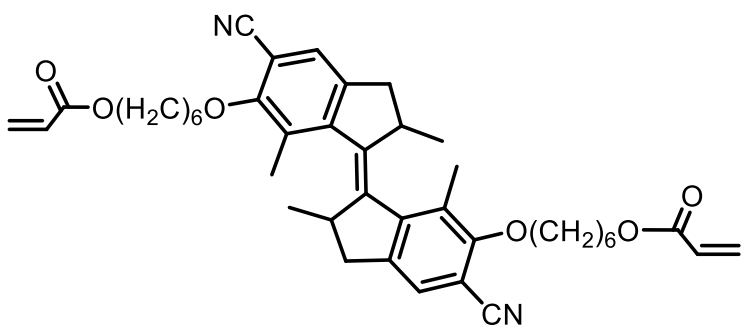

To a solution of **CN-OH** (100 mg, 0.18 mmol) in THF (5 mL), triethylamine (364 mg, 3.6 mmol) and acryloyl chloride (40.7 mg, 0.45 mmol) were added at 0 °C. The mixture was allowed to warm up to room temperature and stirred for 2 h. The mixture was quenched with water (50 mL) and the aqueous phase was extracted with EtOAc (3 × 50 mL). The combined organic layer was washed with saturated NH_4Cl solution (2 × 50 mL) and brine, then dried over Na_2SO_4. The solvent was removed in vacuo and the residue was purified by flash column (SiO2, pentane: EtOAc=6:1) to yield motor **CN-S** as yellow oil (97.7 mg, 0.14 mmol, 80%).

^{1}H-NMR (600 MHz, Methylene Chloride-d_2) δ 7.19 (s, 1H), 6.28 (dd, *J* = 17.3, 1.5 Hz, 1H), 6.04 (dd, *J* = 17.4, 10.4 Hz, 1H), 5.73 (dd, *J* = 10.4, 1.5 Hz, 1H), 4.13 – 4.06 (m, 3H), 3.97 (dt, *J* = 9.1, 6.4 Hz, 1H), 2.79 – 2.74 (m, 1H), 2.67 (dd, *J* = 14.5, 5.3 Hz, 1H), 2.29 (s, 3H), 2.21 (d, *J* = 14.4 Hz, 1H), 1.86 – 1.79 (m, 2H), 1.66 (dq, *J* = 7.9, 6.7 Hz, 2H), 1.52 (dddd, *J* = 14.1, 9.7, 7.2, 3.1 Hz, 2H), 1.42 (tt, *J* = 9.0, 6.4 Hz, 2H), 1.01 (, 3H).

^{13}C NMR (151 MHz, Chloroform-*d*) δ 166.4, 159.8, 147.5, 143.2, 139.3, 130.5, 128.8, 128.6, 126.9, 117.5, 104.5, 74.6, 64.6, 43.2, 39.8, 30.2, 28.6, 25.8, 25.7, 18.4, 16.5.

HRMS (ESI, m/z) calculated for $C_{42}H_{50}N_2O_6Na$ $[M+Na]^+$: 701.3555, found 701.3561.

4-(3-((tert-butyldimethylsilyl)oxy)propoxy)benzoic acid

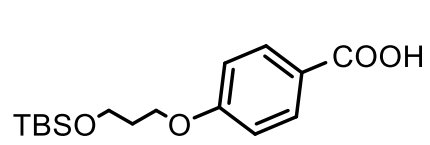

Compound **C3OTBS** was synthesized by following the procedure in ref [2].

^{1}H NMR (401 MHz, DMSO-d_6) δ 7.85 (d, *J* = 8.4 Hz, 1H), 6.97 (d, *J* = 8.5 Hz, 1H), 4.08 (t, *J* = 6.1 Hz, 1H), 3.73 (t, *J* = 6.0 Hz, 1H), 1.89 (h, *J* = 5.9 Hz, 1H), 0.83 (s, 4H), -0.01 (s, 3H).

(*E*)-5,5'-diformyl-2,2',7,7'-tetramethyl-2,2',3,3'-tetrahydro-[1,1'-biindenylidene]-6,6'-diyl bis(4-(3-((tert-butyldimethylsilyl)oxy)propoxy)benzoate) (CHO-L-C3OTBS)

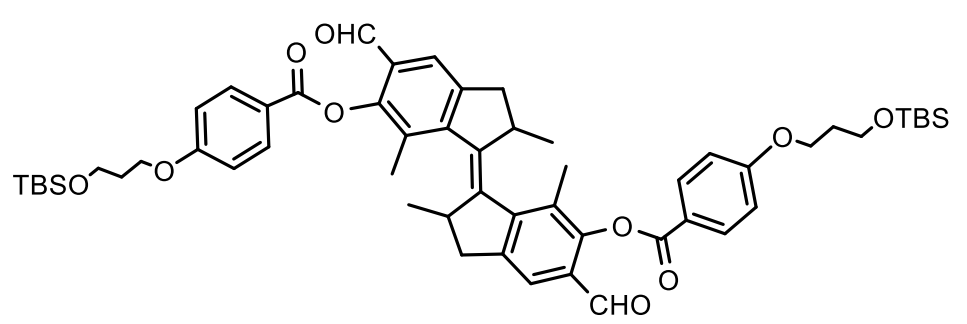

The compound **CHO** (100 mg, 0.27 mmol), 4-(3-((tert-butyldimethylsilyl)oxy)propoxy)benzoic acid (206 mg, 0.67 mmol) and 4-(Dimethylamino)pyridine (DMAP, 65 mg, 0.53 mmol) were dissolved in dry-DCM under nitrogen atmosphere. *N*,*N*'-Dicyclohexylcarbodiimide (DCC, 121 mg, 0.59 mmol) was added into the solution and the mixture

was stirred at room temperature for 6 h. The mixture was quenched with water (100 mL) and the product was extracted with EtOAc (3 × 50 mL). The combined organic layers were washed with brine, then dried over Na_2SO_4. The solvent was removed in vacuo and the residue was purified by column chromatography (SiO2, pentane: EtOAc= 5:1) to yield **CHO-L-C3OTBS** as a yellow solid (225 mg, 0.23 mmol, 88 %).

^{1}H NMR (600 MHz, Chloroform-*d*) δ 10.16 (s, 1H), 8.25 (d, *J* = 8.9 Hz, 2H), 7.70 (s, 1H), 7.07 (d, *J* = 8.9 Hz, 2H), 4.22 (t, *J* = 6.2 Hz, 2H), 3.86 (t, *J* = 5.9 Hz, 2H), 3.01 (p, *J* = 6.4 Hz, 1H), 2.91 (dd, *J* = 14.7, 5.7 Hz, 1H), 2.43 (d, *J* = 14.6 Hz, 1H), 2.09 – 2.03 (m, 2H), 1.15 (d, *J* = 6.5 Hz, 3H).

^{13}C NMR (151 MHz, $CDCl_3$) δ 188.7, 164.7, 164.0, 151.3, 148.5, 143.8, 142.1, 132. 6, 128.4, 123.3, 120.5, 114.6, 64.9, 59.2, 43.2, 40.2, 32.2, 25.9, 18.6, 18.3, 16.3, -5.4.

HRMS measurement for this compound was not successful.

(*E*)-5,5'-dicyano-2,2',7,7'-tetramethyl-2,2',3,3'-tetrahydro-[1,1'-biindenylidene]-6,6'-diyl bis(4-(3-hydroxypropoxy)benzoate) (CN-L-C3OH)

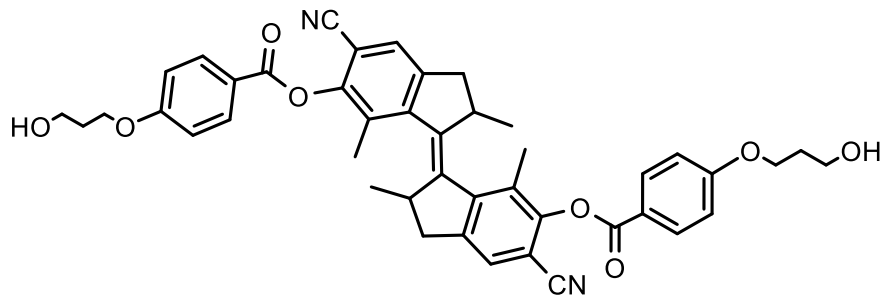

Compound **CHO-L-C3OTBS** (230 mg, 0.24 mmol) and O-(Diphenylphosphoryl)hydroxylamine (DPPH, 128.7 mg, 0.55 mmol) were dissovled in dry-toluene (6 mL). The mixture was stirred at room temperature under nitrogen for 3 h. The reaction was then heated to 95 °C for 12 h. After cooling to room temperature, water (20 mL) was added to quench the reaction and the mixture was extracted with DCM (3 × 20 mL). The combined organic layers were washed with brine (20 mL). then dried over Na_2CO_3 and concentrated under vacuum. The crude product was purified by flash column chromatography on silica gel (SiO_2, pentane: EtOAc= 1: 2) to give a mixture of unilateral/bilateral silyl-protected compounds. It was then hydrolyzed in tetrahydrofuran: water: acetic acid (20 mL: 20mL: 60 mL) under 40 °C for overnight. The volume of the solution was reduced by 50 % via evaporation under reduced pressure. DCM was added to slurry for washing sequentially with H_2O, a 10 % $NaHCO_3$ solution and brine before drying over anhydrous Na_2CO_3. The crude product was purified by column chromatography (SiO_2, pentane: EtOAc= 1: 2) to yield motor **CN-L-C3OH** (61 mg, 0.08 mmol, 35 %) as yellow solid.

^{1}H NMR (600 MHz, Chloroform-*d*) δ 8.25 (d, *J* = 8.9 Hz, 2H), 7.44 (s, 1H), 7.06 (d, *J* = 8.9 Hz, 2H), 4.26 (t, *J* = 6.1 Hz, 2H), 3.92 (t, *J* = 5.9 Hz, 2H), 2.98 (q, *J* = 6.3 Hz, 1H), 2.89 (dd, *J* = 14.5, 5.5 Hz, 1H), 2.40 (d, *J* = 14.8 Hz, 1H), 2.35 (s, 3H), 2.13 (p, *J* = 6.0 Hz, 2H), 1.13 (d, *J* = 6.5 Hz, 3H).

^{13}C NMR (151 MHz, Chloroform-*d*) δ 163.8, 151.0, 147.2, 143.3, 142.2, 132.8, 129.1, 126.8, 120.6, 116.0, 114.6, 106.1, 65.7, 59.9, 43.2, 40.1, 31.9, 18.5, 16.7.

HRMS (ESI, m/z) calculated for $C_{44}H_{42}N_2O_8Na$ $[M+Na]^+$: 749.2836, found 749.2833.

(*E*)-5,5'-dicyano-2,2',7,7'-tetramethyl-2,2',3,3'-tetrahydro-[1,1'-biindenylidene]-6,6'-diyl bis(4-(3-(acryloyloxy)propoxy)benzoate) (CN-L-C3)

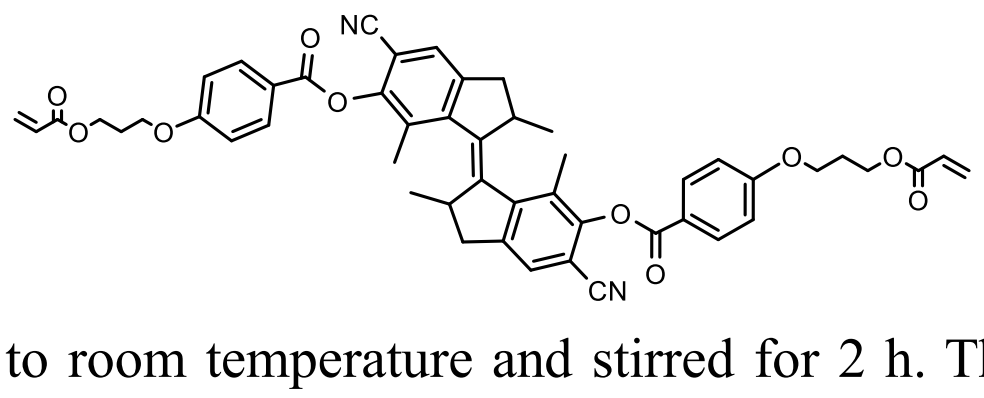

To a solution of **CN-L-C3OH** (50 mg, 0.07 mmol) in THF (5 mL), triethylamine (139.3 mg, 1.38 mmol) and acryloyl chloride (11.2 mg, 0.16 mmol) were added at 0 °C. The mixture was allowed to warm up to room temperature and stirred for 2 h. The mixture was quenched with water (20 mL) and the

aqueous phase was extracted with EtOAc (3 × 20 mL). The combined organic layer was washed with saturated NH_4Cl solution (2 × 20 mL) and brine, then dried over Na_2SO_4. The solvent was removed in vacuo and the residue was purified by flash column (SiO2, pentane: EtOAc= 2:1) to yield motor **CN-L-C3** as yellow solid (23.9 mg, 0.02 mmol, 41 %).

^{1}H NMR (600 MHz, Chloroform-*d*) δ 8.25 (d, *J* = 8.6 Hz, 2H), 7.44 (s, 1H), 7.05 (d, *J* = 8.6 Hz, 2H), 6.45 (dt, *J* = 17.3, 1.3 Hz, 1H), 6.17 (dd, *J* = 17.3, 10.5 Hz, 1H), 5.91 – 5.86 (m, 1H), 4.42 (t, *J* = 6.2 Hz, 2H), 4.21 (t, *J* = 6.1 Hz, 2H), 2.97 (p, *J* = 6.3 Hz, 1H), 2.89 (dd, *J* = 14.8, 5.7 Hz, 1H), 2.40 (d, *J* = 14.7 Hz, 1H), 2.35 (s, 3H), 2.25 (p, *J* = 6.2 Hz, 2H), 1.13 (d, *J* = 6.4 Hz, 3H).

^{13}C NMR (151 MHz, Chloroform-*d*) δ 166.2, 163.7, 163.6, 151.0, 147.2, 143.3, 142.2, 132.8, 131.1, 129.1, 128.3, 126.8, 120.6, 116.0, 114.6, 106.1, 64.8, 61.1, 43.2, 40.1, 28.5, 18.5, 16.7.

HRMS (ESI, m/z) calculated for $C_{50}H_{46}N_2O_{10}Na$ $[M+Na]^+$: 857.3038, found 857.3045.

4-((6-((tert-butyldimethylsilyl)oxy)hexyl)oxy)benzoic acid

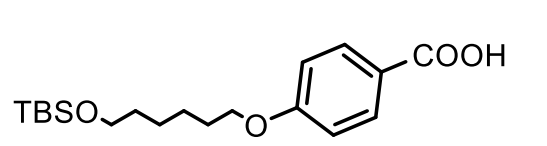

Compound **C6OTBS** was synthesized by following the procedure in ref [2].

^{1}H NMR (401 MHz, DMSO-d_6) δ 7.85 (d, *J* = 8.5 Hz, 2H), 6.97 (d, *J* = 8.5 Hz, 2H), 4.00 (t, *J* = 6.4 Hz, 2H), 3.56 (t, *J* = 6.3 Hz, 2H), 1.70 (p, *J* = 6.8 Hz, 2H), 1.51 – 1.31 (m, 6H), 0.83 (s, 9H), -0.00 (s, 6H).

(*E*)-5,5'-diformyl-2,2',7,7'-tetramethyl-2,2',3,3'-tetrahydro-[1,1'-biindenylidene]-6,6'-diyl bis(4-((6-((tert-butyldimethylsilyl)oxy)hexyl)oxy)benzoate) (CHO-L-C6OTBS)

Under nitrogen atmosphere, the compound **CHO** (100 mg, 0.27 mmol), 4-((6-((tert-butyldimethylsilyl)oxy)hexyl)oxy)benzoic acid (234 mg, 0.66 mmol) and 4-(Dimethylamino)pyridine (DMAP, 65 mg, 0.53 mmol) were dissolved in dry-DCM. *N*,*N'*-Dicyclohexylcarbodiimide (DCC, 121 mg, 0.58 mmol) was added into the solution and the mixture was stirred at room temperature for 6 h. The mixture was quenched with water (100 mL) and the product was extracted with EtOAc (3 × 50 mL). The combined organic layers were washed with brine, then dried over Na_2SO_4. The solvent was removed in vacuo and the residue was purified by column chromatography (SiO2, pentane: EtOAc= 6:1) to yield **CHO-L-C6OTBS** as a yellow solid (250 mg, 0.24 mmol, 90 %).

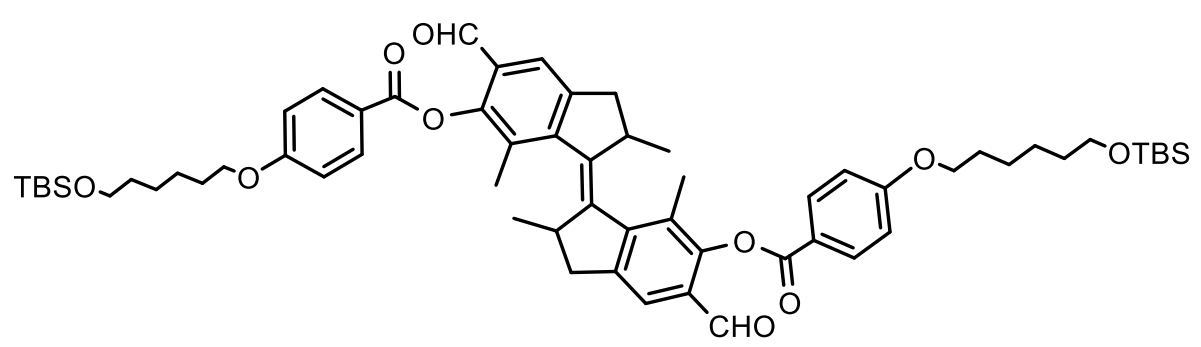

^{1}H NMR (600 MHz, Chloroform-*d*) δ 10.16 (s, 1H), 8.25 (d, *J* = 8.9 Hz, 2H), 7.70 (s, 1H), 7.05 (d, *J* = 8.9 Hz, 2H), 4.10 (t, *J* = 6.5 Hz, 2H), 3.66 (t, *J* = 6.5 Hz, 2H), 3.01 (p, *J* = 6.4 Hz, 1H), 2.91 (dd, *J* = 14.7, 5.7 Hz, 1H), 2.43 (d, *J* = 14.6 Hz, 1H), 2.37 (s, 3H), 1.90 – 1.85 (m, 2H), 1.62 – 1.59 (m, 1H), 1.56 – 1.51 (m, 2H), 1.49 – 1.43 (m, 2H), 1.15 (d, *J* = 6.5 Hz, 3H).

^{13}C NMR (151 MHz, Chloroform-*d*) δ 188.7, 164.7, 164.0, 151.3, 148.5, 143.8, 142.1, 132.6, 128.4, 127.5, 123.3, 120.4, 114.6, 68.4, 63.1, 43.2, 40.2, 32.7, 29.1, 26.0, 25.8, 25.6, 18.6, 18.4, 16.3, -5.2.

HRMS (ESI, m/z) calculated for $C_{62}H_{84}O_{10}Si_2Na$ $[M+Na]^+$: 1067.5509, found 1067.5495.

(*E*)-5,5'-dicyano-2,2',7,7'-tetramethyl-2,2',3,3'-tetrahydro-[1,1'-biindenylidene]-6,6'-diyl bis(4-((6-hydroxyhexyl)oxy)benzoate) (CN-L-C6OH)

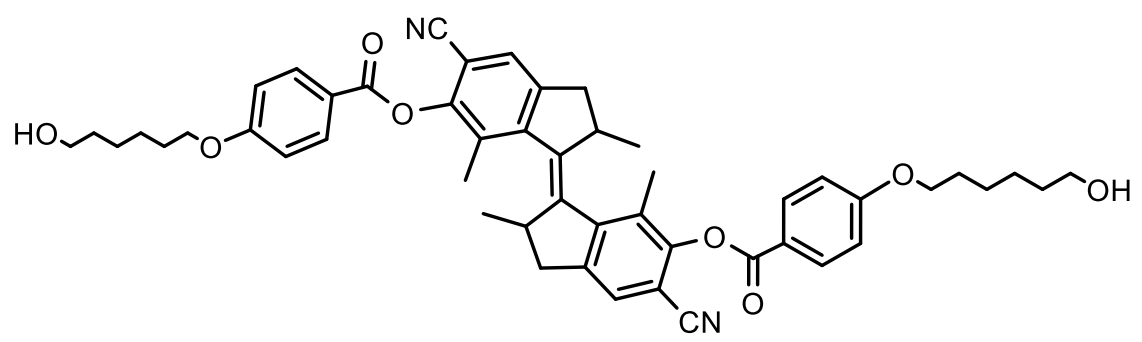

Compound **CHO-L-C6OTBS** (200 mg, 0.19 mmol) and O-(Diphenylphosphoryl)hydroxylamine (DPPH, 102.7 mg, 0.44 mmol) were dissovled in dry-toluene (6 mL). The mixture was stirred at room temperature under nitrogen for 3 h. The reaction was then heated to 95 ℃ for 12 h. After cooling to room temperature, water (20 mL) was added to quench the reaction and the mixture was extracted with DCM (3 × 20 mL). The combined organic layers were washed with brine (20 mL). then dried over Na_2CO_3 and concentrated under vacuum. The crude product was purified by flash column chromatography on silica gel (SiO_2, pentane: EtOAc= 1: 2) to give a mixture of unilateral/bilateral silyl-protected compounds. It was then hydrolyzed in tetrahydrofuran: water: acetic acid (20 mL: 20mL: 60 mL) under 40 ℃ for overnight. The volume of the solution was reduced by 50 % via evaporation under reduced pressure. DCM was added to slurry for washing sequentially with H_2O, a 10 % $NaHCO_3$ solution and brine before drying over anhydrous Na_2CO_3. The crude product was purified by column chromatography (SiO_2, pentane: EtOAc= 1: 2) to yield motor **CN-L-C6OH** (44.7 mg, 0.06 mmol, 29 %) as yellow solid.

^{1}H NMR (600 MHz, Chloroform-*d*) δ 8.25 (d, *J* = 8.9 Hz, 2H), 7.44 (s, 1H), 7.03 (d, *J* = 8.9 Hz, 2H), 4.10 (t, *J* = 6.5 Hz, 2H), 3.71 (t, *J* = 6.5 Hz, 2H), 2.97 (p, *J* = 6.4 Hz, 1H), 2.89 (dd, *J* = 14.8, 5.7 Hz, 1H), 2.40 (d, *J* = 14.7 Hz, 1H), 2.35 (s, 3H), 1.92 – 1.85 (m, 2H), 1.70 – 1.62 (m, 2H), 1.58 – 1.53 (m, 2H), 1.50 (m, *J* = 7.1, 3.0 Hz, 2H), 1.13 (d, *J* = 6.5 Hz, 3H).

^{13}C NMR (151 MHz, Chloroform-*d*) δ 164.0, 163.8, 151.1, 147.2, 143.3, 142.2, 132.7, 126.7, 120.2, 116.0, 114.6, 106.1, 68.3, 62.9, 43.1, 40.1, 32.7, 29.1, 25.8, 25.5, 18.5, 16.7.

HRMS (ESI, m/z) calculated for $C_{50}H_{54}N_2O_8Na$ $[M+Na]^+$: 833.3768, found 833.3772.

(*E*)-5,5'-dicyano-2,2',7,7'-tetramethyl-2,2',3,3'-tetrahydro-[1,1'-biindenylidene]-6,6'-diyl bis(4-((6-(acryloyloxy)hexyl)oxy)benzoate) (CN-L-C6)

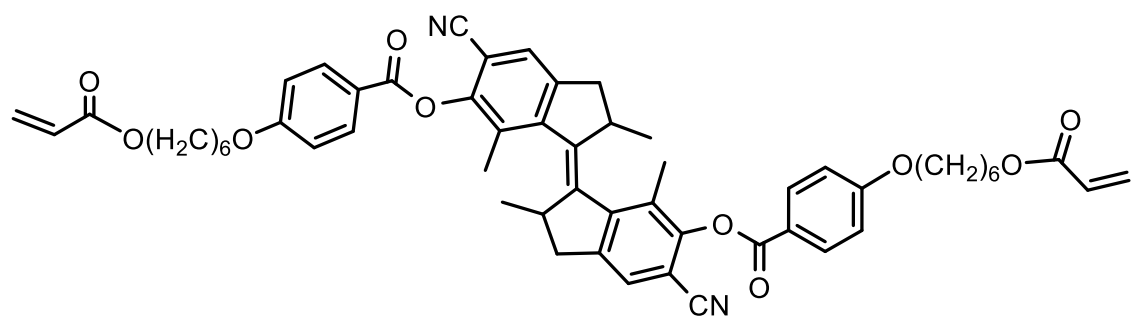

To a solution of **CN-L-C6OH** (30 mg, 0.04 mmol) in THF (5 mL), triethylamine (75 mg, 0.74 mmol) and acryloyl chloride (7.7 mg, 0.09 mmol) were added at 0 °C. The mixture was allowed to warm up to room temperature and stirred for 2 h. The mixture was quenched with water (20 mL) and the aqueous phase was extracted with EtOAc (3 × 20 mL). The combined organic layer was washed with saturated NH_4Cl solution (2 × 20 mL) and brine, then dried over Na_2SO_4. The solvent was removed in vacuo and the residue was purified by flash column (SiO2, pentane: EtOAc= 2:1) to yield motor **CN-L-C6** as yellow solid (16.7 mg, 0.02 mmol, 49 %).

^{1}H NMR (600 MHz, Chloroform-*d*) δ 8.25 (d, *J* = 8.8 Hz, 2H), 7.44 (s, 1H), 7.03 (d, *J* = 8.9 Hz, 2H), 6.43 (dd, *J* = 17.3, 1.5 Hz, 1H), 6.15 (dd, *J* = 17.3, 10.4 Hz, 1H), 5.85 (dd, *J* = 10.4, 1.5 Hz, 1H), 4.21 (t, *J* = 6.6 Hz, 2H), 4.10 (t, *J* = 6.5 Hz, 2H), 2.97 (p, *J* = 6.4 Hz, 1H), 2.89 (dd, *J* = 14.8, 5.7 Hz, 1H), 2.40 (d, *J* = 14.7 Hz, 1H), 2.35 (s, 3H), 1.90 – 1.85 (m, 2H), 1.80 – 1.73 (m, 2H), 1.62 – 1.54 (m, 2H), 1.13 (d, *J* = 6.5 Hz, 3H).

^{13}C NMR (151 MHz, Chloroform-*d*) δ 166.4, 164.0, 163.8, 151.1, 147.2, 143.3, 142.2, 132.7, 130.6, 129.1, 128.6, 126.7, 120.3, 116.0, 114.6, 106.1, 68.2, 64.5, 43.1, 40.1, 29.0, 28.6, 25.7 (d, *J* = 4.8 Hz), 18.5, 16.7.

HRMS (ESI, m/z) calculated for $C_{56}H_{58}N_2O_{10}Na$ $[M+Na]^+$: 941.3986, found 941.3984.

(*E*)-((5,5'-diformyl-2,2',7,7'-tetramethyl-2,2',3,3'-tetrahydro-[1,1'-biindenylidene]-6,6'-diyl)bis(oxy))bis(hexane-6,1-diyl) diacrylate (CHO-S)

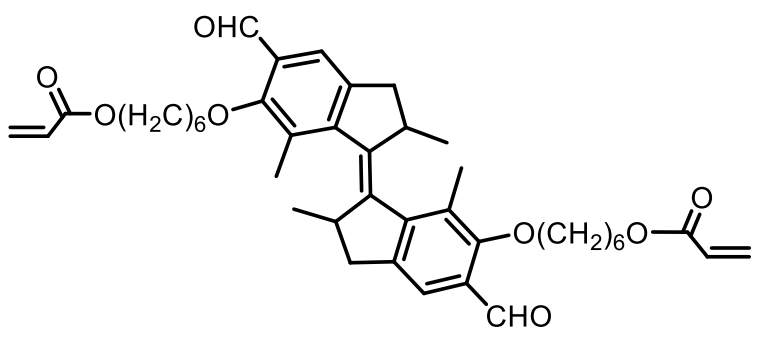

To a solution of **CHO** (100 mg, 0.27 mmol) in MeCN (10 mL), K_2CO_3 (110.6 mg, 0.8 mmol), tetrabutylammonium iodide (295.4 mg, 0.8 mmol) and 6-bromohexyl acrylate (249 mg, 1.06 mmol) were added. The mixture was stirred at 85 °C under nitrogen for 16 h. After cooling to room temperature, the mixture was acidified using a saturated aqueous NH_4Cl solution to pH = 6-7. The product was extracted with EtOAc (3 × 50 mL). The combined organic layers were washed with brine (100 mL), then dried over Na_2CO_3. The solvent was removed in vacuo and the residue was purified by column chromatography (SiO_2, pentane: EtOAc= 3: 1) to yield motor **CHO-S** (115 mg, 0.17 mmol, 63 %) as yellow oil.

^{1}H-NMR (600 MHz, Chloroform-*d*) δ 10.39 (s, 1H), 7.59 (s, 1H), 6.44 (dt, *J* = 17.3, 1.7 Hz, 1H), 6.16 (ddd, *J* = 17.2, 10.4, 1.5 Hz, 1H), 5.85 (dd, *J* = 10.5, 1.7 Hz, 1H), 4.22 (td, *J* = 6.6, 1.5 Hz, 2H), 4.13 – 4.08 (m, 1H), 3.94 (ddd, *J* = 9.9, 7.3, 5.6 Hz, 1H), 2.90 (p, *J* = 6.2 Hz, 1H), 2.80 (dd, *J* = 14.4, 5.7 Hz, 1H), 2.44 (s, 3H), 2.35 (d, *J* = 14.3 Hz, 1H), 1.94 (t, *J* = 7.6 Hz, 2H), 1.77 (q, *J* = 7.2 Hz, 2H), 1.67 – 1.60 (m, 2H), 1.53 (p, *J* = 8.7, 8.3 Hz, 2H), 1.14 (dd, *J* = 6.5, 1.4 Hz, 3H).

^{13}C NMR (151 MHz, Chloroform-*d*) δ 190.3, 166.4, 161.0, 149.1, 143.8, 139.9, 130.6, 128.6 (d, *J* = 2.7 Hz), 128.1, 121.9, 76.2, 64.5, 43.2, 34.0, 30.1, 28.6, 25.9, 25.8, 18.5, 16.4, 1.0.

HRMS (ESI, m/z) calculated for $C_{42}H_{52}O_8Na$ $[M+Na]^+$: 707.3552, found 707.3554.

(*E*)-5,5'-diformyl-2,2',7,7'-tetramethyl-2,2',3,3'-tetrahydro-[1,1'-biindenylidene]-6,6'-diyl bis(4-((6-(acryloyloxy)hexyl)oxy)benzoate) (CHO-L)

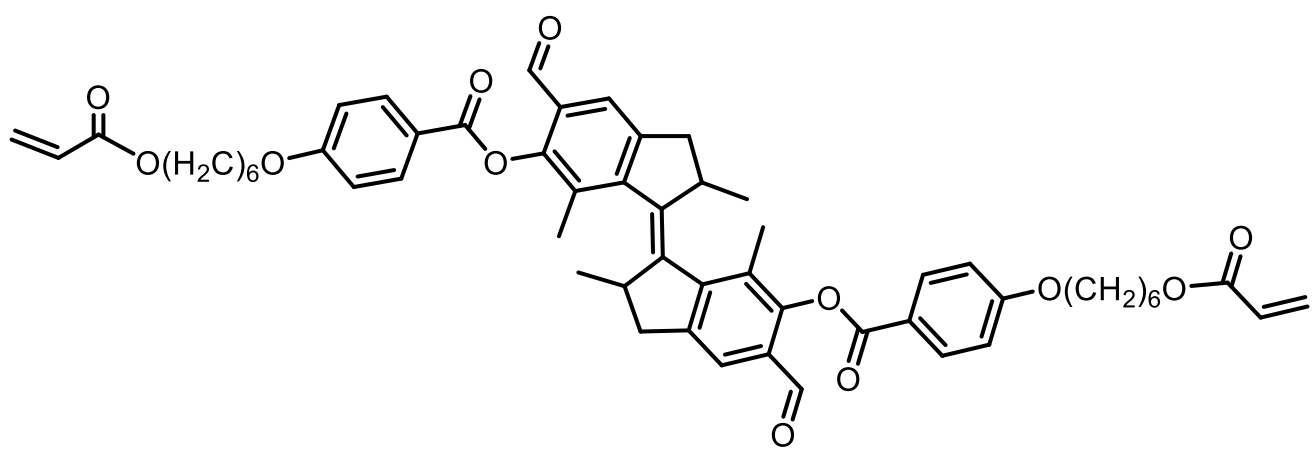

Compound **CHO-L** was synthesized following the procedure in ref [3]. ^{1}H NMR (400 MHz, Chloroform-d) δ 10.13 (s, 1H), 8.23 (d, *J* = 8.9 Hz, 2H), 7.67 (s, 1H), 7.02 (d, *J* = 8.9 Hz, 2H), 6.41 (dd, *J* = 17.3, 1.5 Hz, 1H), 6.13 (dd, *J* = 17.3, 10.4 Hz, 1H), 5.83 (dd, *J* = 10.4, 1.5 Hz, 1H), 4.19 (t, *J* = 6.6 Hz, 2H), 4.08 (t, *J* = 6.4 Hz, 2H), 2.98 (p, *J* = 6.4 Hz, 1H), 2.89 (dd, *J* = 14.7, 5.7 Hz, 1H), 2.40 (d, *J* = 14.6 Hz, 1H), 2.35 (s, 3H), 1.92 – 1.81 (m, 2H), 1.74 (p, *J* = 6.8 Hz, 2H), 1.59 – 1.41 (m, 6H), 1.12 (d, *J* = 6.4 Hz, 3H).

(*E*)-6-((9-(6-((6-(acryloyloxy)hexyl)oxy)-2,4,7-trimethyl-2,3-dihydro-1H-inden-1-ylidene)-9H-fluoren-2-yl)oxy)hexyl acrylate (MSG)

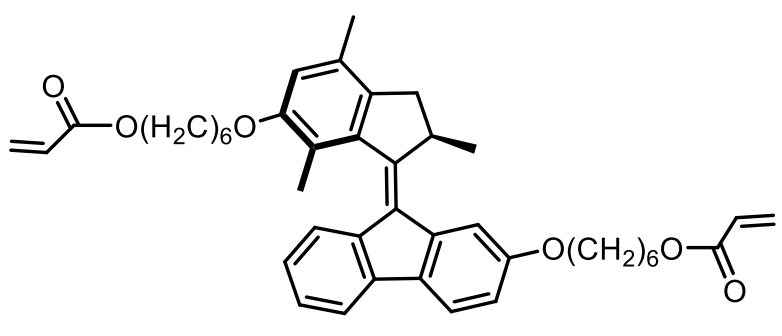

Compound **MSG** was synthesized following the procedure in ref[4].

^{1}H NMR (600 MHz, Chloroform-*d*) δ 7.71 – 7.63 (m, 2H), 7.45 (d, *J* = 2.2 Hz, 1H), 7.34 (dd, *J* = 7.8, 1.0 Hz, 1H), 7.25 (td, *J* = 7.4, 1.1 Hz, 1H), 7.07 (td, *J* = 7.5, 1.2 Hz, 1H), 6.94 (dd, *J* = 8.3, 2.2 Hz, 1H), 6.73 (s, 1H), 6.42 (ddd, *J* = 17.4, 5.7, 1.5 Hz, 2H), 6.15 (ddd, *J* = 17.2, 10.4, 6.3 Hz, 2H), 5.83 (ddd, *J* = 10.4, 6.7, 1.4 Hz, 2H), 4.25 – 4.08 (m, 8H), 4.02 – 3.97 (m, 1H), 3.18 (dd, *J* = 14.4, 5.8

Hz, 1H), 2.55 (d, *J* = 14.4 Hz, 1H), 2.33 (s, 3H), 2.12 (s, 3H), 1.94 – 1.82 (m, 4H), 1.80 – 1.69 (m, 4H), 1.66 – 1.55 (m, 4H), 1.55 – 1.45 (m, 4H), 1.35 (d, *J* = 6.7 Hz, 3H).

3 Characterization of compounds

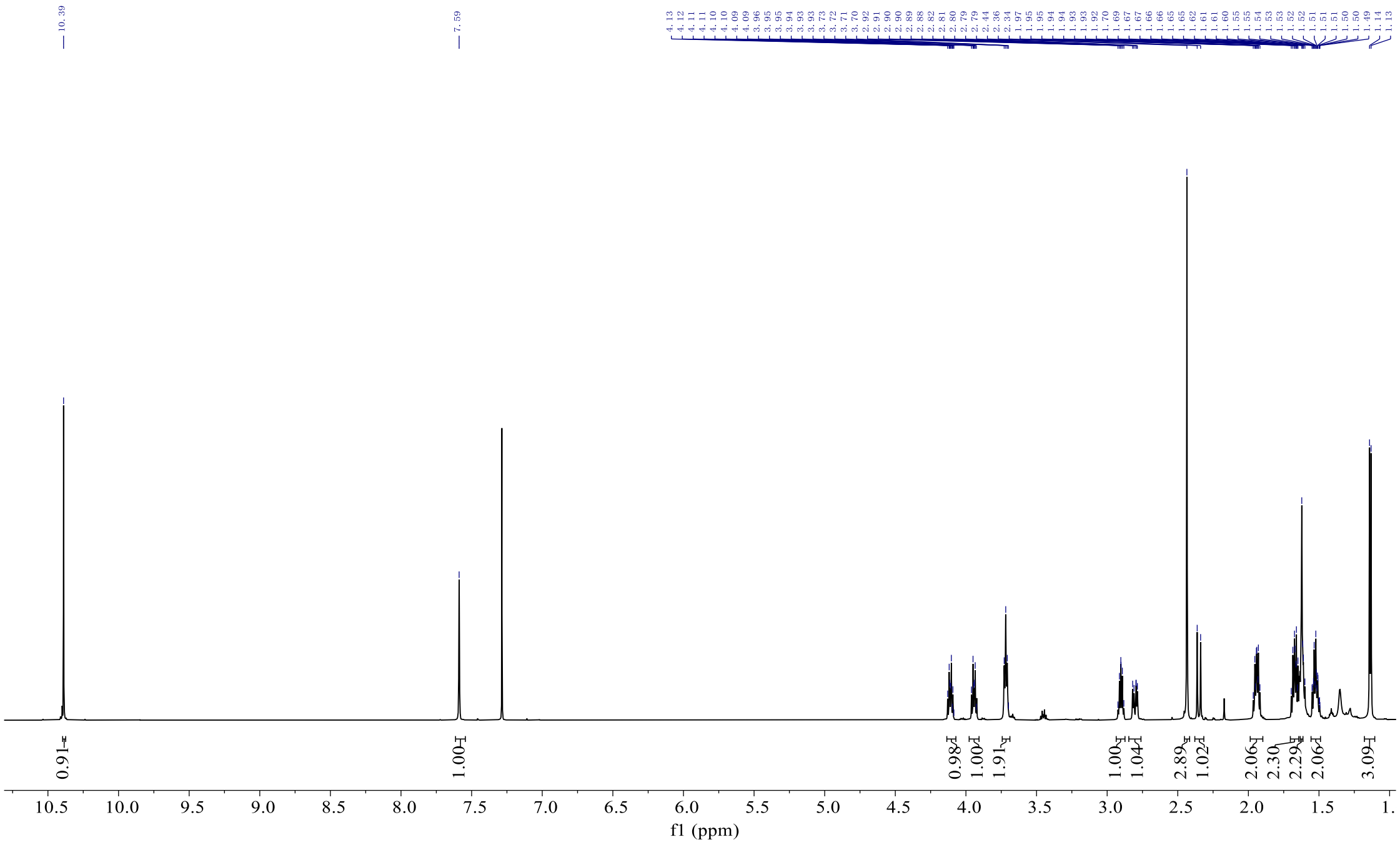

Supplementary Fig. 2 | NMR characterization. ^{1}H-NMR spectrum of compound **CHO-OH** in $CDCl_3$ at 600 MHz.

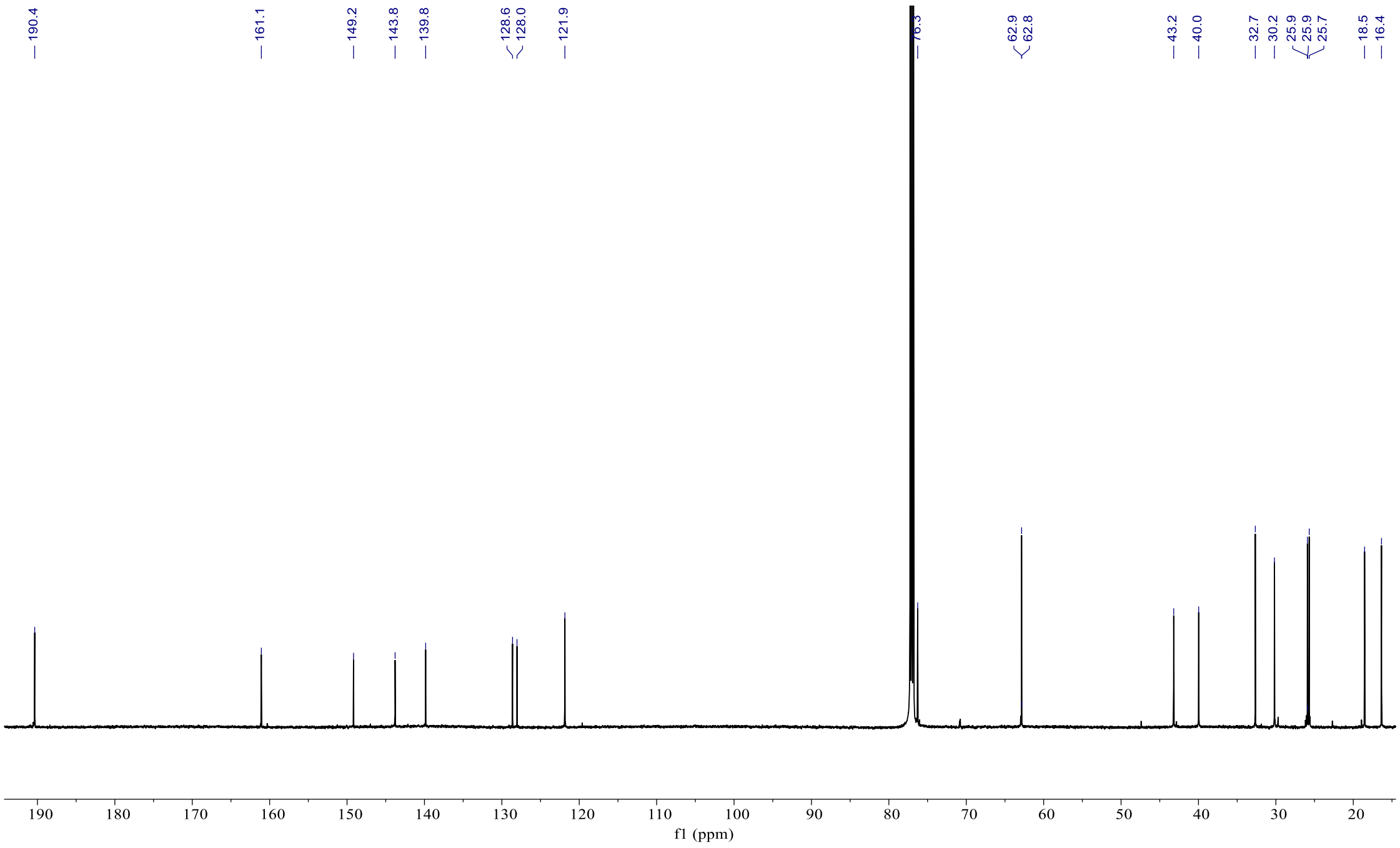

Supplementary Fig. 3 | NMR characterization. ^{13}C-NMR spectrum of compound **CHO-OH** in $CDCl_3$ at 151 MHz.

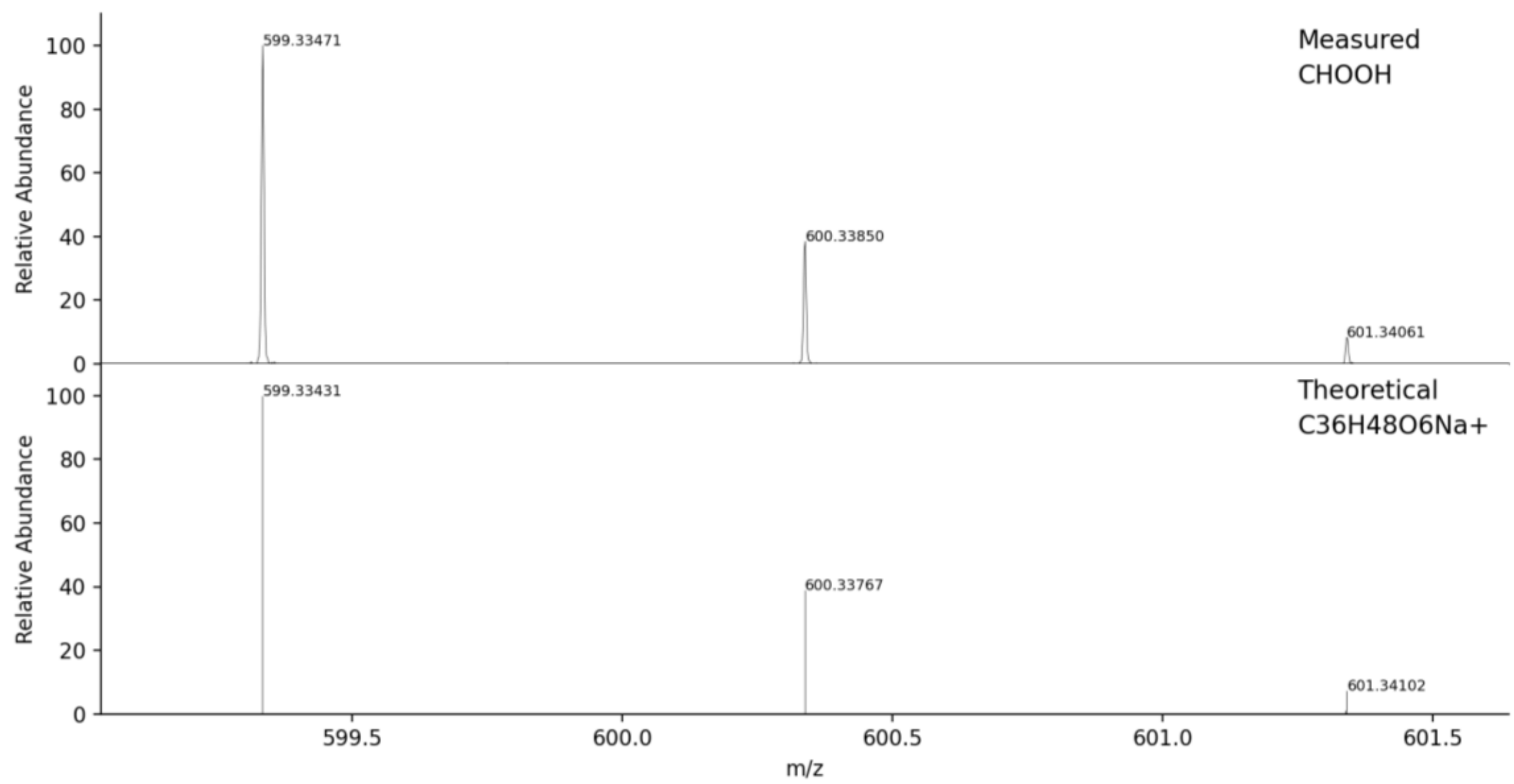

Supplementary Fig. 4 | HRMS characterization. HRMS spectrum of compound **CHO-OH.**

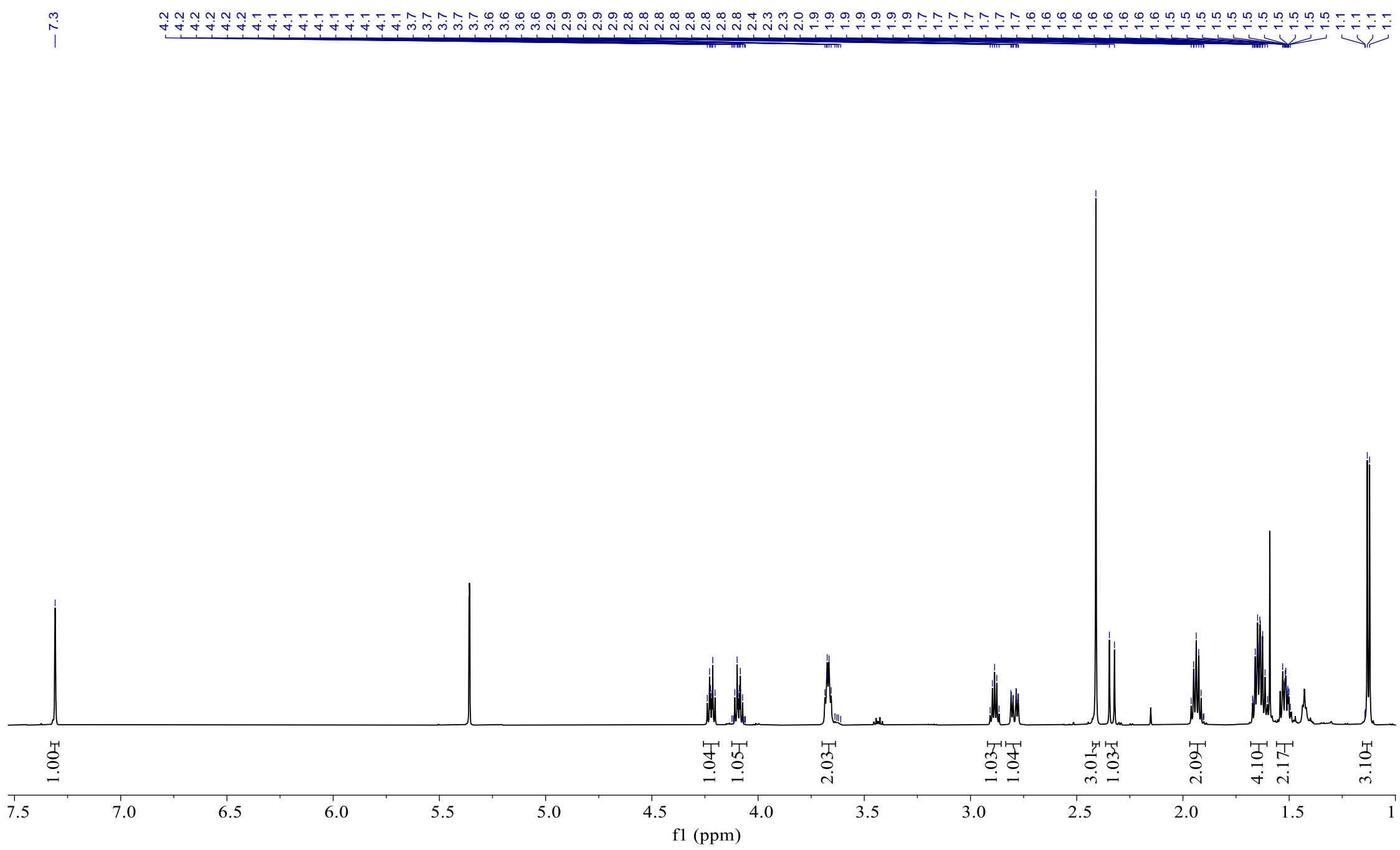

Supplementary Fig. 5 | NMR characterization. ^{1}H-NMR spectrum of compound **CN-OH** in CD_2Cl_2 at 600 MHz.

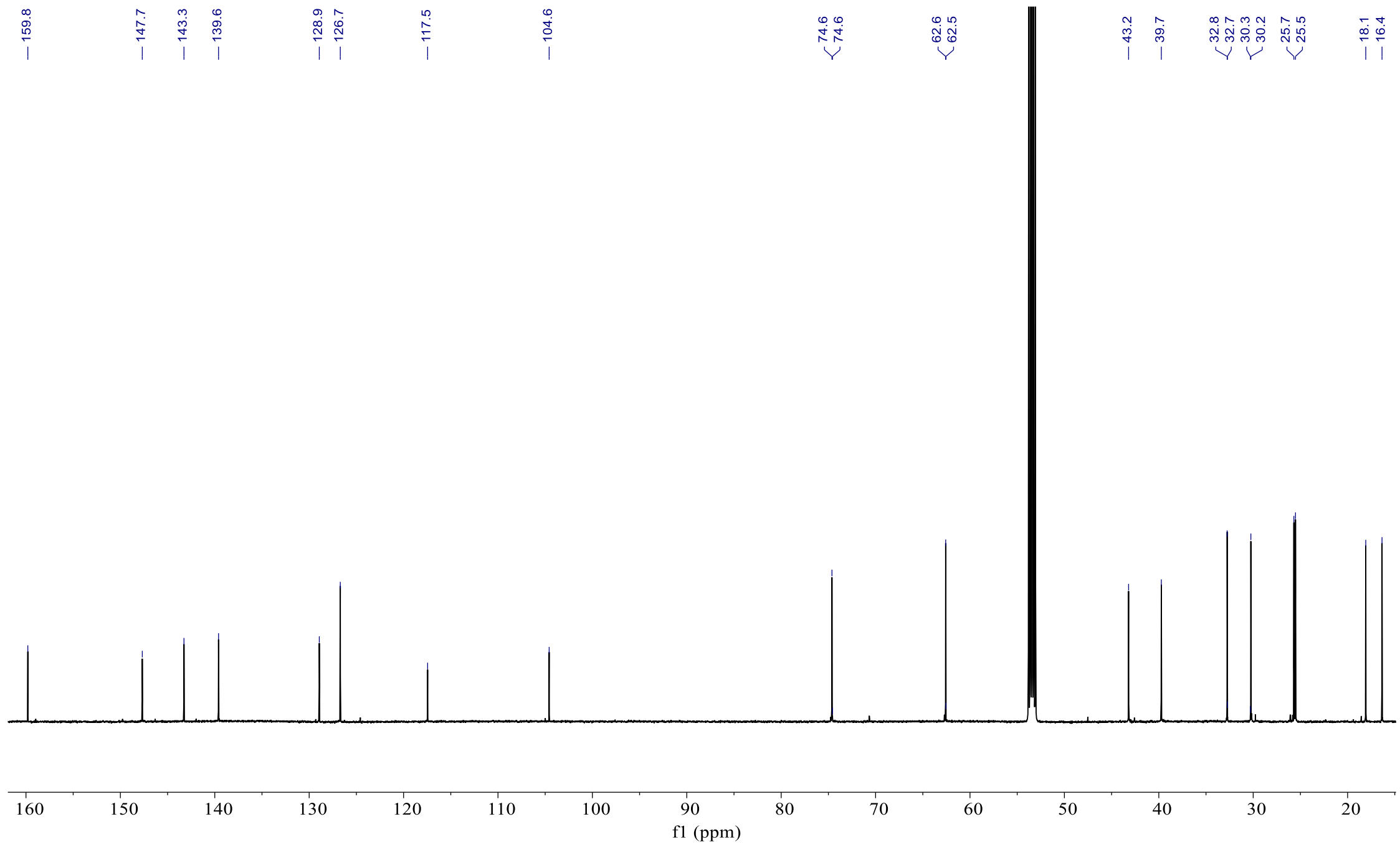

Supplementary Fig. 6 | NMR characterization. ^{13}C-NMR spectrum of compound **CN-OH** in $CDCl_2$ at 151 MHz.

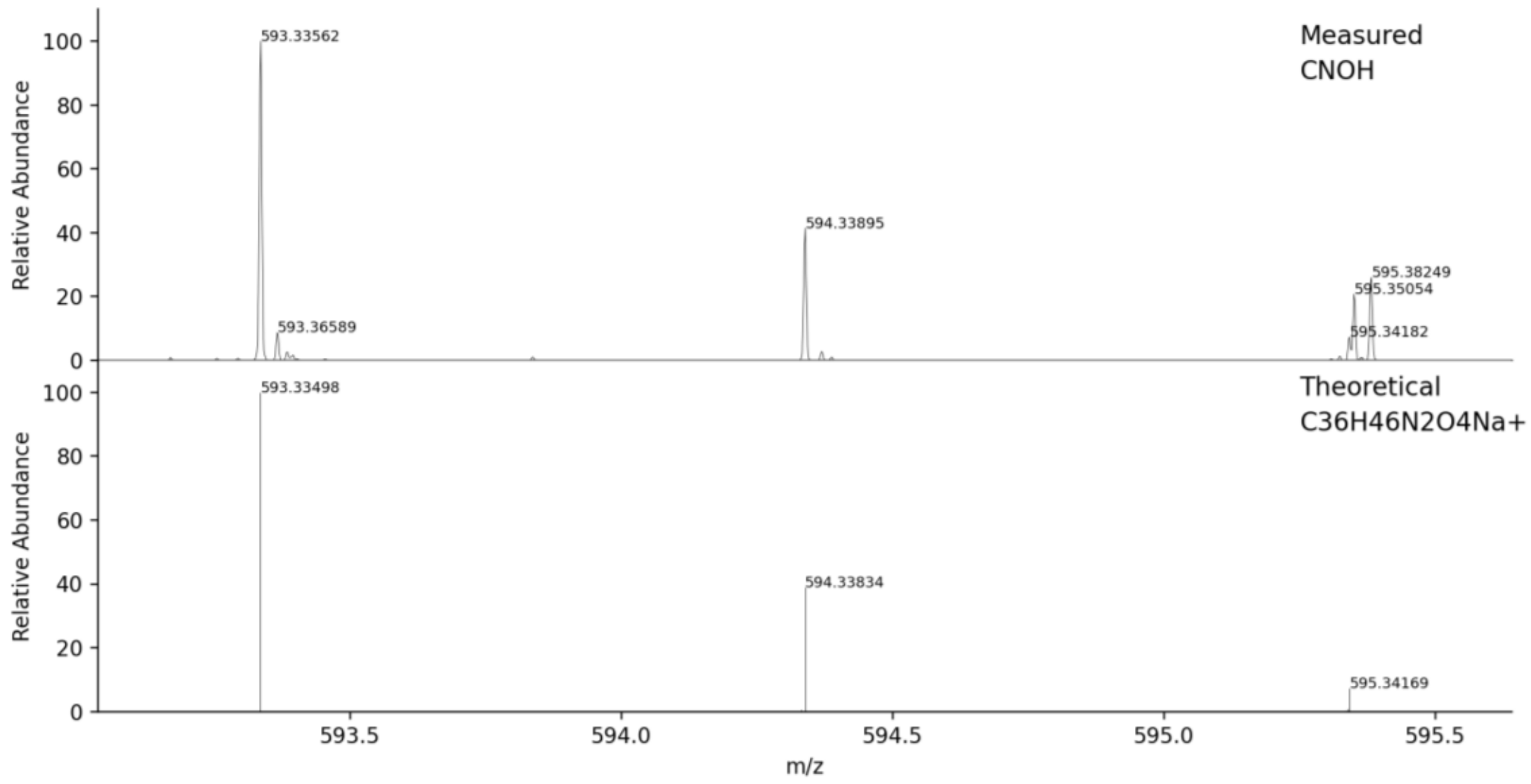

Supplementary Fig. 7 | HRMS characterization. HRMS spectrum of compound **CN-OH.**

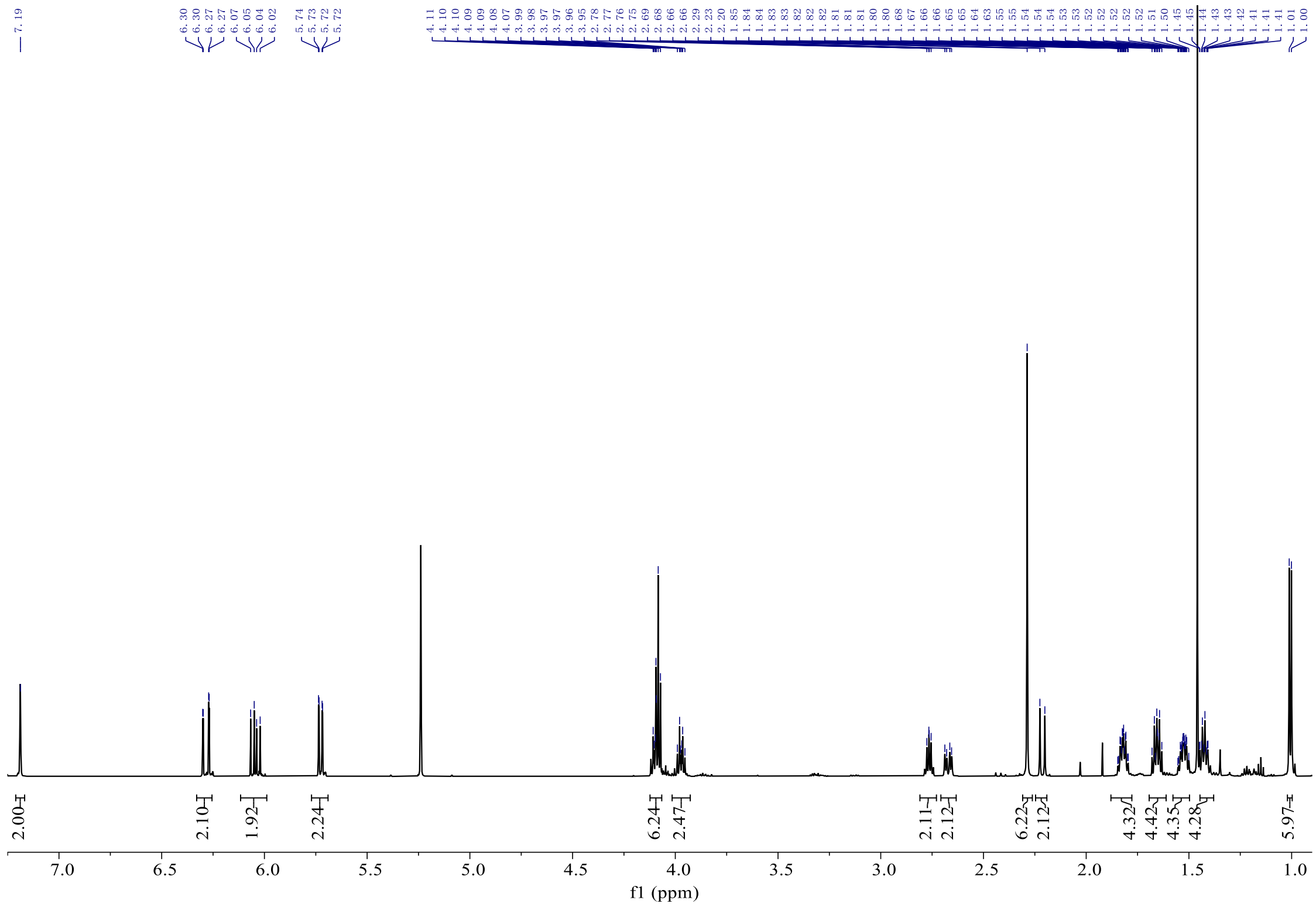

Supplementary Fig. 8 | NMR characterization. ^{1}H-NMR spectrum of compound **CN-S** in $CDCl_3$ at 600 MHz.

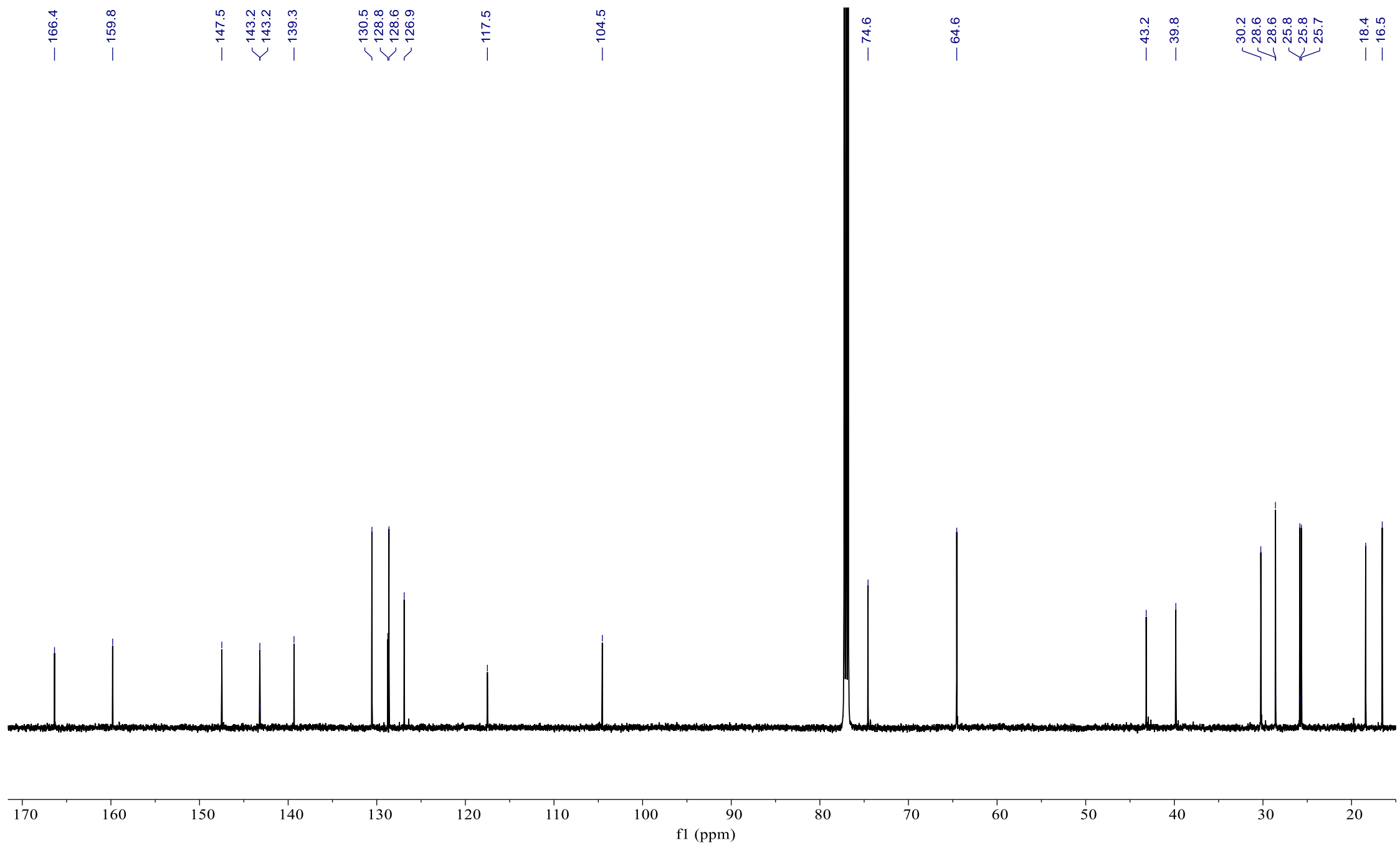

Supplementary Fig. 9 | NMR characterization. ^{13}C-NMR spectrum of compound **CN-S** in $CDCl_3$ at 151 MHz.

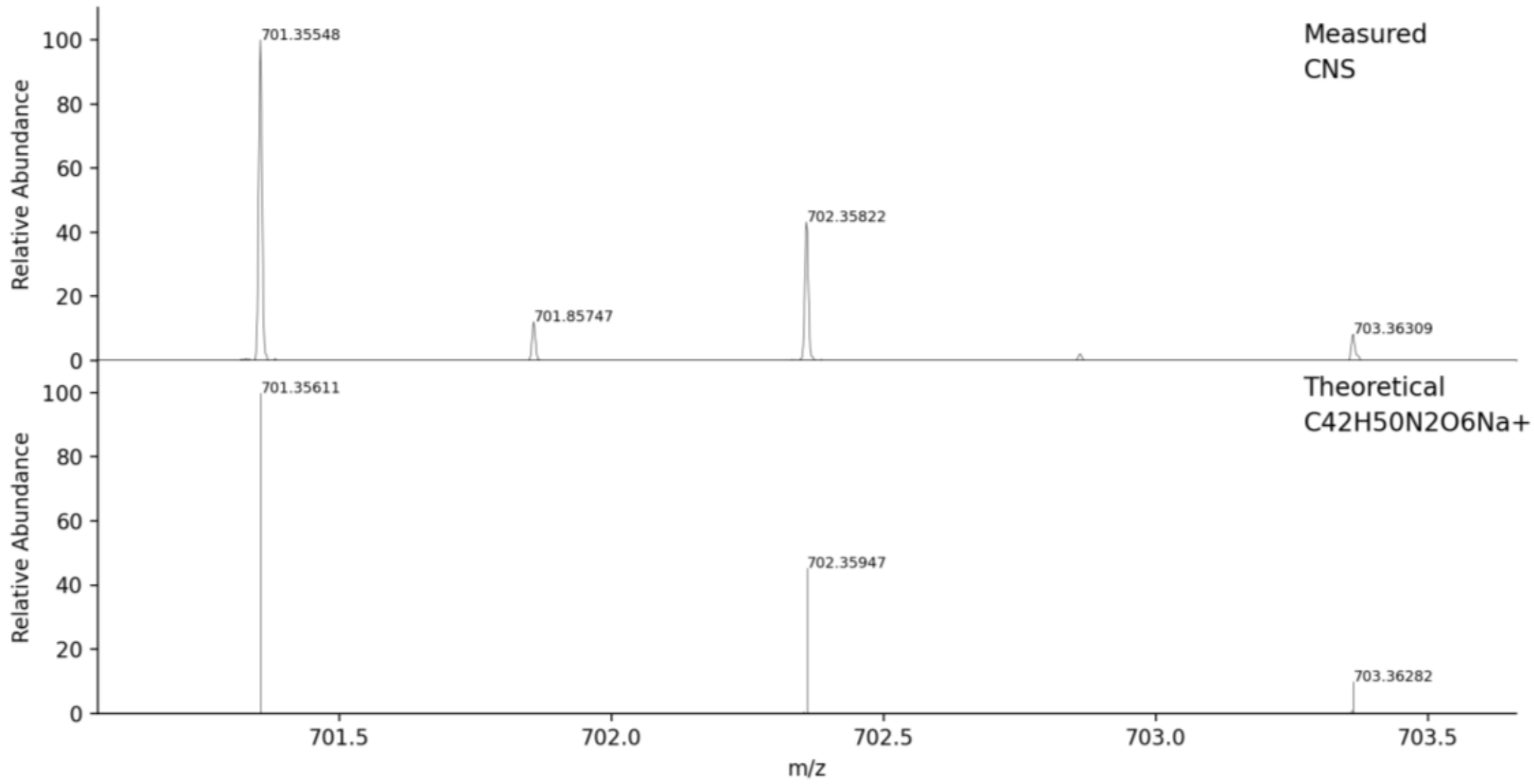

Supplementary Fig. 10 | HRMS characterization. HRMS spectrum of compound **CN-S.**

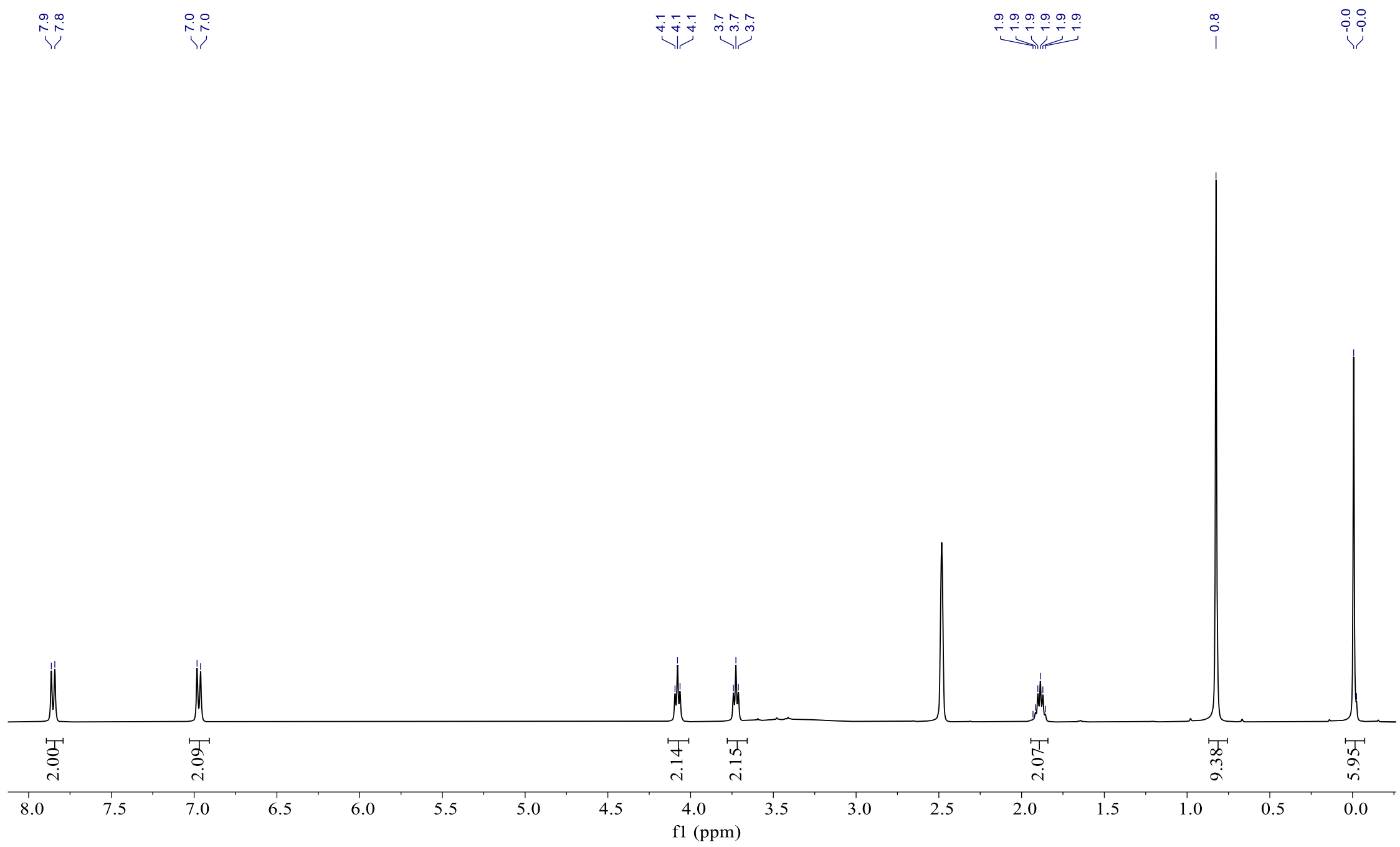

Supplementary Fig. 11 | NMR characterization. ^{1}H-NMR spectrum of compound **C3OTBS** in DMSO at 600 MHz.

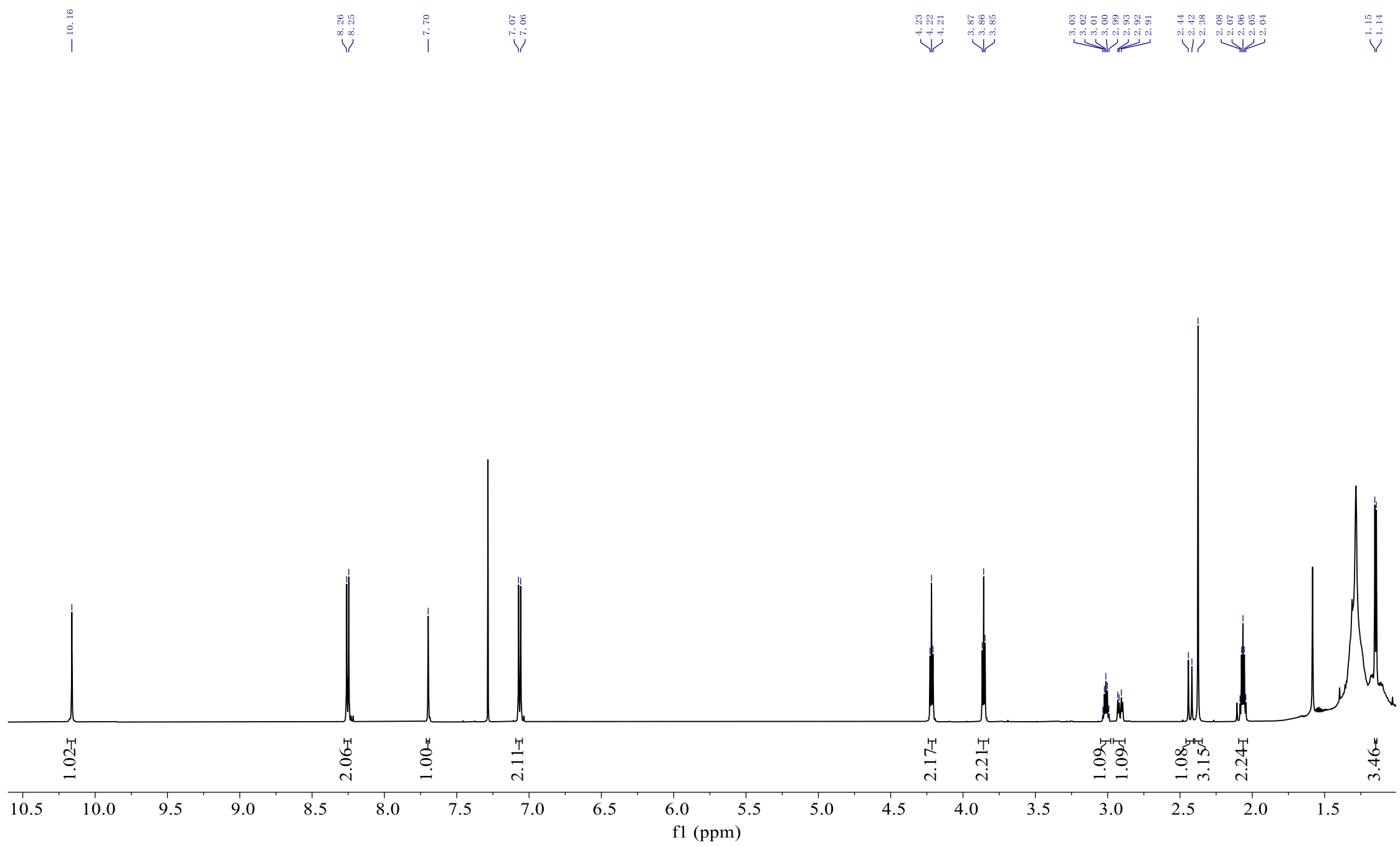

Supplementary Fig. 12 | NMR characterization. ^{1}H-NMR spectrum of compound **CHO-L-C3OTBS** in $CDCl_3$ at 600 MHz.

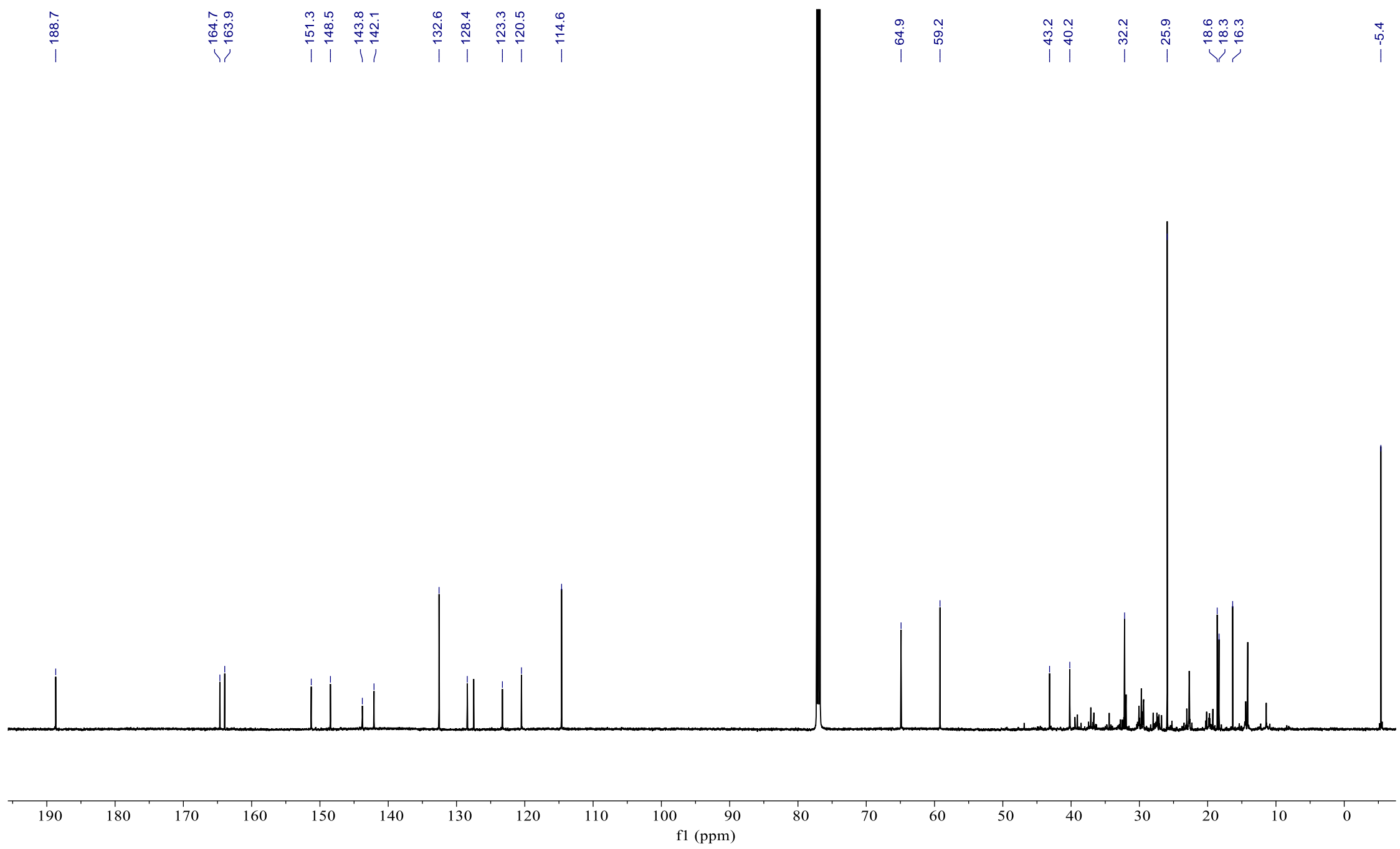

Supplementary Fig. 13 | NMR characterization. ^{13}C-NMR spectrum of compound **CHO-L-C3OTBS** in $CDCl_3$ at 151 MHz.

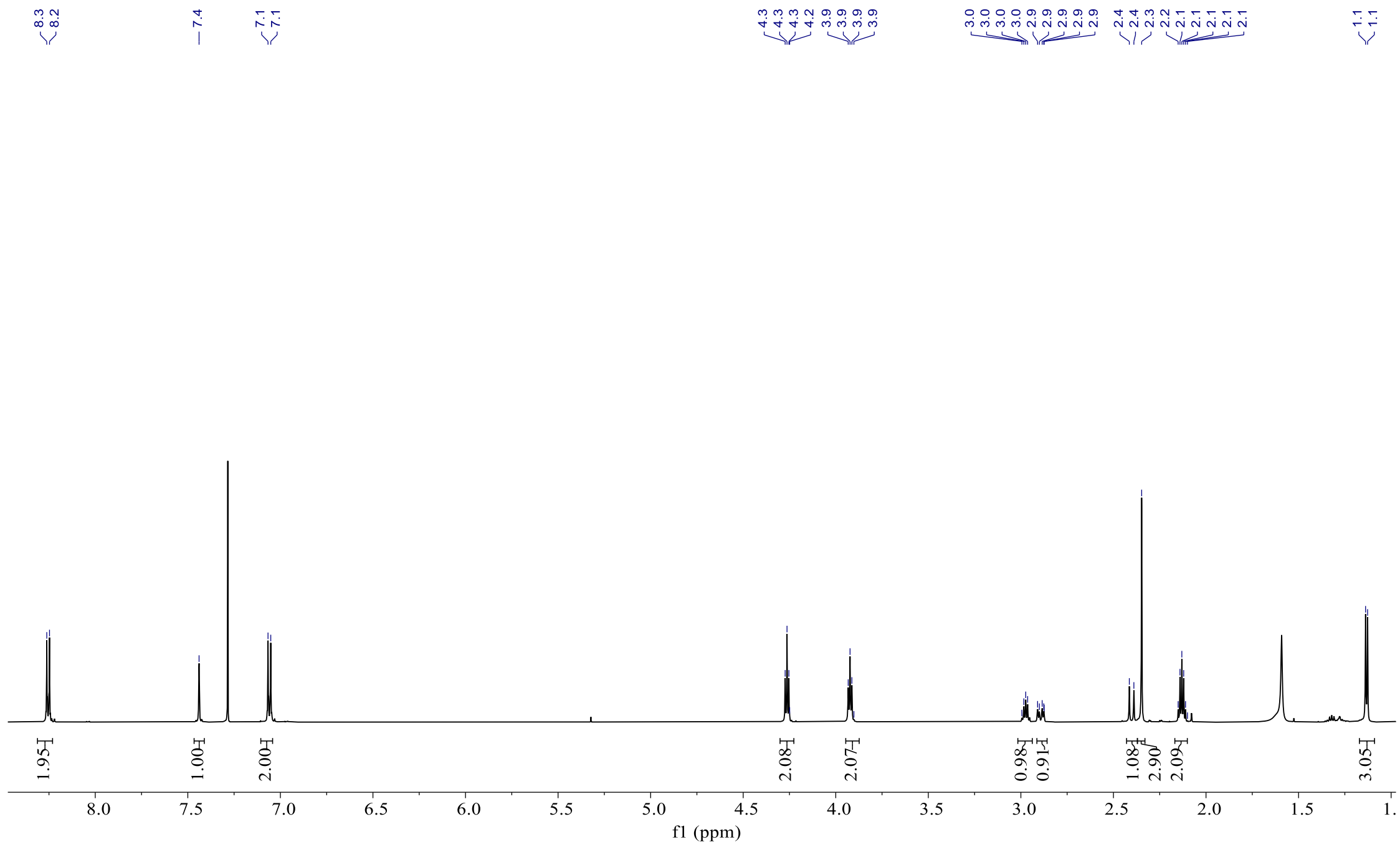

Supplementary Fig. 14 | NMR characterization. ^{1}H-NMR spectrum of compound **CN-L-C3OH** in $CDCl_3$ at 600 MHz.

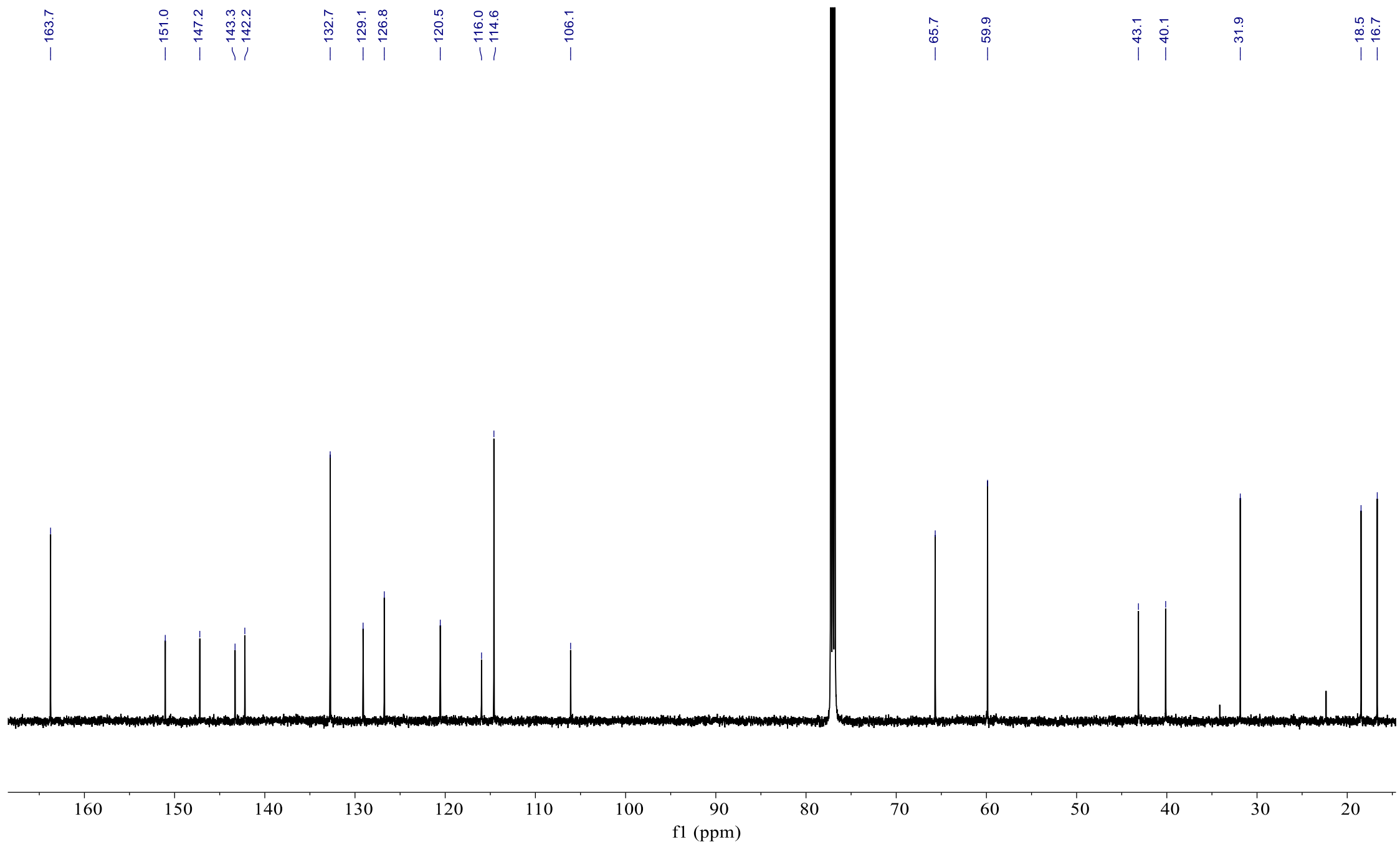

Supplementary Fig. 15 | NMR characterization. ^{13}C-NMR spectrum of compound **CN-L-C3OH** in $CDCl_3$ at 151 MHz.

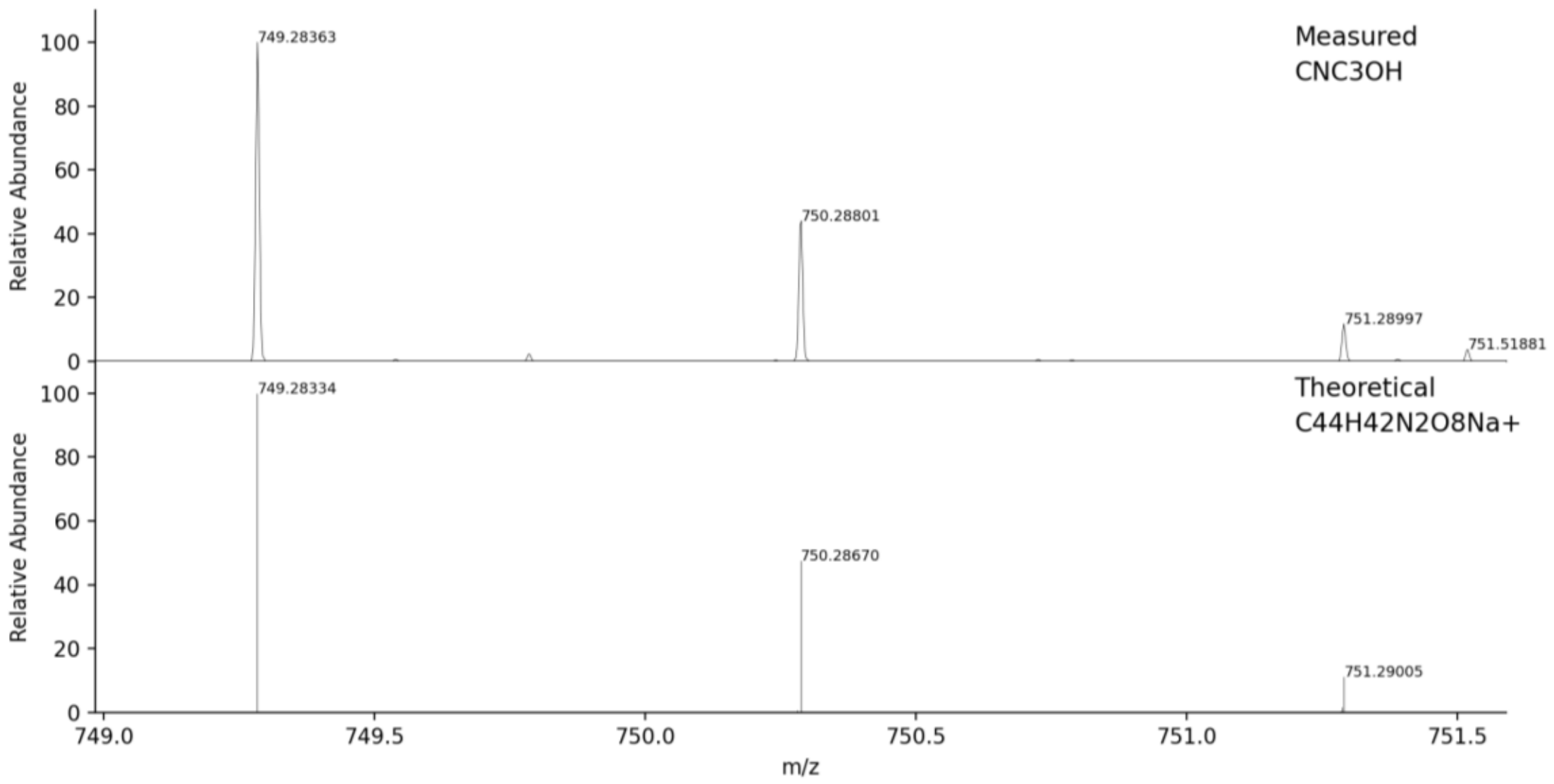

Supplementary Fig. 16 | HRMS characterization. HRMS spectrum of compound **CN-L-C3OH.**

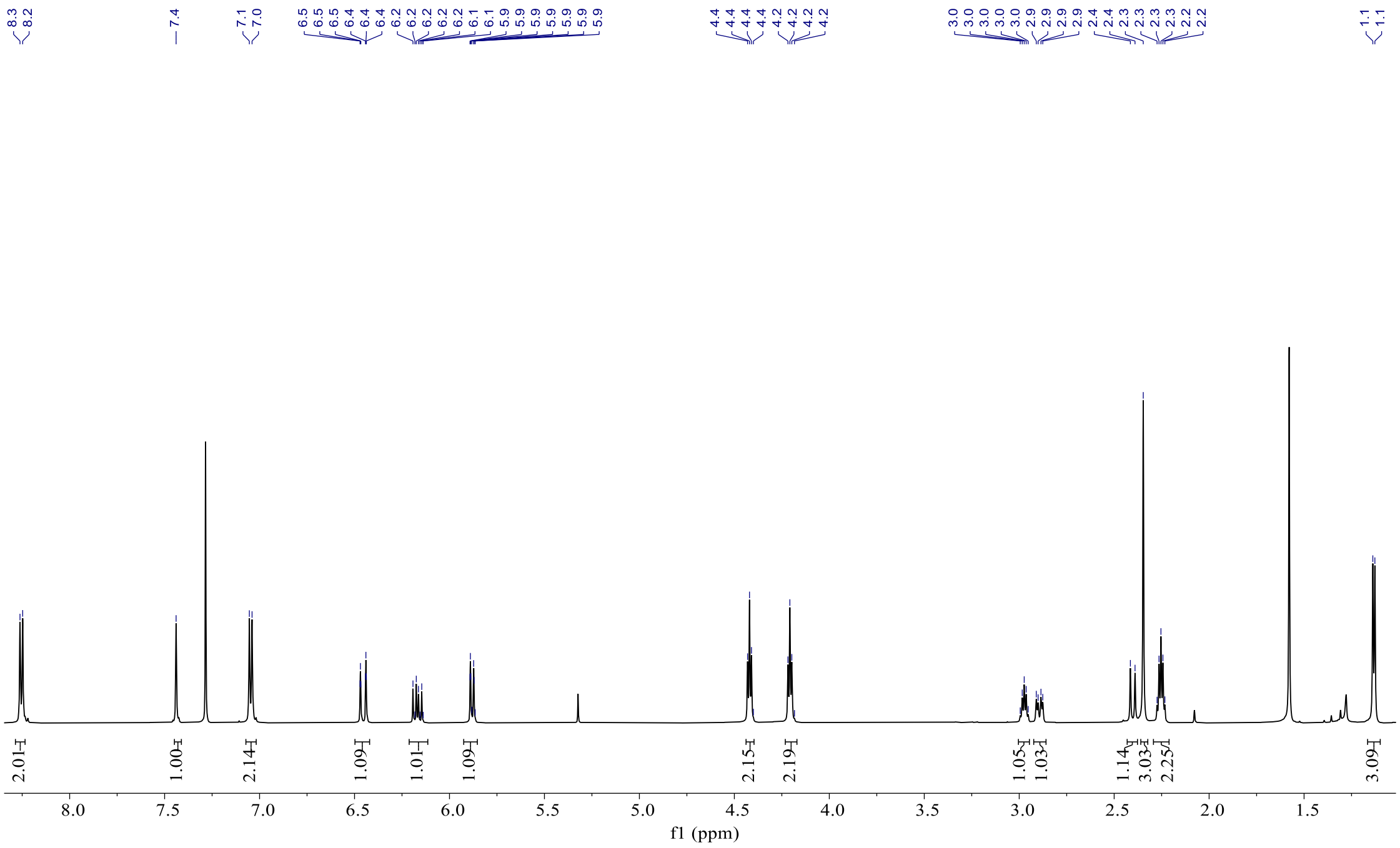

Supplementary Fig. 17 | NMR characterization. ^{1}H-NMR spectrum of compound **CN-L-C3** in $CDCl_3$ at 600 MHz.

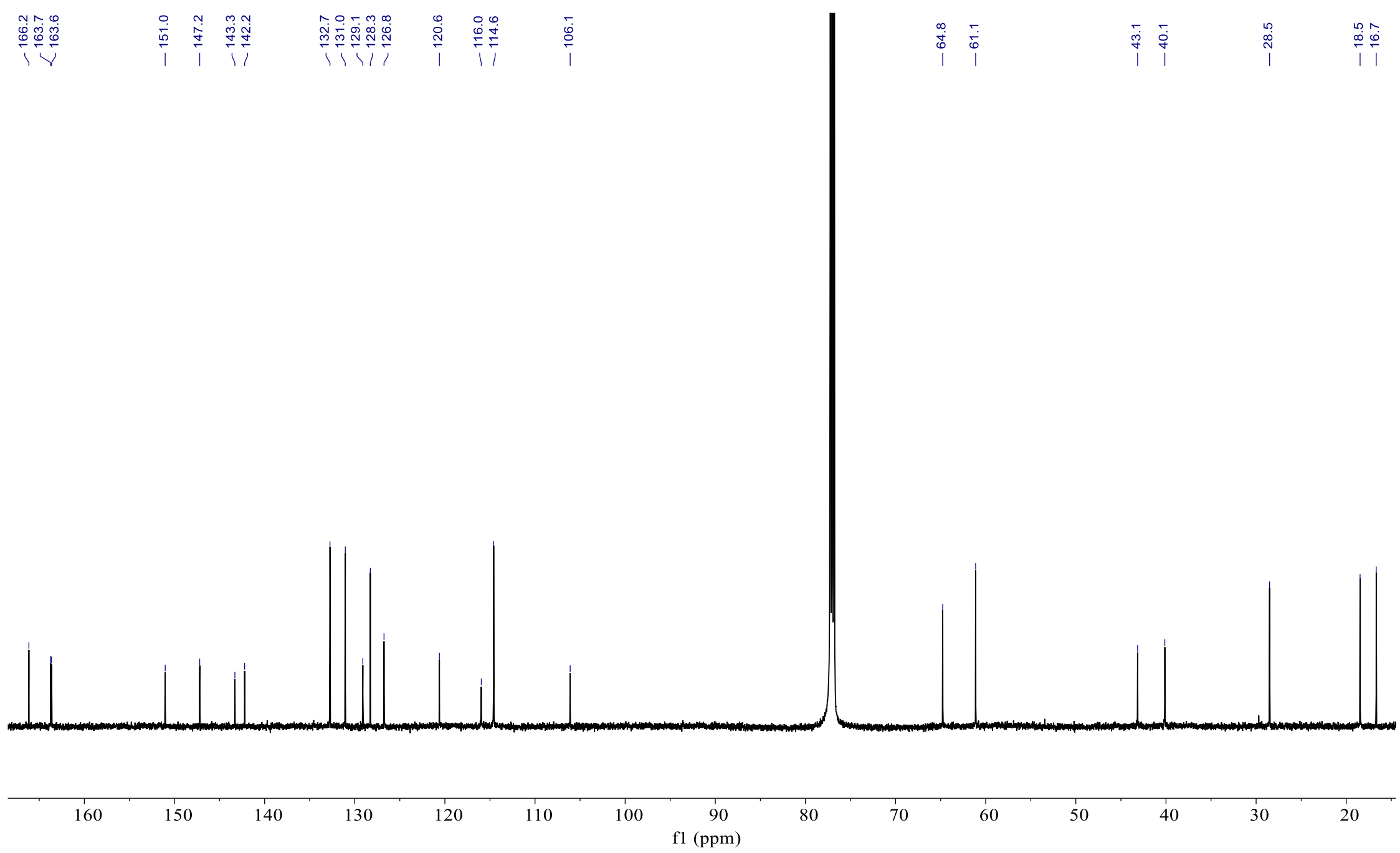

Supplementary Fig. 18 | NMR characterization. ^{13}C-NMR spectrum of compound **CN-L-C3** in $CDCl_3$ at 151 MHz.

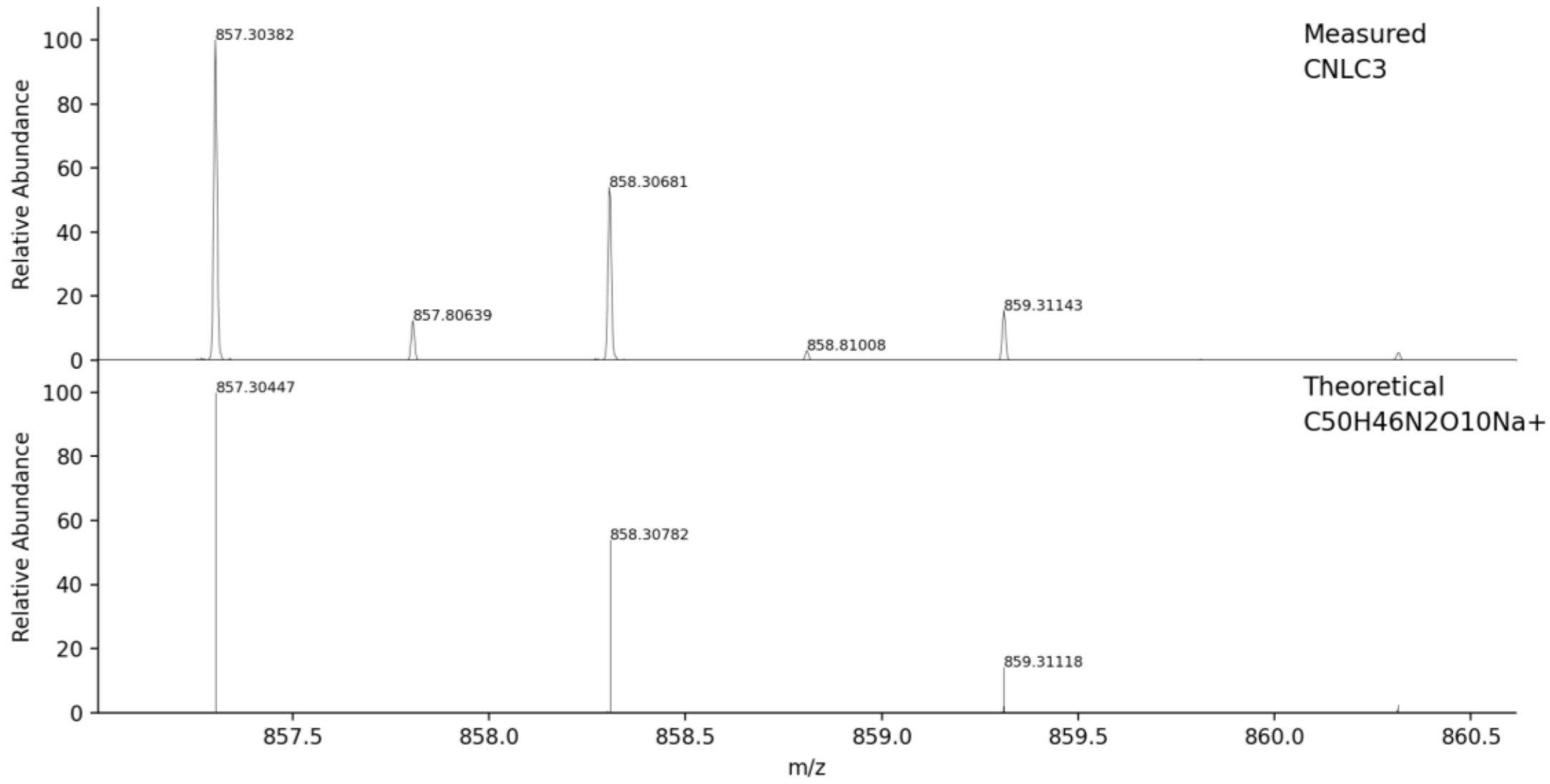

Supplementary Fig. 19 | HRMS characterization. HRMS spectrum of compound **CN-L-C3.**

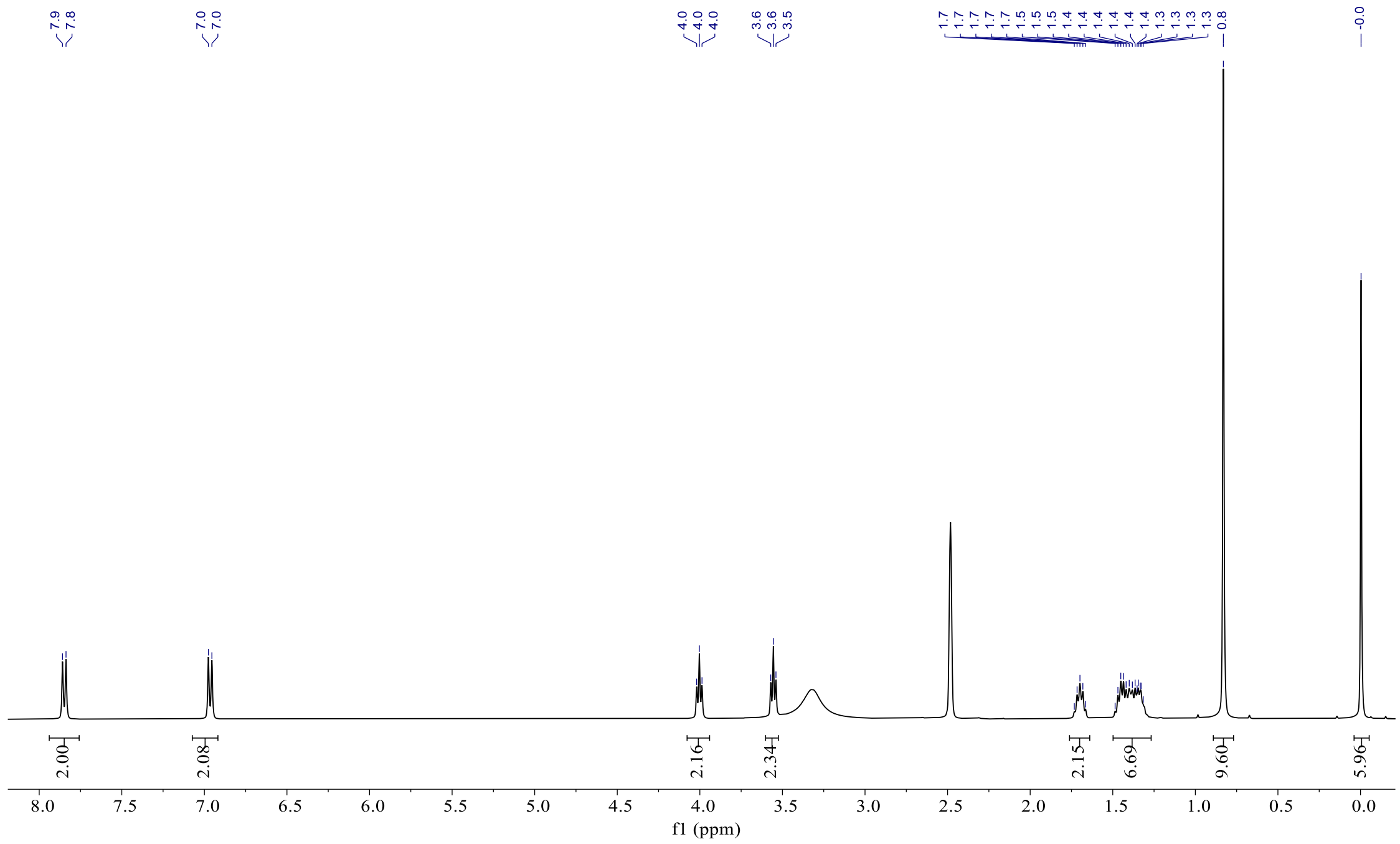

Supplementary Fig. 20 | NMR characterization. ^{1}H-NMR spectrum of compound **C6OTBS** in DMSO at 600 MHz.

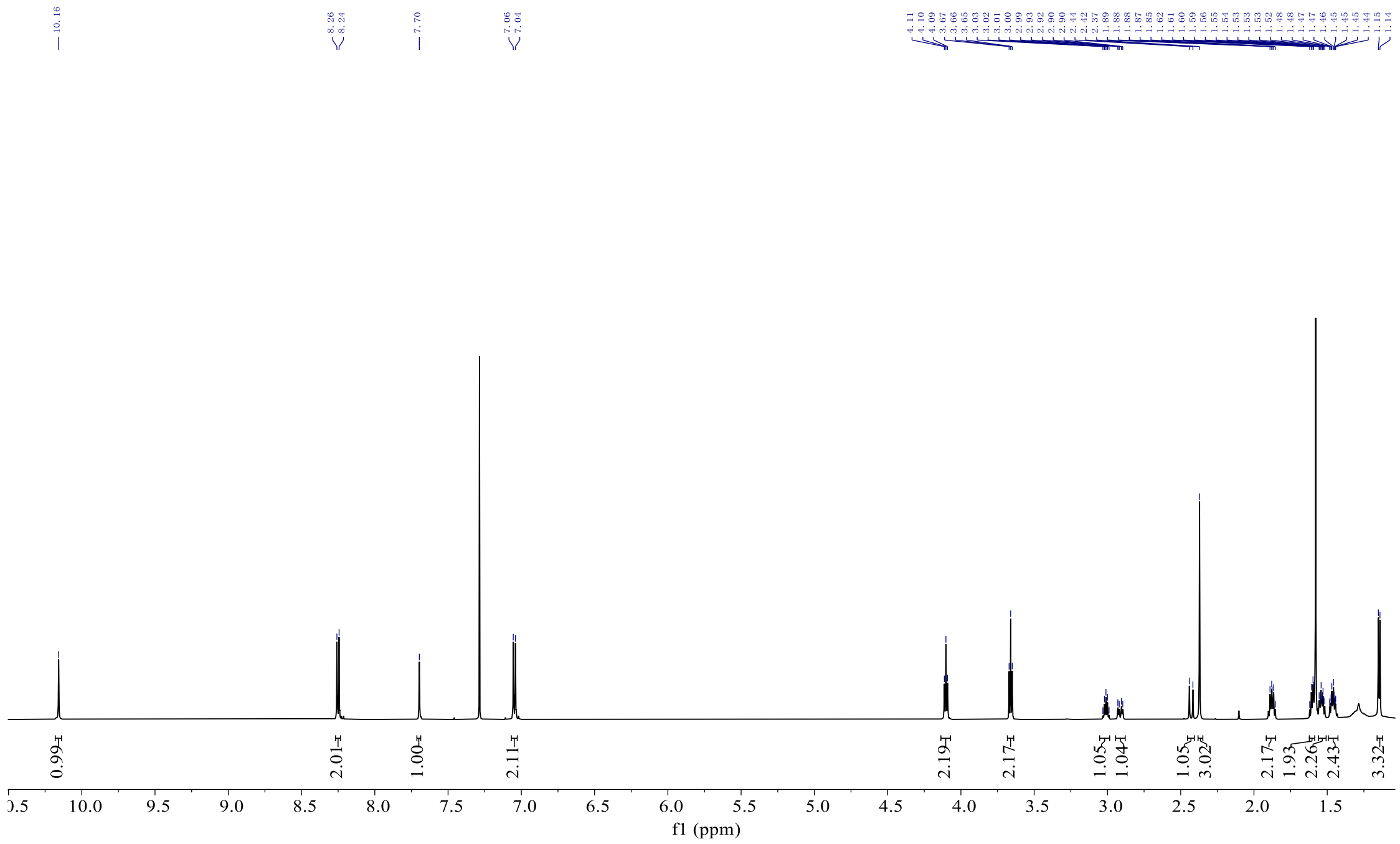

Supplementary Fig. 21 | NMR characterization. ^{1}H-NMR spectrum of compound **CHO-L-C6OTBS** in $CDCl_3$ at 600 MHz.

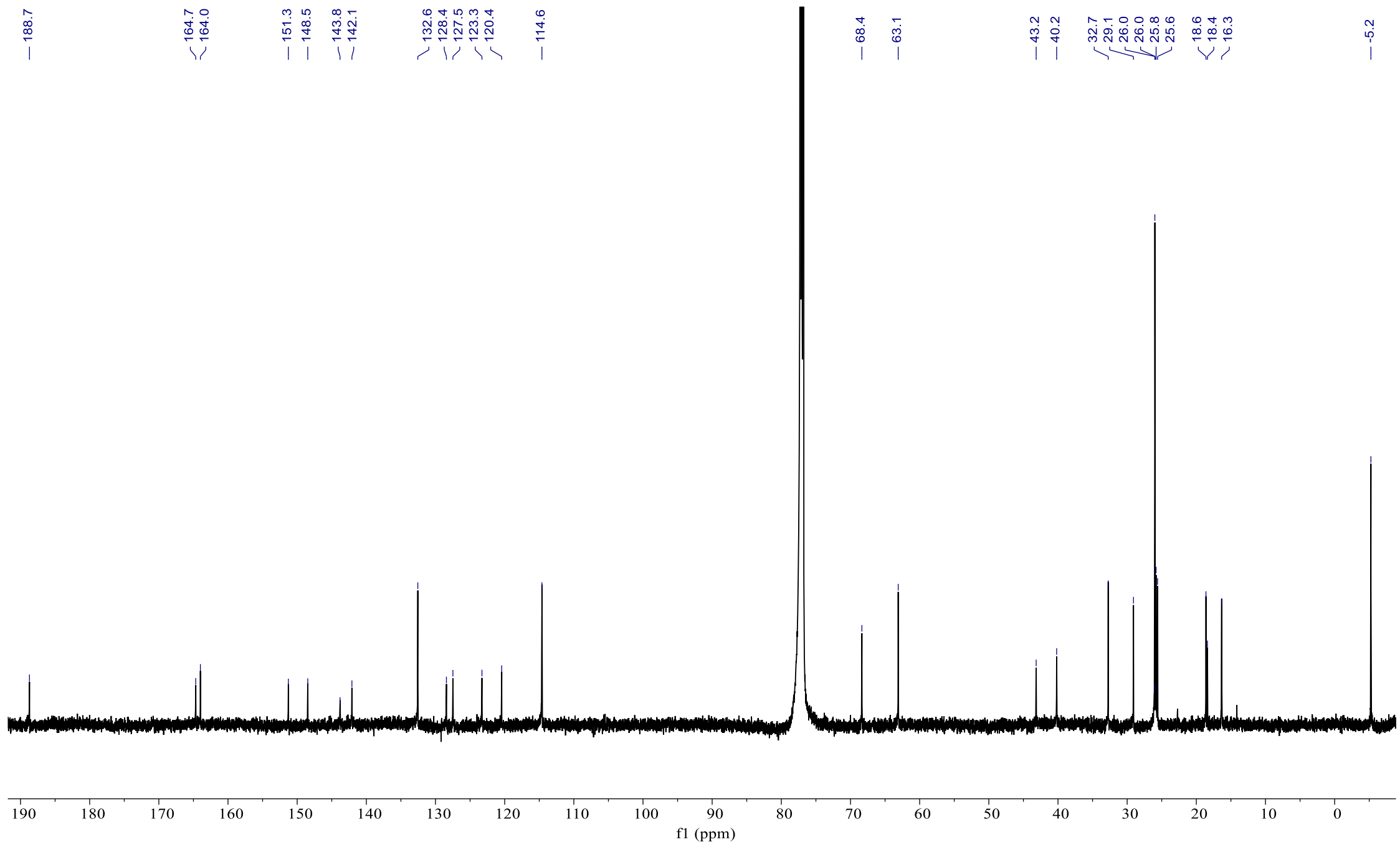

Supplementary Fig. 22 | NMR characterization. ^{13}C-NMR spectrum of compound **CHO-L-C6OTBS** in $CDCl_3$ at 151 MHz.

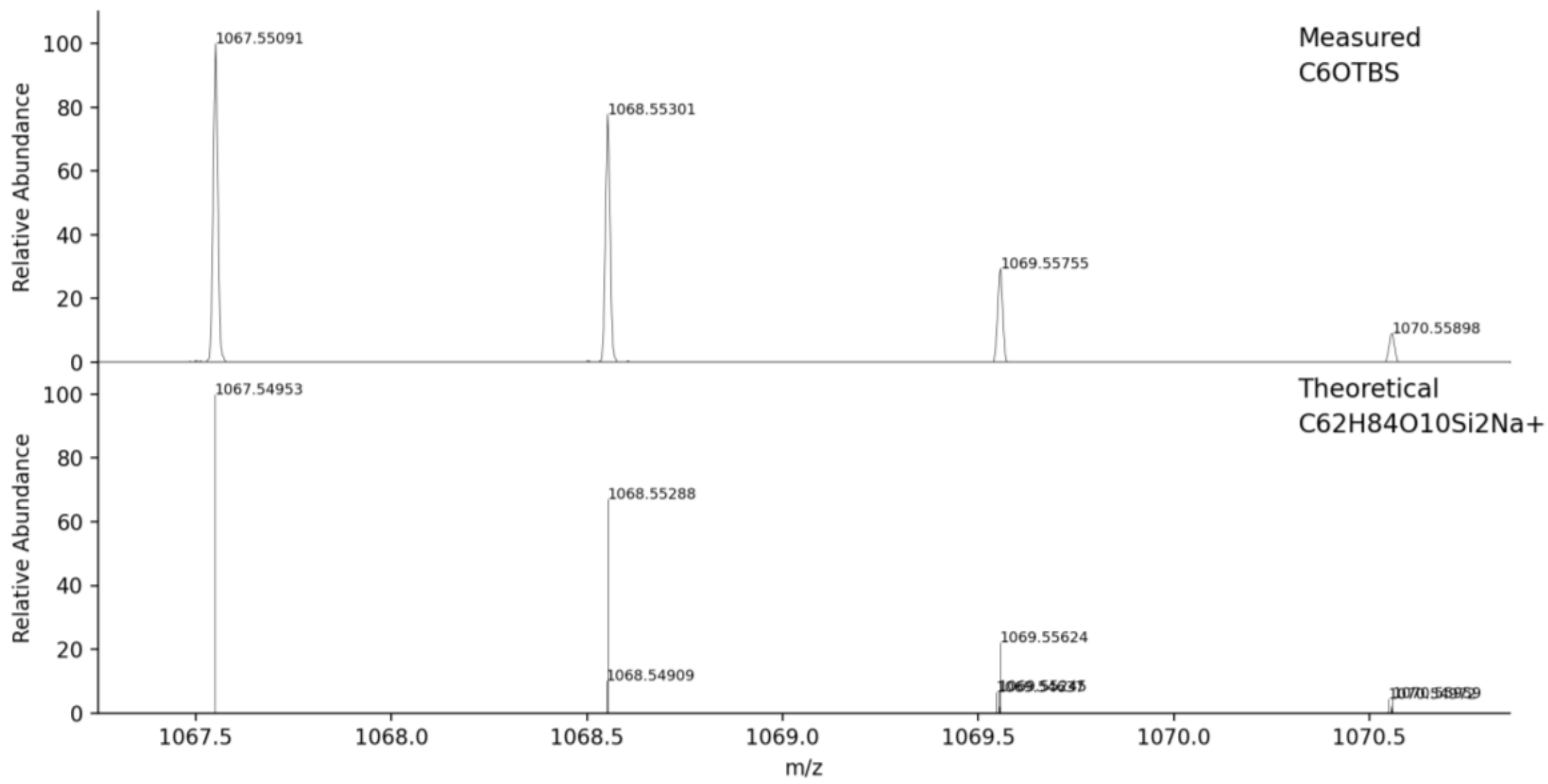

Supplementary Fig. 23 | HRMS characterization. HRMS spectrum of compound **CHO-L-C6OTBS.**

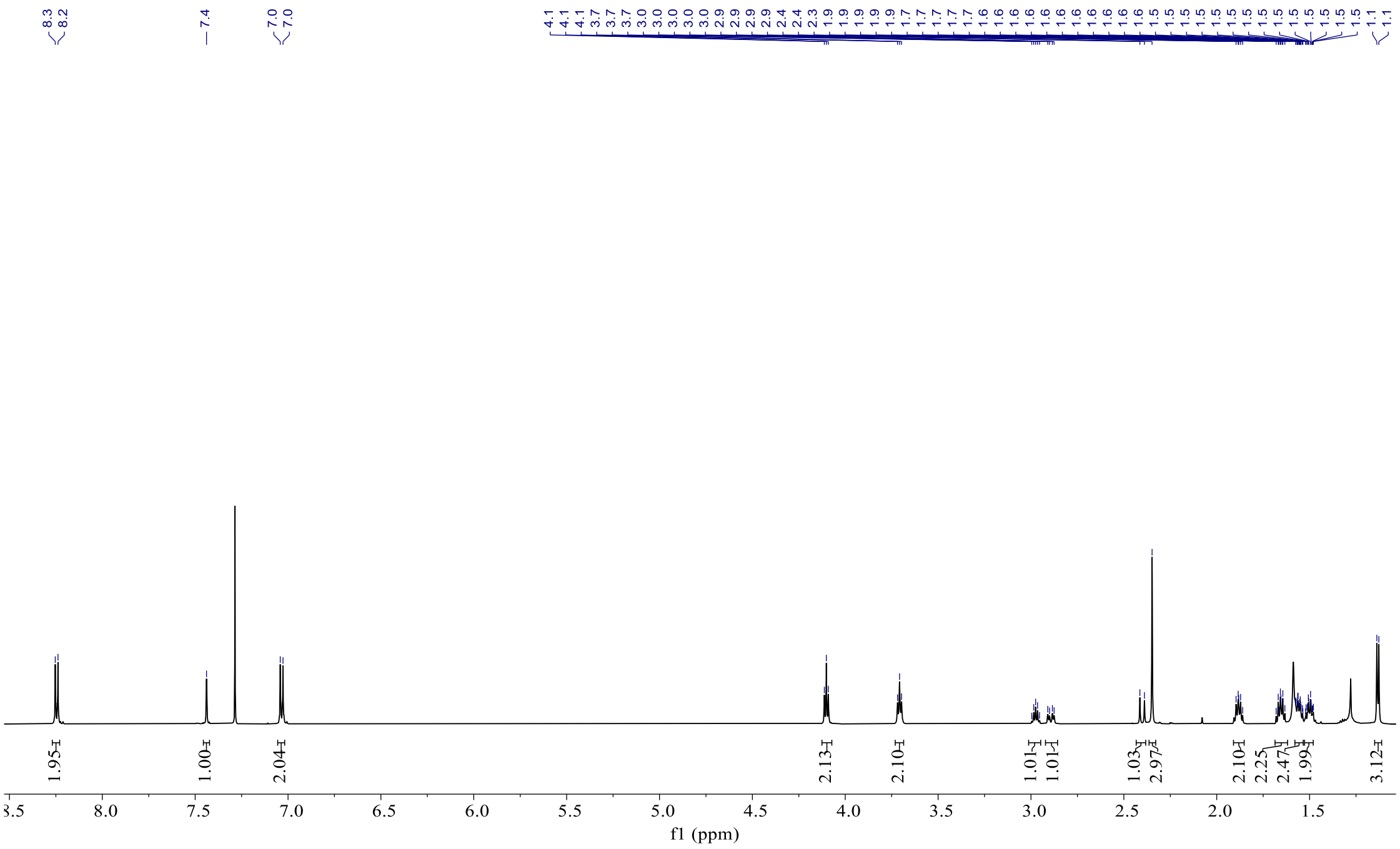

Supplementary Fig. 24 | NMR characterization. ^{1}H-NMR spectrum of compound **CN-L-C6OH** in $CDCl_3$ at 600 MHz.

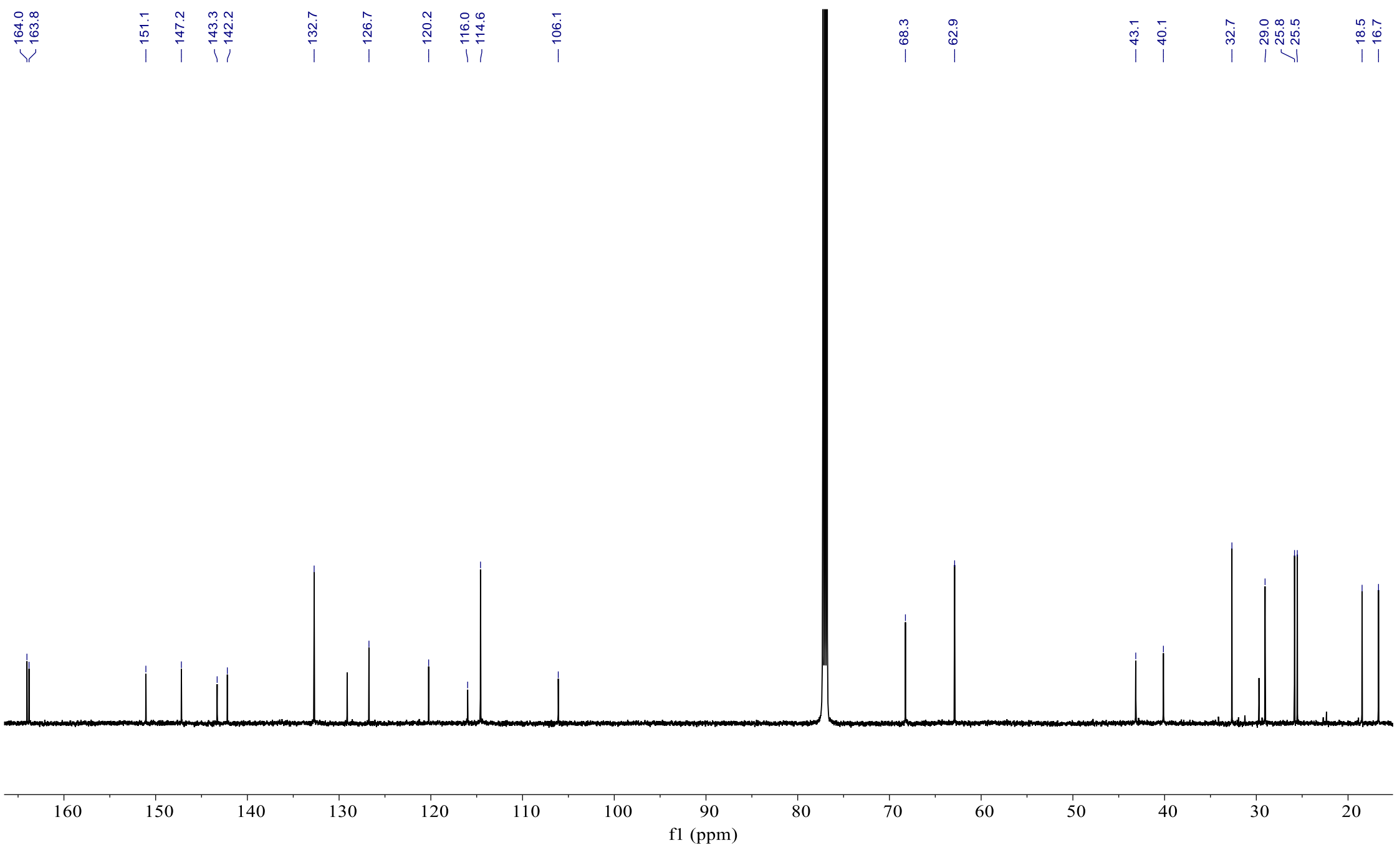

Supplementary Fig. 25 | NMR characterization. ^{13}C-NMR spectrum of compound **CN-L-C6OH** in $CDCl_3$ at 151 MHz.

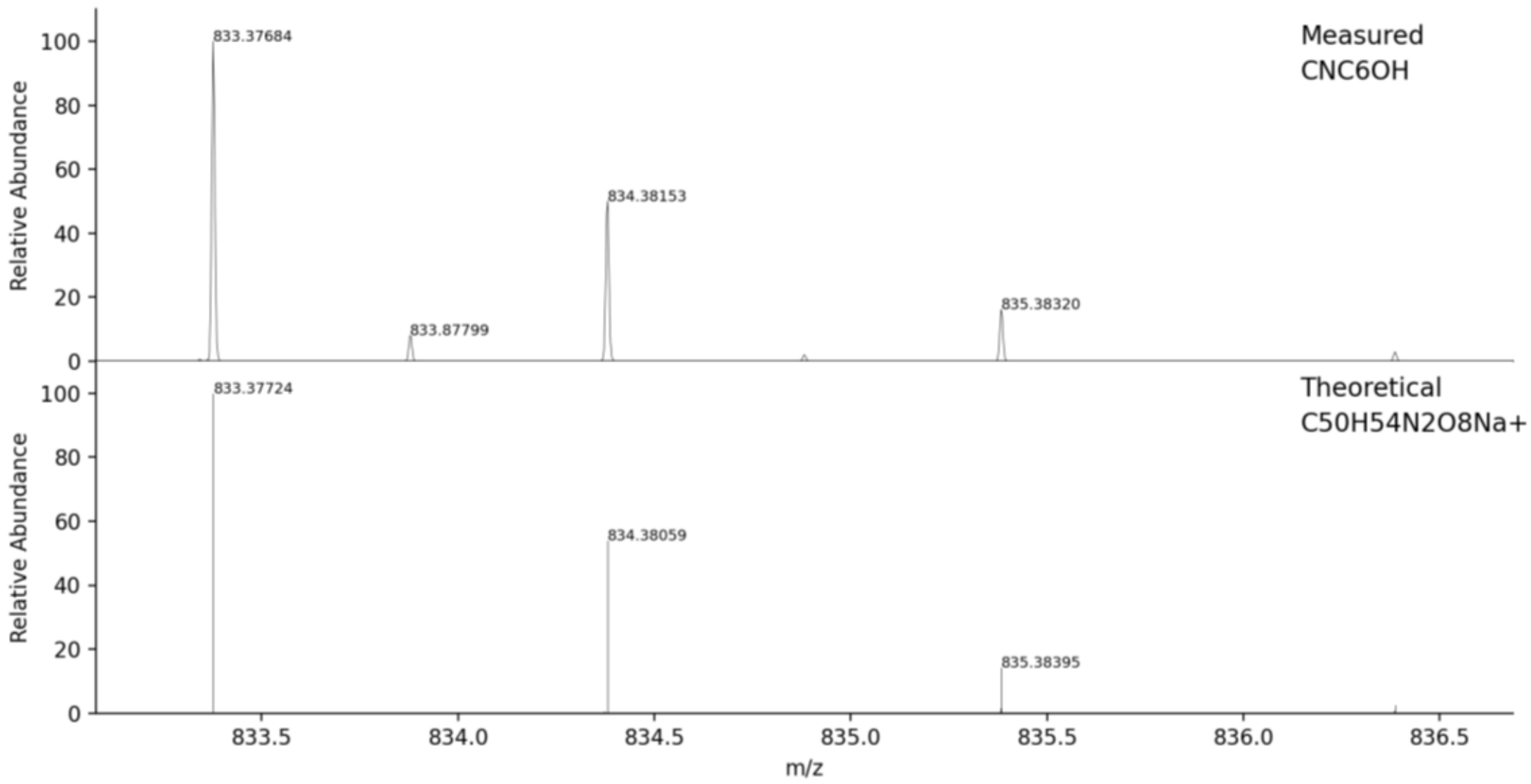

Supplementary Fig. 26 | HRMS characterization. HRMS spectrum of compound **CN-L-C6OH.**

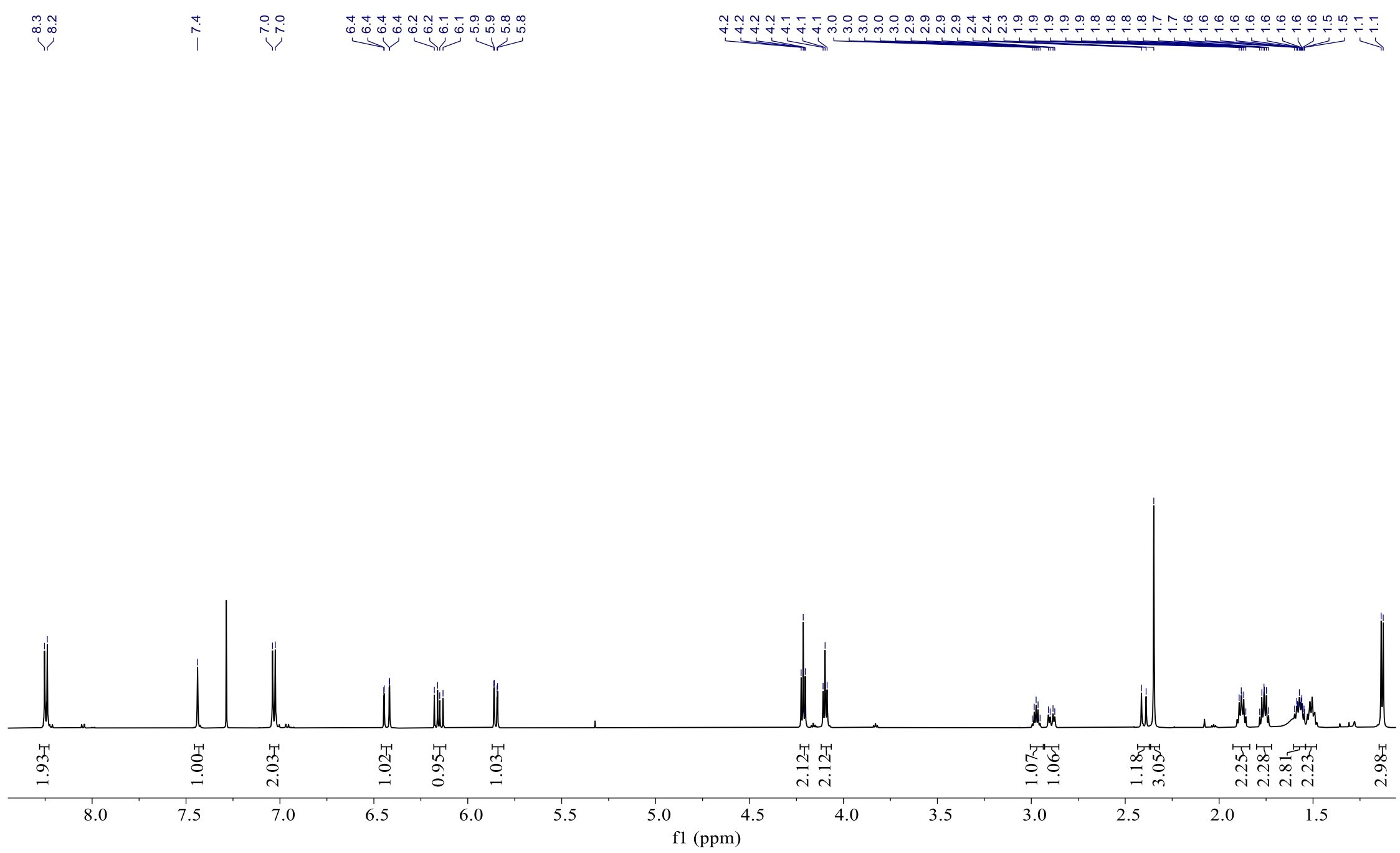

Supplementary Fig. 27 | NMR characterization. ^{1}H-NMR spectrum of compound **CN-L-C6** in $CDCl_3$ at 600 MHz.

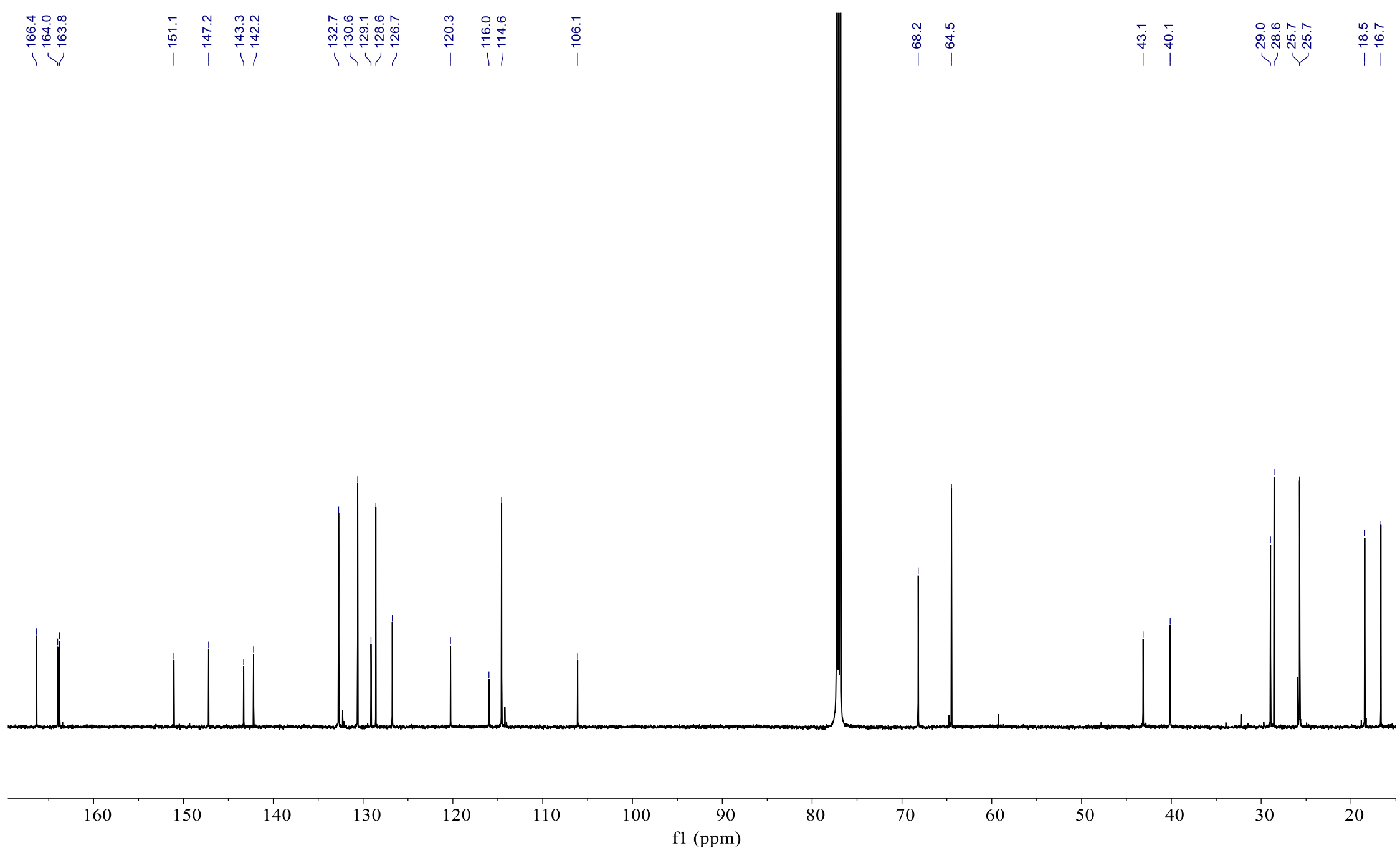

Supplementary Fig. 28 | NMR characterization. ^{13}C-NMR spectrum of compound **CN-L-C6** in $CDCl_3$ at 151 MHz.

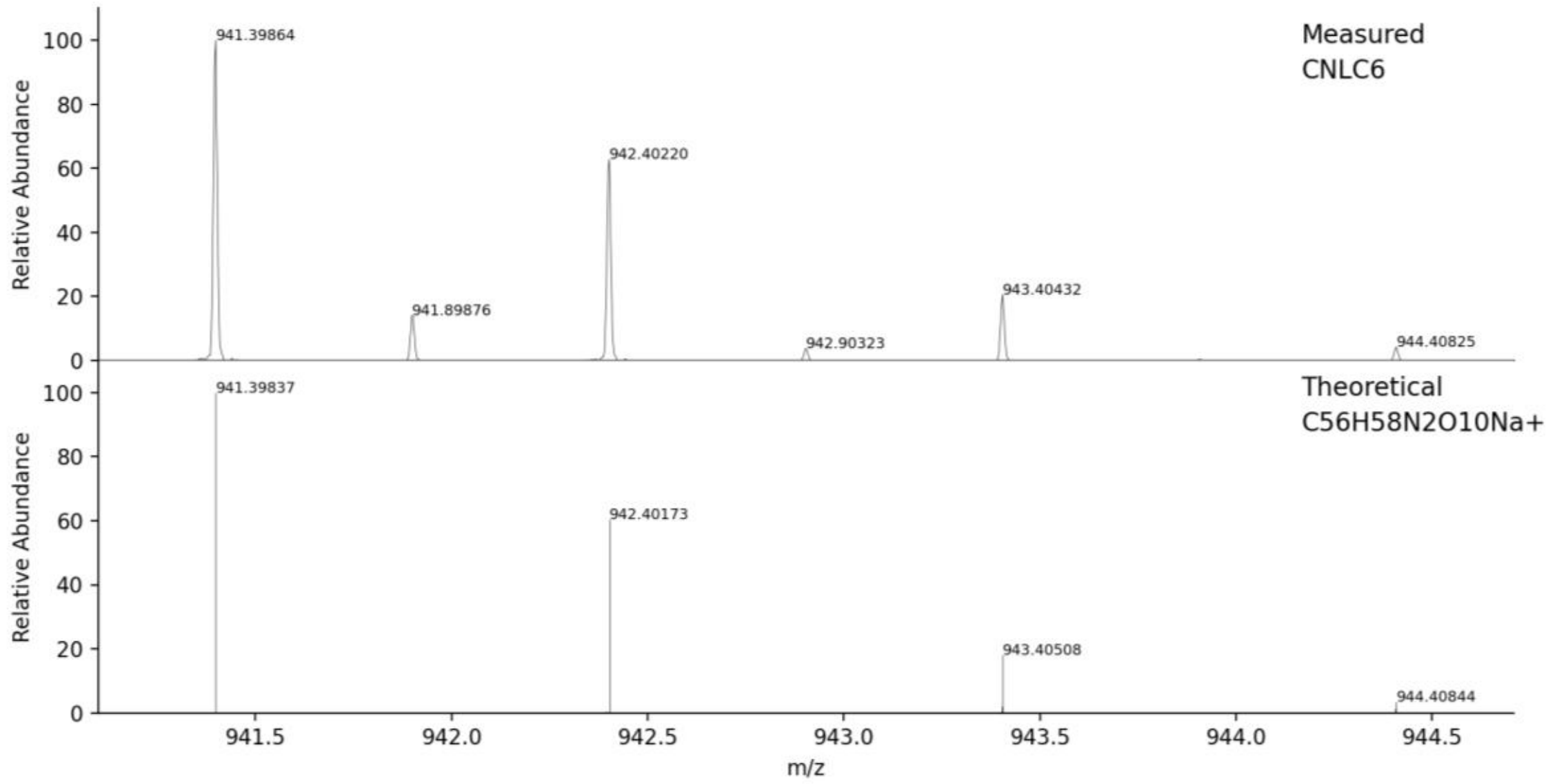

Supplementary Fig. 29 | HRMS characterization. HRMS spectrum of compound **CN-L-C6.**

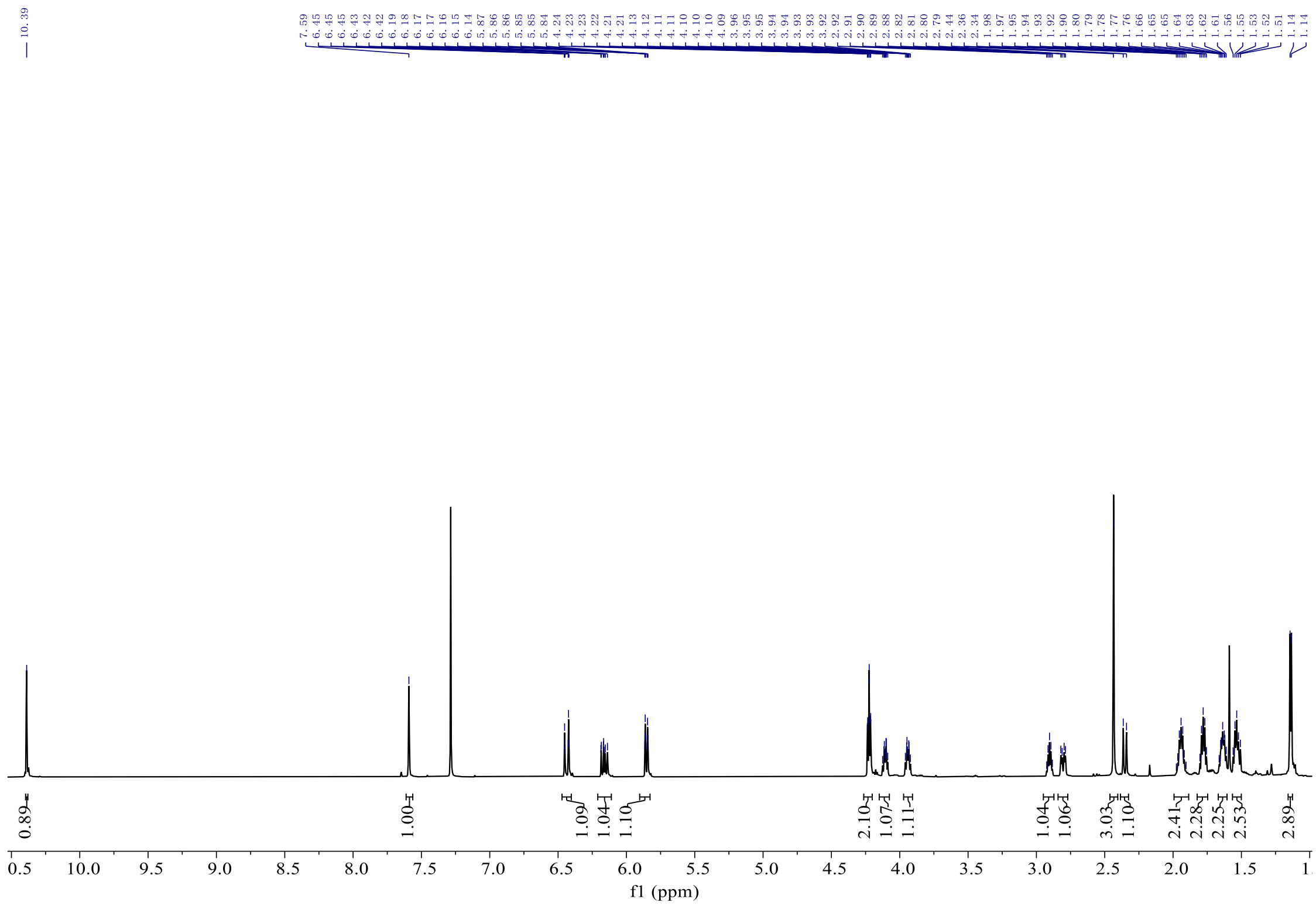

Supplementary Fig. 30 | NMR characterization. ^{1}H-NMR spectrum of compound **CHO-S** in $CDCl_3$ at 600 MHz.

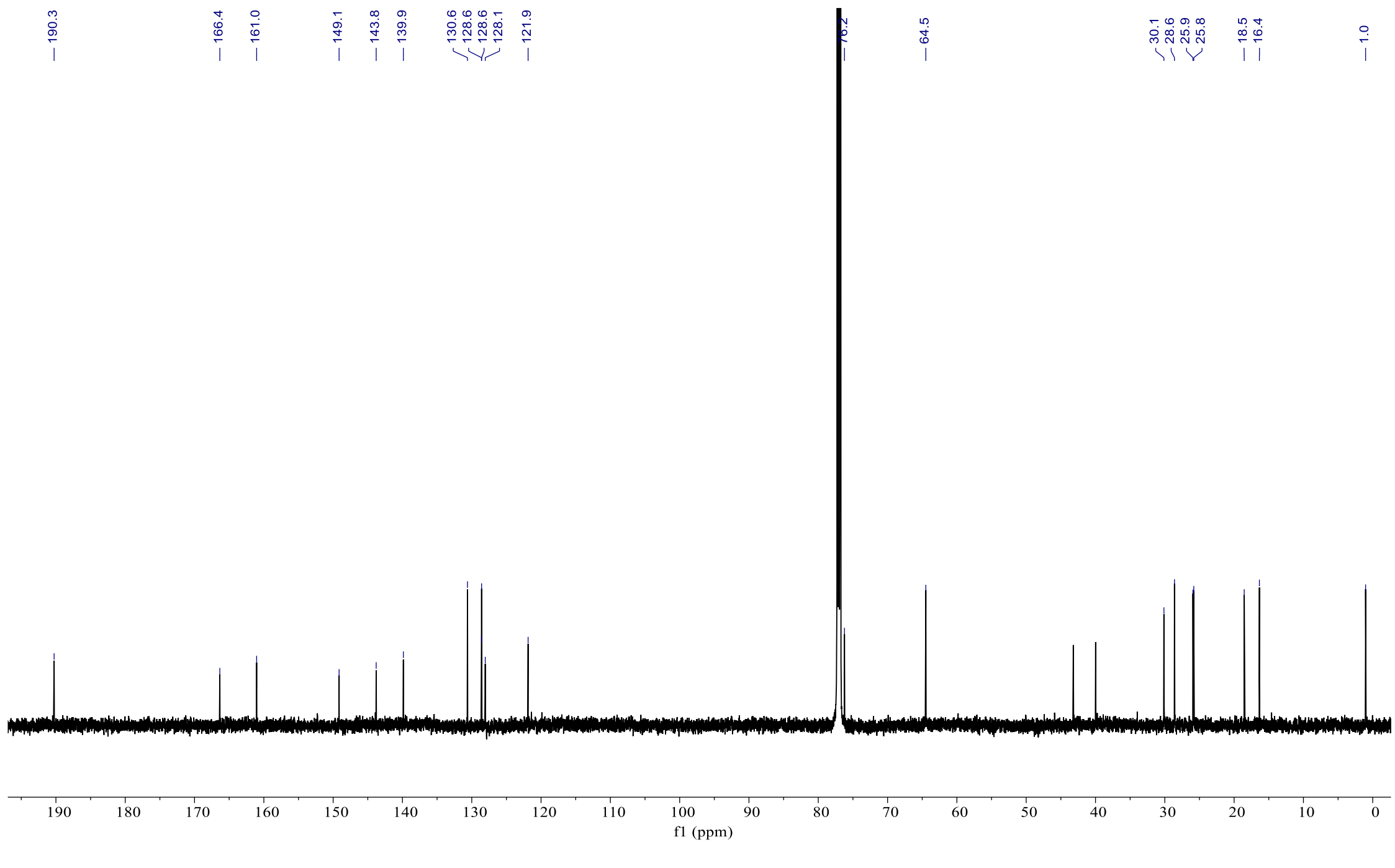

Supplementary Fig. 31 | NMR characterization. ^{13}C-NMR spectrum of compound **CHO-S** in $CDCl_3$ at 151 MHz.

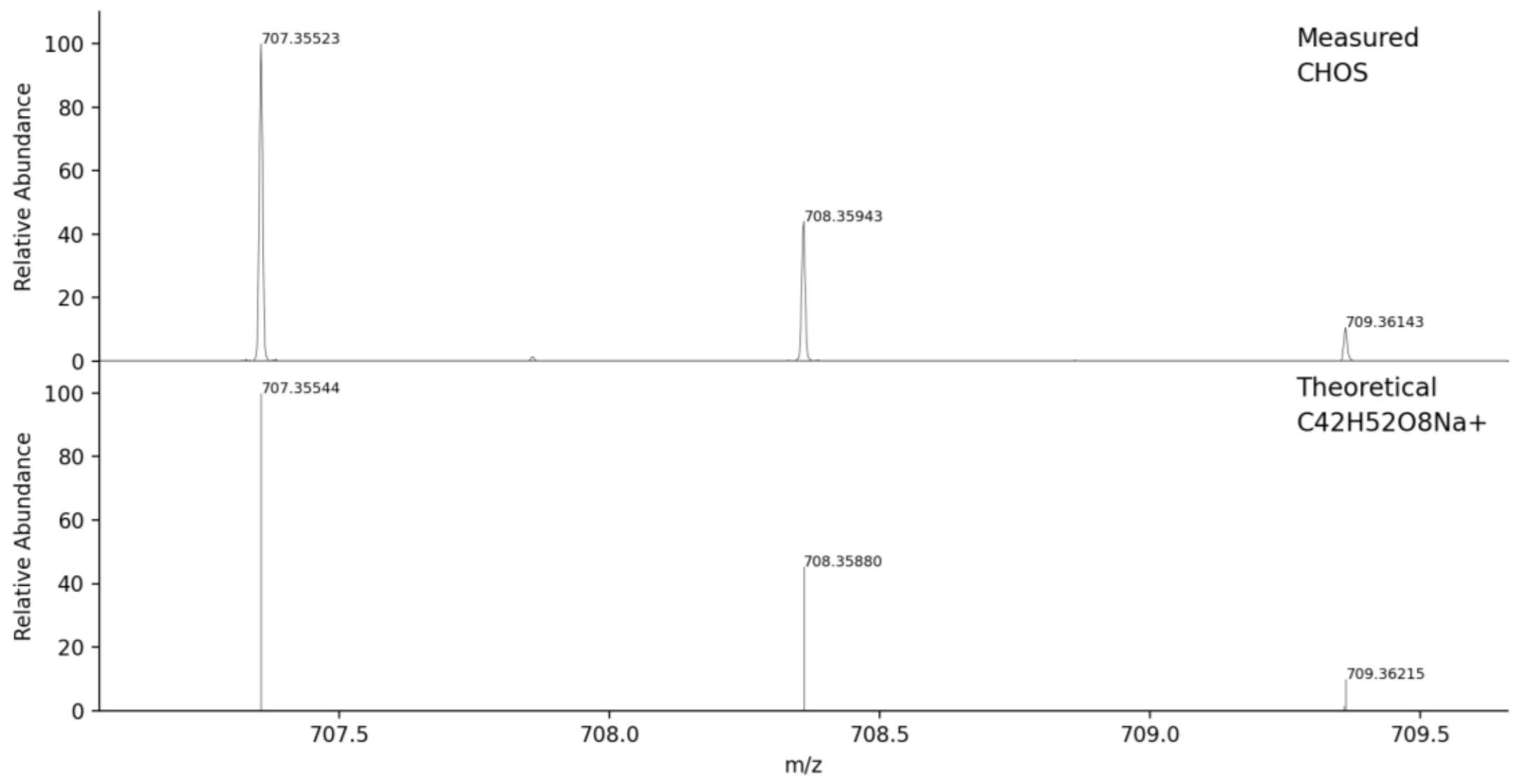

Supplementary Fig. 32 | HRMS characterization. HRMS spectrum of compound **CHO-S.**

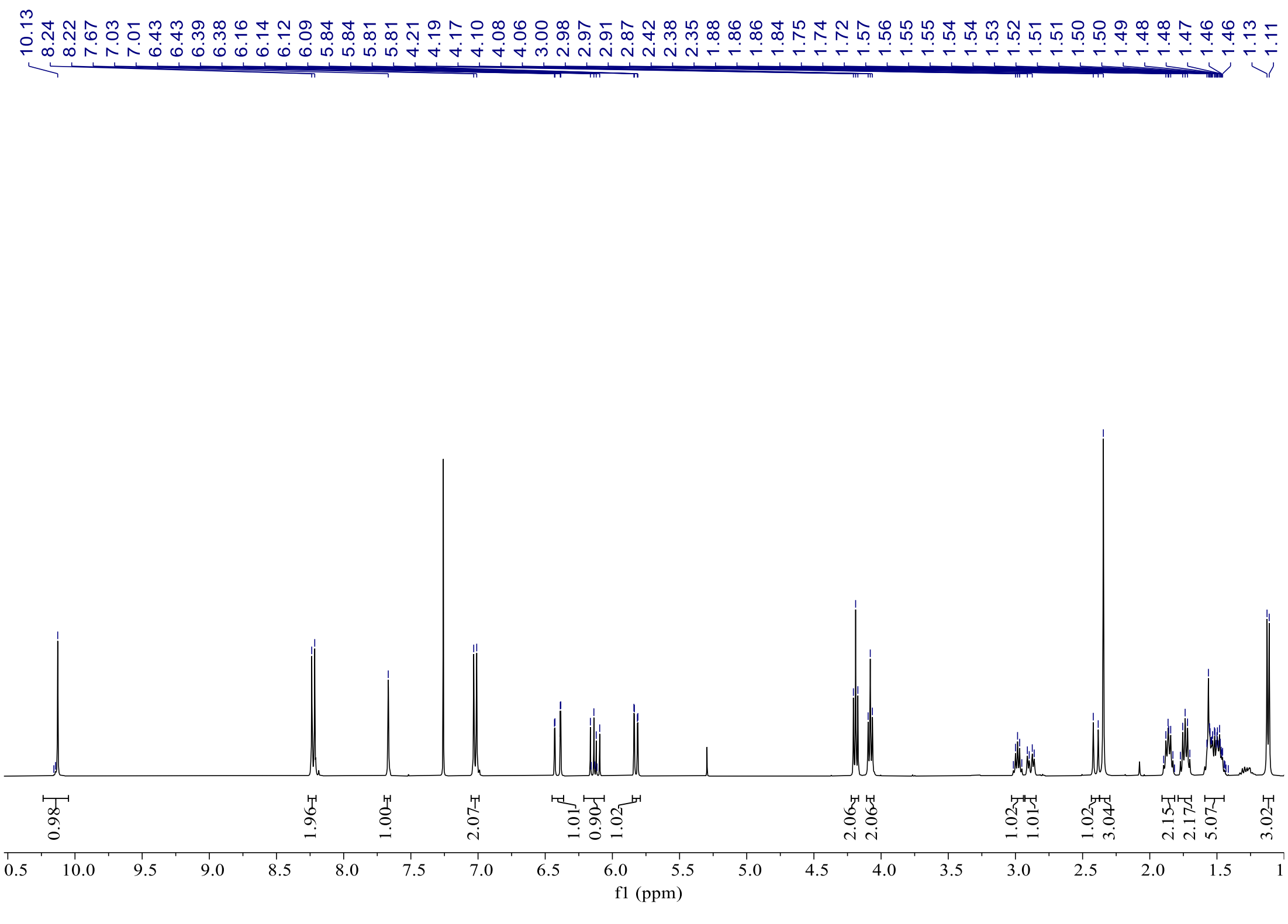

Supplementary Fig. 33 | NMR characterization. ^{1}H-NMR spectrum of compound **CHO-L** in $CDCl_3$ at 400 MHz.

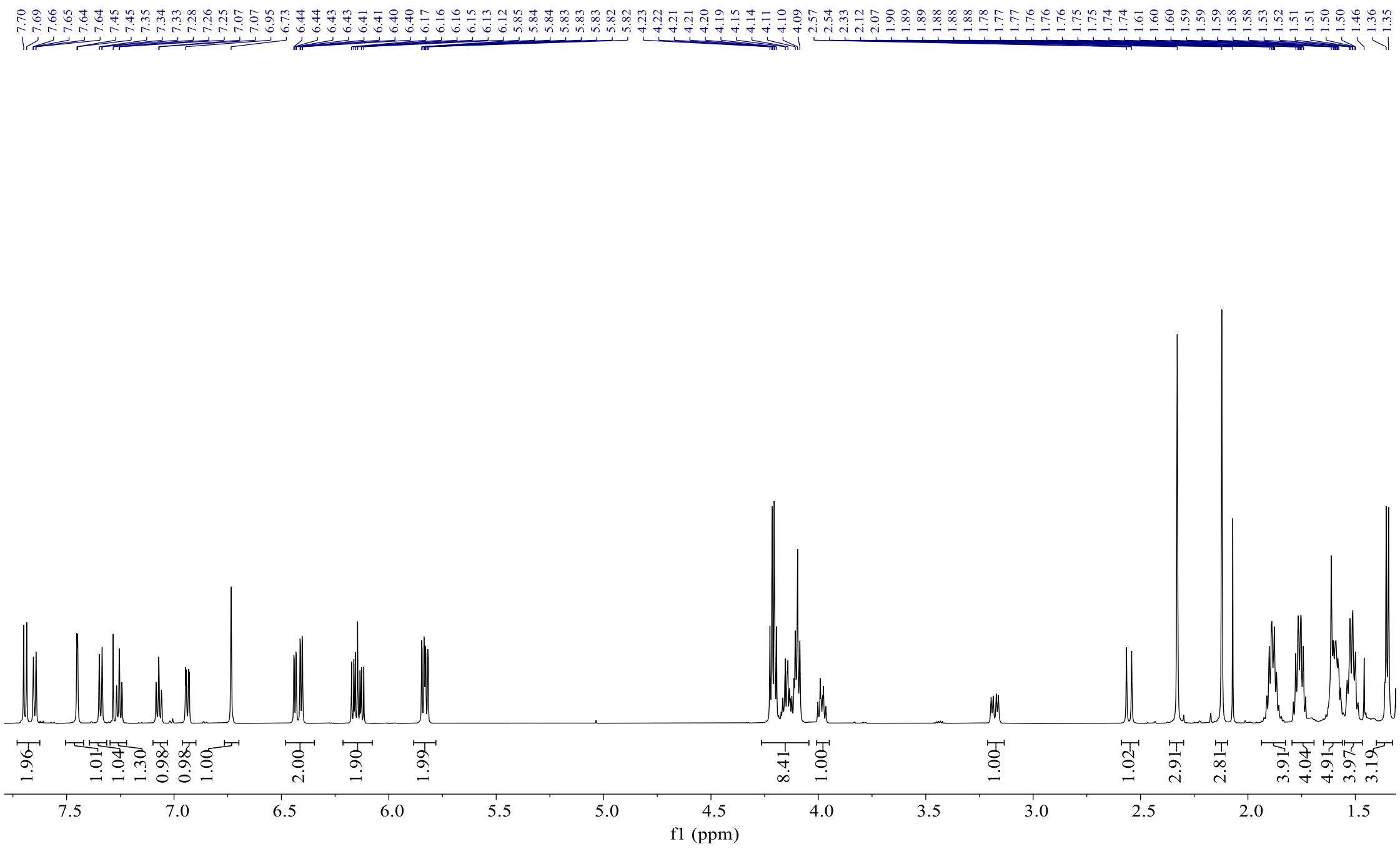

Supplementary Fig. 34 | NMR characterization. ^{1}H-NMR spectrum of compound **MSG** in $CDCl_3$ at 600 MHz.

Supplementary Discussion

4 ^{1}H-NMR studies of motors CN-L-C3, CN-L-C6 and CHO-S in solution

The protons Ha, Hb, and Hc of motor **CN-L-C3** correspond to signals at 2.94 ppm, 2.90 ppm, and 2.41 ppm, respectively, as indicated in Supplementary Fig. 35b (blue line), which is attributed to different chemical environments resulting from the conformation of the five-membered ring. Upon UV irradiation (λ = 340 nm) at room temperature, the sample reached the photostationary state (PSS), accompanied by significant spectral changes that indicate the formation of a new isomer Z_M (green line). The new peaks at 3.72 ppm, 3.30 ppm, and 2.87 ppm were attributed to the Ha, Hb, and Hc protons of Z_M, respectively. Quantification of the isomer conversion ratio by integration yielded a ratio of Z_M to E_S of 97:3. The samples were subsequently stored in a dark environment for 3 weeks, and the characteristic peaks were further shifted to 3.52 ppm, 3.33 ppm, and 2.62 ppm, respectively (purple line). This indicates that Z_M was converted to Z_S by THI process. Spectral analysis showed that the ratio of Z_S to Z_M was more than 99:1, confirming nearly complete thermal conversion under these conditions.

For the motor **CN-L-C6**, partial ^{1}H-NMR spectra corresponding to different states are shown in Supplementary Fig. 35c. The prominent characteristic absorptions of the first-generation motor molecule are the protons Ha, Hb, and Hc, which appear at 2.94 ppm, 2.90 ppm, and 2.40 ppm, respectively (Supplementary Fig. 35c, blue line). The sample was then irradiated with UV light (λ = 340 nm) at room temperature until it reached the photostationary state (PSS). The new absorptions at 3.72 ppm, 3.30 ppm, and 2.87 ppm correspond to Ha, Hb, and Hc of the Z_M-**CN-L-C6** state, respectively (green line). Integration analysis of the spectra yielded a ratio of Z_M to E_S of 95:5. The sample was then stored in the dark for three weeks, and the characteristic peaks shifted further to 3.52 ppm, 3.33 ppm, and 2.63 ppm, indicating conversion of Z_M to Z_S by THI process. Spectral analysis confirmed the ratio of Z_S to Z_M exceeded 99:1, demonstrating nearly complete thermal

conversion under these conditions. Similarly, the motor **CN-L-C3** underwent $E_S \rightarrow Z_M$ photoisomerization under 340 nm irradiation, reaching a Z_M : E_S ratio of 97:3 at PSS. After storage in the dark for 3 weeks, the Z_M isomer almost completely converted to Z_S-isomer (purple line).

The protons Ha, Hb, and Hc of aldehyde motor **CHO-S** correspond to signals at 2.94 ppm, 2.82 ppm, and 2.38 ppm, respectively, as indicated in Supplementary Fig. 35d (blue line), which is attributed to different chemical environments resulting from the conformation of the five-membered ring. The sample was then irradiated with UV light (λ = 365 nm) at room temperature until it reached the photostationary state (PSS), resulting in a significant change in the spectra, indicating the formation of a new isomer Z_M (green line). The new peaks at 3.67 ppm, 3.31 ppm, and 2.86 ppm were attributed to the Ha, Hb, and Hc protons of Z_M, respectively. Quantification of the isomer conversion ratio by integration yielded a ratio of Z_M to E_S of 97:3. The samples were subsequently stored in a dark environment for 5 days, and the characteristic peaks were further shifted to 3.48 ppm, 3.28 ppm, and 2.59 ppm, respectively (purple line). This indicates that Z_M was converted to Z_S by THI process. Spectral analysis showed that the ratio of Z_S to Z_M was more than 99:1, confirming nearly complete thermal conversion under these conditions.

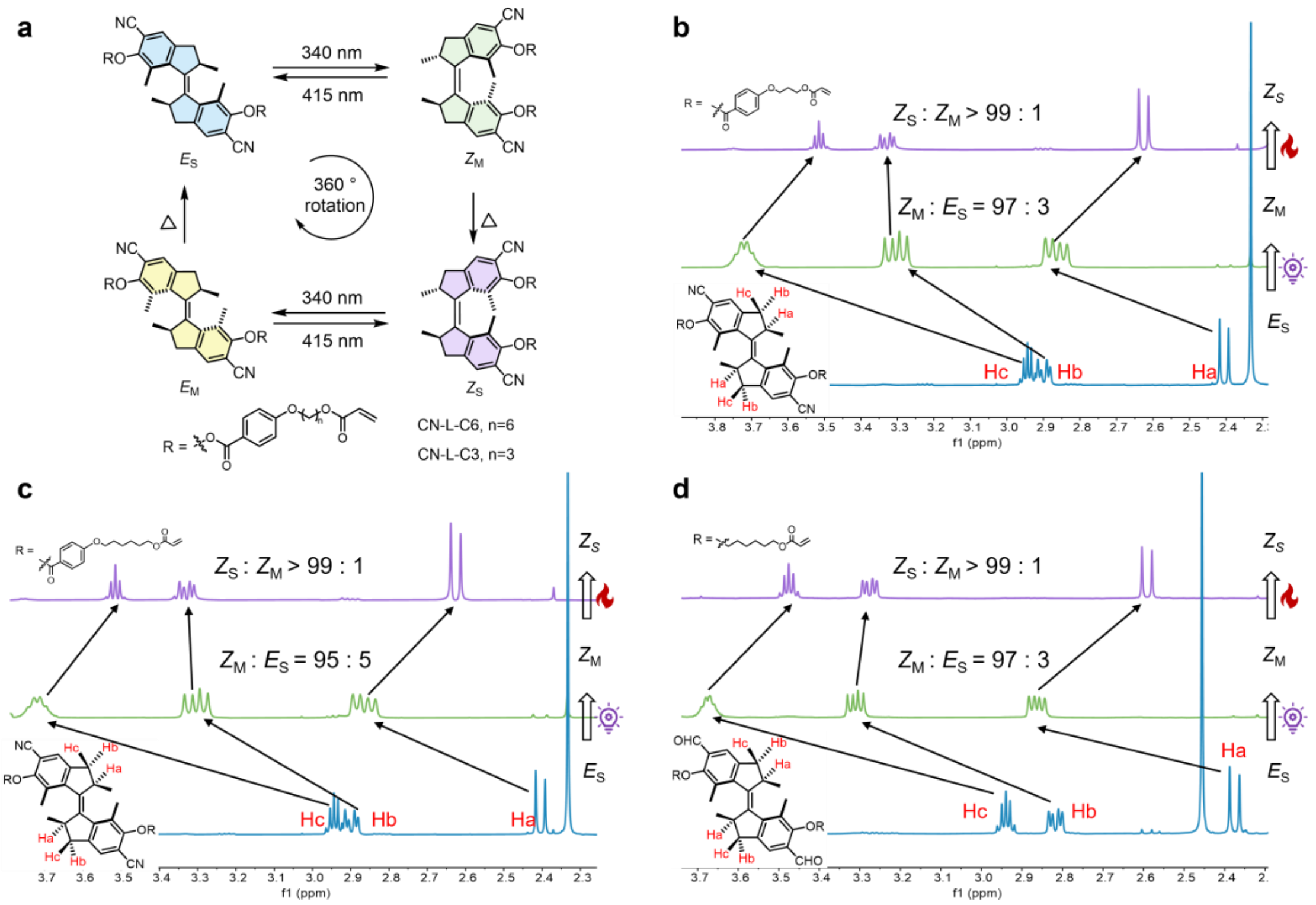

Supplementary Fig. 35 | NMR characterization of functionalized motors rotation in solution. a The unidirectional rotary cycle of cyano motors **CN-L-C3** and **CN-L-C6**. (**b-d**) Partial ^{1}H-NMR of (**b**) **CN-L-C3**, (**c**) **CN-L-C6** and (**d**) **CHO-S** in different states (600 MHz, CD_2Cl_2, 25 °C). The initial E_S state (red line) was converted to Z_M (green line) exposure to 340 nm light for 4 h for cyano motors **CN-L-C3** and **CN-L-C6**, and irradiation with 365 nm for 20 min for aldehyde motor **CHO-S**. The THI to Z_S (blue line) was performed by keeping the sample at r.t. in the dark environment for **CN-L-C3** and **CN-L-C6** for 3 weeks, and **CHO-S** for 5 d.

5 UV-vis studies of motor in solution

5.1 Photochemical isomerization processes

The rotational behavior was further verified by UV-vis spectroscopy. Under 340 nm irradiation, the initial absorption bands of **CN-L-C6** at 329 nm and 343 nm gradually decreased, while a new absorption band centered at 380 nm formed, corresponding to the photochemical formation of the Z_M isomer. This process was reversible under blue light (λ = 415 nm) irradiation (Supplementary Fig. 36a). A similar study of **CN-L-C3**was conducted and exhibited analogous spectral changes (Supplementary Fig. 36b).

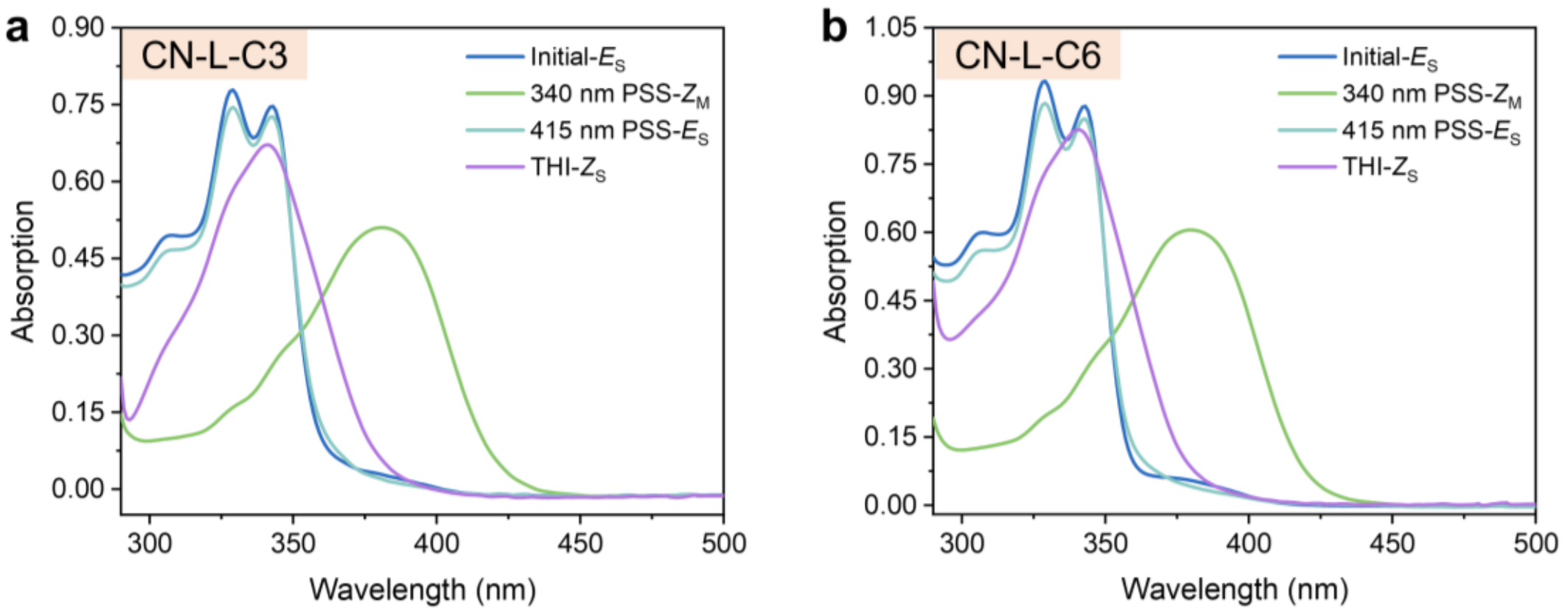

Supplementary Fig. 36 | UV-Vis characterization of functionalized motors rotation in solution. a UV-vis spectra of **CN-L-C3** in different states. **CN-L-C3-**E_S (dark blue line) undergoes photo-isomerization to the Z_M state (green line) upon 340 nm UV irradiation, followed by recovery to the photostationary E_S state (light blue line) upon 415 nm irradiation (3 × 10^{-5} M, toluene, 20 °C). The Z_M state undergoes a thermal helix inversion (THI) to form the Z_S state (purple line) (3×10^{-5} M, toluene, 90 °C, 1 h). **b CN-L-C6** follows a similar rotary cycle to that of **CN-L-C3** (3×10^{-5} M, toluene).

5.2 Thermal Helical Inversion process

Exposure of the unstable isomers **CN-S**-Z_M, **CN-L-C3**-Z_M, **CN-L-C6**-Z_M and **CHO-S**-Z_M samples to darkness induces the onset of the THI step, leading to the formation of the corresponding stable isomers **CN-S**-Z_S, **CN-L-C3**-Z_S, **CN-L-C6**-Z_S and **CHO-S**-Z_S. Based on this, **CN-S**-E_S (5×10^{-5} M), **CN-L-C3**-E_S, **CN-L-C6**-E_S and **CHO-S**-E_S (each at 3×10^{-5} M) were solubilized in toluene and converted them into **CN-S**-Z_M, **CN-L-C3**-Z_M, **CN-L-C6**-Z_M and **CHO-S**-Z_M under UV irradiation. Subsequently, the THI kinetics of **CN-S** and **CHO-S** were measured at 60, 65, 70, 75, and 80 °C, while those of **CN-L-C3** and **CN-L-C6** were determined at 70, 75, 80, 85, and 90 °C. The absorption of **CN-S**-Z_M, **CN-L-C3**-Z_M and **CN-L-C6**-Z_M at 382 nm as well as **CHO-S**-Z_M at 408 nm are plotted (Supplementary Fig. 37 a, c, e, g). Using the Eyring equation for the set of rate constants (k) measured at different temperatures, the half-life ($t_{1/2}$) was calculated of the four compounds at room temperature. Furthermore, by plotting ln(k/T) vs $1/T$ according to the equation, we can calculate $\Delta H^‡$, $\Delta S^‡$, and $\Delta G^‡$. (Supplementary Fig. 37 b, d, f, h and Supplementary Table 1).

$$k = \frac{k_B T}{h} e^{-\Delta G^‡/RT} \quad (1)$$

$$\Delta G^{\ddagger} = \Delta H^{\ddagger} - T\Delta S^{\ddagger} \tag{2}$$

$$\ln\frac{k}{T} = \frac{-\Delta H^{\ddagger}}{R}\cdot\frac{1}{T} + \ln\frac{k_B}{h} + \frac{\Delta S^{\ddagger}}{R} \tag{3}$$

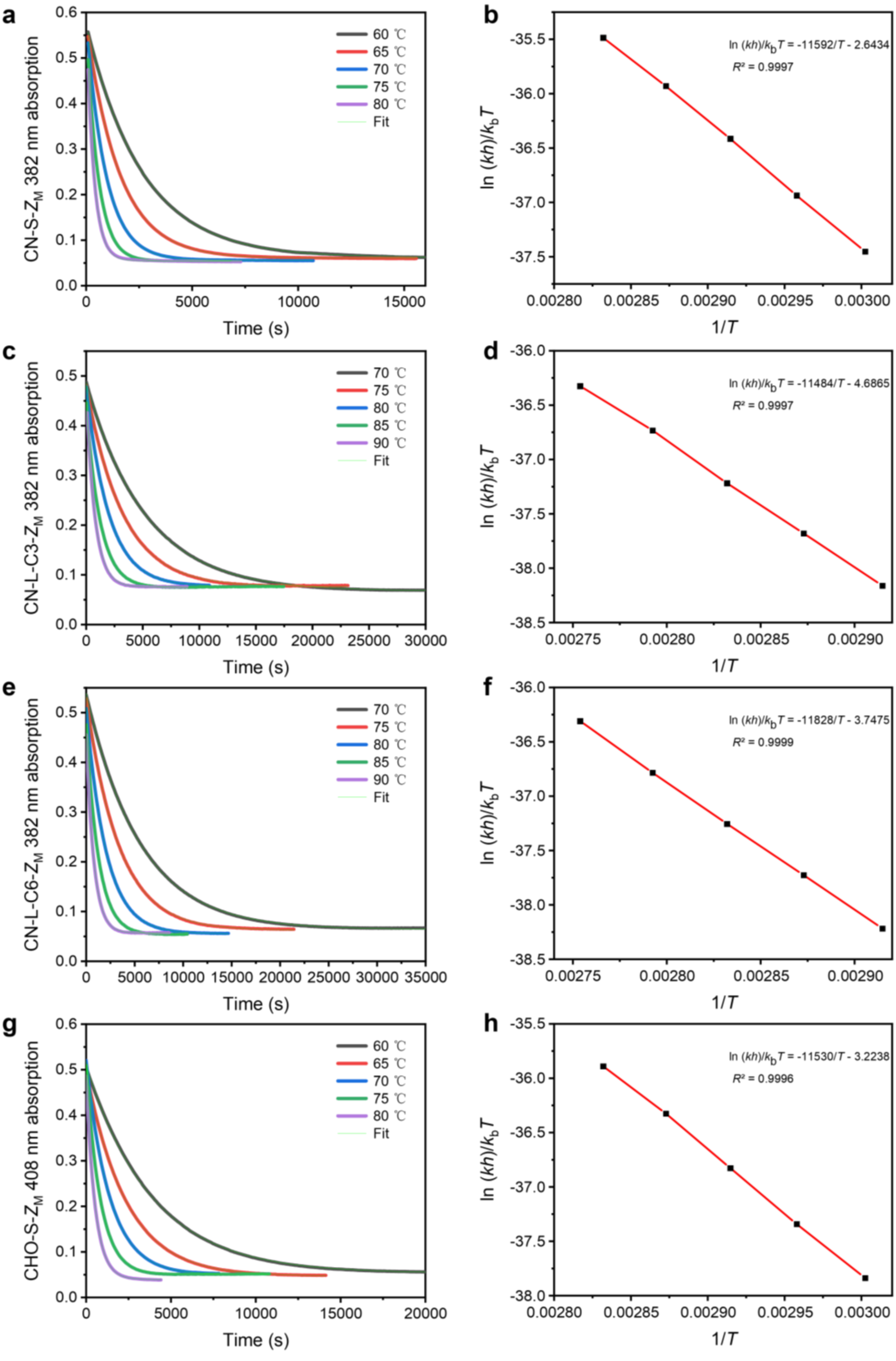

Supplementary Fig. 37 | Characterization of thermal helical inversion process of motors in solution. Time-dependent absorption changes at 382 nm during the THI process of (**a**) **CN-S**-Z_M (5 × 10^{-5} M), (**c**) **CN-L-C6**-Z_M (3 × 10^{-5} M) and (**e**) **CN-L-C3**-Z_M (3 × 10^{-5} M) in toluene, and (**g**) at 408 nm during the THI process of **CHO-S**-Z_M (3 × 10^{-5} M). Eyring plots of the THI step for the $Z_M \rightarrow Z_S$ transition of (**b**) **CN-S**, (**d**) **CN-L-C3**, (**f**) **CN-L-C6**, and (**h**) **CHO-S** in toluene.

Supplementary Table 1 | Summary of the thermodynamic parameters calculated for the CN-S, **CN-L-C3**, **CN-L-C** and **CHO-S**.

	$t_{1/2}$ (h)	$\Delta^{‡}G^{o}$ (kJ/mol)	$\Delta^{‡}H^{o}$ (kJ/mol)	$\Delta^{‡}S^{o}$ (J/mol/K)
CN-S	66.85	102.82	96.38	-21.98
CN-L-C3	358.99	106.91	95.48	-38.97
CN-L-C6	353.51	107.48	98.34	-31.16
CHO-S	96.71	103.72	95.87	-26.80

5.3 Photoisomerization quantum yield

In this study, the Ferrioxalate Chemical Actinometry was employed to determine the photon flux [5,6]. A 3 mL aqueous H_2SO_4 solution (0.05 M, prepared by diluting 270 µL of concentrated 98% H_2SO_4 in 100 mL of water) containing $K_3[Fe(C_2O_4)_3]·3H_2O$ (41 mM, 2.014 g in 100 mL) was placed in a 1 cm quartz cuvette and irradiated at 20 °C for a given period in the dark with a 365 nm LED. An aliquot of 100 µL of irradiated solution was then added to a cuvette containing 1.9 mL of an aqueous H_2SO_4 solution (0.5 M, prepared by diluting 2.7 mL of concentrated 98% H_2SO_4 in 100 mL of water), along with phenanthroline (1 g/L, 100 mg in 100 mL) and NaOAc (122.5 g/L). The mixture was allowed to react for 15 min. The cuvette containing the initial solution (an aqueous H_2SO_4 solution with $K_3[Fe(C_2O_4)_3]·3H_2O$) was irradiated again and this step was repeated to get at least 5 exposures. The absorption at λ=510 nm was measured and compared to an identically prepared non-irradiated sample. The concentration of $[Fe(phenanthroline)_3]^{2+}$ complex was calculated using its molar absorptivity (ε = 11100 M^{-1} cm^{-1}) and considering the dilution. The quantity of Fe^{2+} ions (expressed in mmol) was plotted versus time (expressed in seconds), and the formation rate of Fe^{2+} ions at the given wavelength was obtained from the slope of the linear fit to the equation y=ax+b. This rate can be converted into the photon flux (I) by dividing it by the quantum yield of the $[Fe(phenanthroline)_3]^{2+}$ complex at the wavelength of the used LED and by the probability of photon absorption at the given wavelength ($\Phi^{340\ nm}$=1.22, $\Phi^{365\ nm}$=1.20) [7] of the Fe^{3+} complex (approximately 1 in all cases due to the full absorption mode). The calculated molar photon flux values were: $I^{340\ nm}$=6.45×10^{-6} mE/s (current= 700 mA), $I^{365\ nm}$=2.34×10^{-5} mE/s (current=300 mA).

These values were derived for single-wavelength light sources. Since quantum yield may exhibit

wavelength dependence, this factor represents a potential source of error in the measurements. The solutions of **CN-S**-E_S (8.13×10^{-6} M in DCM, with an absorbance of approximately 0.43 at the irradiation wavelength), **CN-L-C3**- E_S (7.94×10^{-6} M in DCM, absorbance of approximately 0.28), and **CN-L-C6**- E_S (7.63×10^{-6} M in DCM, absorbance of approximately 0.50) were irradiated with UV light at 340 nm to form **CN-S**-Z_M, **CN-L-C3**-Z_M, and **CN-L-C6**-Z_M, respectively. The **CHO-S**-E_S solution (1.46×10^{-5} M in DCM, with an absorbance of approximately 0.3 at the irradiation wavelength) was irradiated with 365 nm UV light to yield **CHO-S**-Z_M. Spectral data were collected by monitoring the absorption changes at the irradiation wavelength. Subsequently, the decay was fitted by the ODE solver present in COPASI, employing the Levenberg-Marquardt algorithm with randomized initial conditions. Quantum yield was calculated by the below equation:

$$\frac{\mathrm{d[A]}}{\mathrm{d}t} = \frac{\Phi_{\text{trans-stable}\rightarrow\text{cis-metastable}} \cdot I \cdot \beta_{\text{trans-stable}}}{N_A \cdot V} + \frac{\Phi_{\text{cis-metastable}\rightarrow\text{trans-stable}} \cdot I \cdot \beta_{\text{cis-metastable}}}{N_A \cdot V} \tag{4}$$

Where I is the molar photon flux, N_A is Avogadro's constant, V is the total volume of the irradiated solution (2 mL), and β is the fraction of photons absorbed by either the E_S or Z_M isomer, determined using the PSS ratio and the corresponding molar extinction coefficients for each isomer. To obtain physically reasonable results, the boundaries for the quantum yield values were fixed between 1×10^{-6} and 1. The LED current (700 mA) was chosen for testing the motors of **CN-S**, **CN-L-C3** and **CN-L-C6**, and the LED current (300 mA) for testing the **CHO-S** motors, and each sample was tested at least 3 times to ensure the reliability of the data. The average values obtained are presented in Supplementary Fig. 38 and Supplementary Table 2.

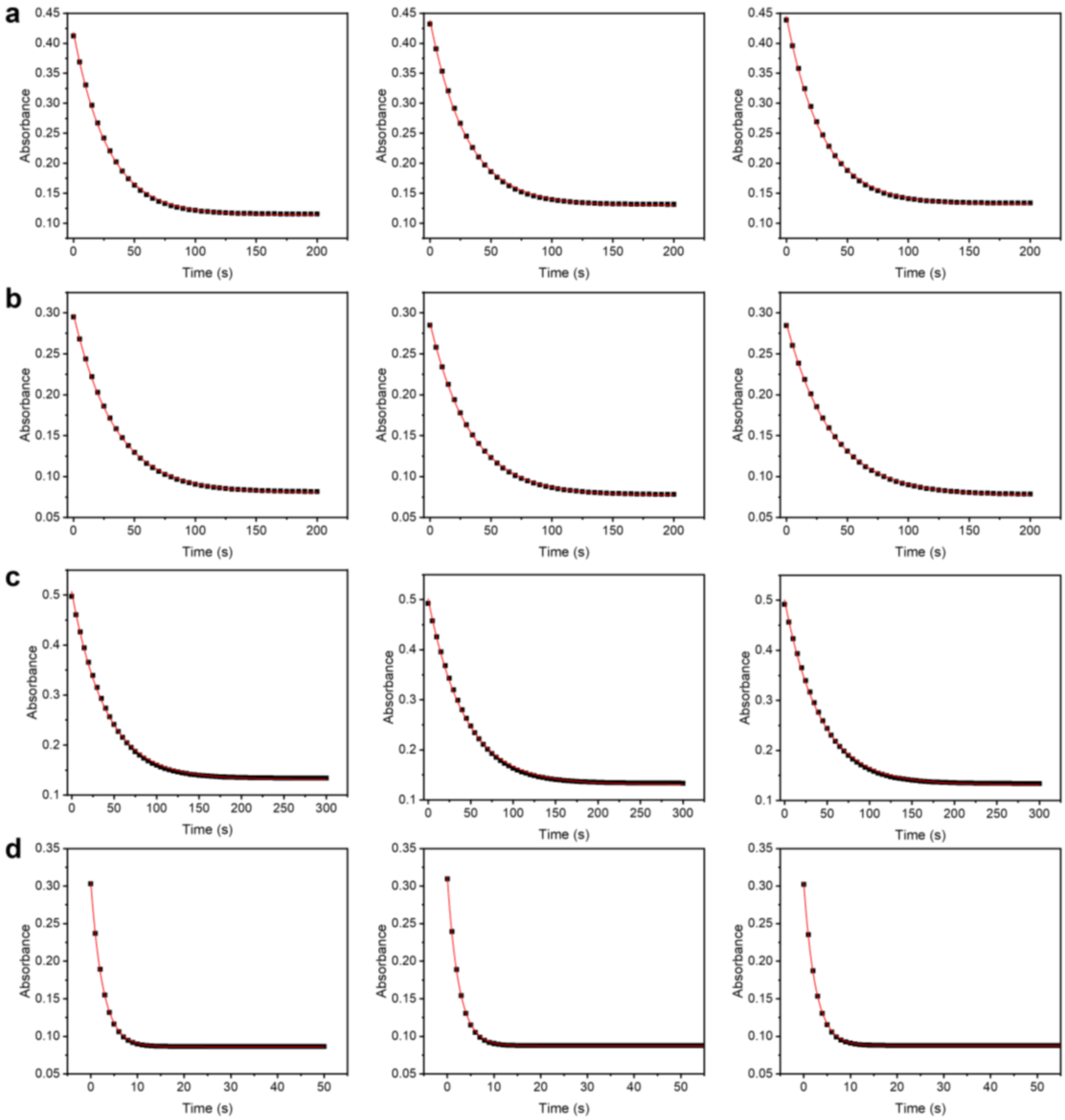

Supplementary Fig. 38 | Quantum yields of photoisomerization of molecular motors in solution. Evolution of the absorption at 340 nm for (**a**) **CN-S**, (**b**) **CN-L-C3**, and (**c**) **CN-L-C6** in DCM under 340 nm irradiation, and (**d**) the absorption changes at 365 nm for **CHO-S** in DCM under 365 nm irradiation. The red line represents the fit obtained with the ODE solver from COPASI.

Supplementary Table 2 | Spectroscopic characteristics of molecular motors. Molar extinction coefficients, PSS and photoisomerization quantum yield data in DCM for motors **CN-S, CN-L-C3, CN-L-C6** and **CHO-S**.

Motors	ε_{E_S} ($M^{-1}cm^{-1}$)	ε_{Z_M} ($M^{-1}cm^{-1}$)	PSS $E_S : Z_M$	Φ $E_S \rightarrow Z_M$	Φ $Z_M \rightarrow E_S$
CN-S	52632.6	11468.4	10 : 90	10.8%±0.5%	5.6%±0.5%
CN-L-C3	36283.8	9239.7	3 : 97	13.0%±0.7%	1.4%±0.7%
CN-L-C6	64755.0	14908.8	5 : 95	6.8%±0.2%	1.6%±0.04%
CHO-S	20743.8	5199.4	3 : 97	84.6%±1.5%	16.3%±0.4%

6 Preparation of LC cells

Square glass pieces (2 cm × 2 cm) were cut and sequentially washed with anhydrous ethanol and acetone, then wiped with dust-free paper and blown dry with N_2 to clean the glass surface. The glass substrates were spin-coated with a polyimide (PI) solution promoting planar LC alignment for 1 min at 5000 rpm, followed by placing them on a hot stage at 80 °C for 1 h, and then heated to 180 °C overnight to form the PI planar alignment layer. Then the planar substrates can be rubbed using a velvet cloth to form an alignment layer. Two glass substrates with the same alignment layer are glued together in parallel with UV-curable adhesive containing 25 μm spacers to obtain a planar LC cell.

7 Preparation LC mixtures and LCPNs

Various molecular optical units—including aldehyde motors (**CHO-L**, **CHO-S**), cyano motors (**CN-L-C3**, **CN-L-C6**, **CN-S**), azobenzene (**Azo**), and second-generation motor **MSG**—were each dissolved in DCM together with liquid crystal monomers (C6M, C6BP, C6BP6, and the non-polymerizable low molar mass LC **E7**) and photoinitiators. These compounds were thoroughly mixed and evaporated overnight at 60 ℃ to remove the solvent to obtain the LC mixtures.

The mixtures were injected into planar-aligned liquid crystal cells (the thickness of the cell is 25 μm) at 50 ℃. The chemical structures of the components and their ratios are summarized in Supplementary Table 3. The mixtures were left at 50 ℃ for 30 min to improve the molecular arrangement and then polymerized by irradiation with blue light (λ= 470 nm) for 10 min. After polymerization, the LC cell was annealed at 100 °C for 10 min, cooled to room temperature and opened so that the desired film could be peeled off the glass substrate.

Supplementary Table 3 | Compositions of liquid crystal photopolymerizable mixtures.

	Structures	LCPN (wt%)
Molecular optical units		3
C6M	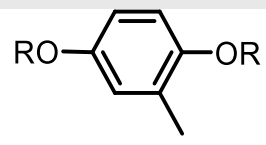	22
C6BP	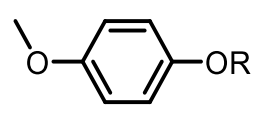	50
C6BP6	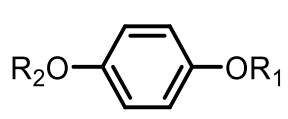	15
E7	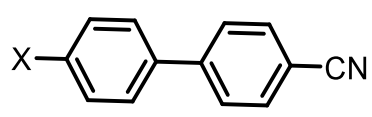	10
Irg 819	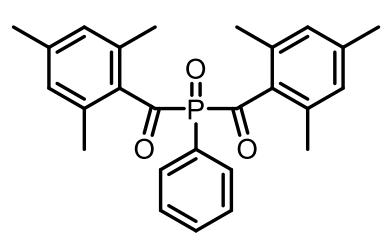	+2

R_1 = R = 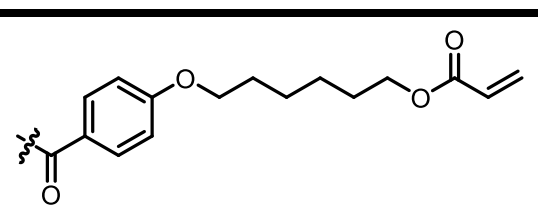 R_2 = 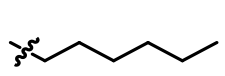

8 DSC characterization of LCPNs

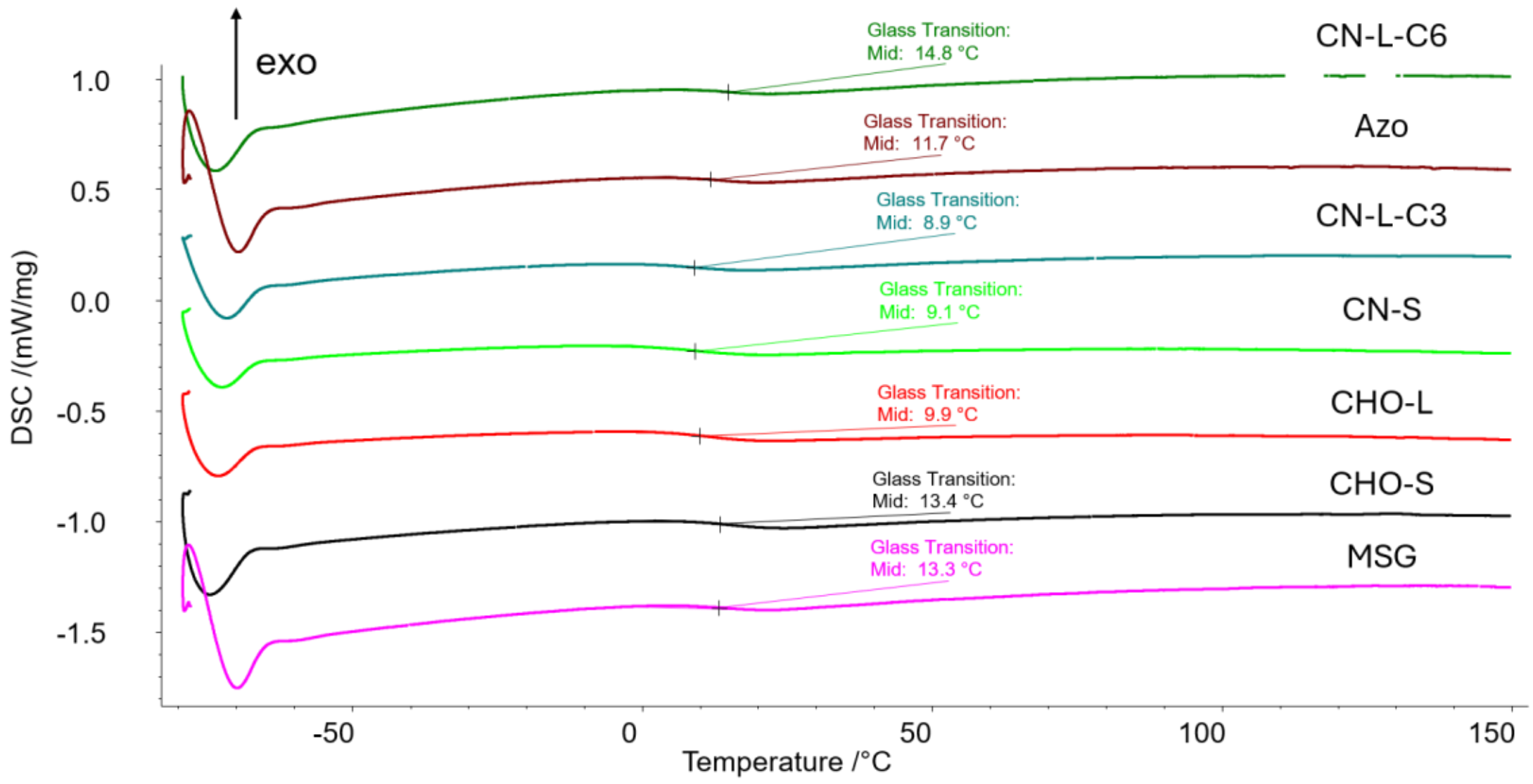

Supplementary Fig. 39 | Phase behavior of the motorized LCPNs. DSC curves of all LCPNs studied in the work. Glass transition temperatures are indicated in the graph. The second heating curves are shown.

9 Comparative study on the photo-actuation performance of LCPNs driven by different photo-responsive units

LCPNs containing different photo-responsive units were cut into ribbons with dimensions of 1 mm × 1 cm, and one end of each strip was fixed onto a glass substrate. The samples were then irradiated with UV light (λ= 365 nm). To ensure UV exposure across all ribbons uniformly, the diameter of the UV light spot was controlled at 5 cm, which was sufficient to cover the entire sample area. Additionally, the light intensity at 365 nm was specifically adjusted to be consistent with that of the 340 nm irradiation used in the main text, enabling a direct comparison of actuation performance under different wavelengths.

All ribbons were in an upright position in the initial state, and the ribbons were gradually bent towards the light with the increase of irradiation time (Supplementary Movie 2). Supplementary Fig. 40 shows the time-dependent tip displacement curves of the various LCPN ribbons during UV irradiation. As can be seen from the figure, under 365 nm UV irradiation, the LCPN films based on aldehyde motors (**CHO-L** and **CHO-S**) exhibited significantly faster response rates compared to other systems, among which CHO-L displayed both the fastest actuation and the largest tip displacement (deformation), indicating the most efficient photo-actuation performance. The performance of **CHO-S** was slightly lower than that of **CHO-L** but comparable to that of the azobenzene-doped LCPN films. In contrast, the ribbons of LCPNs constructed based on cyano motors (**CN-L-C3, CN-L-C6, CN-S**) responded slower and with smaller tip displacements, suggesting the actuation efficiency at this wavelength was significantly limited. In addition, the **MSG** films demonstrated the slowest response rate and the smallest deformation, and their overall driving performance was significantly lower than that of other types of photo-responsive systems. The results of this comparative experiment indicate that the structure of the photo-responsive units significantly affects the driving behavior of the materials under different wavelengths of light

stimulation, providing an important basis for optimizing the photo-responsive performance of LCPN.

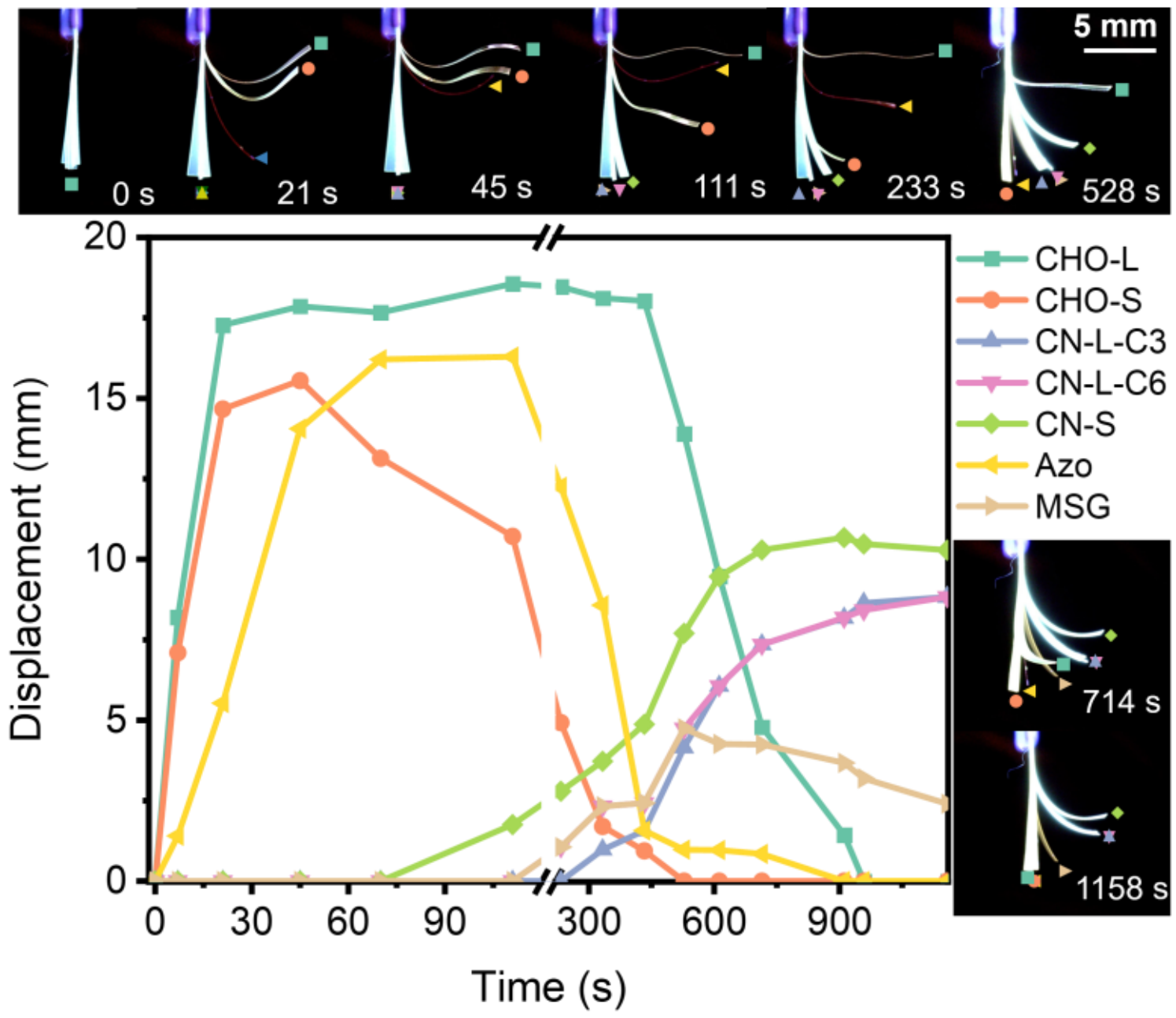

Supplementary Fig. 40 | Comparative photoactuation study of the LCPNs. Tip displacement curves of different LCPN ribbons upon 365 nm UV light irradiation. The snapshots illustrate the actuation process, with the corresponding sample names indicated using scatters matching the displacement curves. The intensity of 365 nm light was 2.2 mW/cm².

10 Fatigue study of bending motion and bending under water

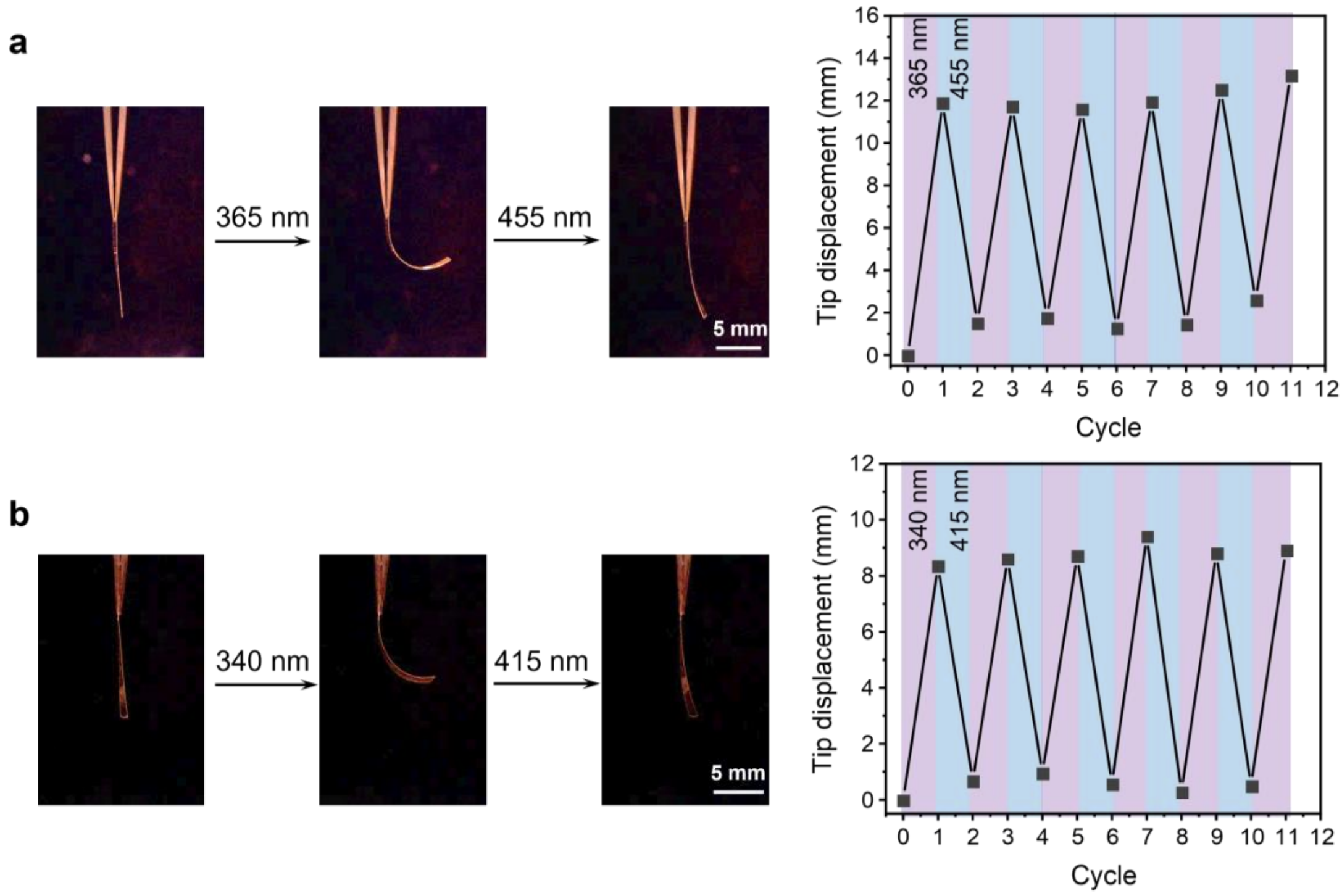

Supplementary Fig. 41 | Fatigue study of actuations processes. Reversible bending behaviour of the **CHO-L** (**a**) and **CN-L-C6** (**b**) polymer ribbons upon UV/blue light irradiation. Molecular alignment is parallel to the long axis

of the ribbon. The light source is on the right.

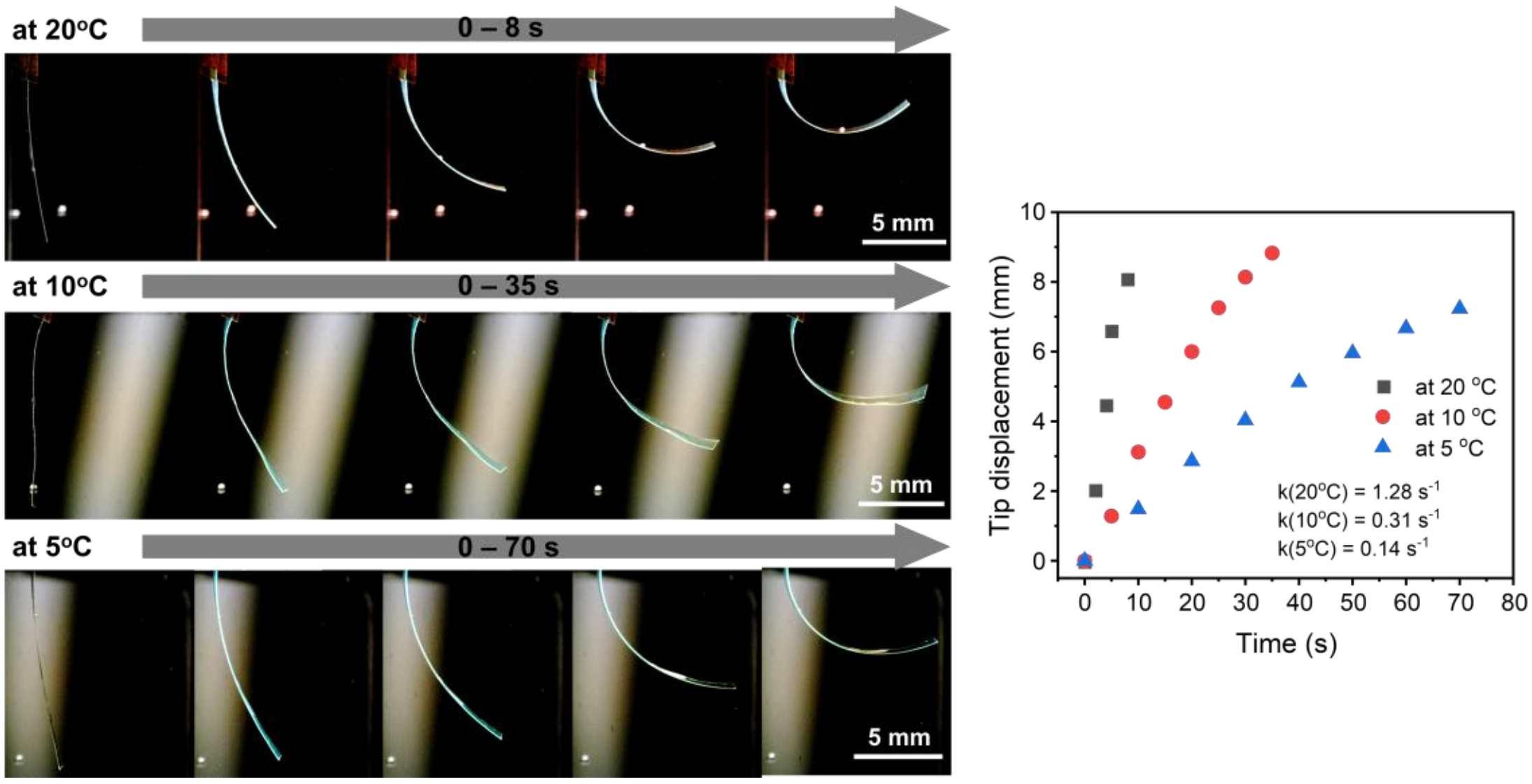

Supplementary Fig. 42 | **Actuation under water.** Bending behaviour of the **CHO-L** polymer ribbon operating under water of decreasing temperature. Kinetic curved of the bending (at the bottom). Rate constants of the process are provided in the graph. In all cases, the ribbons actuate toward the light source (on the right). However, the kinetics are temperature dependent. Lower temperatures lead to slower bending, consistent with the increased elastic modulus and viscoelastic damping at lower temperatures. Importantly, actuation still occurs even at temperatures below the glass transition, demonstrating that the photochemically generated forces from the motors are sufficient to overcome the network stiffness and drive deformation.

11 DFT calculations

The ground state structures of motors were optimized with the composite method r^2SCAN-3c, [8] using the conductor-like polarizable continuum solvent model CPCM (acetonitrile). [9] We performed a vertical excitation calculation for optimized geometries of E_S-**CN-L-C6** and E_S-**CHO-L**, by using the time-dependent density functional theory method (TD-DFT) without Tamm-Dancoff approximation, with ωB97X-D4 [10] hybrid functional and def2-TZVPP basis set using solvent model CPCM (acetonitrile) considering 30 singlet transitions. Orientations of $S_0 \rightarrow S_1$ transition dipole moments (TDMs) were extracted from those calculations to interpret the spectral data on motor alignment in the liquid crystal network. All calculations have been performed using the Orca 5.0.4 package [11].

Molecular geometry modulation

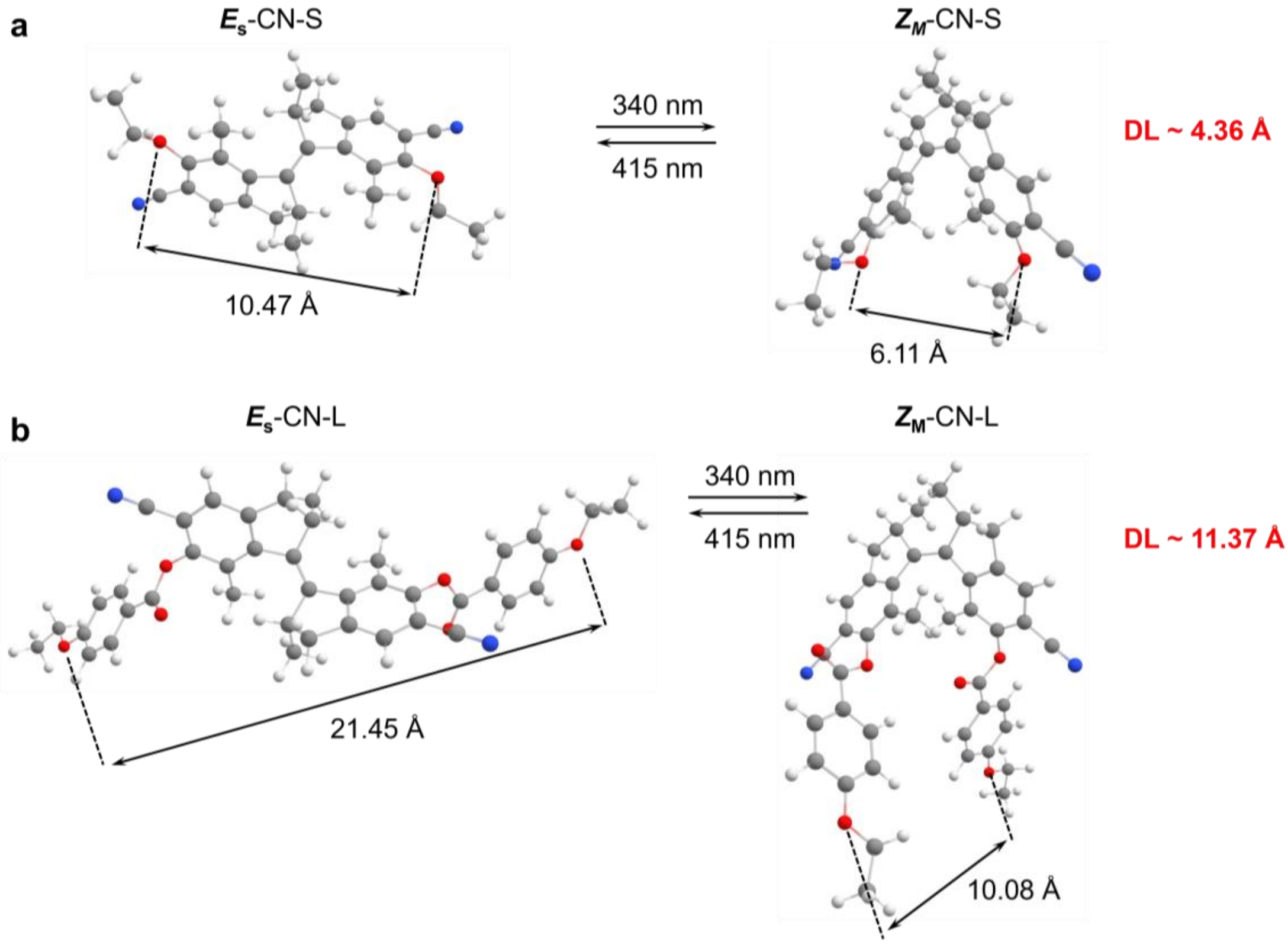

Supplementary Fig. 43 | Molecular geometry changes upon isomerization of molecular motors. DFT-optimized structure for the lowest-energy conformer of E_S-**CN-S**, Z_M-**CN-S**, E_S-**CN-L**, and Z_M-**CN-L**. To simplify calculations, long aliphatic spacers and acrylic terminal fragments were replaced by ethoxy-substituents. The length of the rigid core was estimated as the distance between lateral oxygen atoms. Final geometry optimization: r^{2}scan-3c/ CPCM (ACN). For the short-core **CN-S** motor (**a**), photoisomerization induces a molecular contraction of approximately 4.36 Å. In contrast, the long-core **CN-L** motor (**b**) exhibits a substantially larger contraction of ~11.37 Å. A comparable trend is expected for the aldehyde-substituted motors (**CHO-S** and **CHO-L**). These results indicate that extending the rigid core roughly doubles the molecular shape change upon photoisomerization, which provides a possible mechanistic basis for the observed increase in photogenerated force (as summarized in Supplementary Table 4 of the main text).

12 Molecular alignment studies

Molecular alignment probe (network alignment)

We quantified the molecular order within the networks using a dichroic probe (Disperse Red 1, DR1) combined with polarized UV-vis spectroscopy (Supplementary Fig. 44). Thin films (10 μm) of **CN-L-C6** and **CHO-L** networks doped with ~0.5 wt% DR1 were prepared in cells promoting unidirectional alignment. Polarized absorbance spectra were recorded as a function of polarization angle relative to the alignment direction (defined by the rubbing of the polyimide aligning layers). The degree of order was quantified via the linear dichroism parameter:

$$D = \frac{A_{\parallel} - A_{\perp}}{A_{\parallel} + A_{\perp}} \tag{5}$$

where $A_{//}$ and $A_{\perp}$ are the absorbances measured parallel and perpendicular to the director,

respectively. In this definition, D=0 corresponds to an isotropic state, while D=1 indicates perfect alignment.

Both networks exhibit a high degree of molecular order. Notably, the **CN-L-C6** system shows slightly higher D values, suggesting improved alignment, which we attribute to the specific structure of the cyano-substituted motor core that perturbs the liquid crystalline order to a lesser extent.

Importantly, UV irradiation does not result in any measurable change in the dichroism parameter (Supplementary Fig. 44), indicating that the overall liquid crystalline order remains unchanged during actuation. This observation rules out mechanisms involving large-scale reorganization of the network and supports a model in which actuation arises from local, directional motion of the molecular motors.

These results collectively support the conclusion that the observed shape change and force generation originate from the rotational motion of the molecular motors, which exert forces on the polymer network strands, rather than from changes in network morphology or phase behavior.

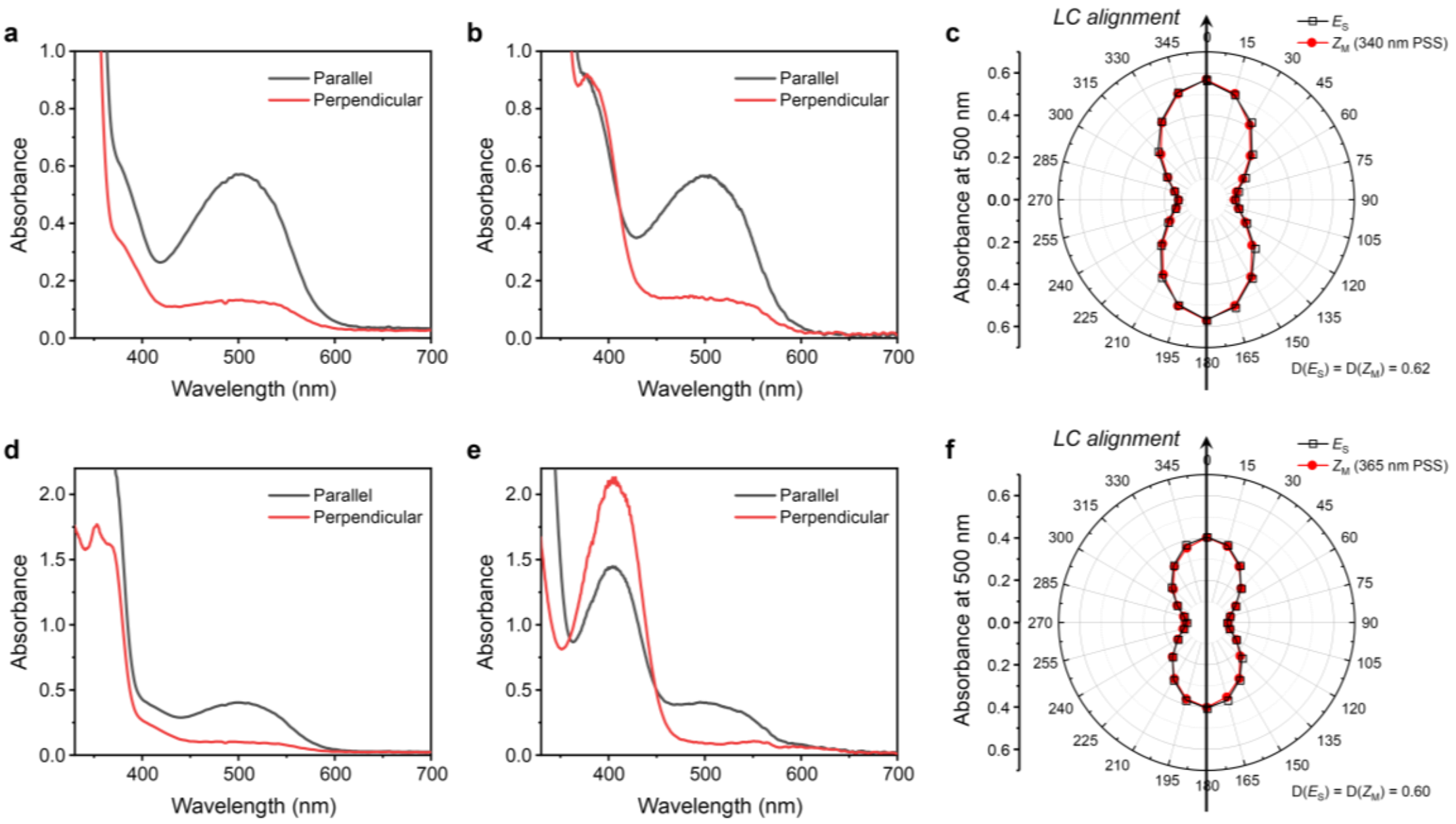

Supplementary Fig. 44 | Polymer network alignment study. Polarized light absorbance spectra and corresponding polar plots of **CN-L-C6** (**a, b, c**) and **CHO-L** (**d, e, f**) films doped with DR1 dye before and exposure to UV light. Parallel and Perpendicular denote to the absorbance components polarized in respect to the LC alignment. Dichroism values (D) are provided in the plots **c** and **f**.

Molecular motors alignment

We prepared uniaxially aligned thin films (~2 μm) of **CN-L-C6** and **CHO-L** and quantified molecular orientation using polarized light UV-vis spectroscopy. By measuring the absorbance as a function of the angle between the polarization direction and the liquid crystal director, we constructed polar plots of the $S_0 \rightarrow S_1$ transition. The orientation of the corresponding transition dipole moments (TDMs) was independently obtained from TD-DFT calculations (ωB97X-D/def2-TZVPP, CPCM acetonitrile), confirming the assignment of the absorption bands and its location in respect to the chemical structure (Supplementary Fig. 45). We found that the CN-based motor exhibits significantly higher orientational order compared to the CHO analogue (D ≈ 0.37 vs. 0.22).

Given that the photogenerated force arises from directional molecular contraction along the motor axis, the degree of alignment directly determines how effectively individual molecular motions are translated into macroscopic stress. In this context, the higher degree of alignment of the CN motors leads to a more cooperative and directional force generation within the network, which explains the larger measured photogenerated forces despite their lower quantum yield.

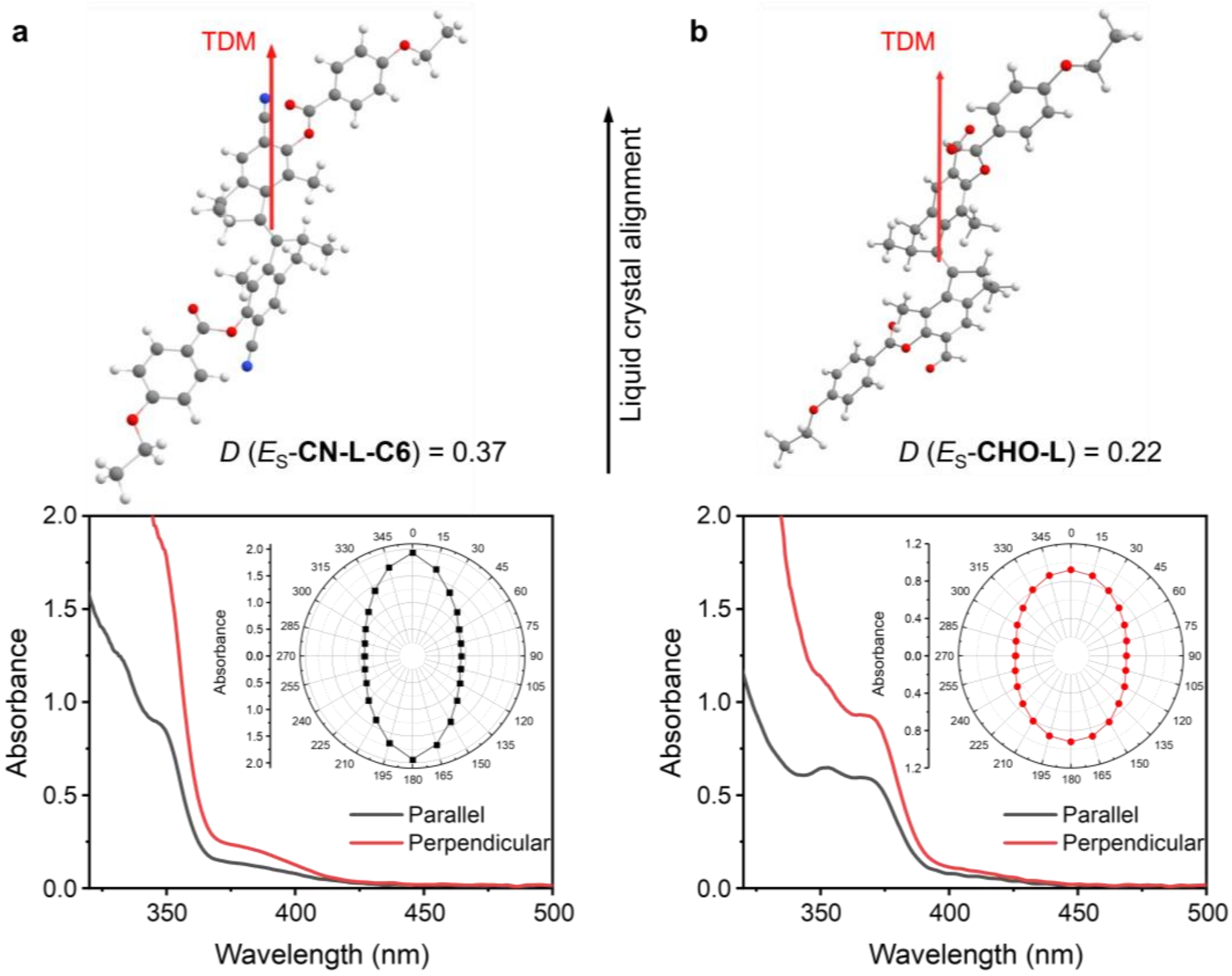

Supplementary Fig. 45 | Molecular motors alignment study. Scheme demonstrating an alignment of molecular motor **CN-L-C6** (**a**) and **CHO-L** (**b**) (DFT optimized geometry) with respect to alignment of the liquid crystals. TDM – transition dipole moment. Polarized absorbance spectra of molecular motor in unidirectionally aligned LCPNs. Angular dependence as well as dichroism value (*D*) showed in the inserts.

13 Thermal imaging study

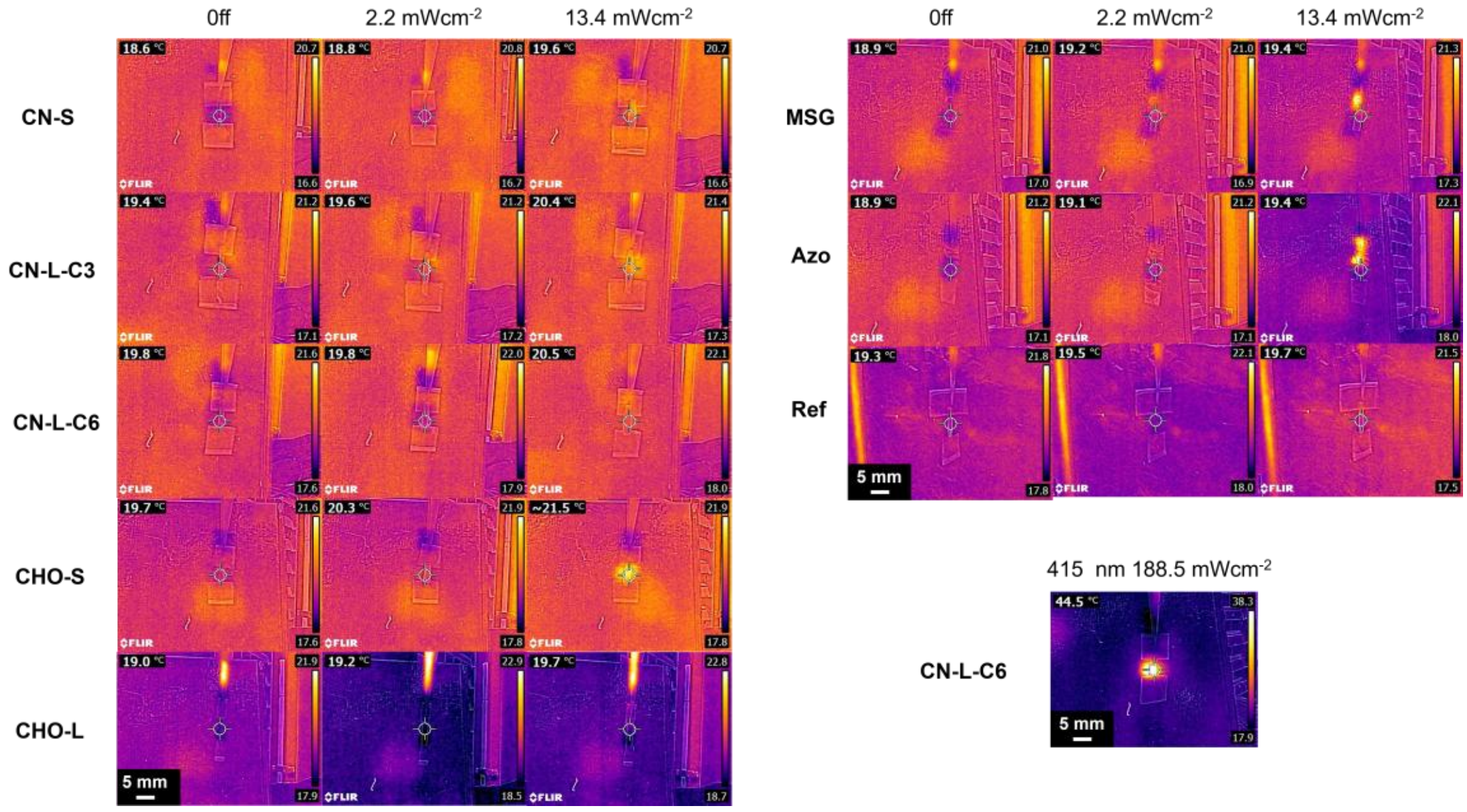

Supplementary Fig. 46 | Thermal imaging study. IR imaging of the LCPN ribbons subjected to UV irradiation. Light intensity is shown on the top. All cyano-motors-continuing films were irradiated with 340 nm, while the others with 365 nm UV light. In reference LCPN (Ref) photoactive molecules were replaced by C6M crosslinker. Bottom right panel: IR image of **CN-L-C6** film under high-intensity blue (415 nm) light. It is clearly seen, neither irradiation at 2.2 mW/cm^2 (used for bending experiments) nor at 13.4 mW/cm^2 (used for photomechanical characterization) results in a significant temperature increase. At the highest UV intensity, a minor temperature rise in the range of 0.5-1.7 °C was observed for LCPNs containing photoactive moieties, while the reference LCPN lacking photoresponsive groups exhibited a comparable increase of approximately 0.4 °C. In contrast, upon irradiation at substantially higher light intensity (as employed for back-switching of the **CN-L-C6** film), a pronounced temperature increase of approximately 25 °C was detected.

14 Mechanical properties

Assumptions for molecular force estimation

To provide molecular level perspective on the mechanical force performed by the various light responsive units, we calculated the normalized force generated per molecular motor/switch. It is important to emphasize that these values are intended as approximate estimates for the purpose of contextual comparison and to illustrate general trends in transduction efficiency, rather than as absolute physical constants.

The calculations are based on the following framework and assumptions: i) The parameters for molecular density and loading are taken from the reported data in corresponding published literature. ii) A central assumption is that 100% of the photoactive units incorporated into the material are successfully activated by light and contribute equally to the macroscopic photogenerated stress. In a practical experimental setting, this represents an upper limit estimate. It is highly probable that a fraction of the motors remains inactive or exhibit reduced switching efficiency due to local steric hindrance within the surrounding matrix or attenuation of light intensity through the sample thickness. iii) We acknowledge that the performance values of molecular motors cited from literature may not reflect the fully optimized performance of those specific molecules within their environment.

Because the complex interplay between molecular motion and network mechanics involves numerous variables (e.g., local order parameters, viscoelastic relaxation, and crosslinking density, etc.), a precise determination of individual molecular force is extremely challenging.
Consequently, we provide these normalized values as a comparative tool to highlight the relative enhancements achieved through the structural optimization of the motor core. These estimates support the general trend that rigid-core extension and spacer length optimization lead to a significantly more efficient translation of molecular motion into macroscopic mechanical work within this standardized network architecture and that media represented by the aligned liquid crystalline network seems very efficient to harness molecular rotation of motors.

To estimate the photo-generated force contributed by individual photo-responsive units within the LCPN film, the following methodology was adopted:
Given the dimensions of the LCPN film and assuming a material density of ρ = 1×10^{-3} g/mm³, the mass of the LCPN film can be calculated as:

$$m_{\mathrm{LCPN}} = \rho \cdot V \tag{6}$$

where V is the volume of the film.
The photo-responsive units were incorporated into the polymer network at a concentration of 3 wt%. Thus, the total mass of photo-responsive units within the film can be determined by:

$$m_{\mathrm{units}} = 3\ \mathrm{wt\%} \times m_{\mathrm{LCPN}} \tag{7}$$

The total mass of the photo-responsive unit cores can be calculated by:

$$m_{\mathrm{cores}} = \frac{M_{\mathrm{core}}}{M_{\mathrm{unit}}} \times m_{\mathrm{units}} \tag{8}$$

Where M_{core} is the molar mass of the core, M_{unit} is the molar mass of the unit.
During the mechanical testing under light irradiation, the total force output of the LCPN film (F_{LCPN}) can be recorded and is therefore known. Assuming that the photomechanical response arises solely from the photo-responsive cores embedded in the network, the force generated by the photo-responsive cores (F_{cores}) can be calculated by:

$$F_{\mathrm{cores}} = \frac{F_{\mathrm{LCPN}}}{m_{\mathrm{cores}}} \tag{9}$$

The mass of a single photo-responsive molecule is calculated based on its molar mass M, using Avogadro's number N_{A}=6.022×10^{23} mol^{-1}:

$$m_{\mathrm{one\ unit}} = \frac{M_{\mathrm{unit}}}{N_{\mathrm{A}}} \tag{10}$$

Assuming that the photomechanical response is solely due to the embedded photoresponsive units, the average photogenerated force per unit can be calculated by:

$$F_{\mathrm{one\ unit}} = \frac{m_{\mathrm{one\ unit}}}{m_{\mathrm{LCPN}}} \times F_{\mathrm{LCPN}} \tag{11}$$

This calculation allows the estimation of photo-generated force on a per-molecule unit (Supplementary Table 4), which is critical for evaluating the efficiency of photo-responsive elements and comparing them across different molecular designs and material systems.

Supplementary Table 4 | Estimation of photogenerated forces. Summary of the size, mass and molecular weight

of the photo-responsive units in each LCPN system, as well as the total photogenerated force under external stimulation and the calculated force generated per photo-responsive unit and per gram of active molecular unit (motor core in the given case).

	L (mm)	W (mm)	H (mm)	m_{LCPN} (g×10^{-4})	m_{units} (g×10^{-6})	M (g/mol)	m_{unit} (g×10^{-21})	m_{cores} (g ×10^{-6})	F_{LCPN} (mN)	F_{cores} (N/g)	F_{unit} (N×10^{-20})
**CHO-L**	4.93	1.97	0.025	2.43	7.28	925	1.54	4.84	25.1	5190	529
**CHO-S**	5.3	2	0.025	2.65	7.95	684.9	1.14	4.35	16.4	3770	235
**CN-L-C3**	5.8	1.97	0.025	2.86	8.57	834.9	1.39	6.25	37.1	5940	600
**CN-L-C6**	6.3	1.97	0.025	3.10	9.31	919.1	1.53	6.16	40.2	6520	659
**CN-S**	5.2	1.8	0.025	2.34	7.02	678.9	1.13	3.81	22.1	5800	355
**Azo**	6.23	2.07	0.025	3.22	9.67	522.3	0.87	3.93	21.7	5520	195
**MSG**	5.07	1.67	0.025	2.12	6.35	662.9	1.10	3.38	3.6	1070	62

Calculation of Photogenerated Force in the Hydrogel System 1 [12]

In the hydrogel system, a gel strip was observed to lift a weight of approximately 55.5 mg over a vertical distance of 3.5 mm under light irradiation. According to the data reported in this article, the gravitational potential energy of the driving process is 2 μJ. It is assumed that this potential energy is caused by the isomerization of 7.95 x 10^{-4} mmol of motor (0.27 mg of motor cores). Given that 1 J = 1 N·m, the force F_{hydrogel} generated by gel strip can be calculated from the energy E and the displacement d using the equation:

$$F_{\mathrm{hydrogel}} = \frac{E}{d} = \frac{2\ \mu\mathrm{J}}{3.5\ \mathrm{mm}} = 5.71 \times 10^{-4}\ \mathrm{N}$$

Using Avogadro's number N_A=6.022×10^{23} mol^{-1}, the number of motors N can be calculated by:

$$N = n \times N_{\mathrm{A}} = 7.95 \times 10^{-4}\ \mathrm{mmol}\ \times 6.022 \times 10^{23}\ \mathrm{mol}^{-1} = 4.79 \times 10^{17}$$

The force F_{motor} generated by a single motor is:

$$F_{\mathrm{motor}} = \frac{F_{\mathrm{hydrogel}}}{N} = \frac{5.71 \times 10^{-4}\ \mathrm{N}}{4.79 \times 10^{17}} = 1.19 \times 10^{-21}\mathrm{N}$$

Assuming that the force F_{hydrogel} generated by the photoactive motor cores under UV irradiation. Therefore, the corresponding force generated by the gram of motor cores can be estimated using the relation:

$$F_{\mathrm{cores}} = \frac{F_{\mathrm{hydrogel}}}{m_{\mathrm{cores}}} = \frac{5.71 \times 10^{-4}\ \mathrm{N}}{0.27\ \mathrm{mg}} = 2.11\ \mathrm{N} \cdot \mathrm{g}^{-1}$$

Calculation of Photogenerated Force in the gel System 2 [13]

In a previously reported gel system, it is known that a motor-gel composite contained 13 mg of molecular motors, including 0.4 mg of photoactive motor cores, and could lift a 1.15 g weight. Based on the reported data, the total force exerted during this lifting process was calculated to be $F_{gel} = 1.13 \times 10^{-2}$ N, which corresponds to 1.59×10^{-11} nN per motor actually contributing to the lifting motion.

Assuming that the entire force originates from the photoactive motor cores, the specific force generated per unit mass of motor core can be estimated as follows:

$$F_{\mathrm{cores}} = \frac{F_{\mathrm{gel}}}{m_{\mathrm{cores}}} = \frac{1.13 \times 10^{-2}\mathrm{N}}{0.4\ \mathrm{mg}} = 28.3\ \mathrm{N} \cdot \mathrm{g}^{-1}$$

This value represents the light-induced force generated by the motor cores embedded within the gel matrix.

Calculation of Photogenerated Force in the fiber [14]

In a motor-based fiber system, it was reported that a 0.4 mg piece of paper was lifted by 1.25 cm during light actuation. The total mechanical work done by the fiber in this process was 0.05 μJ. Given that 1 J = 1 N·m, the total photogenerated force can be calculated as:

$$F_{\mathrm{fiber}} = W \cdot H = 0.05\ \mu\mathrm{J} \times 1.25\,\mathrm{cm} = 4 \times 10^{-6}\ \mathrm{N}$$

The fiber had a diameter (d) of 300 μm and a length (L) of 8 mm, resulting in a volume:

$$V = \pi \times \left(\frac{d}{2}\right)^2 \times L = 3.14 \times \left(\frac{300\mu\mathrm{m}}{2}\right)^2 \times 8\ \mathrm{mm} = 0.565\ \mathrm{mm}^3$$

Assuming a material density of $\rho = 1 \times 10^{-3}$ g/mm³, the total mass of the fiber is:

$$m_{\mathrm{fiber}} = \rho \cdot V = 1 \times 10^{-3}\mathrm{g/mm}^3 \times 0.565\ \mathrm{mm}^3 = 5.65 \times 10^{-4}\ \mathrm{g}$$

The content of molecular motors in the material is known to be 5 wt%, therefore the total mass of motors in the fiber strip can be calculated as:

$$m_{\mathrm{motors}} = 5\ \mathrm{wt\%} \times\ m_{\mathrm{fiber}} = 2.83 \times 10^{-5}\ \mathrm{g}$$

The molar mass of the motor is M_{motor} = 992.6 g/mol, so the mass of a single motor molecule is:

$$m_{\mathrm{motor}} = \frac{M_{\mathrm{motor}}}{N_{\mathrm{A}}} = \frac{992.6\ \mathrm{g} \cdot \mathrm{mol}^{-1}}{6.022 \times 10^{23}\ \mathrm{mol}^{-1}} = 1.65 \times 10^{-21}\mathrm{g}$$

Assuming the photogenerated force originates solely from the photoisomerization of motor molecules, the force generated per motor is:

$$F_{\mathrm{motor}} = \frac{m_{\mathrm{motor}}}{m_{\mathrm{motors}}} \times F_{\mathrm{fiber}} = \frac{1.65 \times 10^{-21}\mathrm{g}}{2.83 \times 10^{-5}\mathrm{g}} \times 4 \times 10^{-4}\mathrm{N} = 2.33 \times 10^{-22}\mathrm{N}$$

The molar mass of the motor core is M_{core} = 453.1 g/mol, so the total mass of motor cores in the fiber is:

$$m_{\mathrm{cores}} = \frac{M_{\mathrm{core}}}{M_{\mathrm{motor}}} \times m_{\mathrm{motors}} = \frac{453.1\ \mathrm{g/mol}}{992.6\ \mathrm{g/mol}} \times 2.83 \times 10^{-5}\mathrm{g} = 1.29 \times 10^{-5}\mathrm{g}$$

Assuming the entire force is generated by the motor cores, the specific light-induced force per gram of core is:

$$F_{\mathrm{cores}} = \frac{F_{\mathrm{fiber}}}{m_{\mathrm{cores}}} = \frac{4 \times 10^{-4}\ \mathrm{N}}{1.29 \times 10^{-5}\mathrm{g}} = 0.31\ \mathrm{N/g}$$

Calculation of Photogenerated Force in the LCPN [15]

Under UV irradiation, the maximum photogenerated force of the LCPN system was measured to be F_{LCPN} = 3 mN. The dimensions of the LCPN strip are known as follows: Length (L) = 1 cm, Width (W) = 1 mm, and Thickness (H) = 50 μm, thus the volume (V) of the strip can be calculated as:

$$V = L \times W \times H = 10\ \mathrm{mm}\ \times\ 1\ \mathrm{mm}\ \times\ 0.05\ \mathrm{mm}\ =\ 0.5\ \mathrm{mm}^3$$

Assuming the density of the LCPN film is $\rho = 1 \times 10^{-3}$ g/mm³, the total mass of the film is:

$$m_{\mathrm{LCPN}} = \rho \cdot V = 1\ \times\ 10^{-3}\ \mathrm{g/mm^3}\ \times\ 0.5\ \mathrm{mm}^3\ =\ 5\ \times\ 10^{-4}\ \mathrm{g}$$

The motors were incorporated into the polymer network at a concentration of 3 wt%. Thus, the total mass of motorss within the film can be determined by:

$$m_{\mathrm{motors}} = 3\ wt\% \times m_{\mathrm{LCPN}} = 0.03\ \times\ 5\ \times\ 10^{-4}\ \mathrm{g}\ =\ 1.5\ \times\ 10^{-5}\ \mathrm{g}$$

The molar mass of the molecular motor is known to be M_{motor} = 656.4 g/mol, therefore, the mass of a single motor molecule is:

$$m_{\mathrm{motor}} = \frac{M_{\mathrm{motor}}}{N_{\mathrm{A}}} = \frac{656.4\ \mathrm{g \cdot mol^{-1}}}{6.022 \times 10^{23}\ \mathrm{mol^{-1}}} = 1.09 \times 10^{-21}\mathrm{g}$$

Assuming that the entire photo-generated force originates from the photoisomerization of embedded molecular motors, the force generated per motor is:

$$F_{\mathrm{motor}} = \frac{m_{\mathrm{motor}}}{m_{\mathrm{motors}}} \times F_{\mathrm{LCPN}} = \frac{1.09 \times 10^{-21}\mathrm{g}}{1.5 \times 10^{-5}\mathrm{g}} \times 3 \times 10^{-3}\mathrm{N} = 2.18\ \times\ 10^{-19}\ \mathrm{N}$$

The molar mass of the motor core is M_{core} = 346.2 g/mol, thus the total mass of motor cores in the film is:

$$m_{\mathrm{cores}} = \frac{M_{\mathrm{core}}}{M_{\mathrm{motor}}} \times m_{\mathrm{motors}} = \frac{346.2\ \mathrm{g/mol}}{656.4\ \mathrm{g/mol}} \times 1.5 \times 10^{-5}\mathrm{g} = 7.91 \times 10^{-6}\ \mathrm{g}$$

Assuming the photo-generated force is caused solely by the motor cores, the specific force per gram of motor core is calculated as:

$$F_{\mathrm{cores}} = \frac{F_{\mathrm{LCPN}}}{m_{\mathrm{cores}}} = \frac{3 \times 10^{-3}\ \mathrm{N}}{7.91 \times 10^{-6}\ \mathrm{g}} = 379\ \mathrm{N/g}$$

15 Fluorescence properties of cyano and aldehyde motros

To elucidate the origin of fluorescence, we first performed studies of the molecular motors in solution. We found that the CN-substituted motor exhibits approx. 4-fold higher emission intensity compared to the CHO analogue (Supplementary Fig. 47a). This difference correlates with its lower photoisomerization efficiency, indicating competition between radiative decay and photochemical isomerization pathways in the excited state. These competing processes are inversely related and are strongly influenced by the electronic nature of the substituents (CN vs. CHO). We further evaluated the reversibility and fatigue resistance of the fluorescence response in solution. The CN based motor shows stable and reversible fluorescence switching under repeated 340nm/415nm irradiation cycles (Figures S47 b, c), demonstrating good photochemical robustness.

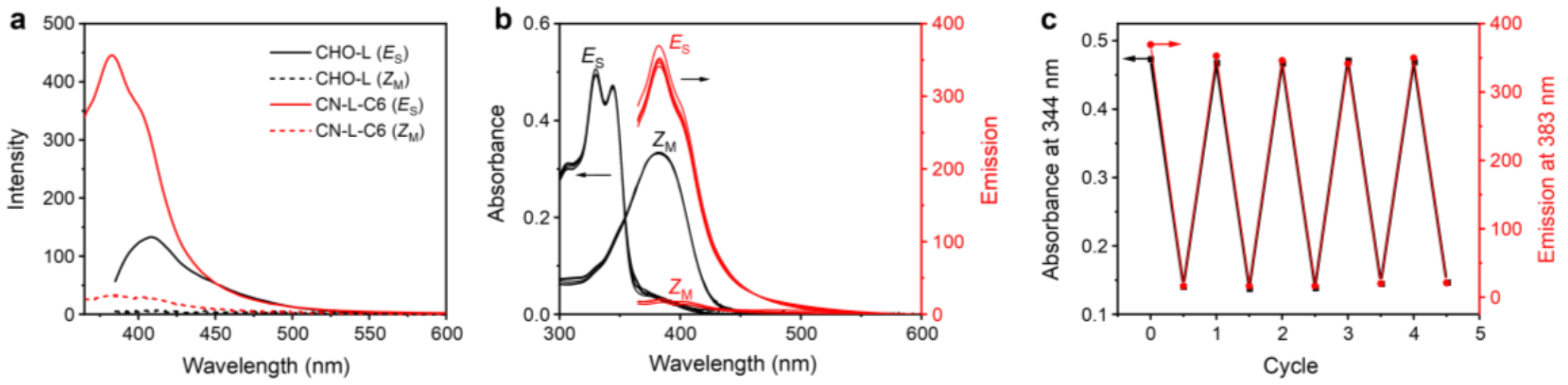

Supplementary Fig. 47 | Fluorescence properties of cyano and aldehyde motros in solution. a Comparison of

fluorescence spectra of **CN-L-C6** and **CHO-L** in DCM solution. **b, c** Fatigue study of **CN-L-C6** upon cyclic irradiation with 340 nm (to produce Z_M) and 415 nm (to produce E_S) light.

Then, we investigated the fluorescence behavior in the polymer networks. Compared to solution, the emission spectra of LCPNs exhibit notable differences, particularly the appearance of a long-wavelength emission band around 480 nm and a relative suppression of the ~380 nm peak (Supplementary Fig. 48a). To clarify this effect, we prepared thin films (~2 µm), in which the short-wavelength emission becomes dominant (Supplementary Fig. 48b). This observation indicates that the apparent spectral shift in thicker films originates primarily from reabsorption (inner filter effects), as supported by the corresponding transmission spectra (Figures S48 a, b).

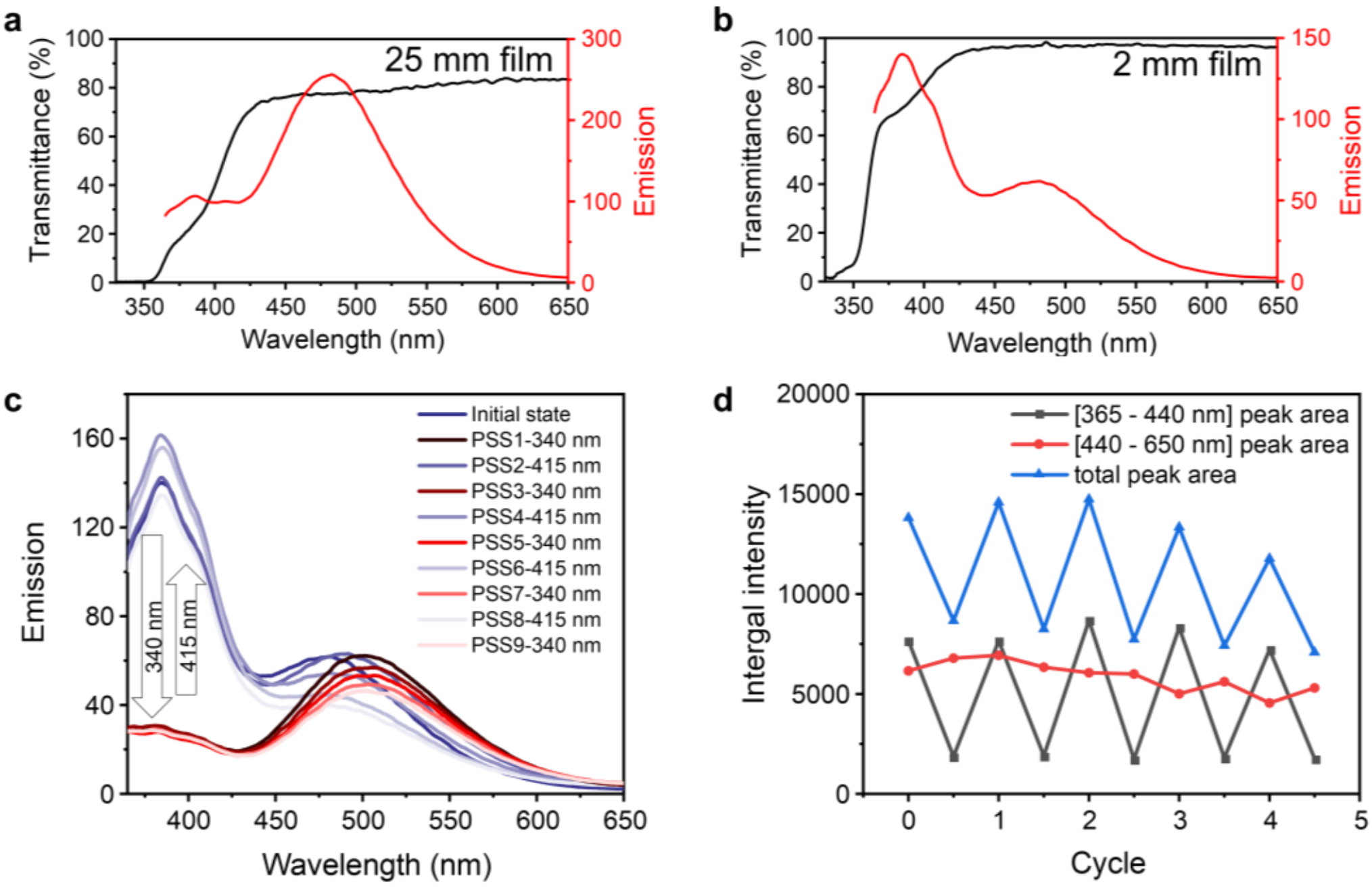

Supplementary Fig. 48 | Fluorescence properties of cyano motor in the network. Transmission and emission spectra of **CN-L-C6** films of 25 µm (**a**) and 2 µm (**b**) in thickness. **c** Fatigue study of 2 µm-thick film of **CN-L-C6** upon sequential irradiation with 340 nm and 415 nm light. **d** Corresponding integral intensities of emission for both peaks.

The origin of the ~480 nm emission band is not fully understood. As this feature is absent in reference networks lacking photoresponsive moieties (which show only autofluorescence around 377 nm; Supplementary Fig. 49b), we attribute it tentatively to specific interactions between the molecular motors and the polymer network environment (e.g., local polarity, aggregation, or constrained conformations).

Importantly, fluorescence switching in the networks is reversible. In 2 µm **CN-L-C6** films, cyclic irradiation (340 nm/415 nm) results in repeatable on/off modulation of the ~380 nm emission (Figures S48 c, d). Quantitative analysis of the integrated emission intensity confirms good reversibility with minimal fatigue for this band, while the longer-wavelength emission shows a slight decrease over repeated cycles.

In thicker films (~25 µm), similar switching behavior is observed (Supplementary Fig. 49a), accompanied by modulation of both emission bands, which accounts for the macroscopic color changes.

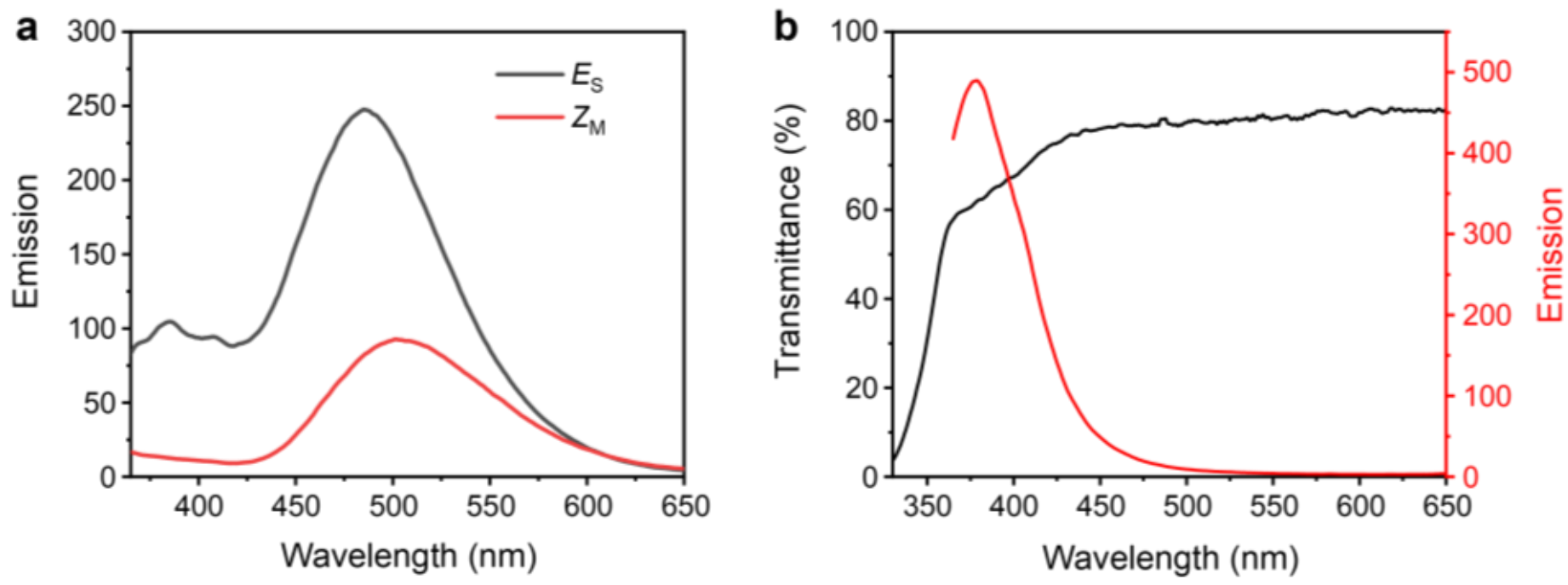

Supplementary Fig. 49 | Fluorescence properties of cyano motor in the network. a Emission spectra of **CN-L-C6** films of 25 μm before and after irradiation to 340 nm UV light. **b** Transmission and emission spectra of 25 μm-thick film of reference LCPN where photoresponsive units were replaced by C6M crosslinker.

Under 340 nm UV irradiation, the fluorescence emission spectra of the 25 μm thick LCPN films were significantly changed (Supplementary Fig. 50), as evidenced by the apparent redshift of the emission peak and the change of fluorescence intensity.

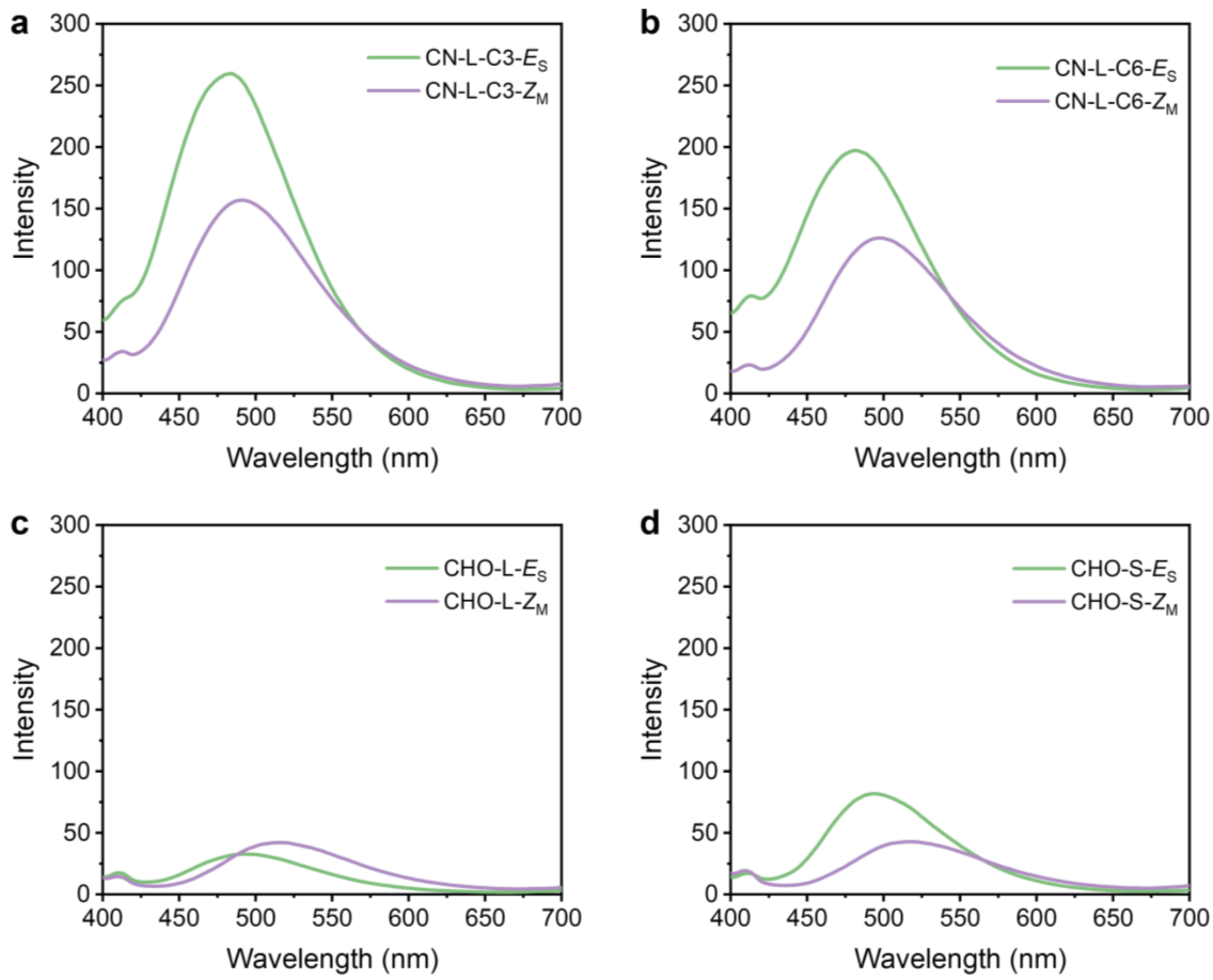

Supplementary Fig. 50 | Fluorescence properties of motorized LCPNs. Fluorescence spectra of different LCPN films (25 μm) before (green curve) and after (purple curve) irradiation under UV light. (**a**) **CN-L-C3**; (**b**) **CN-L-C6**; (**c**) **CHO-L**; (**d**) **CHO-S**.

16 Shape encoding of LCPN ribbons

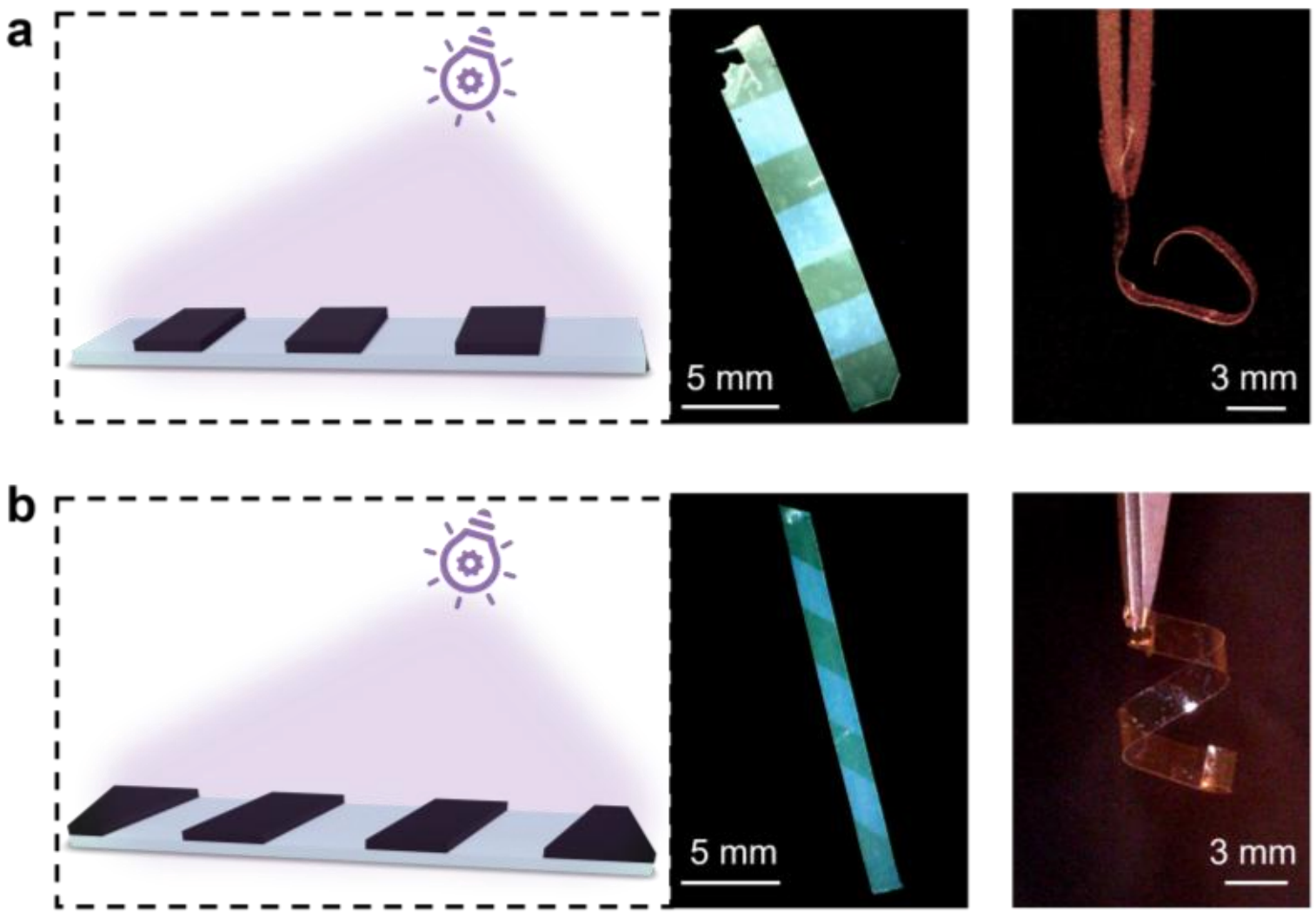

Supplementary Fig. 51 | Shape encoding and fluorescence patterning in the CN-S LCPN film. Shape coding of LCPN ribbons by masking. **a** The stripes of the mask were oriented perpendicular to the LC alignment direction, and the LCPN ribbon was irradiated by 340 nm light on the side covered by the photomask (left panel). The corresponding striped fluorescence pattern can be clearly observed upon UV excitation (middle panel). A bent deformation is gradually formed after detaching the ribbon from the glass substrate (right panel). **b** The mask stripes were aligned at a 45° angle relative to the LC alignment, and the masked side of the LCPN ribbon was exposed to 340 nm light (left panel). Under UV excitation, a fluorescent pattern matching the mask appeared (middle panel). After stripping, the ribbon spontaneously formed a right-handed (RH) helical deformation (right panel).

17 Optimized geometries of cyano and aldehyde motors

Supplementary Table 5 | Computational data. Optimized geometries of E_S-**CHO-L**, E_S-**CN-L-C6**, E_S-**CN-S**, Z_M-**CN-L-C6**, and Z_M-**CN-S**.

E_S-CHO-L			
C	-1.06403	-0.46069	0.16032
C	-1.03585	0.27193	-1.03307
C	-2.42555	0.73051	-1.29716
C	-3.30896	-0.30249	-0.61145
C	-2.47949	-0.60069	0.65957
C	-2.79154	1.96686	-1.66413
C	-1.86511	3.15849	-1.86224
C	-2.48637	4.17829	-0.87766
C	-3.95532	3.84474	-0.94416
C	-4.141	2.56211	-1.47552
C	-5.03471	4.61535	-0.57563
C	-6.32785	4.11134	-0.7439
C	-6.48728	2.86122	-1.33991
C	-5.41824	2.06994	-1.74735
C	0.09601	-0.96628	0.70074

C	1.30839	-0.77048	0.0336
C	1.30304	-0.08524	-1.18245
C	0.14578	0.42728	-1.76005
C	-3.46812	-1.58648	-1.43311
C	-1.91375	3.72169	-3.28635
C	0.19879	1.02404	-3.13804
C	-5.67858	0.79766	-2.50277
C	2.51298	-1.34842	0.66325
C	-7.44632	4.95872	-0.28298
H	-4.28267	0.10985	-0.35503
H	-2.68247	-1.5897	1.07371
H	-2.68775	0.14325	1.43455
H	-0.84085	2.91861	-1.58386
H	-2.2772	5.21324	-1.1534
H	-2.10617	4.01074	0.1347
H	-4.9006	5.60629	-0.1537
H	0.08463	-1.52682	1.63018
H	-2.49457	-2.05471	-1.60107
H	-3.92054	-1.39891	-2.40615
H	-4.0963	-2.29964	-0.89436
H	-1.25019	4.58548	-3.37026
H	-2.92703	4.0505	-3.53161
H	-1.61194	2.98553	-4.03042
H	0.82588	0.41478	-3.79025
H	0.61525	2.03318	-3.13114
H	-0.80064	1.0655	-3.5648
H	-6.45426	0.95919	-3.25228
H	-6.02188	-0.00397	-1.84518
H	-4.7727	0.46713	-3.00536
O	3.63643	-1.35922	0.20615
H	2.31117	-1.80922	1.64775
O	-8.61599	4.64245	-0.22548
H	-7.127	5.9658	0.04301
O	-7.76811	2.39668	-1.61681
C	-8.40722	1.76189	-0.60435
O	-7.87063	1.53252	0.45357
O	2.5031	0.14722	-1.84233
C	3.04338	-0.88932	-2.53012
O	2.43446	-1.91227	-2.73387
C	4.41545	-0.60516	-2.99616
C	5.10813	0.53778	-2.61006
C	5.05104	-1.54529	-3.81325
C	6.4174	0.74948	-3.0182
C	6.34967	-1.34677	-4.22879

C	7.04543	-0.19869	-3.8279
H	4.6297	1.26975	-1.97275
H	4.5151	-2.43713	-4.11389
H	6.93428	1.64263	-2.69746
H	6.85285	-2.06985	-4.85947
C	-9.79228	1.38541	-0.95119
C	-10.34855	1.64297	-2.19992
C	-10.56908	0.75345	0.02529
C	-11.65669	1.27691	-2.48623
C	-11.87002	0.39018	-0.24277
C	-12.42296	0.6466	-1.50422
H	-9.75963	2.13476	-2.96321
H	-10.13865	0.55336	0.99863
H	-12.0641	1.48698	-3.46492
H	-12.48181	-0.09707	0.5069
O	-13.70125	0.24938	-1.67502
C	-14.31496	0.43873	-2.95729
H	-13.72521	-0.08158	-3.71803
H	-14.32934	1.50616	-3.19706
C	-15.71706	-0.12264	-2.87391
H	-16.30031	0.40539	-2.1171
H	-15.69286	-1.18458	-2.62213
H	-16.21522	-0.00695	-3.8384
O	8.3168	-0.09584	-4.26915
C	9.09347	1.03765	-3.85682
H	9.13118	1.06827	-2.76391
H	8.61293	1.95295	-4.21482
C	10.47811	0.88042	-4.44452
H	10.43483	0.84418	-5.53474
H	10.95067	-0.0335	-4.07954
H	11.09671	1.7309	-4.15159
***E*~S~-CN-L-C6**			
C	-1.04764	-0.41406	0.55951
C	-1.0016	0.28258	-0.65675
C	-2.38209	0.74903	-0.94522
C	-3.28353	-0.25854	-0.247
C	-2.47163	-0.52667	1.04331
C	-2.72665	1.98178	-1.34039
C	-1.78962	3.16603	-1.52862
C	-2.43211	4.19473	-0.56526
C	-3.90161	3.87499	-0.67839
C	-4.077	2.58476	-1.20128
C	-4.98048	4.67018	-0.36987

C	-6.26005	4.16079	-0.61468
C	-6.41704	2.90727	-1.19529
C	-5.33762	2.0945	-1.53171
C	0.09739	-0.91376	1.13659
C	1.30485	-0.73896	0.45512
C	1.32476	-0.11479	-0.7885
C	0.17913	0.39816	-1.38787
C	-3.44308	-1.5616	-1.0375
C	-1.80205	3.71943	-2.95712
C	0.24921	0.95875	-2.77935
C	-5.56387	0.81675	-2.28587
H	-4.25716	0.1687	-0.01401
H	-2.68785	-1.50102	1.48424
H	-2.6821	0.24222	1.79274
H	-0.77302	2.92396	-1.22384
H	-2.20539	5.22728	-0.8351
H	-2.08724	4.02389	0.45895
H	-4.86032	5.66535	0.04039
H	0.07865	-1.44029	2.08296
H	-2.47242	-2.0444	-1.17846
H	-3.87786	-1.39268	-2.02196
H	-4.08768	-2.25397	-0.49126
H	-1.13819	4.58394	-3.02928
H	-2.80897	4.04435	-3.23199
H	-1.47892	2.97852	-3.68731
H	0.8719	0.32541	-3.41271
H	0.68286	1.96068	-2.78967
H	-0.7468	1.00756	-3.21348
H	-6.3407	0.95792	-3.03898
H	-5.88576	0.00842	-1.62738
H	-4.64728	0.50958	-2.78499
O	-7.7063	2.51337	-1.52038
C	-8.27376	1.54397	-0.74737
O	-7.70226	1.06436	0.20048
O	2.53713	0.03845	-1.44471
C	3.06022	-1.0605	-2.06111
O	2.5089	-2.13309	-2.03558
C	4.34165	-0.76738	-2.72858
C	4.83416	0.52733	-2.86247
C	5.07649	-1.83695	-3.25095
C	6.03929	0.76647	-3.5062
C	6.2785	-1.6143	-3.88455
C	6.77002	-0.30861	-4.01591
H	4.27249	1.36427	-2.46869

H	4.69454	-2.84504	-3.14675
H	6.39569	1.78191	-3.60336
H	6.86087	-2.43519	-4.28511
C	-9.62836	1.18589	-1.20709
C	-10.25419	1.84005	-2.26425
C	-10.3028	0.15354	-0.54615
C	-11.53253	1.48145	-2.66555
C	-11.57053	-0.2151	-0.93702
C	-12.19663	0.44855	-2.00076
H	-9.74552	2.64244	-2.7825
H	-9.81821	-0.35648	0.27723
H	-11.99669	2.00723	-3.48759
H	-12.10147	-1.01415	-0.43383
O	-13.43796	0.02334	-2.31112
C	-14.13551	0.67588	-3.38314
H	-13.56129	0.56261	-4.30729
H	-14.22472	1.74249	-3.15764
C	-15.49672	0.02807	-3.50255
H	-16.06122	0.14148	-2.57505
H	-15.40151	-1.03543	-3.7296
H	-16.05664	0.50546	-4.30912
O	7.95574	-0.18713	-4.64602
C	8.52657	1.12268	-4.78133
H	8.64961	1.56425	-3.78804
H	7.84618	1.75298	-5.36157
C	9.86006	0.97004	-5.4778
H	9.73215	0.53161	-6.46928
H	10.52908	0.33449	-4.89452
H	10.32561	1.95106	-5.59053
C	-7.42662	4.93675	-0.30391
N	-8.35904	5.56017	-0.04682
C	2.5313	-1.23649	1.00778
N	3.51832	-1.63342	1.44609
Z$\boldsymbol{Z_M}$**-CN-L-C6**			
C	1.27926	-0.02634	5.19417
C	0.72422	0.36346	3.95594
C	1.22612	1.68453	3.59713
C	2.43938	1.96636	4.48846
C	2.17896	1.04904	5.7164
C	0.6947	2.55919	2.69104
C	1.49135	3.58839	1.88197
C	0.63474	3.73392	0.59297
C	-0.74503	3.3445	1.02041

C	-0.69229	2.6475	2.24784
C	-1.93567	3.59411	0.37677
C	-3.12328	3.15117	0.98646
C	-3.07211	2.53667	2.24383
C	-1.88085	2.30175	2.92896
C	0.98335	-1.24011	5.77367
C	0.11478	-2.10766	5.08858
C	-0.36569	-1.75046	3.8215
C	-0.04481	-0.54772	3.19692
C	2.60777	3.41728	4.93253
C	2.92401	3.19592	1.53053
C	-0.46112	-0.33746	1.77369
C	-1.93922	1.79968	4.33794
H	3.35734	1.61362	3.99464
H	3.09883	0.65817	6.16156
H	1.65655	1.62817	6.49162
H	1.48158	4.55997	2.39923
H	1.00464	3.0266	-0.16371
H	0.68277	4.73699	0.15879
H	-1.97486	4.11556	-0.57458
H	1.39621	-1.53199	6.73432
H	1.67368	3.78305	5.37412
H	3.39059	3.47548	5.69683
H	2.8875	4.09222	4.12228
H	2.93862	2.19541	1.08326
H	3.32406	3.90291	0.79535
H	3.59988	3.196	2.38724
H	0.20535	0.37596	1.28384
H	-0.43167	-1.28407	1.22726
H	-1.48311	0.05226	1.70303
H	-2.83421	2.18226	4.83667
H	-1.97858	0.70488	4.38377
H	-1.05508	2.12014	4.89422
O	-1.25308	-2.60629	3.1826
C	-0.74059	-3.72452	2.55772
O	0.44844	-3.96585	2.54587
O	-4.28926	2.24255	2.84568
C	-4.63651	0.90943	2.93851
O	-3.95454	0.0327	2.44866
C	-1.79606	-4.52809	1.93748
C	-1.43255	-5.71306	1.27569
C	-3.14543	-4.1566	1.97555
C	-2.38906	-6.50068	0.67244
C	-4.11737	-4.94017	1.37162

C	-3.74153	-6.12077	0.71481
H	-0.3877	-6.00562	1.24322
H	-3.44542	-3.24417	2.47952
H	-2.11735	-7.41835	0.15972
H	-5.15346	-4.62685	1.41709
C	-5.89042	0.71711	3.66843
C	-6.6216	1.78093	4.20985
C	-6.37148	-0.59386	3.8275
C	-7.80697	1.55482	4.89376
C	-7.54753	-0.83149	4.50473
C	-8.27719	0.24224	5.04353
H	-6.26481	2.79899	4.09759
H	-5.80662	-1.42193	3.41047
H	-8.3522	2.39719	5.30204
H	-7.92712	-1.84067	4.63236
O	-4.60118	-6.95535	0.09539
C	-6.01242	-6.62838	0.10468
H	-6.15667	-5.65229	-0.37662
H	-6.35731	-6.56524	1.14495
C	-6.72986	-7.72348	-0.64781
H	-6.57576	-8.6935	-0.16582
H	-6.37784	-7.78094	-1.68214
H	-7.80279	-7.50916	-0.65914
O	-9.41519	-0.09134	5.68579
C	-10.22193	0.96477	6.26228
H	-10.51991	1.66094	5.46752
H	-9.6219	1.50879	7.00343
C	-11.42396	0.31052	6.90062
H	-11.11706	-0.38691	7.68574
H	-12.01471	-0.23088	6.1557
H	-12.057	1.08133	7.35039
C	-4.37901	3.35008	0.36021
N	-5.40213	3.51314	-0.16137
C	-0.26205	-3.35195	5.65126
N	-0.57436	-4.36968	6.11188
E			
C	-1.12997	-0.56511	0.68503
C	-1.0412	-0.04694	-0.62573
C	-2.38848	0.42672	-1.00165
C	-3.35018	-0.41051	-0.16569
C	-2.55608	-0.51274	1.16552
C	-2.67839	1.61061	-1.59189
C	-1.6696	2.70253	-1.92746

C	-2.27557	3.90585	-1.15336
C	-3.75504	3.6473	-1.26789
C	-3.99035	2.28988	-1.58606
C	-4.79988	4.52316	-1.13572
C	-6.12461	4.06108	-1.3368
C	-6.35646	2.71314	-1.68974
C	-5.27359	1.82103	-1.85987
C	-0.02164	-1.05796	1.33537
C	1.20563	-1.05257	0.64642
C	1.28313	-0.59812	-0.68179
C	0.15564	-0.10408	-1.35376
C	-3.573	-1.8213	-0.7285
C	-1.62083	3.03283	-3.42585
C	0.24066	0.2071	-2.81834
C	-5.54564	0.451	-2.40479
C	2.3796	-1.54811	1.26923
C	-7.13775	5.05002	-1.27785
N	-7.89359	5.93044	-1.23469
N	3.33114	-1.95197	1.79586
O	2.48026	-0.72988	-1.33469
O	-7.56897	2.17117	-1.94895
H	-4.30483	0.10258	-0.02283
H	-2.83543	-1.38665	1.7631
H	-2.72102	0.38705	1.77386
H	-0.67282	2.45281	-1.55379
H	-1.97822	4.87294	-1.57218
H	-1.95972	3.87466	-0.10176
H	-4.63814	5.57146	-0.9019
H	-0.07824	-1.45528	2.34449
H	-2.62453	-2.3701	-0.75903
H	-3.98358	-1.80556	-1.74039
H	-4.26438	-2.37503	-0.08463
H	-0.8742	3.81135	-3.61507
H	-2.59484	3.40819	-3.7607
H	-1.37169	2.15974	-4.03319
H	0.92836	-0.48731	-3.30915
H	0.61117	1.21963	-3.01187
H	-0.7468	0.11978	-3.27763
H	-6.22286	0.51174	-3.26199
H	-6.02501	-0.2009	-1.66522
H	-4.61288	-0.01386	-2.72657
C	-8.77379	2.68923	-1.32801
H	-8.53173	3.03486	-0.31535
H	-9.15182	3.53277	-1.91478

C	-9.77512	1.55754	-1.29738
H	-9.4035	0.72399	-0.69413
H	-9.98489	1.19874	-2.30964
H	-10.71112	1.91748	-0.85887
C	3.26335	0.49598	-1.39644
H	2.65562	1.29098	-1.84758
H	3.5212	0.79331	-0.37042
C	4.49633	0.20905	-2.2175
H	4.22392	-0.08519	-3.23582
H	5.08684	-0.59207	-1.76231
H	5.11652	1.10913	-2.27066
**Z_M-CN-S**			
C	1.60708	-0.18258	5.22928
C	1.02268	0.14481	3.98424
C	1.31594	1.53866	3.67042
C	2.44404	1.984	4.60866
C	2.27356	1.02138	5.81551
C	0.70071	2.34545	2.7527
C	1.38942	3.48742	1.99567
C	0.56323	3.56311	0.68282
C	-0.77327	3.00844	1.06106
C	-0.67311	2.27594	2.26536
C	-1.96751	3.14436	0.39251
C	-3.11653	2.56567	0.96152
C	-3.04629	1.91851	2.20952
C	-1.83385	1.77089	2.89556
C	1.54687	-1.4552	5.74681
C	0.89342	-2.44569	4.99093
C	0.37259	-2.1483	3.71939
C	0.44852	-0.8594	3.17263
C	2.39338	3.43788	5.06998
C	2.8698	3.28427	1.68443
C	0.03092	-0.65108	1.7499
C	-1.84846	1.15084	4.25838
C	0.78216	-3.76971	5.48454
C	-4.35573	2.62768	0.27715
N	-5.3606	2.67572	-0.30141
N	0.69367	-4.84568	5.91007
O	-0.15032	-3.18699	2.99409
O	-4.19141	1.38744	2.7451
H	3.42036	1.76779	4.14868
H	3.21928	0.79126	6.31531
H	1.61145	1.4924	6.55701
H	1.24374	4.43568	2.53515

H	1.03591	2.91965	-0.0738
H	0.50947	4.57487	0.26976
H	-2.037	3.67879	-0.55028
H	1.98938	-1.70451	6.70665
H	1.39551	3.67405	5.45722
H	3.11596	3.58727	5.88005
H	2.63063	4.15497	4.28251
H	3.02459	2.30161	1.22415
H	3.19893	4.04891	0.97201
H	3.51517	3.3546	2.56155
H	0.65547	0.11514	1.28089
H	0.11963	-1.58767	1.19405
H	-1.00736	-0.30855	1.66423
H	-2.8038	1.33039	4.75474
H	-1.7069	0.06358	4.20885
H	-1.03723	1.55242	4.8716
C	-5.06138	2.36123	3.39273
H	-4.50377	2.84156	4.20833
H	-5.34363	3.13262	2.6645
C	-6.27119	1.6207	3.90618
H	-5.97816	0.85015	4.62599
H	-6.81479	1.14848	3.08197
H	-6.94337	2.32467	4.40637
C	-1.60509	-3.23126	2.98469
H	-1.95178	-3.39575	4.01453
H	-1.99575	-2.26622	2.63613
C	-2.02295	-4.35713	2.07111
H	-1.61793	-5.31163	2.42121
H	-1.67401	-4.17676	1.04955
H	-3.1149	-4.42825	2.05717

Supplementary Reference

[1] Sheng, J. et al. Formylation boosts the performance of light-driven overcrowded alkene-derived rotary molecular motors. *Nat. Chem.* **16** 1330-1338 (2024).

[2] Chen, S. H., Chen, H. M. P., University of Rochester. Glassy chiral-nematic liquid crystals and optical devices containing same. *U.S. Patent* **7**,001,648 (2006).

[3] Long, G. Photoresponsive Liquid Crystal Polymer Materials Based on Molecular Motors, Chapter 4. (PhD thesis, University of Groningen, 2025).

[4] Hou, J. et al. Phototriggered complex motion by programmable construction of light-driven molecular motors in liquid crystal networks. *J. Am. Chem. Soc.* **144**, 6851-6860 (2022).

[5] Stranius, K., Börjesson, K. Determining the photoisomerization quantum yield of photoswitchable molecules in solution and in the solid state. *Sci. Rep.* **7**, 41145 (2017).

[6] Kuhn, H. J., Braslavsky, S. E., Schmidt, R. Chemical actinometry (IUPAC Technical Report). *Pure Appl. Chem.* **76**, 2105-2146 (2004).

[7] Montalti, M., Credi, A., Prodi, L., Gandolfi, M. T. Handbook of Photochemistry. (CRC Press,

2006).

[8] Grimme, S., Hansen, A., Ehlert, S., Mewes, J. M. r2SCAN-3c: A “Swiss army knife” composite electronic-structure method. *J. Chem. Phys.* **154** (2021).

[9] Barone, V., Cossi, M. Quantum calculation of molecular energies and energy gradients in solution by a conductor solvent model. *J. Phys. Chem. A* **102**, 1995-2001 (1998).

[10] Najibi, A., Goerigk, L. DFT-D4 counterparts of leading meta-generalized-gradient approximation and hybrid density functionals for energetics and geometries. *J. Comput. Chem.* **41**, 2562-2572 (2020).

[11] Neese, F., Wennmohs, F., Becker, U., Riplinger, C. The ORCA quantum chemistry program package. *J. Chem. Phys.* **152** (2020).

[12] Perrot, A., Wang, W.-z., Buhler, E., Moulin, E., Giuseppone, N. Bending Actuation of Hydrogels through Rotation of Light-Driven Molecular Motors. *Angew. Chem. Int. Ed.* **62**, e202300263 (2023).

[13] Yao, X. et al. Scalable Approach to Molecular Motor-Polymer Conjugates for Light-Driven Artificial Muscles. *Adv. Mater.* **36**, 2403514 (2024).

[14] Chen, J. et al. Artificial muscle-like function from hierarchical supramolecular assembly of photoresponsive molecular motors. *Nat. Chem.* **10**, 132-138 (2018).

[15] Ryabchun, A. et al. Macroscopic motion from synchronized molecular power strokes. *Chem* **9**, 3544-3554 (2023).
