# Supplementary material for: Efficient molecular motors in liquid crystal networks enable the integration of fluorescence with large opto-mechanical effects
Source: Nat Commun. 2026 Jul 30;17:9241. doi: 10.1038/s41467-026-76177-0 (PMC13527073; doi:10.1038/s41467-026-76177-0)
Supplement: Supplementary file 2 [file 41467_2026_76177_MOESM2_ESM.pdf]

Description of Additional Supplementary Files

File Name: Supplementary Movie 1
Description: Comparative study of the photo-driven deformation of motorized networks under 340 nm light irradiation (motor-based films shown in the front view are, from left to right: **CHO-L**, **CHO-S**, **CN-L-C3**, **CN-L-C6**, and **CN-S**).

File Name: Supplementary Movie 2
Description: Comparative study of the photo-driven deformation of networks containing different photoresponsive units under 365 nm light irradiation (the photoresponsive units of the films shown in the front view are, from left to right: **CHO-L**, **CHO-S**, **CN-L-C3**, **CN-L-C6**, **CN-S**, **Azo** and **MSG**).

File Name: Supplementary Movie 3
Description: Deformation behaviors of the **CN-S** motor based LCPN films (with the mask stripe direction perpendicular to the LC alignment direction) after irradiation with 340 nm light under different light sources.

File Name: Supplementary Movie 4
Description: Deformation behaviors of the **CN-S** motor based LCPN films (with the mask stripe direction oriented at 45° to the LC alignment direction) after exposure to 340 nm light under different light sources.
