# Supplementary material for: Efficient molecular motors in liquid crystal networks enable the integration of fluorescence with large opto-mechanical effects
Source: Nat Commun. 2026 Jul 30;17:9241. doi: 10.1038/s41467-026-76177-0 (PMC13527073; doi:10.1038/s41467-026-76177-0)
Supplement: Supplementary file 3 [file 41467_2026_76177_MOESM7_ESM.pdf]

nature portfolio

Peer Review File

Efficient Molecular Motors in Liquid Crystal Networks Enable the Integration of Fluorescence with Large Opto-mechanical Effects

Corresponding Author: Professor Ben Feringa

This file contains all reviewer reports in order by version, followed by all author rebuttals in order by version.

Version 0:

Reviewer comments:

Reviewer #1

(Remarks to the Author)
This manuscript reports light-driven liquid crystal polymer networks incorporating structurally optimized molecular motors, aiming to achieve enhanced macroscopic actuation force together with fluorescence-assisted optical readout. The experimental work is extensive and technically competent. The work is experimentally comprehensive and conceptually sound, and the discovery and utilization of intrinsic motor fluorescence coupled with photoactuation adds a compelling multifunctional dimension. Overall, the study is original and of high relevance to the fields of soft actuators, responsive materials, and molecular machines. However, several important issues must be addressed before publication.
1. A key premise of the manuscript is that the reported force enhancement arises predominantly from photochemical motor activity rather than photothermal effects. The current analysis, however, is indirect and insufficient to support this distinction at a general-interest journal level. To rigorously support this claim, the following would be required: quantitative temperature mapping under illumination for samples of different thicknesses, alignments, and motor concentrations (e.g., IR thermography or embedded micro-sensors).
2. The manuscript attributes enhanced force generation primarily to structural optimization of the molecular motors. However, the intermediate steps linking molecular structure to network-level mechanics are not sufficiently demonstrated. A convincing causal framework would require: quantitative measurements of network orientation and order (e.g., WAXS/SAXS-derived order parameters) before and after illumination; Stress-strain and relaxation analyses to clarify whether force enhancement originates from elastic, viscoelastic, or irreversible network responses.
3. The manuscript emphasizes force enhancements of one to several orders of magnitude, yet these claims are supported primarily by representative examples. To meet the standards of Nature Communications, the following would be necessary: long-term cycling and fatigue tests to assess durability and reproducibility; statistical analysis across multiple independent samples ($n \geq 3$-5) with appropriate error bars.
4. While the fluorescence response adds an interesting multifunctional aspect, it is presented largely descriptively. A more compelling integration would require: quantitative correlation between fluorescence signals and actuation strain or stress under identical conditions; demonstration of reversibility and cycling stability of the fluorescence response; clarification of the physical origin of the fluorescence changes (e.g., motor conformation vs. local environment vs. network rearrangement), potentially through spectral or lifetime analysis.
5. The patterned deformation and fluorescence encoding demonstrations are visually appealing but remain illustrative. To substantiate broader relevance, the study would need: scaling experiments with larger sample dimensions or different geometries. Moreover, the demonstrated actuation is limited to simple bending, and the lack of more complex or functionally relevant actuation modes weakens the claimed application potential.
6. Mechanistic explanation of rigid-core enhancement needs stronger support. The claim that rigid core extension leads to larger geometrical changes and enhanced actuation is plausible but currently qualitative. Additional support is recommended, such as: schematic or computational illustration of ES/ZM geometrical differences, literature precedents, or clearer acknowledgment of this as a working hypothesis.

7. Fluorescence mechanism requires deeper discussion. Since fluorescence is a key functional output, the manuscript should discuss the origin of emission (intrinsic motor vs. aggregation/network effects), reversibility over multiple switching cycles, and the relation between motor isomeric states and emission behavior.

8. In line 289, the statement "the MSG sample exhibits the weakest photo-generated stress and modulus, with the photothermal effect accounting for up to 48%" is inconsistent with the corresponding data in Figure 3E.

9. It would be informative if the manuscript discussed or provided data on the mechanical recovery performance of LCPNs with different molecular motors under 415 nm blue light irradiation (e.g., recovery rate and recovery efficiency), to better illustrate the reversibility of the actuation behavior.
10. The intrinsic mechanism behind the opposite-direction actuation observed under 415 nm irradiation in Figure 4C is not clearly explained.

Reviewer #2

(Remarks to the Author)
In this manuscript entitled "Efficient Molecular Motors in Liquid Crystal Networks: Integrating Fluorescence with Large Opto-mechanical Effects", the authors design and synthesize a set of tunable molecular motor units with modified substituents and tailored rigidity. These units effectively regulate both the mechanical properties and photo-responsive behaviors of liquid crystal polymer networks. Comparative analysis with traditional azobenzene and advanced molecular motors reveals clear correlations between molecular structure, macroscopic actuation performance, and material stiffness. The work offers valuable insights for future soft actuators and adaptive photonic systems. Nevertheless, several important points need further explanation and experimental support prior to publication. I thus suggest a minor revision based on the comments below.
1.In this study, the authors synthesized two first-generation molecular motors functionalized with strong electron-withdrawing groups (cyano and aldehyde), both of which enhance the photoresponsive performance. However, the motor bearing the aldehyde group exhibited notably longer half-life and higher quantum yields compared to the cyano-substituted analog. Given that both substituents are electron-withdrawing, what explains this distinct difference in photophysical behavior?
2.Throughout the experiment, efforts were made to mitigate photothermal effects. It would be helpful to clarify whether these thermal effects originate primarily from the irradiation source or from the photoswitches themselves. Additionally, how was the light intensity optimized, and what is the relationship between light intensity and the resulting thermal load?
3.The mechanical properties of the liquid crystal polymer network are significantly influenced by photothermal effects. To what extent do thermal contributions affect key network properties such as modulus, actuation strain, and response kinetics?
4.For the actuation tests in Figure 3, a film size of 1 cm*1 mm was used. How would varying the aspect ratio—either decreasing or increasing it—affect the response speed and magnitude of actuation? Given that sample dimensions often play a critical role in macroscopic deformation, further discussion on size effects would be valuable.
5.The films were pre-stretched by 1% prior to testing. What is the rationale for selecting this specific pre-strain level? Further justification and explanation regarding the determination of this 1% pre-stretch would strengthen the methodological clarity of the study.

Reviewer #3

(Remarks to the Author)
The manuscript "Efficient Molecular Motors in Liquid Crystal Networks: Integrating Fluorescence with Large Opto-mechanical Effects" by Ryabchun, Feringa, and co-authors describes an integration of liquid crystal polymer network with diverse molecular motor-containing photoswitchable motifs.
In previous literature, such systems were already demonstrated (which is properly acknowldedged in references). However, the novelty of the current approach is the comparison between diverse photochromic units, and indication of structural factors that enhance the efficiency of light energy conversion into mechanical force and macroscopic effects. Here is, where the current manuscript increases the general understanding, overcomes earlier reports which described single systems, and offers design principles, which altogether constitute its significance to the field.
The experiments properly support the conclusions and claims, the methodology is sound, and the experimental data complete and reproducible.

I would gladly recommend publication of the manuscript, after the following comments are addressed:
1) in the description of LCPN preparation (line 114, page 4): "In order to reduce the elastic modulus of the LCPN, a non-polymerizable low molar mass LC mixture (E7) was also added" - could the authors clarify for non-experts, why does that additive reduce the elastic modulus (or, if this is a purely empirical observation, maybe a reference to the original sources of this observation can be added).
2) in table 1, (line 193, page 6), and in the text, the motors decorated with aldehydes ("CHO") have consistently higher quantum yields of photoisomerization, than the motors decorated with cyano groups ("CN"). Was that phenomenon already known before for such types of a motor? If so, could you indicate explicitly in which references is it discussed? If not, then could you add a broader discussion on what might be the reasons for such a considerable difference?
3) in line 275 (page 8) - a typo: "previously" instead of "previous"
4) in lines 280-285 - the authors notice that the photogenerated force of LCPNs generated with CN-motors is higher than that with CHO-motors, even though the CHO-motors have significantly higher quantum yield. Their argument is the larger dipole moment of the -CN group versus -CHO and its role in increasing coupling with other components of the network. While the difference in dipole moments in favor of the -CN group is a fact, I am not convinced of the relation that the authors propose - I don't understand what exact interactions should better transfer the light-induced force from the CN-motor to the surrounding network, than from the CHO-motor. Is this supported by any observable or calculation? Or could it be empirically proven, e.g. by measuring the photogenerated forces in the LCPNs doped with more (or less) of the E7 (an additive which is claimed to

influence the elastic modulus/stiffness of the material)? If the LCPNs are more stiff (e.g. without E7), than the advantage of the CN-motors should be more visible. And if the LCPNs are less stiff (e.g. with more E7 as originally), then the advantage of CN-motors should be lost in favor of CHO-motors which convert light into movement more efficiently (Q.Y. from the Table 1) than the CN-motors at the single molecule level.
5) line 346 (page 11) - the authors notice that the cyano and aldehyde motors in LCPN films exhibit "noticeable fluorescence". Are they also fluorescent in homogenous solutions? If not, where does the fluorescence come from in the LC polymer network? Are the motors so sterically confined?

Version 1:

Reviewer comments:

Reviewer #1

(Remarks to the Author)
This manuscript presents molecular-motor-based liquid crystal polymer networks with tunable substituents, rigid-core extension, and spacer length, and examines how these parameters affect actuation, photogenerated stress, modulus, and fluorescence. The comparative design is meaningful, and the integration of photomechanical response with fluorescence is potentially interesting for responsive soft materials. The revised manuscript has added more data,However, several central conclusions are currently stronger than the evidence supports. I therefore recommend major revision.
1. The manuscript argues that force generation is mainly photochemical in origin, but this distinction is not yet established rigorously enough. The added thermal imaging is helpful, yet the current analysis still does not fully separate photochemical and photothermal contributions across the different systems. The corresponding claims should be better supported or stated more cautiously.
2. The proposed link between molecular design, alignment, stiffness, and force generation is plausible, but still only partly demonstrated. The added dichroism data improve the argument, although the evidence remains somewhat indirect and limited in scope. Stronger network-level support would be needed for the broader mechanistic claims.
3. The normalization of force to individual molecular units appears too assumption-dependent for the strong comparative conclusions currently made. These values should be presented more clearly as approximate estimates, and the claims of orders-of-magnitude superiority should be moderated.
4. The fluorescence data are interesting, but this part remains more descriptive than mechanistic. In particular, the origin of the longer-wavelength emission in the networks is still not fully resolved, and fluorescence-actuation coupling is not yet quantitatively established. This section should be strengthened or interpreted more conservatively.
5. The comparison with azobenzene and the second-generation motor is informative, but it should be interpreted more carefully because the systems are tested under different excitation wavelengths. As such, the current data do not fully support broad claims of general superiority.
6. The manuscript attributes the performance differences mainly to motor structure, but the influence of network composition is not fully separated. A small control study varying E7 content or motor loading in representative CN- and CHO-based networks would strengthen this conclusion.

Reviewer #2

(Remarks to the Author)
The work is revised accordingly and ready for publication.

Reviewer #3

(Remarks to the Author)
The authors have sufficiently addressed all the issues raised in my first review. The responses to other reviewers' comments are also convincing. Therefore I recommend publication of the revised manuscript in the current shape - without further corrections nor comments from my side.

Version 2:

Reviewer comments:

Reviewer #1

(Remarks to the Author)
The authors has respond the comments carefully. I would recommend its acceptance as it.

Open Access This Peer Review File is licensed under a Creative Commons Attribution 4.0 International License, which permits use, sharing, adaptation, distribution and reproduction in any medium or format, as long as you give appropriate credit to the original author(s) and the source, provide a link to the Creative Commons license, and indicate if changes were made.
In cases where reviewers are anonymous, credit should be given to 'Anonymous Referee' and the source.
The images or other third party material in this Peer Review File are included in the article's Creative Commons license, unless indicated otherwise in a credit line to the material. If material is not included in the article's Creative Commons license and your intended use is not permitted by statutory regulation or exceeds the permitted use, you will need to obtain permission directly from the copyright holder.
To view a copy of this license, visit https://creativecommons.org/licenses/by/4.0/

Detailed Response to Reviewer Comments

"Efficient Molecular Motors in Liquid Crystal Networks: Integrating Fluorescence with Large Opto-mechanical Effects"

Research Article, No. NCOMMS-25-103033

We thank the reviewers for the scholarly evaluation and valuable comments regarding our manuscript, which we have improved according to their suggestions, as detailed in the following. All significant changes in the main text as well as the SI are highlighted in yellow.

Reviewer #1:

This manuscript reports light-driven liquid crystal polymer networks incorporating structurally optimized molecular motors, aiming to achieve enhanced macroscopic actuation force together with fluorescence-assisted optical readout. The experimental work is extensive and technically competent. The work is experimentally comprehensive and conceptually sound, and the discovery and utilization of intrinsic motor fluorescence coupled with photoactuation adds a compelling multifunctional dimension. Overall, the study is original and of high relevance to the fields of soft actuators, responsive materials, and molecular machines. However, several important issues must be addressed before publication.

Answer: We thank reviewer for the careful reading of our manuscript and high evaluation of the novelty and data quality and for all useful suggestion which will undoubtedly improve the manuscript in the revised version.

Comment 1. A key premise of the manuscript is that the reported force enhancement arises predominantly from photochemical motor activity rather than photothermal effects. The current analysis, however, is indirect and insufficient to support this distinction at a general-interest journal level. To rigorously support this claim, the following would be required: quantitative temperature mapping under illumination for samples of different thicknesses, alignments, and motor concentrations (e.g., IR thermography or embedded micro-sensors).

Answer: Following the reviewer's suggestion, we performed temperature mapping of the LCPNs at the light intensities used throughout this study. As the sample thickness, molecular alignment, and concentration of photoactive moieties were not varied, all investigated materials were subjected to IR thermal imaging under identical conditions.

As shown in Fig. R1, neither irradiation at 2.2 mW/cm^2 (used for bending experiments) nor at 13.4 mW/cm^2 (used for photomechanical characterization) results in a significant temperature increase. At the highest UV intensity, a minor temperature rise in the range of 0.5-1.7 °C was observed for LCPNs containing photoactive moieties, while the reference LCPN lacking photoresponsive groups exhibited a comparable increase of approximately 0.4 °C.

In contrast, upon irradiation at substantially higher light intensity (as employed for back-switching of the CN-L-C6 film), a pronounced temperature increase of approximately 25 °C was detected. This thermal effect is consistent with the rapid stress increase observed in the corresponding mechanical response (Fig. 3c), indicating a thermally driven contribution under these conditions.

Additional evidence supporting the photochemical origin of the actuation at low light intensities is provided in Fig. R9. Specifically, the CHO-L LCPN ribbon exhibits comparable actuation behavior when irradiated under cold water (5 °C), where thermal effects are effectively suppressed.

This data has been additionally highlighted in the revised version of the manuscript (please see page 8) and added to the SI (Supplementary Fig. 46).

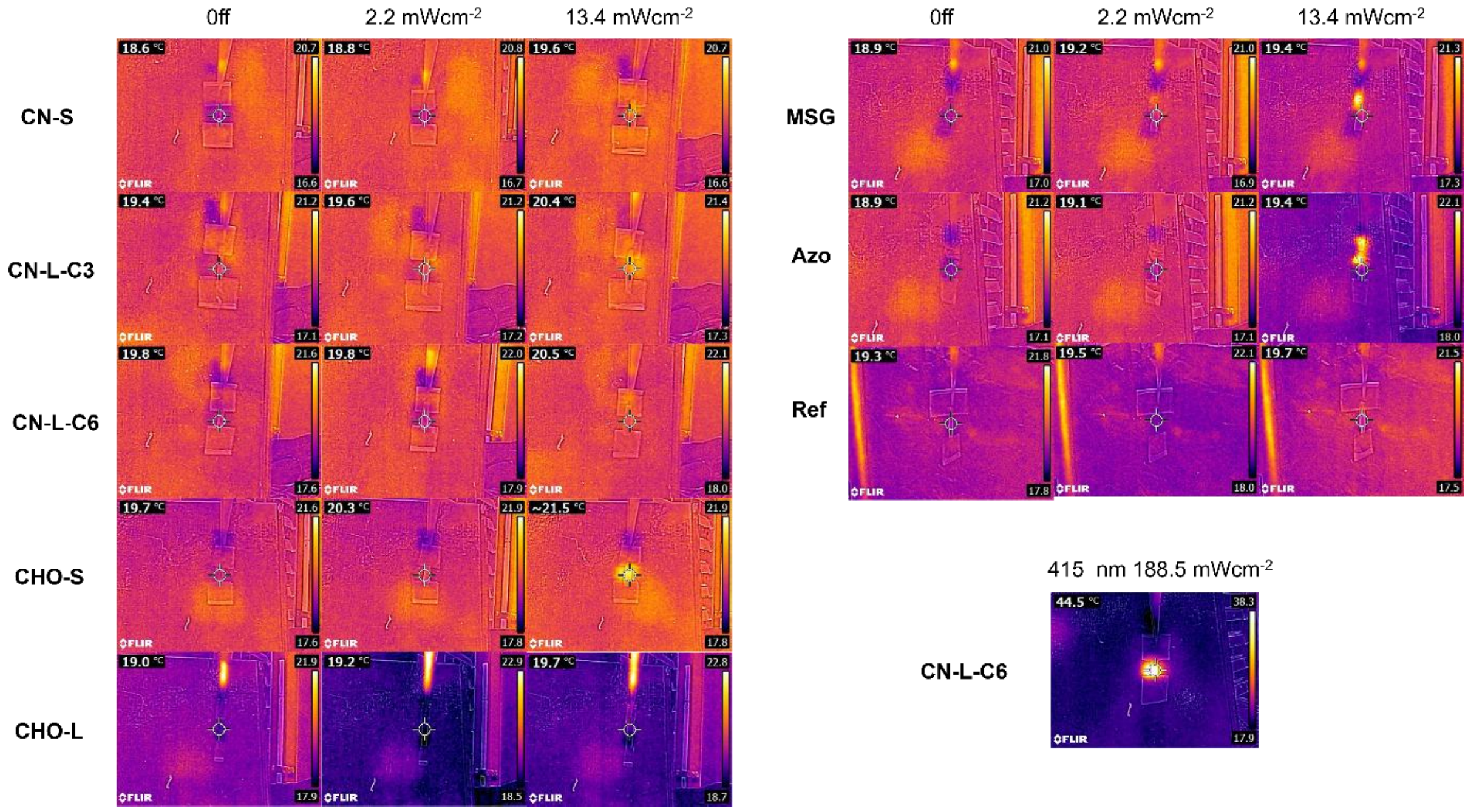

Fig. R1. IR imaging of the LCPN ribbons subjected to UV irradiation. Light intensity is shown on the top. All cyano-motors-continuing films were irradiated with 340 nm, while the others with 365 nm UV light. In the reference LCPN (Ref) photoactive molecules were replaced by C6M crosslinker. Bottom right panel: IR image of CN-L-C6 film under high-intensity blue (415 nm) light.

Comment 2. The manuscript attributes enhanced force generation primarily to structural optimization of the molecular motors. However, the intermediate steps linking molecular structure to network-level mechanics are not sufficiently demonstrated. A convincing causal framework would require: quantitative measurements of network orientation and order (e.g., WAXS/SAXS-derived order parameters) before and after illumination; Stress-strain and relaxation analyses to clarify whether force enhancement originates from elastic, viscoelastic, or irreversible network responses.

Answer: We appreciate the referee for the comment. In the present work, our primary objective is to demonstrate how the operation of molecular motors generates mechanical forces and to compare the force output across different motor designs. To isolate the effect of molecular structure, we employed a consistent materials platform based on well-established liquid

crystalline polymer networks (LCPNs), whose mechanical behavior has been extensively characterized in prior studies. [1,2,3]

Nevertheless, following the reviewer's suggestion, we have performed additional characterization to further elucidate the structure-property relationships in these systems.

First, we investigated the thermal and phase behavior of the networks (Fig. R2). The glass transition temperatures of all materials were found to lie in the range of 8.9 - 14.8 °C, confirming that the networks are in the rubbery state under the conditions of all mechanical experiments (room temperature). Moreover, no evidence of isotropization was observed, consistent with the highly crosslinked nature of the networks. This excludes the possibility of phase-transition-driven contributions to the observed actuation upon irradiation.

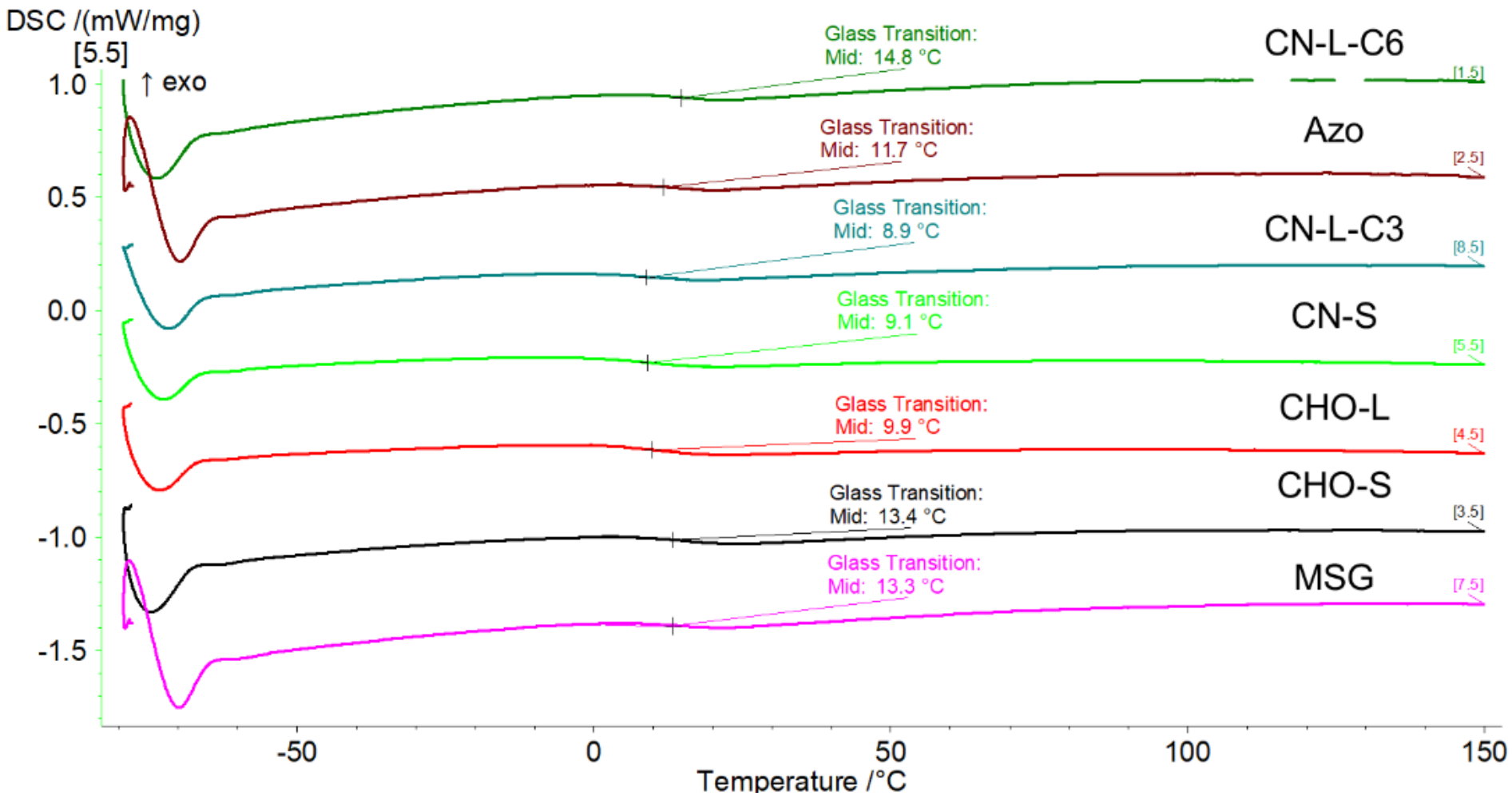

Fig. R2. DSC curves of all LCPNs studied in the work. Glass transition temperatures are indicated in the graph.

Second, we quantified the molecular order within the networks using a dichroic probe (Disperse Red 1, DR1) combined with polarized UV-Vis spectroscopy (Fig. R3). Thin films (10 μm) of CN-L-C6 and CHO-L networks doped with ~0.5 wt% DR1 were prepared in cells promoting unidirectional alignment. Polarized absorbance spectra were recorded as a function of polarization angle relative to the alignment direction (defined by the rubbing of the polyimide aligning layers). The degree of order was quantified via the linear dichroism parameter:

$$D=(A_{\parallel}-A_{\perp})/(A_{\parallel}+A_{\perp}),$$

where $A_{\parallel}$ and $A_{\perp}$ are the absorbances measured parallel and perpendicular to the director, respectively. In this definition, D = 0 corresponds to an isotropic state, while D = 1 indicates perfect alignment.

---

[1] Hikmet, R. A. M., and D. J. Broer. "Dynamic mechanical properties of anisotropic networks formed by liquid crystalline acrylates." *Polymer* 32.9 (1991): 1627-1632.

[2] White, Timothy J., and Dirk J. Broer. "Programmable and adaptive mechanics with liquid crystal polymer networks and elastomers." *Nature materials* 14.11 (2015): 1087-1098.

[3] Liu, Danqing, and Dirk J. Broer. "Liquid crystal polymer networks: preparation, properties, and applications of films with patterned molecular alignment." *Langmuir* 30.45 (2014): 13499-13509.

Both networks exhibit a high degree of molecular order. Notably, the CN-L-C6 system shows slightly higher *D* values, suggesting improved alignment, which we attribute to the specific structure of the cyano-substituted motor core that perturbs the liquid crystalline order to a lesser extent.

Importantly, UV irradiation does not result in any measurable change in the dichroism parameter (Fig. R3), indicating that the overall liquid crystalline order remains unchanged during actuation. This observation rules out mechanisms involving large-scale reorganization of the network and supports a model in which actuation arises from local, directional motion of the molecular motors.

These results collectively support the conclusion that the observed shape change and force generation originate from the rotational motion of the molecular motors, which exert forces on the polymer network strands, rather than from changes in network morphology or phase behavior.

Finally, we emphasize that the enhancement in force output correlates with the molecular design of the motors, while the polymer network architecture remains constant across the studied series. All observed photomechanical responses are fully reversible, further supporting a predominantly photochemical (rather than irreversible or viscoelastic) origin.

The corresponding data and discussion have been incorporated into the revised manuscript (page 7 and 8) and SI (Supplementary Fig. 39 and 44).

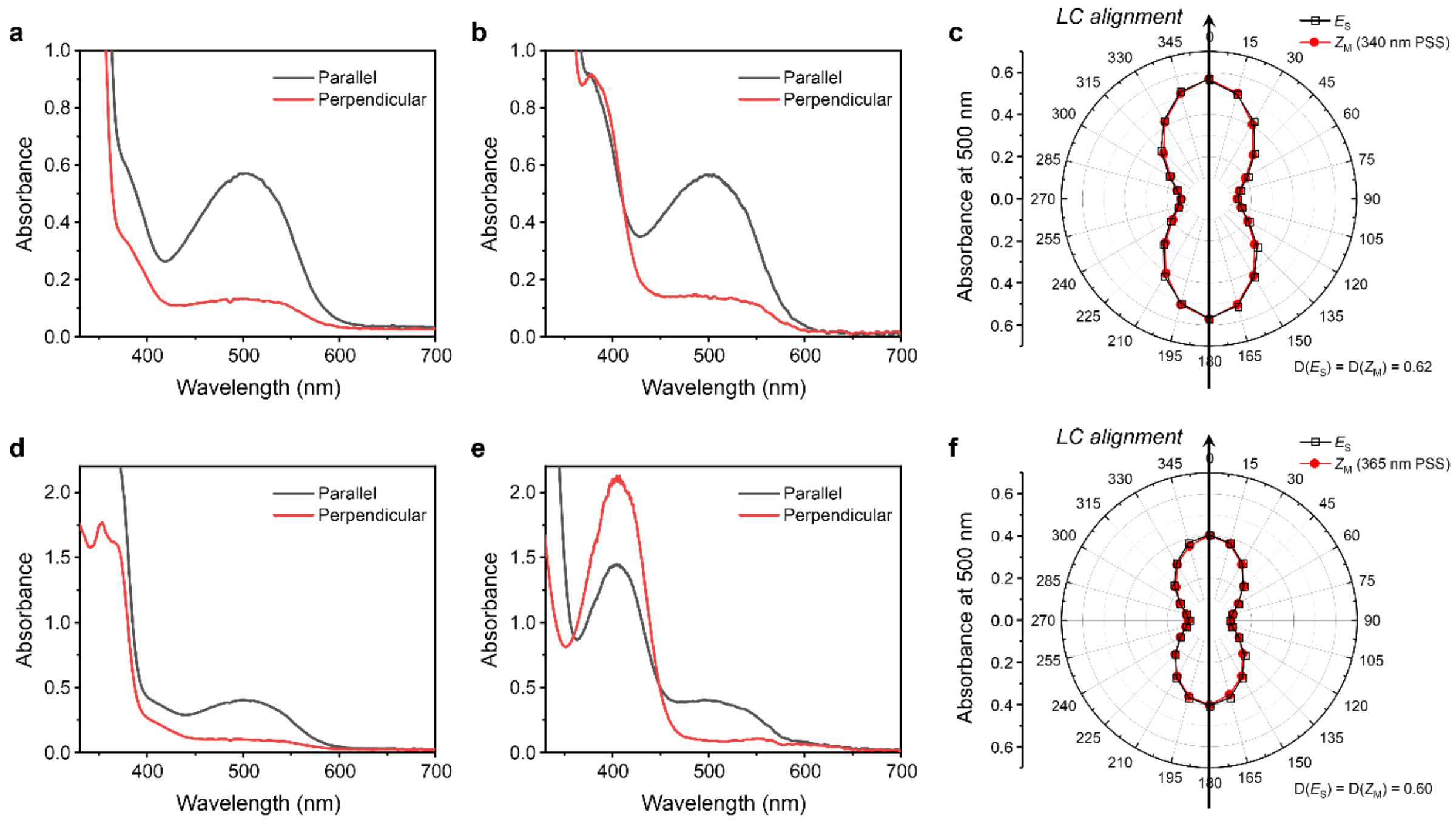

Fig. R3. Polarized light absorbance spectra and corresponding polar plots of CN-L-C6 (**a, b, c**) and CHO-L (**d, e, f**) films doped with DR1 dye before and exposure to UV light. Parallel and Perpendicular denote to the absorbance components polarized in respect to the LC alignment. Dichroism values (*D*) are provided in the plots **c** and **f**.

Comment 3 and 9. The manuscript emphasizes force enhancements of one to several orders of magnitude, yet these claims are supported primarily by representative examples. To meet the standards of Nature Communications, the following would be necessary: long-term cycling

and fatigue tests to assess durability and reproducibility; statistical analysis across multiple independent samples (n ⩾ 3-5) with appropriate error bars.

It would be informative if the manuscript discussed or provided data on the mechanical recovery performance of LCPNs with different molecular motors under 415 nm blue light irradiation (e.g., recovery rate and recovery efficiency), to better illustrate the reversibility of the actuation behavior.

Answer: We would like to emphasize that the central aim of this study is a comparative evaluation of force generation in materials incorporating different molecular motors. To ensure that the observed differences can be directly attributed to molecular design, all polymer networks were synthesized, aligned, and processed using identical protocols, thereby minimizing variations in network structure and macroscopic properties.

We would like to stress that we do not extrapolate our findings beyond the specific systems investigated. The comparison presented in Fig. 3F combines force data reported for motorized synthetic materials in the literature and is included solely to provide context and a concise overview of the current state of the field.

Regarding statistical analysis, all mechanical measurements were performed on at least three samples, as indicated in the manuscript. The reported trends were consistently reproduced across these replicates.

Concerning long-term cycling and fatigue behavior, we agree that such analysis is relevant for evaluating durability. However, this aspect lies beyond the main scope of the present work, which focuses on establishing structure-property relationships between molecular motor design and force generation. Nevertheless, within the experimental timeframe, the photomechanical response was reproducible over multiple actuation cycles, with no observable degradation. As the actuation is driven by photochemical isomerization of the molecular motors, the recovery and efficiency are governed by the intrinsic photophysical properties of the motors. This relationship is reflected in the bending dynamics shown in Fig. 3A, where faster actuation is observed for motors with higher quantum yields of isomerization (Table 1). Due to the high efficiency and near-complete conversion of the photoisomerization process for cyano and aldehyde motors, the actuation is inherently reversible. This is evidenced by the cyclic mechanical response shown in Fig. 3C, demonstrating repeatable forward and backward isomerization.

To further support this point, we performed additional cyclic irradiation experiments using alternating UV and blue light on CHO-L (Fig. R4a) and CN-L-C6 (Fig. R4b) polymer ribbons. As shown in Fig. R4, both systems exhibit consistent and reversible bending behavior over repeated cycles, confirming efficient mechanical recovery.

These additional results and the corresponding discussion have been incorporated into the revised manuscript (page 7) and Supporting Information (Supplementary Fig. 41).

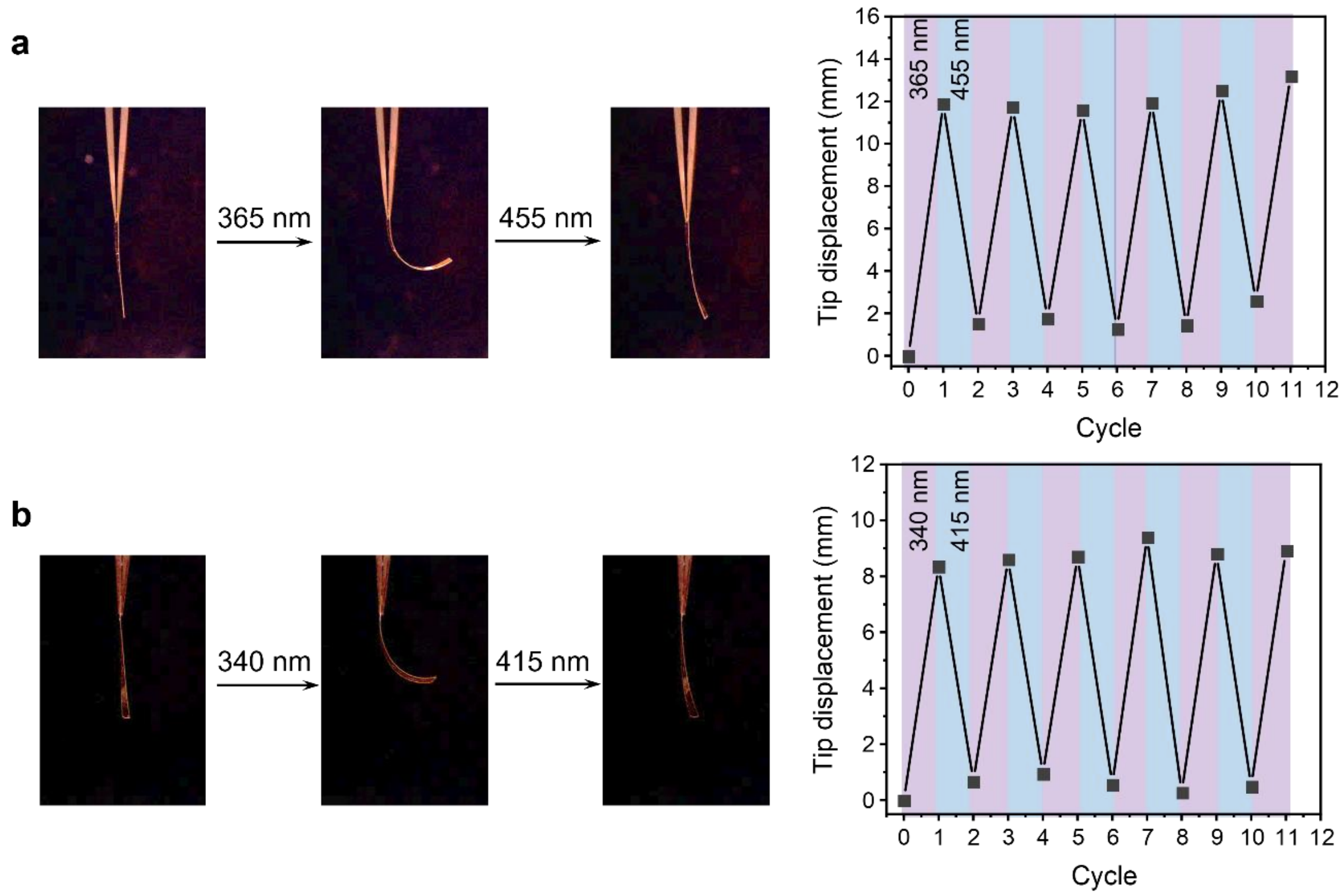

Fig. R4. Reversible bending behaviour of the CHO-L (**a**) and CN-L-C6 (**b**) polymer ribbons upon UV/blue light irradiation. Molecular alignment is parallel to the long axis of the ribbon. The light source is on the right.

Comment 4 and 7: While the fluorescence response adds an interesting multifunctional aspect, it is presented largely descriptively. A more compelling integration would require: quantitative correlation between fluorescence signals and actuation strain or stress under identical conditions; demonstration of reversibility and cycling stability of the fluorescence response; clarification of the physical origin of the fluorescence changes (e.g., motor conformation vs. local environment vs. network rearrangement), potentially through spectral or lifetime analysis.

Fluorescence mechanism requires deeper discussion. Since fluorescence is a key functional output, the manuscript should discuss the origin of emission (intrinsic motor vs. aggregation/network effects), reversibility over multiple switching cycles, and the relation between motor isomeric states and emission behavior.

Answer: We thank the reviewer for these valuable comments and agree that an additional description of the fluorescence response would be beneficial. Accordingly, we have expanded and revised the discussion.

To elucidate the origin of fluorescence, we first performed studies of the molecular motors in solution. As shown in Fig. R5 a, b, both CN-L-C6 and CHO-L motors exhibit pronounced fluorescence in their E_S states, with emission maxima centered at 383 nm and 410 nm, respectively. In contrast, the corresponding Z_M states are nearly non-emissive. The emission profiles closely follow the absorption spectra, confirming that fluorescence originates from the intrinsic electronic structure of the motors.

Quantitatively, the CN-substituted motor exhibits approx. 4-fold higher emission intensity compared to the CHO analogue (Fig. R5c). This difference correlates with its lower photoisomerization efficiency, indicating competition between radiative decay and photochemical isomerization pathways in the excited state. These competing processes are inversely related and are strongly influenced by the electronic nature of the substituents (CN vs. CHO) (please also see page 12 of this response).

We further evaluated the reversibility and fatigue resistance of the fluorescence response in solution. The CN-motor shows stable and reversible fluorescence switching under repeated 340nm/415nm irradiation cycles (Fig. R5 d, e), demonstrating good photochemical robustness.

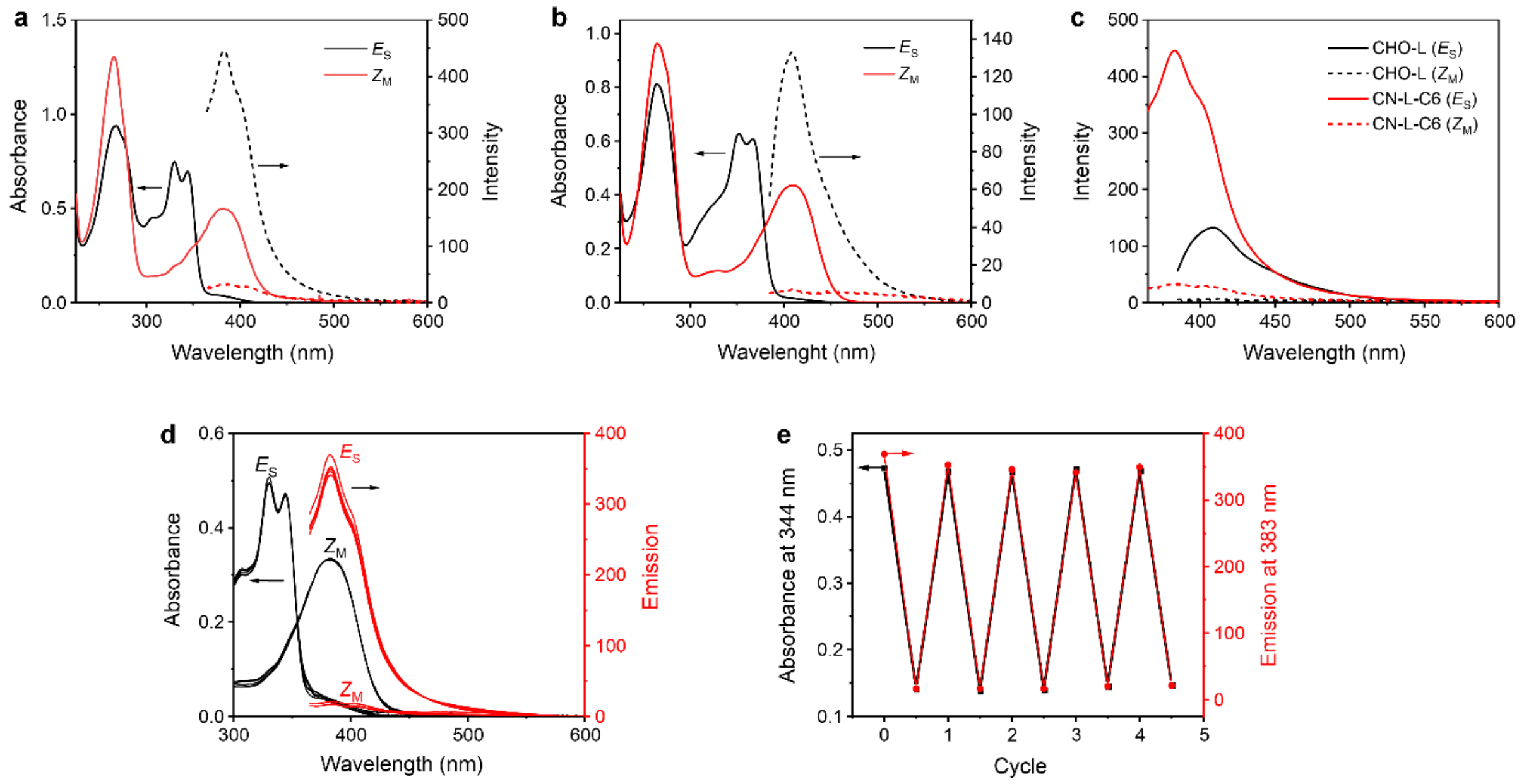

Fig. R5. Absorbance and emission spectra of CN-L-C6 (**a**) and CHO-L (**b**) in E_S and Z_M states. **c)** Comparison of fluorescence spectra of CN-L-C6 and CHO-L. **d, e** Fatigue study of CN-L-C6 upon cyclic irradiation with 340 nm (to produce Z_M) and 415 nm (to produce E_S) light. Measurements were performed in DCM solution.

Then, we investigated the fluorescence behavior in the polymer networks. Compared to solution, the emission spectra of LCPNs exhibit notable differences, particularly the appearance of a long-wavelength emission band around 480 nm and a relative suppression of the ~380 nm peak (Fig. R6a). To clarify this effect, we prepared thin films (~2 μm), in which the short-wavelength emission becomes dominant (Fig. R6b). This observation indicates that the apparent spectral shift in thicker films originates primarily from reabsorption (inner filter effects), as supported by the corresponding transmission spectra (Fig. R6 a, b).

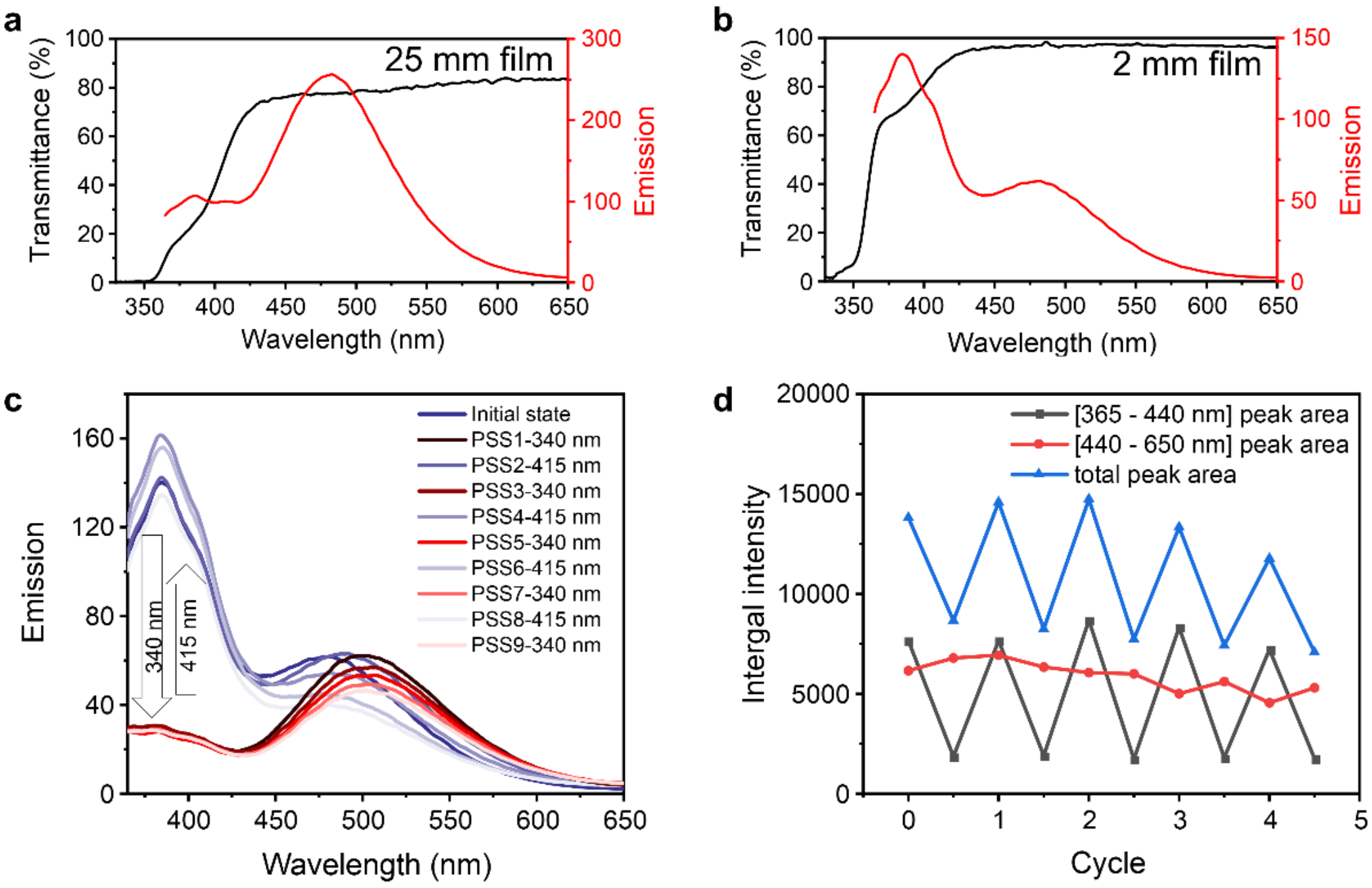

Fig. R6. Transmission and emission spectra of CN-L-C6 films of 25 µm (**a**) and 2 µm (**b**) in thickness. **c** Fatigue study of 2 µm-thick film of CN-L-C6 upon sequential irradiation with 340 nm and 415 nm light. **d** Corresponding integral intensities of emission for both peaks.

The origin of the ~480 nm emission band is not fully understood. As this feature is absent in reference networks lacking photoresponsive moieties (which show only autofluorescence around 377 nm; Fig. R7b), we attribute it tentatively to specific interactions between the molecular motors and the polymer network environment (e.g., local polarity, aggregation, or constrained conformations). A detailed investigation of this effect, including advanced spectroscopic and lifetime analysis, goes beyond the scope of the present study but represents an important direction for our future work.

Importantly, fluorescence switching in the networks is reversible. In 2 µm CN-L-C6 film, cyclic irradiation (340 nm / 415 nm) results in repeatable on/off modulation of the ~380 nm emission (Fig. R6 c, d). Quantitative analysis of the integrated emission intensity confirms good reversibility with minimal fatigue for this band, while the longer-wavelength emission shows a slight decrease over repeated cycles. In thicker films (~25 µm), similar switching behavior is observed (Fig. R7a), accompanied by modulation of both emission bands, which accounts for the macroscopic color changes.

The new data and the expanded discussion have been incorporated into the Supporting Information (Supplementary Fig. 47-49) and the revised manuscript (page 12-13, and revised Figure 4).

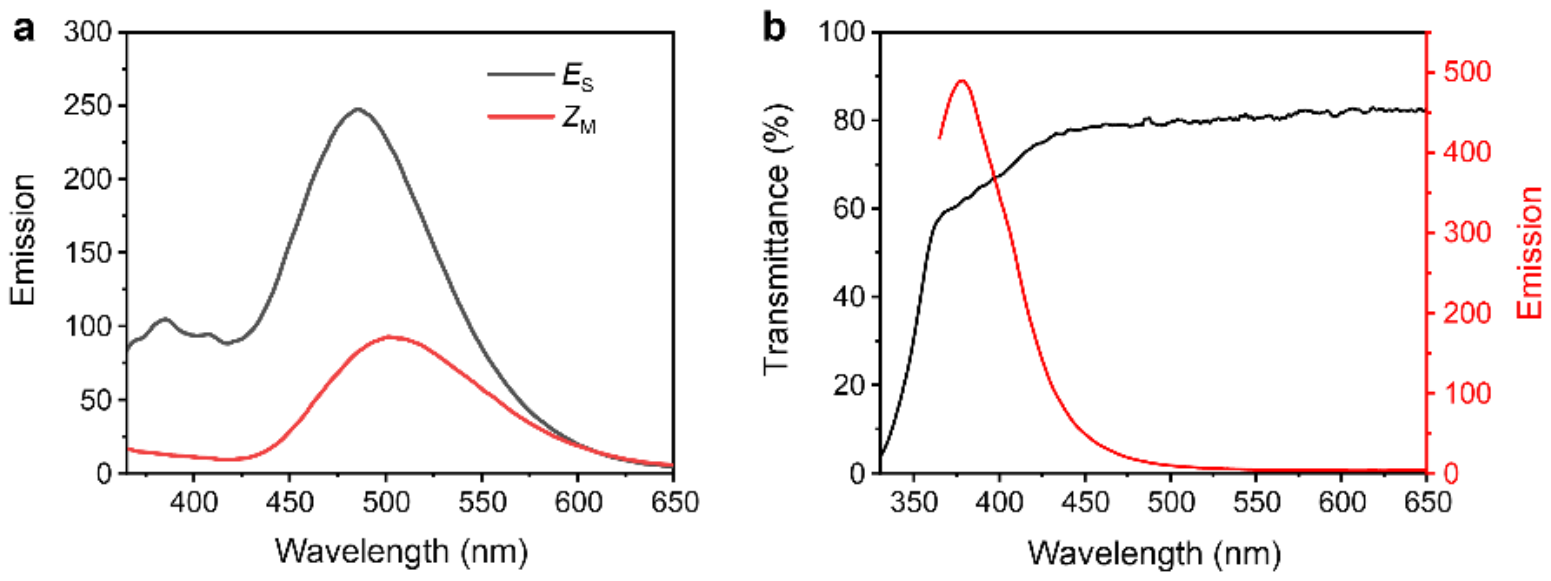

Fig. R7. a Emission spectra of CN-L-C6 films of 25 μm before and after irradiation to 340 nm UV light. **b** Transmission and emission spectra of 25 μm-thick film of reference LCPN where photoresponsive units were replaced by C6M crosslinker.

Comment 5. The patterned deformation and fluorescence encoding demonstrations are visually appealing but remain illustrative. To substantiate broader relevance, the study would need: scaling experiments with larger sample dimensions or different geometries. Moreover, the demonstrated actuation is limited to simple bending, and the lack of more complex or functionally relevant actuation modes weakens the claimed application potential.

Answer: This experiment services as proof of the parental of combining actuation and fluorescence properties by using single molecular scaffold. We believe that the provided experiments clear demonstrate that, while further extension of this concept goes beyond the scope of the current work and will be addressed in our follow up research.

Regarding the range of actuation modes, we would like to note that the system is not limited to simple bending. In addition to bending deformations, we also demonstrate reversible winding and unwinding behavior (Fig. 4f and Supplementary Fig. 51), indicating that the material is capable of more complex, torsional actuation modes.

Comment 6. Mechanistic explanation of rigid-core enhancement needs stronger support. The claim that rigid core extension leads to larger geometrical changes and enhanced actuation is plausible but currently qualitative. Additional support is recommended, such as: schematic or computational illustration of ES/ZM geometrical differences, literature precedents, or clearer acknowledgment of this as a working hypothesis.

Answer: We thank the reviewer for this suggestion and have performed additional computational analysis to provide stronger support for the role of rigid-core extension in enhancing actuation. To quantify the geometrical changes, we carried out DFT geometry optimizations of the E_S and Z_M states of CN-S and CN-L motors (Fig. R8). Calculations were performed using the r²SCAN-3c composite method with the CPCM solvent model (acetonitrile), employing simplified ethoxy substituents for computational efficiency.

In case of the short-core CN-S motor (Fig. R8a), photoisomerization induces a molecular contraction of approximately 4.36 Å, measured as the distance between lateral oxygen atoms. In contrast, the long-core CN-L motor (Fig. R8b) exhibits a substantially larger contraction of ~11.37 Å. A comparable trend is expected for the aldehyde-substituted motors (CHO-S and CHO-L). These results indicate that extending the rigid core roughly doubles the molecular shape change upon photoisomerization, which provides a possible mechanistic basis for the observed increase in photogenerated force (as summarized in Table 2 of the main text).

The computational methodology and discussion have been added to the Supporting Information (Section 11, Supplementary Fig. 43) and highlighted in the revised main text (page 7).

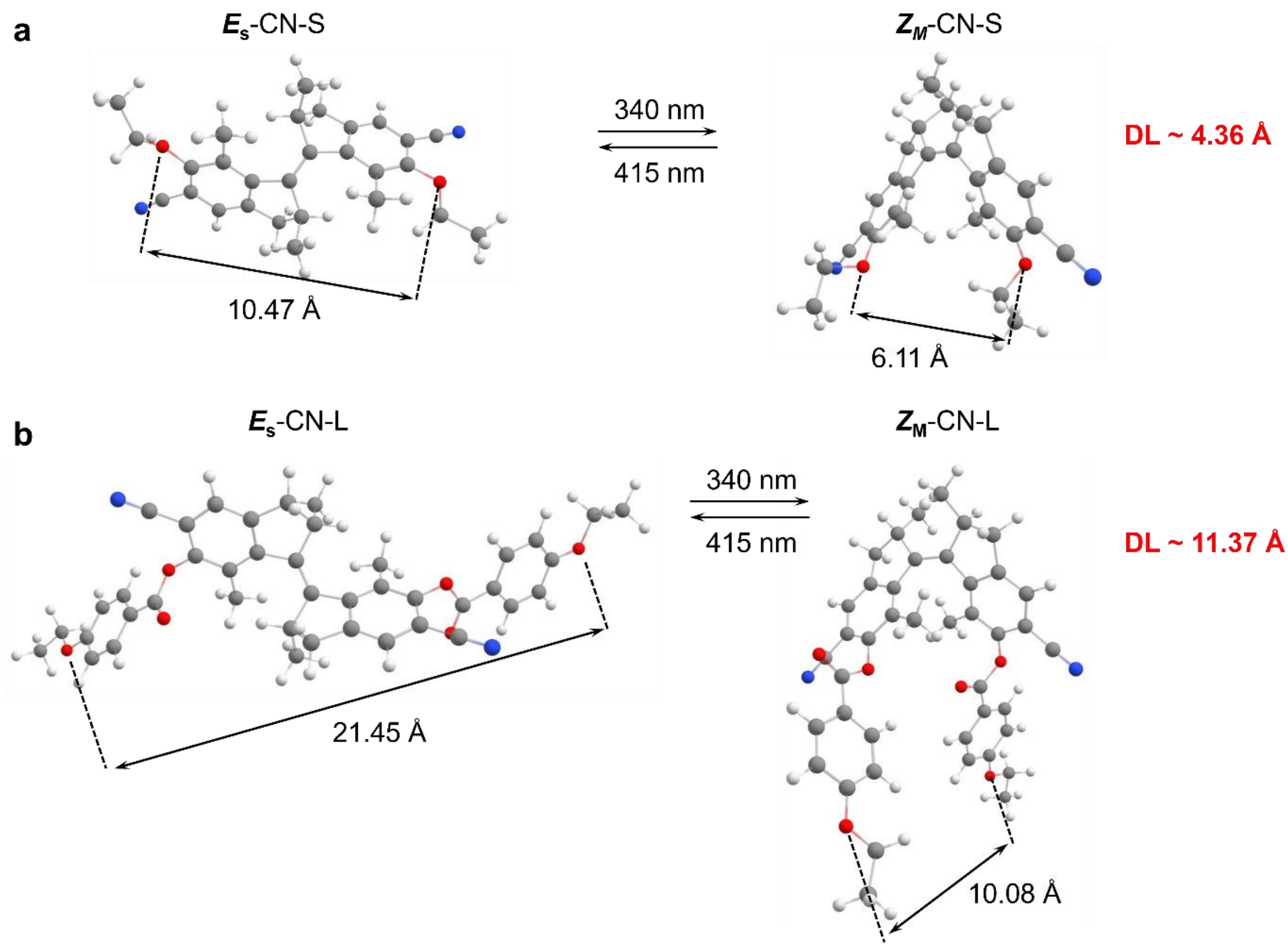

Fig. R8. DFT-optimized structure for the lowest-energy conformer of E_S-CN-S, Z_M-CN-S, E_S-CN-L, and Z_M-CN-L. To simplify calculations long aliphatic spacers and acrylic terminal fragments were replaced by ethoxy-substituents. The length of the rigid core was estimated as the distance between lateral oxygen atoms. Final geometry optimization: r^2scan-3c/ CPCM (ACN).

Comment 8. In line 289, the statement "the MSG sample exhibits the weakest photo-generated stress and modulus, with the photothermal effect accounting for up to 48%" is inconsistent with the corresponding data in Figure 3E.

Answer: We apologies for the type, it has been corrected as 42% in the revised manuscript.

Comment 10. The intrinsic mechanism behind the opposite-direction actuation observed under 415 nm irradiation in Figure 4C is not clearly explained.

Answer: The ribbon presented in Fig. 4f (formerly Fig. 4c) contains patterned areas where molecular motors in E_S (non-irradiated areas) and in Z_M state (irradiated areas). When such ribbon is exposed to UV light it bends toward the light source due to contracting forces building up in non-irradiated areas where E_S-to-Z_M transformation takes place. However, if the ribbon is exposed to blue light, it bends away from the light sources due to pushing forces building up in the irradiated areas where Z_M-to-E_S transformation occurs, while non-irradiated areas are "silent" since they E_S state does not absorb blue light. We additionally clarified this behaviour in the revised version of the manuscript (page 14).

Reviewer #2:

In this manuscript entitled "Efficient Molecular Motors in Liquid Crystal Networks: Integrating Fluorescence with Large Opto-mechanical Effects", the authors design and synthesize a set of tunable molecular motor units with modified substituents and tailored rigidity. These units effectively regulate both the mechanical properties and photo-responsive behaviors of liquid crystal polymer networks. Comparative analysis with traditional azobenzene and advanced molecular motors reveals clear correlations between molecular structure, macroscopic actuation performance, and material stiffness. The work offers valuable insights for future soft actuators and adaptive photonic systems. Nevertheless, several important points need further explanation and experimental support prior to publication. I thus suggest a minor revision based on the comments below.

Answer: We thank reviewer for high evaluation of our work and for valuable suggestions.

Comment 1. In this study, the authors synthesized two first-generation molecular motors functionalized with strong electron-withdrawing groups (cyano and aldehyde), both of which enhance the photoresponsive performance. However, the motor bearing the aldehyde group exhibited notably longer half-life and higher quantum yields compared to the cyano-substituted analog. Given that both substituents are electron-withdrawing, what explains this distinct difference in photophysical behavior?

Answer: We appreciate referee for pointing this out. While both cyano and aldehyde groups are electron-withdrawing, their influence on the excited-state landscape of the motors differs significantly. Experimentally, we observe that the aldehyde-functionalized motors exhibit a higher quantum yield of photoisomerization, near-quantitative photoconversion, and a more pronounced red-shift in absorption. In contrast, the cyano-motors show a lower quantum yield of isomerization, less red-shifted absorption, but enhanced photoluminescence and, importantly, a stronger optomechanical response.

These differences cannot be explained solely by the electron-withdrawing strength of the substituents and instead reflect their distinct electronic and photophysical characteristics. The aldehyde group can participate in extended conjugation with the motor core and introduces low-lying $n \rightarrow \pi^*$ states (dark states), which may facilitate access to non-radiative pathways and/or triplet manifolds. Such states are known to enhance photoisomerization efficiency by promoting population of reactive excited states, as also suggested in our previous work (Nat. Chem. 2024, 16(8), 1330-1338). In contrast, the cyano group is a more linear and predominantly π-accepting substituent, which primarily supports $\pi \rightarrow \pi^*$ transitions. This results in a comparatively "cleaner" excited-state landscape with fewer competing non-radiative pathways, thereby favouring radiative decay and leading to enhanced fluorescence. The higher emission efficiency is consistent with the reduced quantum yield of isomerization, reflecting competition between radiative relaxation and photochemical conversion.

These observations suggest that the CHO and CN substituents direct the excited-state dynamics along different pathways: the aldehyde group promotes efficient photochemical isomerization via additional non-radiative channels, whereas the cyano group favors emissive decay and limits access to reactive states.

We would like to note that a full mechanistic elucidation of these effects would require detailed spectroscopic and theoretical analysis, which lies beyond the scope of the present study. Nevertheless, we have incorporated this mechanistic rationale into the revised manuscript (page 9) to clarify the origin of the observed differences.

Comment 2. Throughout the experiment, efforts were made to mitigate photothermal effects. It would be helpful to clarify whether these thermal effects originate primarily from the irradiation source or from the photoswitches themselves. Additionally, how was the light intensity optimized, and what is the relationship between light intensity and the resulting thermal load?

Answer: To address this, we performed additional temperature mapping of the LCPNs using an IR camera (Fig. R1). As shown, irradiation at 2.2 $mWcm^2$ (used for bending experiments) and 13.4 $mWcm^2$ (used for photomechanical studies) produces almost negligible temperature increase. At the highest UV intensity, we observed a temperature rise of 0.5–1.7 °C in LCPNs containing photoactive moieties, while a reference network lacking photoswitches exhibited a similar increase of 0.4 °C. These results indicate that under standard experimental conditions, thermal effects from the motor molecules themselves are minimal, and most of the observed photomechanical response arises from photochemical isomerization.

The UV light intensity of 13.4 $mWcm^2$ was specifically chosen as a balance between activating a maximal fraction of motors and minimizing thermal contributions (typically below 15%), allowing the onset of actuation to be clearly resolved in the stress curves. Further increases in light intensity lead to larger temperature rises. For example, during back-switching of CN-L-C6 films under high-intensity blue light, we observed a substantial temperature increase of ~25 °C, which coincides with the sharp jump in recorded stress (Fig. 3c). To isolate the photochemical contribution to the generated stress, we calculated the difference between the total recorded stress and the photothermal contribution (Fig. 3c and 3e). Finally, an additional control experiment under cold water (5 °C) demonstrated efficient shape-shifting in response to UV light, further confirming that actuation is photochemically, rather than thermally, driven (Fig. R9).

These results and the corresponding discussion have been incorporated into the main text (page 8) and Supporting Information (Supplementary Fig. 42 and 46).

Comment 3. The mechanical properties of the liquid crystal polymer network are significantly influenced by photothermal effects. To what extent do thermal contributions affect key network properties such as modulus, actuation strain, and response kinetics?

Answer: As reported in our IR measurements, the maximum temperature increase under the experimental light intensities does not exceed 1.7 °C. Given that all tested LCPNs are in the rubbery state at room temperature (glass transition temperatures ranging from 8.9 to 14.8 °C; Fig. R2), such small thermal deviations are not expected to significantly affect the network's modulus, actuation strain, or overall mechanical integrity.

Nevertheless, we agree that the response kinetics may depend on the viscoelastic properties of the network. To investigate this, we performed actuation experiments with CHO-L polymer ribbons under water at different temperatures (20, 10, and 5 °C). In all cases, the ribbons actuated toward the light source (Fig. R9). However, the kinetics were temperature-dependent. Lower temperatures led to slower bending, consistent with the increased elastic modulus and viscoelastic damping at lower temperatures. Importantly, actuation still occurs even at temperatures below the glass transition, demonstrating that the photochemically generated forces from the motors are sufficient to overcome the network stiffness and drive deformation.

This information has been added to the main text (page 7-8) and to SI (Supplementary Fig. 39 and 42).

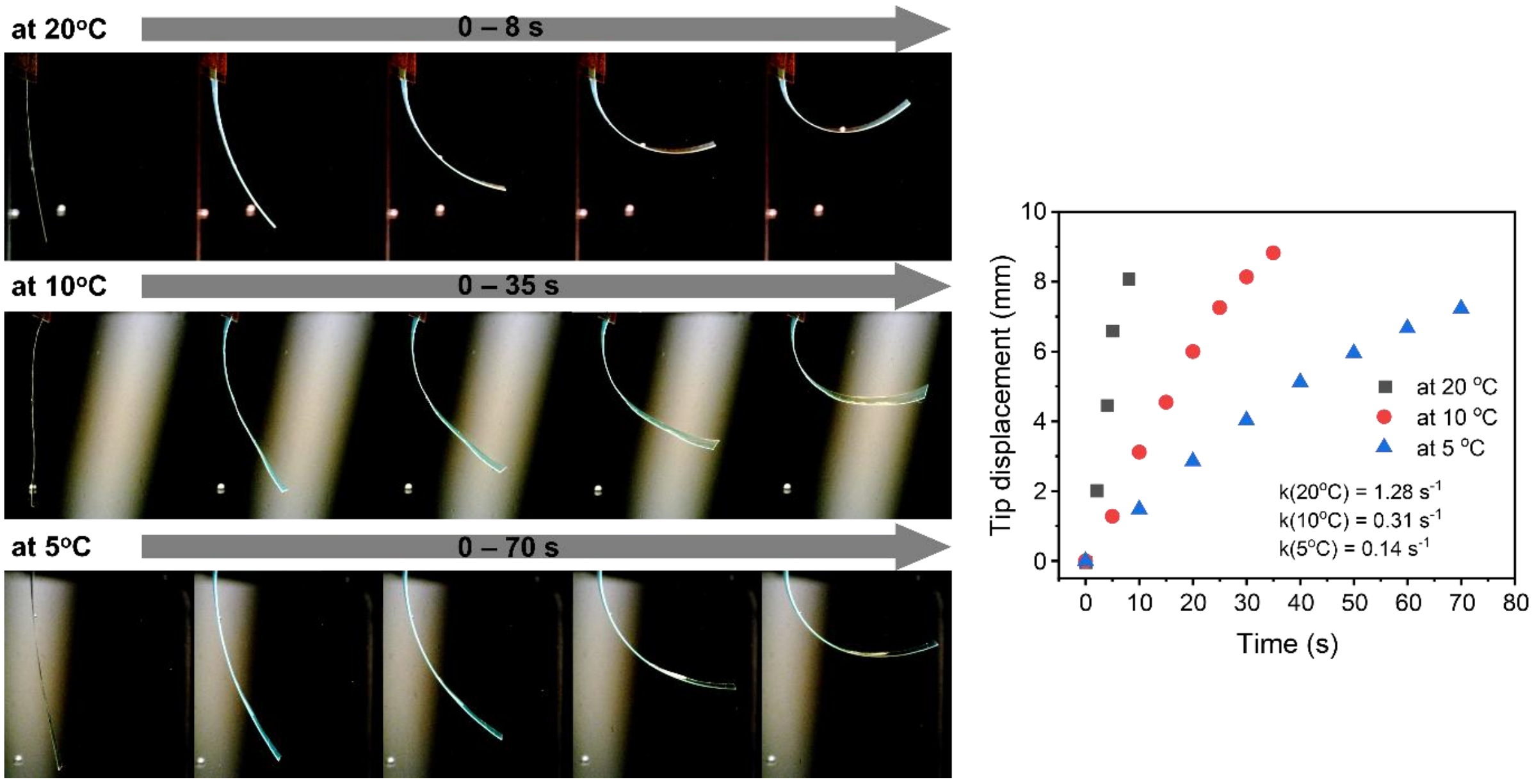

Fig. R9. Bending behaviour of the CHO-L polymer ribbon operating under water of decreasing temperature. Kinetic curved of the bending (at the bottom). Rate constants of the process are provided in the graph.

Comment 4. For the actuation tests in Figure 3, a film size of 1 cm*1 mm was used. How would varying the aspect ratio—either decreasing or increasing it—affect the response speed and magnitude of actuation? Given that sample dimensions often play a critical role in macroscopic deformation, further discussion on size effects would be valuable.

Answer: It is important to note that the mechanical stress generated by the material is independent of the sample aspect ratio, as stress is defined as force per unit area. To further eliminate potential size effects, all reported mechanical forces have been normalized per gram of motor cores or per one molecular motor core (Table 2), ensuring that comparisons are meaningful across samples.

In this study, we focused on ribbons with a high aspect ratio, which allows for predictable, robust bending-type actuation. Lower aspect ratio samples tend to resist simple bending due to inherent Poisson effects and edge constraints, often resulting in more complex deformations such as twisting, buckling, or saddle-shaped geometries. While these effects are interesting, exploring them would deviate from the primary focus of this work, which is to establish the correlation between molecular motor design and controlled photomechanical response.

Comment 5. The films were pre-stretched by 1% prior to testing. What is the rationale for selecting this specific pre-strain level? Further justification and explanation regarding the determination of this 1% pre-stretch would strengthen the methodological clarity of the study.

Answer: Based on literature, a pre-strain in the range of 1–5% is generally sufficient to ensure that the sample is taut and that the load cell operates within a regime sensitive enough to detect small force fluctuations. Importantly, applying a low pre-strain also keeps the network

within its linear elastic regime, avoiding any nonlinear deformation that could complicate data interpretation.

In our experiments, after applying the 1% pre-strain, we allowed the stress to stabilize and ensured that no residual stress relaxation (tailing) remained. This procedure confirmed that the sample was mechanically equilibrated before measurement. The selected pre-strain of 1% thus satisfies all these criteria, providing reproducible and reliable measurements.

Following the reviewer's suggestion, we have clarified these reasons in page 8.

Reviewer #3:

The manuscript "Efficient Molecular Motors in Liquid Crystal Networks: Integrating Fluorescence with Large Opto-mechanical Effects" by Ryabchun, Feringa, and co-authors describes an integration of liquid crystal polymer network with diverse molecular motor-containing photoswitchable motifs.

In previous literature, such systems were already demonstrated (which is properly acknowldedged in references). However, the novelty of the current approach is the comparison between diverse photochromic units, and indication of structural factors that enhance the efficiency of light energy conversion into mechanical force and macroscopic effects. Here is, where the current manuscript increases the general understanding, overcomes earlier reports which described single systems, and offers design principles, which altogether constitute its significance to the field.

The experiments properly support the conclusions and claims, the methodology is sound, and the experimental data complete and reproducible.

Answer: We thank reviewer for high evaluation of our work and for valuable suggestions.

Comment 1. in the description of LCPN preparation (line 114, page 4): "In order to reduce the elastic modulus of the LCPN, a non-polymerizable low molar mass LC mixture (E7) was also added" - could the authors clarify for non-experts, why does that additive reduce the elastic modulus (or, if this is a purely empirical observation, maybe a reference to the original sources of this observation can be added).

Answer: An addition of the non-polymerizable low molar mass liquid crystals into the networks softens them (reduces the glass transition temperature and consequently elastic modules). This approach is common in the field and also known as *plasticisation* in polymer science (and additives called plasticizers). In the case of LCPNs plasticizer should be liquid crystalline to perform its main function and not disturb liquid crystalline order.

We made this issue clearer in the revised manuscript (page 4) and supply relevant references [*Nat.Commun., 2019,* 10, 4819; *Adv. Mater. Interfaces*, 2025, *12*(9), 2400812].

Comment 2. in table 1, (line 193, page 6), and in the text, the motors decorated with aldehydes ("CHO") have consistently higher quantum yields of photoisomerization, than the motors decorated with cyano groups ("CN"). Was that phenomenon already known before for such types of a motor? If so, could you indicate explicitly in which references is it discussed? If not, then could you add a broader discussion on what might be the reasons for such a considerable difference?

Answer: In our previous study we rationalized the effect of the aldehyde groups (Nat. Chem. 2024, 16(8), 1330-1338) however the role of cyano groups is not fully understood. More details discussion can be found in page 11 of this response (our answer to the Comment 1 of the Referee 2). This information has been added to the revised manuscript (page 12).

Comment 3. in line 275 (page 8) - a typo: "previously" instead of "previous"

Answer: It has been corrected.

Comment 4. in lines 280-285 - the authors notice that the photogenerated force of LCPNs generated with CN-motors is higher than that with CHO-motors, even though the CHO-motors have significantly higher quantum yield. Their argument is the larger dipole moment of the -CN group versus -CHO and its role in increasing coupling with other components of the network. While the difference in dipole moments in favor of the -CN group is a fact, I am not convinced of the relation that the authors propose - I don't understand what exact interactions should better transfer the light-induced force from the CN-motor to the surrounding network, than from the CHO-motor. Is this supported by any observable or calculation? Or could it be empirically proven, e.g. by measuring the photogenerated forces in the LCPNs doped with more (or less) of the E7 (an additive which is claimed to influence the elastic modulus/stiffness of the material)? If the LCPNs are more stiff (e.g. without E7), than the advantage of the CN-motors should be more visible. And if the LCPNs are less stiff (e.g. with more E7 as originally), then the advantage of CN-motors should be lost in favor of CHO-motors which convert light into movement more efficiently (Q.Y. from the Table 1) than the CN-motors at the single molecule level.

Answer: We appreciate referee to bringing this aspect of our work up, and for the useful insight. We agree that the initial explanation based solely on dipole moment differences was insufficiently supported. To address this, we performed additional experiments and calculations to directly probe the alignment of the motors within the liquid crystalline polymer networks (LCPNs).

We prepared uniaxially aligned thin films (~2 μm) of CN-L-C6 and CHO-L and quantified molecular orientation using polarized light UV-vis spectroscopy. By measuring the absorbance as a function of the angle between the polarization direction and the liquid crystal director, we constructed polar plots of the $S_0 \rightarrow S_1$ transition. The orientation of the corresponding transition dipole moments (TDMs) was independently obtained from TD-DFT calculations (ωB97X-D/def2-TZVPP, CPCM acetonitrile), confirming the assignment of the absorption bands and its location in respect to the chemical structure (Fig. R10).

From these measurements, we determined the linear dichroism parameter (*D*), which provides a quantitative measure of molecular alignment. We found that the CN-based motor exhibits significantly higher orientational order compared to the CHO analogue ($D \approx 0.37$ vs. 0.22).

Given that the photogenerated force arises from directional molecular contraction along the motor axis, the degree of alignment directly determines how effectively individual molecular motions are translated into macroscopic stress. In this context, the higher degree of alignment of the CN motors leads to a more cooperative and directional force generation within the network, which explains the larger measured photogenerated forces despite their lower quantum yield.

We acknowledge the reviewer's suggestion regarding modulation of network stiffness (e.g., via E7 content) as a valuable approach to further probe structure-property relationships. While such experiments would provide additional insight, they would also introduce another variable into the system and fall beyond the scope of the present study, which is designed to isolate the effect of molecular motor structure within a constant network framework.

These results and the corresponding discussion have been incorporated into the revised manuscript (page 9) and Supporting Information (Supplementary Fig. 45).

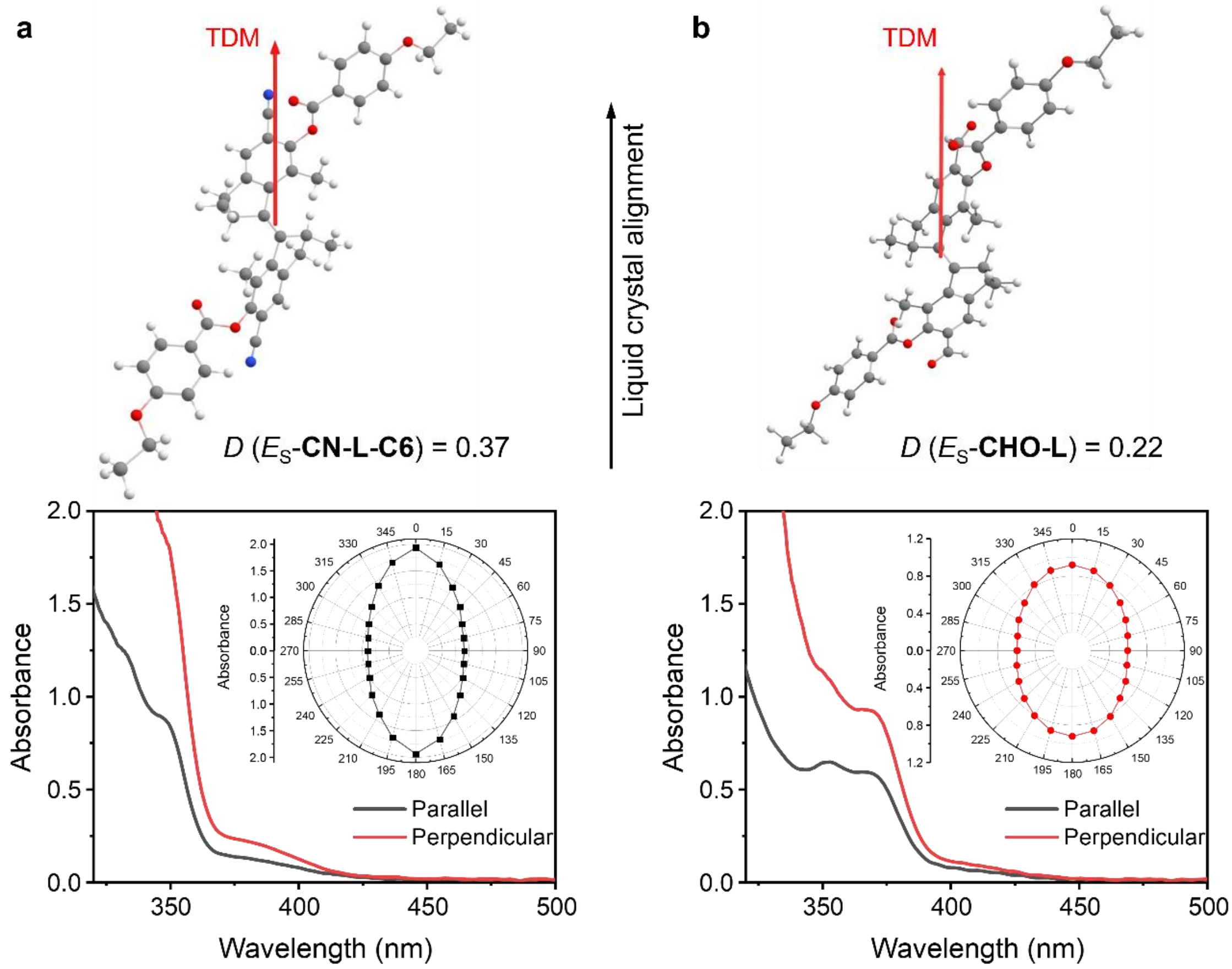

Fig. R10. Scheme demonstrating an alignment of molecular motor CN-L-C6 (**a**) and CHO-L (**b**) (DFT optimized geometry) with respect to the liquid crystals alignment. TDM – transition dipole moment. Polarized absorbance spectra of molecular motor in unidirectionally aligned LCPNs. Angular dependence as well as dichroism value (*D*) showed in the inserts.

Comment 5. line 346 (page 11) - the authors notice that the cyano and aldehyde motors in LCPN films exhibit "noticeable fluorescence". Are they also fluorescent in homogenous solutions? If not, where does the fluorescence come from in the LC polymer network? Are the motors so sterically confined?

Answer: Both CN- and CHO-motors are indeed fluorescent in homogeneous solution, with the CN-motor exhibiting approximately 4-fold higher emission intensity compared to the CHO-motor (Fig. R5). The fluorescence arises from the intrinsic electronic structure of the motors and is not solely a consequence of confinement within the polymer network.

In the liquid crystalline polymer networks (LCPNs), the fluorescence is similarly observed, with additional spectral features emerging due to network effects such as inner filter effects and, as we believe, specific interactions with the polymer matrix (Fig. R6 and R7).

A detailed discussion of these results is provided in the main text (page 12) and Supporting Information (Supplementary Fig. 47-49), as also summarized in our response to previous comments (pages 6-8 in this response).

Detailed Response to Reviewer Comments

"Efficient Molecular Motors in Liquid Crystal Networks: Integrating Fluorescence with Large Opto-mechanical Effects"

Research Article, No. NCOMMS-25-103033A

Reviewer #1:

This manuscript presents molecular-motor-based liquid crystal polymer networks with tunable substituents, rigid-core extension, and spacer length, and examines how these parameters affect actuation, photogenerated stress, modulus, and fluorescence. The comparative design is meaningful, and the integration of photomechanical response with fluorescence is potentially interesting for responsive soft materials. The revised manuscript has added more data, However, several central conclusions are currently stronger than the evidence supports. I therefore recommend major revision.

Answer: We thank the reviewer for the positive assessment of our molecular design and for acknowledging the value of the integration of photomechanical and fluorescence responses. We appreciate the feedback regarding the strength of our conclusions. In this revised version, we have systematically moderated our language throughout the manuscript and added further clarification to ensure that our interpretations are strictly aligned with the experimental evidence and supporting data provided. We believe these refinements have resulted in a more balanced and scientifically rigorous presentation of our results.

Comment 1. The manuscript argues that force generation is mainly photochemical in origin, but this distinction is not yet established rigorously enough. The added thermal imaging is helpful, yet the current analysis still does not fully separate photochemical and photothermal contributions across the different systems. The corresponding claims should be better supported or stated more cautiously.

Answer: We appreciate that referee pointed out the inherent difficulty of decoupling photochemical and photothermal contributions in light-responsive LCPNs.

We have supported the argument for a predominantly photochemical mechanism by highlighting three key lines of experimental evidence: i) As shown in the force curves (Fig. 3), the photogenerated stress persists after the light source is removed. While photothermal effects dissipate almost instantaneously upon switching off illumination, the persistent (long-lived) stress observed here is a hallmark of the photochemical accumulation of the motor's metastable states. ii) Control experiments provided in Fig. S42. To physically suppress temperature rise, we performed actuation experiments in a water-cooled environment. The retention of significant actuation force under these conditions strongly supports its photochemical origin. iii) Most of our characterization was performed at very low light intensities (~2 mWcm^{-2}). As shown in our IR thermography (Fig. S46), the temperature increase at these intensities is negligible, yet the mechanical response remains robust.

In Figure 3c,d, we intentionally used higher UV intensities to "force" a visible photothermal component. This allowed us to clearly distinguish between the fast thermal spike (which disappears immediately) and the steady photochemical build-up. This comparison demonstrates that at the standard intensities used elsewhere in the study, the thermal contribution is minimal.

As suggested, we have revised the text throughout (see pages 8) to state these findings more conservatively, replacing absolute claims with phrasing such as "predominantly photochemical" in origin and "photothermal contributions remain secondary and at most being minor effects at the intensities studied."

Comment 2. The proposed link between molecular design, alignment, stiffness, and force generation is plausible, but still only partly demonstrated. The added dichroism data improve the argument, although the evidence remains somewhat indirect and limited in scope. Stronger network-level support would be needed for the broader mechanistic claims.

Answer: We agree with the referee that establishing a universal, quantitative link between molecular architecture and macroscopic network mechanics is a profound challenge in the field of responsive polymer materials. However, our work was specifically designed as a controlled comparative study to isolate the structural influence of the molecular motor itself.

To ensure that the observed variations in force generation and modulus are directly attributable to the molecular design of the motors, we maintained a standardized polymer matrix and alignment protocol across all samples. This experimental control is crucial. It ensures that differences in performance arise from the motor's internal structural optimization (e.g. rigid-core extension, spacer length) rather than unintended variations in the network processing.

While we acknowledge that linear dichroism provides a molecular-level view, we believe that when combined with our macroscopic mechanical data (stress curves), it offers a consistent picture of how molecular motion translates to network performance.

As suggested, we have explicitly clarified the scope of our mechanistic claims in the revised manuscript (page 7-8). We now present our findings as a framework for understanding molecular efficiency within this optimized LCPN architecture, while noting that the absolute values of these relationships may shift in different polymer scaffolds. We have moderated the "broader mechanistic claims" to reflect this focused, comparative perspective.

Comment 3: The normalization of force to individual molecular units appears too assumption-dependent for the strong comparative conclusions currently made. These values should be presented more clearly as approximate estimates, and the claims of orders-of-magnitude superiority should be moderated.

Answer: We acknowledge the referee's concern regarding the assumptions inherent in normalizing macroscopic force to individual molecular units. While this normalization was intended to provide a molecular level perspective on macroscopic efficiency, we agree that the complexity of the network (e.g., variations in local environment, orientation distribution, and switching kinetics) makes these values approximate estimates rather than absolute physical constants.

In response, we have implemented the following changes: i) In the revised manuscript (page 11, Table 2, and Fig. 3f), these values are now explicitly presented as "approximation estimates of force". We have added a subsection “Assumptions for molecular force estimation” to the Supplementary Information (Section 14) detailing the assumptions made. ii) We have removed the "orders-of-magnitude" terminology where it relied on these normalized values. iii) We have clarified that the utility of this normalization is to facilitate a qualitative comparison between different motor architectures within a similar matrix, rather than to define the absolute force of a single molecule.

We believe these revisions provide a more conservative and scientifically grounded comparison of the different motor systems and a realistic description of our findings.

Comment 4. The fluorescence data are interesting, but this part remains more descriptive than mechanistic. In particular, the origin of the longer-wavelength emission in the networks is still not fully resolved, and fluorescence-actuation coupling is not yet quantitatively established. This section should be strengthened or interpreted more conservatively.

Answer: We agree with referee that a full quantum-mechanical or photophysical elucidation of the emission origins would be of high interest. However, determining the precise mechanism (e.g., distinguishing between conformational changes, local environment polarity, or transition to aggregate-induced states) in a highly anisotropic, crosslinked polymer network is a significant undertaking that we believe goes well beyond the scope of this materials-centric study.

Our primary goal was to report the discovery and functional utility of this intrinsic fluorescence as a real-time, non-destructive readout for macroscopic actuation, a feature that is not reported so far for motorized polymer systems.

As suggested, we have revised the discussion (page 12) to be more cautious. We now explicitly state that the longer-wavelength emission represents an experimental observation and discuss several potential origins as working hypotheses.

We believe this approach maintains the integrity of our materials discovery while acknowledging the complex photophysical questions that remain for future specialized studies.

Comment 5. The comparison with azobenzene and the second-generation motor is informative, but it should be interpreted more carefully because the systems are tested under different excitation wavelengths. As such, the current data do not fully support broad claims of general superiority.

Answer: We agree with the reviewer that excitation wavelength is a critical parameter in photoresponsive systems. However, we believe that comparing these systems at their respective absorption maxima, while maintaining identical light intensities, provides the most meaningful assessment of their intrinsic performance.

Testing all systems at a single wavelength would be physically non-representative, as it would favour one chromophore while providing insufficient energy to another that does not absorb in that regime. By using the optimal wavelength for each, we are comparing the optimal near peak capability of each motor/switch within the standardized LCPN matrix.

To ensure a fair comparison, we kept the light intensity, motor loading, and polymer network composition constant. This allows us to attribute the differences in stress generation and modulus to the molecular architecture and its coupling with the network, rather than extrinsic factors.

We have taken referee's advice and revised the discussion in page 7.

Comment 6. The manuscript attributes the performance differences mainly to motor structure, but the influence of network composition is not fully separated. A small control study varying E7 content or motor loading in representative CN- and CHO-based networks would strengthen this conclusion.

Answer: We appreciate referee's suggestion to explore the influence of the network matrix. However, we believe that introducing variations in the E7 content or motor loading at this stage would shift the focus of the study away from the primary objective: isolating the impact of molecular motor architecture on mechanical work.

In experimental materials science, a standard approach to evaluate a series of molecular components is to maintain a constant environment. By using a single, standardized network composition for all samples (based on well-established literature protocols [21, 23-25, 27,41]), which are now mentioned in page 16, we ensure that the observed changes in stress, modulus, and fluorescence are purely a function of the molecular motor's structural modifications.

Varying E7 content or loading would introduce secondary effects, such as changes in the glass transition temperature (T_g), crosslinking density, and order parameters. These extrinsic factors would make it difficult, if not impossible, to clearly separate whether gain of performance is due to the motor's design or due to polymer network's mechanical state.

To address this point, we have added a clarifying statement in the discussion (page 7). We now acknowledge that while network composition and loading certainly play a role in absolute performance, the relative performance trends established here provide a clear and rigorous roadmap for motor-structure optimization within LCPN systems.

We believe that keeping the matrix constant is the most scientifically robust way to validate our comparative molecular design.
